# Synthesis of Canthin-4-ones and Isocanthin-4-ones via B Ring Construction

**DOI:** 10.1021/acs.joc.4c00440

**Published:** 2024-04-24

**Authors:** Maria Koyioni, Andreas Kourtellaris, Panayiotis A. Koutentis

**Affiliations:** Department of Chemistry, University of Cyprus, P.O. Box 20537, 1678 Nicosia, Cyprus

## Abstract

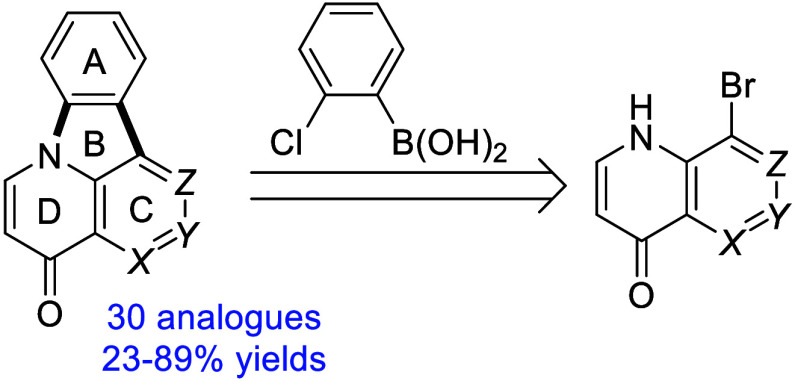

Canthin-4-one is synthesized via a six-step procedure starting from commercially available 3-amino-4-bromopyridine in 26% overall yield. 3-Amino-4-bromopyridine is initially converted to 8-bromo-1,5-naphthyridin-4(1*H*)-one. O-Methylation, intermolecular Pd-catalyzed C–C coupling, and demethylation afford the key intermediate, 8-(2-chlorophenyl)-1,5-naphthyridin-4(1*H*)-one, for which intramolecular C–N coupling completes the synthesis of the canthin-4-one skeleton. Ten canthin-4-one analogues were prepared in addition to the parent compound. With minor modifications, the synthesis also applies to the synthesis of two series of isocanthin-4-ones.

## Introduction

**Figure 1:**
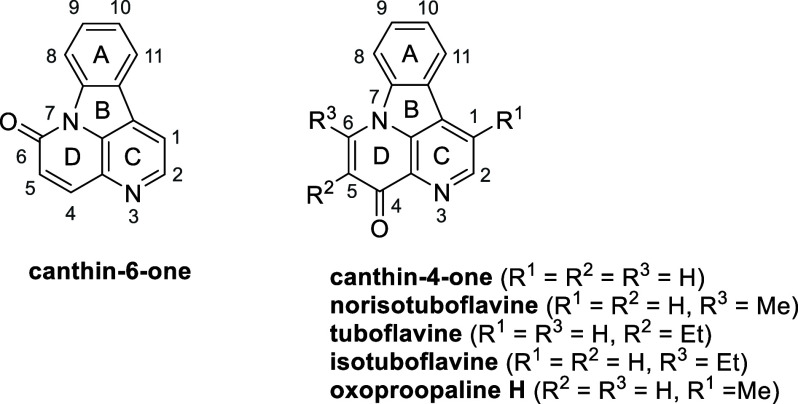
Structures of canthin-6-one and canthin-4-one and natural analogues of canthin-4-one.

Canthin-4-ones represent the lesser-known family of canthine alkaloids,^1^ the most well-known of which are the canthin-6-ones.^2^ More than 60 natural analogues of canthin-6-ones have been isolated to date, but only four natural analogues of canthin-4-ones are known: tuboflavine,^3^ norisotuboflavine,^4^ isotuboflavine,^4^ and oxoproopaline H^5^ (Figure 1). The latter was only recently isolated from a mutant ΔstnK4 of *Streptomyces flocculus* CGMCC4, and its structure elucidated by extensive one- and two-dimensional nuclear magnetic resonance (NMR).^5^

**Scheme 1:**
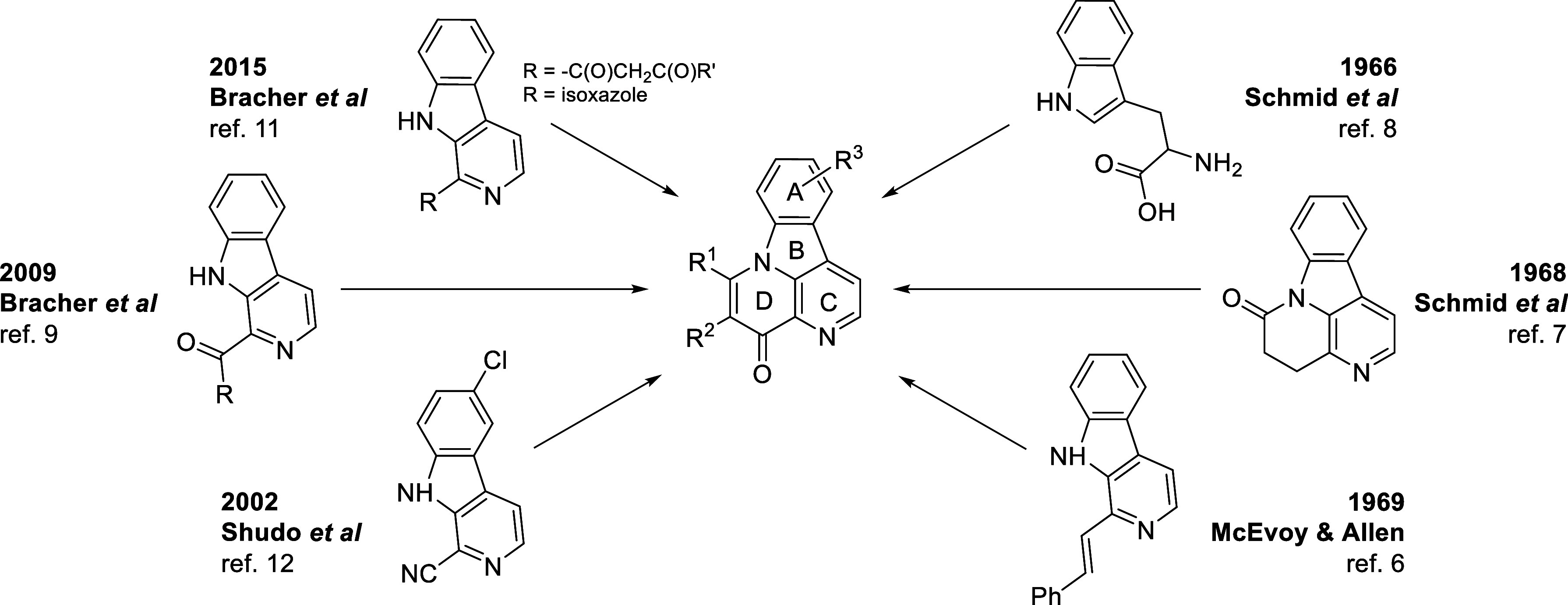
Literature Syntheses of Canthin-4-ones

Several syntheses for norisotuboflavine,^6^ isotuboflavine,^7^ and tuboflavine^8^ have been reported. In 1966, Schmid et al. reported a four-step synthesis for tuboflavine, starting from tryptophan in 0.3% overall yield,^7^ while in 1968, they reported the syntheses of norisotuboflavine and isotuboflavine,^6^ in three steps starting from 4,5-dihydrocanthin-6-one in <1% overall yield. An alternative five-step synthesis for norisotuboflavine was also reported by McEvoy and Allen, starting from the natural product benzalharman with an overall yield of <1%.^6^ More recently, in 2009, Bracher reported a one-step, moderate- to good-yielding synthesis by reaction of carbolines with Bredereck’s reagent.^9^ The same approach was used for the synthesis of 5-methoxycanthin-4-one,^10^ which was mistakenly identified as a new alkaloid named drymaritin isolated from *Drymaria diandra*,^11^ which was then confirmed to be the already known alkaloid cordatanine (4-methoxycanthin-6-one).^12^ In 2015, Bracher et al. reported two new methods for the syntheses of norisotuboflavine and tuboflavine.^13^ The first involved the reaction of 1-acetyl-β-carboline with either *N*-acetylbenzotriazole or *N*-propanoylbenzotriazole, which, via the *in situ* formation of 1,3-diketone intermediates, afforded in one step norisotuboflavine and isotuboflavine, in 34% and 16% yields, respectively. The second approach involved a four-step sequence starting from 1-bromo*-β*-carboline giving norisotuboflavine and isotuboflavine, in 50% and 47% yields, respectively. While these routes (Scheme 1) provided the natural products in yields higher than those of the prior syntheses, they lacked versatility. Multistep routes to synthetic canthin-4-ones starting from 9*H*-pyrido[3,4-*b*]indole-1-carbonitrile were also reported in the patent literature but with low overall yields.^14^ A high-yielding synthesis of chiral *S*-(−)-5,6-dihydrocanthin-4-ones was recently reported by Batra et al.^15^

While scarce, both natural and synthetic canthin-4-ones have potential in medicinal chemistry, with antimicrobial^16^ and phosphodiesterase-inhibitory^14^ activities reported to date. Nevertheless, due to the lack of good general syntheses, there is limited access to structurally diverse canthin-4-one libraries, which are needed to probe structure–activity relationships. In addition, all of the known routes are based on the construction of the D ring via β-carboline precursors.

**Scheme 2:**
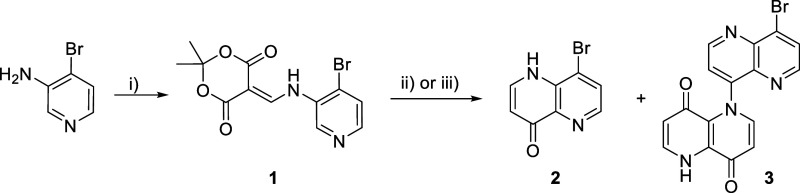
Synthesis of 8-Bromo-1,5-naphthyridin-4(1*H*)-one (**2**) Reagents and conditions: (i) Meldrum’s acid (1.1 equiv), CH(OEt)_3_ (2.0 equiv), MeCN, reflux, 1.75 h, 91%; (ii) Ph_2_O (0.08 mol/L), 250 °C, 8.5 min (**2**, 12%; **3**, 4%); (iii) Ph_2_O (0.03 mol/L), 250 °C, 0.5 min (**2**, 52%; **3**, 0%).

In 2017, we reported the synthesis of 3-deazacanthin-4-ones via construction of the B ring,^17^ starting from 8-haloquinol-4-ones using sequential Suzuki–Miyaura and C–N coupling reactions. This route allowed diversification of the A ring thanks to commercially accessible 2-halo(het)aryl boronic acids, esters, and trifluoroborates. The method was originally introduced by our group for the synthesis of canthin-6-ones.^18^ To apply this synthesis to canthin-4-ones, we needed access to unknown 8-bromo-1,5-naphthyridin-4(1*H*)-one (**2**) (Scheme 2). Our initial efforts to synthesize this scaffold via the thermolysis of the corresponding ylidene **1** gave only intractable tar. Herein, we report the successful thermolysis of aza-ylidene **1** and the subsequent synthesis of canthin-4-ones.

Furthermore, we extend the method to structurally related isocanthin-4-ones.

## Results and Discussion

Ylidene **1**, needed to access the desired 8-bromo-1,5-naphthyridin-4(1*H*)-one (**2**), was prepared according to literature procedures from commercially available 3-amino-4-bromopyridine in 91% yield. Initial attempts to thermolyze ylidene **1** under conditions analogous to those used for the synthesis of 8-bromoquinol-4-one **2** (∼0.61 mol/L at 220 °C, 5 min)^17^ led to the formation of intractable black solids. Dilution of the reaction mixture gave intractable black solids along with naphthyridinone **2** in 12% yield and side product **3** in 4% yield (Scheme 2).

The structural elucidation of side product **3** was not immediately obvious (see section S1 of the Supporting Information), but its formation suggested further dilution of the reaction mixture and/or shortening of the reaction time. After a series of trial-and-error experiments, the optimum concentration of the reaction was found, 0.03 mol/L, at ∼250 °C for 0.5 min. The reaction performed at a 1.83 mmol scale gave the desired naphthyridinone **2** in 52% yield. Attempts to improve this yield further were unsuccessful. Initial attempts to create an efficient C–C coupling at position C-8 of 8-bromonaphthyridinone **2** involved the reaction of naphthyridinone **2** with 2-chlorophenylboronic acid (1.5 equiv), using two sets of conditions: (i) Pd(dppf)Cl_2_·DCM (5 mol %), K_2_CO_3_ (2 equiv), and dioxane/H_2_O (75:25) or (ii) XPhos Pd G4 (2.5 mol %), K_2_CO_3_ (2 equiv), and MeOH. Both sets of conditions were investigated under conventional heating or microwave (MW) irradiation, under an air or Ar atmosphere, but failed to give a complete reaction [20–30% of the starting material consumed by thin layer chromatography (TLC)]. Tentatively, this was attributed to poisoning of the Pd catalyst with naphthyridinone **2** acting as a bidentate ligand to form complex **4**. Complexes of Pd with bidentate ligands with a similar structural motif are reported in the literature.^19^

**Scheme 3:**
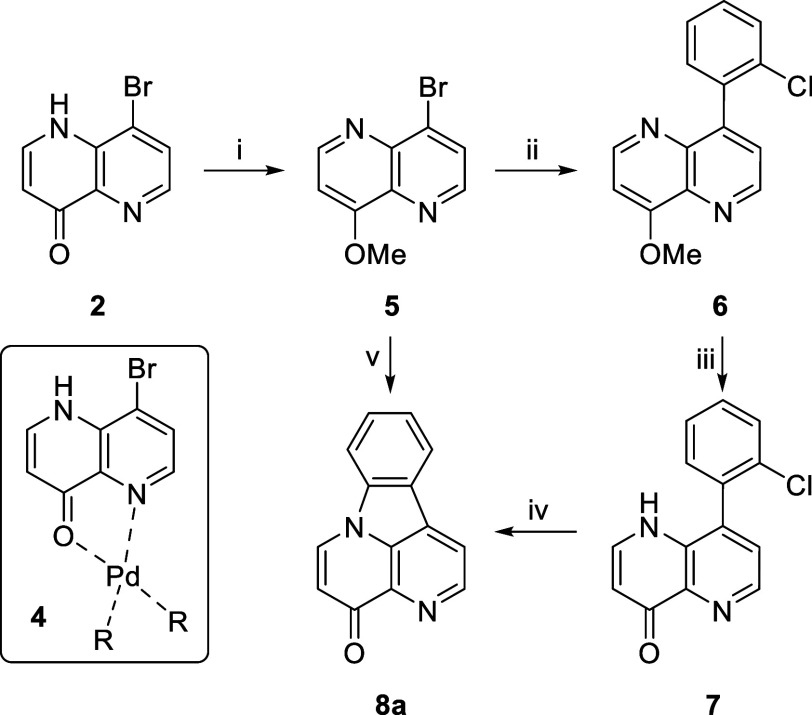
Synthesis of Canthin-4-one **8a** from 8-Bromo-1,5-naphthyridin-4(1*H*)-one (**2**) Reagents and conditions: (i) MeI (1.2 equiv), K_2_CO_3_ (1.2 equiv), DMF, ∼20 °C, 16 h, 70%; (ii) 2-ClC_6_H_4_B(OH)_2_ (1.5 equiv), K_2_CO_3_ (2 equiv), Pd(dppf)Cl_2_·DCM (1.25 mol %), dioxane/H_2_O (75:25), ∼88 °C, 8 min, 74%; (iii) concentrated HCl/H_2_O (50:50), dioxane, ∼101 °C, 40 min, 96%; (iv) K_2_CO_3_ (2 equiv), CuI (10 mol %), *N*,*N*′-dimethyl-*trans*-1,2-cyclohexanediamine (DMCDA) (20 mol %), dioxane/H_2_O (75:25), ∼88 °C, 5.5 h, 76%; (v) (a) 2-ClC_6_H_4_B(OH)_2_ (1.5 equiv), K_2_CO_3_ (2 equiv), Pd(dppf)Cl_2_·DCM (1.25 mol %), dioxane/H_2_O (75:25), ∼88 °C, 12 min; (b) concentrated HCl, dioxane/H_2_O (75:25), ∼88 °C, 2 h; (c) K_2_CO_3_ (10 equiv), CuI (10 mol %), DMCDA (20 mol %), dioxane/H_2_O (75:25), ∼88 °C, 12 h, 80%.

To resolve this, O-methylated analogue **5** was prepared using MeI and K_2_CO_3_ in DMF (Scheme 3). Gratifyingly, the reaction of methoxynaphthyridine **5** with 2-chlorophenylboronic acid (1.5 equiv), Pd(dppf)Cl_2_·DCM (5 mol %), and K_2_CO_3_ (2 equiv) in a dioxane/H_2_O (75:25) solvent heated at reflux gave the desired product **6** in high yield. The loading of the catalyst could be decreased to 1.25 mol % without any detrimental effect on the reaction time or yield. O-Demethylation of methoxynaphthyridine **6** using aqueous HCl in dioxane gave **7**, and subsequent C–N coupling gave the desired canthin-4-one **8a** (76%) (18% over seven steps, starting from commercial aminopyridine). The last three steps (Suzuki coupling, demethylation, and C–N coupling) were telescoped, affording canthin-4-one **8a** in an improved yield of 80% versus 54% over the three steps, and a 26% overall yield from 3-amino-4-bromopyridine.

With a successful synthesis for canthin-4-one **8a**, we applied this method to synthesize isocanthin-4-ones. Translocating the nitrogen atom in the C ring can give two possible isocanthin-4-ones, **9a** and **10a**. Of these, only isocanthin-4-one **9a** and four of its derivatives have appeared in a patent.^14^

**Scheme 4:**
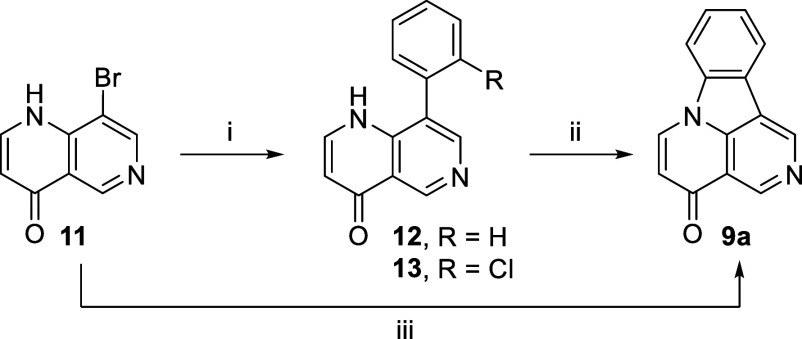
Synthesis of Isocanthin-4-one **9a** Reagents and conditions: (i) 2-ClC_6_H_4_B(OH)_2_ (1.5 equiv), Pd(dppf)Cl_2_·DCM (5 mol %), K_2_CO_3_ (2 equiv), dioxane/H_2_O (75:25), MW (*T* = 120 °C, 150 W, ∼50 psi), 45 min; (ii) K_2_CO_3_ (2 equiv), CuI (10 mol %), DMCDA (20 mol %), dioxane/H_2_O (75:25), MW (*T* = 120 °C, 150 W, ∼50 psi), 6 h; (iii) (a) 2-ClC_6_H_4_B(OH)_2_ (1.5 equiv), Pd(dppf)Cl_2_·DCM (5 mol %), K_2_CO_3_ (2 equiv), dioxane/H_2_O (75:25), MW (*T* = 120 °C, 150 W, ∼50 psi), 45 min; (b) K_2_CO_3_ (2 equiv), CuI (10 mol %), DMCDA (20 mol %), dioxane/H_2_O (75:25), MW (*T* = 120 °C, 150 W, ∼50 psi), 6 h, 69%.

As in the case of canthin-4-one, the literature synthesis of isocanthin-4-one **9a** involves the construction of the D ring. Fortunately, the required naphthyridinone **11** needed for the synthesis of isocanthin-4-one **9a** via our approach was known.^20^ The Suzuki–Miyaura coupling of naphthyridinone **11** with 2-chlorophenylboronic acid, using our standard conditions, led to considerable unreacted starting material (TLC). Increasing the Pd loading from 5 to 25 mol % or performing the reaction under MW radiation^20^ led to a complete reaction; however, in both cases, product **13** was obtained as a mixture with protodechlorinated 8-phenylnaphthyridinone **12**, which was difficult to separate (Scheme 4). Protodechlorinated product **12** was synthesized in pure form by reaction of the naphthyridinone with phenylboronic acid, which allowed its full characterization (see section S2). Interestingly, with phenylboronic acid the reaction was performed successfully under conventional heating and 5 mol % Pd(dppf)Cl_2_·DCM giving the desired product in 71% yield. Further development of the reaction with 2-chlorophenylboronic acid was pursued using the MW conditions, owing to the lower catalyst loading and cleaner reaction mixture observed under these conditions. The C–N coupling of key intermediate **13** was also performed under MW radiation. The two-step reaction sequence, Suzuki–Miyaura coupling followed by C–N coupling, was telescoped to afford the desired isocanthin-4-one **9a** in 69% yield.

The telescoped reaction was also performed on a larger scale using conventional heating conditions and an increased level of the Pd catalyst (25 mol %) to give the desired isocanthin-4-one **9a** in 60% yield.

In a similar manner, 8-bromo-1,7-naphthyridin-4(1*H*)-one (**15**) could give us access to isocanthin-4-one **10a**. Naphthyridinone **15** was not reported in the literature, and like naphthyridinone **2**, our initial attempts to thermolyze ylidene **14** led to complex mixtures. Nevertheless, applying our “optimized” high-dilution thermolysis conditions described above to ylidene **14** gave the desired product **15** in 85% yield.

**Scheme 5:**
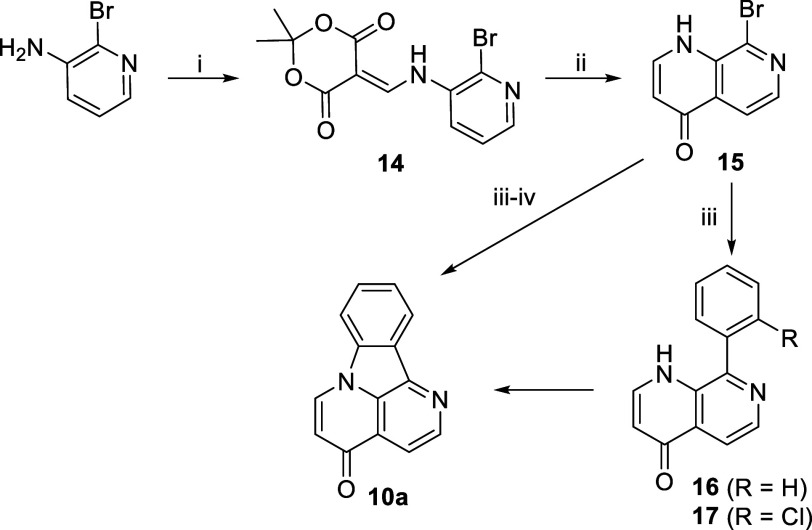
Synthesis of Isocanthin-4-one **10a** Reagents and conditions: (i) Meldrum’s acid (1.1 equiv), CH(OEt)_3_ (1.2 equiv), MeCN, ∼82 °C, 5 h, 75%; (ii) Ph_2_O, ∼250 °C, 0.5 min, 85%; (iii) 2-ClC_6_H_4_B(OH)_2_ (1.5 equiv), Pd(dppf)Cl_2_·DCM (5 mol %), K_2_CO_3_ (2 equiv), dioxane/H_2_O (75:25), ∼88 °C, 15 min; (iv) K_2_CO_3_ (2 equiv), CuI (10 mol %), DMCDA (20 mol %), H_2_O (1 equiv), dioxane/H_2_O (75:25), ∼88 °C, 12 h, 77%.

Our standard Suzuki–Miyaura coupling conditions worked well for this analogue giving a complete reaction within 10 min, without the need for MW irradiation or increased Pd loading. As in the case of naphthyridinone **11**, the reaction led to a difficult to separate mixture of the desired product **17** and protodechlorinated side product **16**. Performing the C–N coupling in one pot gave the desired isocanthin-4-one **10a** in 77% yield (Scheme 5).

With three optimized procedures in hand for the synthesis of canthin-4-one **8a** and isocanthin-4-ones **9a** and **10a**, a variety of 2-chlorophenylboronic acids were subjected to our optimized reaction conditions (Table 1). Sterically hindered 6-substituted (6-MeO and 6-Cl) 2-chlorophenylboronic acids failed in the Suzuki–Miyaura coupling. Both electron-releasing and -withdrawing substituents were tolerated in the synthesis of canthinones **8** and isocanthinones **10**. In the synthesis of isocanthinones **9**, the reaction was more sensitive to electron-withdrawing substituents, which afforded lower yields. In total, 28 new substituted analogues were prepared.

**Table 1:**
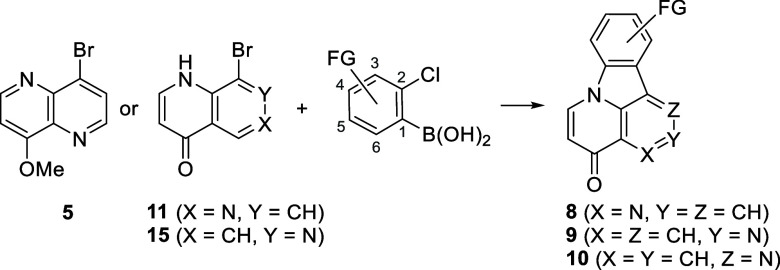
Analogues of Canthin-4-one **8** and Isocanthin-4-ones **9** and **10**

entry	condition^a^	ArB(OH)_2_ (equiv)	FG^b^	first step (min)	yield (%)
1	A	1.5	4-Me	10	**8b** (74)
2	A	1.5	4-MeO	10	**8c** (35)
3	A	3.0	4-F_3_C	10	**8d** (67)
4	A	1.5	4-Cl	20	**8e** (35)
5	A	1.5	4-F	10	**8f** (89)
6	A	1.5	5-MeO	10	**8g** (69)
7	A	3.0	5-F_3_CO	10	**8h** (67)
8	A	3.0	5-F_3_C	20	**8i** (63)
9	A	3.0	5-Cl	10	**8j** (61)
10	A	1.5	5-F	10	**8k** (63)
11	B	1.5	4-Me	45	**9b** (88)
12	B	3.0	4-MeO	45	**9c** (68)
13	B	3.0	4-F_3_C	45	**9d** (49)
14	B	1.5	4-F	45	**9e** (36)
15	B	1.5	5-MeO	45	**9f** (85)
16	B	3.0	5-F_3_CO	45	**9g** (26)
17	B	1.5	5-F	45	**9h** (23)
18	C	1.5	3-Cl	10	**10b** (47)
19	C	1.5	4-Me	15	**10c** (78)
20	C	1.5	4-MeO	5	**10d** (81)
21	C	3.0	4-F_3_C	50	**10e** (47)
22	C	3.0	4-Cl	12	**10f** (71)
23	C	1.5	4-F	60	**10g** (67)
24	C	1.5	5-MeO	15	**10h** (66)
25	C	1.5	5-F_3_CO	20	**10i** (62)
26	C	3.0	5-Cl	10	**10j** (65)
27	C	1.5	5-F	6	**10k** (70)

a Condition A (one pot): **5** (1 equiv), PdCl_2_(dppf)·DCM (1.25 mol %), K_2_CO_3_ (2 equiv), dioxane/H_2_O (75:25), ∼88 °C; (ii) concentrated HCl, ∼88 °C, 2 h; (iii) K_2_CO_3_ (10 equiv), CuI (10 mol %), DMCDA (20 mol %), ∼88 °C, 12 h. Condition B (one pot): (i) **11** (1 equiv), PdCl_2_(dppf)·DCM (1.25 mol %), K_2_CO_3_ (2 equiv), dioxane/H_2_O (75:25), MW (150 W, *T* = 120 °C, ∼60 psi), 45 min; (ii) K_2_CO_3_ (2 equiv), CuI (10 mol %), DMCDA (20 mol %), MW (150 W, *T* = 120 °C, ∼60 psi), 6 h. Condition C (one pot): (i) **15** (1 equiv), PdCl_2_(dppf)·DCM (5 mol %), K_2_CO_3_ (2 equiv), dioxane/H_2_O (75:25), ∼88 °C; (ii) K_2_CO_3_ (2 equiv), CuI (10 mol %), DMCDA (20 mol %), ∼88 °C, 12 h.

b Functional group.

## Conclusions

A new and efficient method for accessing canthin-4-ones via the construction of the B ring was developed. The parent canthin-4-one was prepared in six steps from commercially available 3-amino-4-bromopyridine, with the last three steps telescoped in one pot (26% overall yield). A key step was the synthesis of the previously unknown 8-bromo-1,5-naphthyridin-4(1*H*)-one (**2**). Suzuki–Miyaura coupling with commercially available 2-chlorophenylboronic acids followed by Cu-catalyzed C–N coupling afforded the canthin-4-one skeleton. The method was general with 10 analogues prepared in good yields (35–89%). The methodology was also applied for the synthesis of two aza isomers of the canthin-4-one. In this way, a library of 19 isocanthin-4-ones was also prepared (23–88% yields).

## Experimental Section

### General Methods and Materials

All chemicals were commercially available except those whose synthesis is described. All volatiles were removed under reduced pressure. The DCM/NH_3_ solvent mixture was prepared by extracting aqueous NH_4_OH (∼500 mL) with DCM (4 × 500 mL). The DCM layers were combined, dried (Na_2_SO_4_), filtered, and stored in an amber glass bottle. Dioxane was distilled from CaH_2_ before use. The combined DCM extracts were dried (Na_2_SO_4_), filtered, and stored in a glass bottle. All microwave experiments were performed in a CEM Discover Microwave Reactor with an external surface sensor in sealed vials. For conventional heating of the reaction mixtures, aluminum heating blocks were used. All reaction mixtures and column eluents were monitored by TLC using commercial aluminum-backed TLC plates (Kieselgel 60 F_254_). The plates were observed under ultraviolet (UV) light at 254 and 365 nm. The technique of dry flash chromatography was used throughout for all non-TLC scale chromatographic separations using silica gel 60 (<0.063 mm).^21^ Melting points were determined using a PolyTherm-A, Wagner & Munz, Kofler hot-stage microscope apparatus or using a TA Instruments DSC Q1000 instrument with samples hermetically sealed in aluminum pans under an argon atmosphere [using heating rates of 5 °C/min (DSC mp listed by onset and peak values)]. Samples were purified by bulk recrystallization from hot, filtered saturated solutions slowly cooled to room temperature (unless otherwise stated). Recrystallization solvents are listed after melting points. UV–visible spectra were recorded using a Shimadzu UV-1900 spectrophotometer, and inflections are identified by the abbreviation “inf”. Infrared spectra were recorded on a Shimadzu FTIR-NIR Prestige-21 spectrometer fitted with a Pike Miracle Ge ATR accessory, and strong, medium, and weak peaks are represented by s, m, and w, respectively. ^1^H and ^13^C NMR spectra were recorded, as indicated, on a Bruker Avance 300 machine at 300 and 75 MHz, respectively, or on a Bruker Avance 500 machine at 500 and 125 MHz, respectively. Deuterated solvents were used for homonuclear lock, and the signals are referenced to the deuterated solvent peaks. Attached proton test (APT) NMR studies were used for the assignment of the ^13^C peaks C (quaternary), CH_2_, CH_3_, and CH_4_ as C (s), C (d), C (t), and C (q), respectively. Mass spectrometry data were recorded with a matrix-assisted laser desorption/ionization time of flight (MALDI-TOF) mass spectrometer (positive mode) on a Bruker Autoflex III Smartbeam instrument or with the Agilent 1260 Infinity II Preparative LC/MSD System. Elemental analysis was performed using a Euro-Vector EA3000 CHN elemental analyzer. 8-Bromo-1,6-naphthyridin-4(1*H*)-one (**11**) was prepared according to a literature procedure (see the Supporting Information).^20^

### Synthesis of Meldrum’s Acid Ylidenes

#### 5-{[(4-Bromopyrid-3-yl)amino]methylene}-2,2-dimethyl-1,3-dioxane-4,6-dione (**1**)

To a solution of 3-amino-4-bromopyridine (3.06 g, 17.7 mmol) in MeCN (50 mL) were added Meldrum’s acid (2.93 g, 20.3 mmol) and CH(OEt)_3_ (6.0 mL, 36.0 mmol), and the mixture was heated to ∼82 °C (reflux) for 1.75 h. Then the solvent was removed in vacuo, and the residue was recrystallized to give compound **1** (5.79 g, 91%) as beige-pink needles: mp (hot stage) 161.5–163.0 °C (EtOH); mp (DSC) onset 164.61 °C, peak max 165.25 °C (EtOH); *R*_*f*_ = 0.48 (80:20 DCM/*t*-BuOMe); λ_max_ (DCM, nm) 267 inf (log ε = 3.85), 271 inf (3.87), 290 inf (4.08), 300 inf (4.17), 326 (4.41); *v*_max_ (ATR, cm^–1^) 1730m (C=O), 1678m, 1607s, 1555m, 1433m, 1317m, 1312m, 1269s, 1234m, 1221m, 1206m, 1184m, 1145m, 1065m, 1018m, 1009m, 935m, 891w, 853w, 816m, 799m, 787m, 752w, 727m; ^1^H NMR (CDCl_3_, 500 MHz) δ 11.56 (1H, d, *J* = 13.0 Hz), 8.683 (1H, s), 8.678 (1H, d, *J* = 13.5 Hz), 8.32 (1H, d, *J* = 5.5 Hz), 7.62 (1H, d, *J* = 5.5 Hz), 1.77 (6H, s); ^13^C{^1^H} NMR (CDCl_3_, 125 MHz) δ 165.4 (s), 163.0 (s), 152.0 (d), 147.8 (d), 139.1 (d), 134.2 (s), 128.2 (d), 124.6 (s), 105.8 (s), 90.1 (s), 27.4 (q); *m*/*z* (MALDI-TOF) 329 (^81^Br, MH^+^, 31%), 327 (^79^Br, MH^+^, 30), 297 (23), 271 (90), 269 (100), 227 (71), 225 (82). Anal. Calcd for C_12_H_11_BrN_2_O_4_: C, 44.06; H, 3.39; N, 8.56. Found: C, 44.01; H, 3.47; N, 8.43.

#### 5-{[(2-Bromopyrid-3-yl)amino]methylene}-2,2-dimethyl-1,3-dioxane-4,6-dione (**14**)

To a solution of 3-amino-2-bromopyridine (1.2 g, 6.8 mmol) in anhydrous MeCN (25 mL) were added Meldrum’s acid (1.07 g, 7.5 mmol) and CH(OEt)_3_ (1.4 mL, 8.2 mmol), and the mixture was heated to ∼82 °C (reflux) for 5 h. The solvent was evaporated in vacuo, and the solid residue was recrystallized to give compound **14** (1.66 g, 75%) as pale pink needles: mp (hot stage) 170.6–172.0 °C (EtOH); mp (DSC) onset 173.6 °C, peak max 173.9 °C (EtOH); decomposition (DSC) onset 191.6 °C, peak max 196.4 °C; *R*_*f*_ = 0.50 (90:10 DCM/*t*-BuOMe); λ_max_ (DCM, nm) 287 inf (log ε = 4.27), 292 (4.31), 335 (4.63); *v*_max_ (ATR, cm^–1^) 1721m (C=O), 1686m, 1618m, 1560m, 1479w, 1445m, 1400s, 1379m, 1319m, 1275m, 1227m, 1204m, 1146w, 1051m, 1022m, 1009w, 991m, 930m, 893w, 847w, 799m, 785m, 763w, 731m; ^1^H NMR (CDCl_3_, 500 MHz) δ 11.62 (1H, br d, *J* = 13.5 Hz), 8.60 (1H, d, *J* = 13.5 Hz), 8.28 (1H, dd, *J* = 5.0, 1.5 Hz), 7.69 (1H, dd, *J* = 8.0, 1.5 Hz), 7.40 (1H, dd, *J* = 8.0, 4.5 Hz), 1.77 (6H, s); ^13^C{^1^H} NMR (125 MHz, CDCl_3_) δ 165.1 (s), 163.2 (s), 151.4 (d), 147.1 (d), 134.4 (s), 134.2 (s), 124.1 (d), 124.0 (d), 105.8 (s), 90.4 (s), 27.4 (q); *m*/*z* (ESI) 329 (^81^Br, MH^+^, 42%), 327 (^79^Br, MH^+^, 44%), 303 (98%), 301 (100%). Anal. Calcd for C_12_H_11_BrN_2_O_4_: C, 44.06; H, 3.39; N, 8.56. Found: C, 43.97; H, 3.45; N, 8.26.

### Synthesis of 8-Halonaphthyridinones

#### 8-Bromo-1,5-naphthyridin-4(1*H*)-one (**2**) (optimized conditions)

Ph_2_O (60 mL) was heated to ∼250 °C (external temperature), and 5-{[(4-bromopyrid-3-yl)amino]methylene}-2,2-dimethyl-1,3-dioxane-4,6-dione (**1**) (600 mg, 1.83 mmol) was added in one portion. After 0.5 min, the reaction mixture was immediately removed from the heat source (metal heating block) and after 1 min poured into cold *n*-hexane (−40 °C) (1000 mL). The mixture was cooled to approximately −40 °C for 2 h, and the precipitate was filtered, washed with *n*-pentane, and recrystallized to give compound **2** (214.8 mg, 52%) as beige microcrystalline powder: mp (hot stage) 158.0–160.0 °C (PhH); *R*_*f*_ = 0.13 (82:18 DCM-NH_3_/EtOH); λ_max_ (EtOH, nm) 205 (log ε = 4.53), 234 inf (4.19), 241 (4.34), 248 (4.34), 273 inf (3.81), 279 (3.80), 287 inf (3.74), 321 inf (3.86), 333 inf (4.00), 341 (4.03); ν_max_ (ATR, cm^–1^) 3200–2800w br (NH), 1620m (C=O), 1603m, 1572m, 1551m, 1504s, 1435w, 1400m, 1290m, 1240w, 1209m, 1198m, 1121w, 1084w, 1065w, 898w, 852w, 826m, 810m, 787m; ^1^H NMR (CD_3_OD, 500 MHz) δ 8.52 (1H, d, *J* = 5.0 Hz), 8.05–8.03 (2H, m), 6.55 (1H, d, *J* = 7.0 Hz); ^13^C{^1^H} NMR (CD_3_OD, 125 MHz) δ 178.4 (s), 147.8 (d), 142.6 (d), 141.7 (s), 137.3 (s), 131.8 (d), 125.8 (s), 113.1 (d); *m*/*z* (ESI) 249 (^81^Br, MH^+^ + Na, 100%), 247 (^79^Br, MH^+^ + Na, 93%), 227 (^81^Br, MH^+^, 55%), 225 (^79^Br, MH^+^, 55%). Anal. Calcd for C_8_H_5_BrN_2_O: C, 42.70; H, 2.24; N, 12.45. Found: C, 42.56; H, 2.62; N, 12.39.

#### 8′-Bromo-4*H*-[1,4′-bi(1,5-naphthyridine)]-4,8(5*H*)-dione (**3**)

Ph_2_O (20 mL) was heated to ∼250 °C (external temperature), and ylidene **1** (200 mg, 0.61 mmol) was added in one portion. After 2 min, the reaction mixture was left to cool to <80 °C, and the solution was poured into cold *n*-hexane (−40 °C) (1000 mL). The mixture was cooled to approximately −40 °C for 2 h, and the precipitate was filtered. The collected solid was washed with *n*-pentane and recrystallized to give compound **3** as beige powder (29 mg, 13%): decomposition (DSC) onset 307.1 °C, peak max 316.0 °C (PhMe/EtOH); *R*_*f*_ = 0.71 (78:22 EtOH/DMSO); λ_max_ (DMSO, nm) 302 inf (log ε = 4.02), 320 inf (4.16), 329 (4.21), 340 (4.24); *v*_max_ (ATR, cm^–1^) 3280–2960w br (NH), 1599s (C=O), 1574s, 1547m, 1518w, 1483m, 1466m, 1385m, 1337w, 1292m, 1273m, 1244w, 1225m, 1188s, 1099w, 1086w, 1055w, 1038w, 986w, 945m, 868m, 837m, 814m, 789m, 777m; ^1^H NMR (DMSO-*d*_6_, 500 MHz) δ 11.96 (1H, br s), 8.62 (1H, d, *J* = 4.5 Hz), 8.23 (1H, d, *J* = 4.8 Hz), 7.991 (1H, d, *J* = 4.8 Hz), 7.990 (1H, d, *J* = 7.5 Hz), 7.77 (1H, d, *J* = 7.2 Hz), 6.46 (1H, d, *J* = 7.8 Hz), 6.03 (1H, d, *J* = 7.2 Hz); ^13^C{^1^H} NMR (DMSO-*d*_6_, 125 MHz) δ 171.8 (s, C=O), 171.2 (s, C=O), 152.4 (d), 151.0 (d), 149.6 (s), 144.2 (d), 140.9 (s), 140.7 (s), 137.7 (d), 135.5 (s), 132.8 (s), 132.1 (s), 128.9 (d), 121.5 (d), 113.9 (d), 110.9 (d); *m*/*z* (MALDI-TOF) 393 (^81^Br, M^+^ + Na, 29%), 391 (^79^Br, M^+^ + Na, 25%), 371 (^81^Br, M^+^, 92%), 369 (^79^Br, M^+^, 100%). Anal. Calcd for C_16_H_9_BrN_4_O_2_: C, 52.06; H, 2.46; N, 15.18. Found: C, 51.89; H, 2.53; N, 15.24.

#### 8-Bromo-1,7-naphthyridin-4(1*H*)-one (**15**)

Ph_2_O (60 mL) was heated to ∼250 °C (external temperature) and supplemented with 5-{[(2-bromopyridin-3-yl)amino]methylene}-2,2-dimethyl-1,3-dioxane-4,6-dione (**14**) (600 mg, 1.83 mmol), and the mixture heated for 0.5 min, cooled to ∼20 °C, and filtered to remove insoluble solids. The filtrate was poured into *n*-hexane (800 mL) and cooled to approximately −40 °C for 0.5 h, and the formed precipitate was collected by filtration and recrystallized to give compound **15** as beige needles (352 mg, 85%): mp (DSC) onset 135.6 °C, peak max 161.0 °C (PhMe); *R*_*f*_ = 0.26 (90:10 DCM-NH_3_/EtOH); λ_max_ (DCM, nm) 209 (log ε = 4.26), 223 inf (4.06), 255 (3.88), 281 (3.95), 292 (3.93), 315 inf (3.91), 322 inf (3.59), 332 (3.58), 346 (3.49); *v*_max_ (ATR, cm^–1^) 3280–2800w br (NH), 1684w, 1620m, 1593m, 1535m, 1508s, 1441m, 1406w, 1371w, 1319m, 1290m, 1246m, 1200m, 1184m, 1123m, 1080m, 1055m, 899m, 814m; ^1^H NMR (DMSO-*d*_6_, 300 MHz) δ 11.49 (1H, br s), 8.25 (1H, d, *J* = 5.1 Hz), 7.95–7.94 (2H, m), 6.24 (1H, d, *J* = 7.5 Hz); ^13^C{^1^H} NMR (DMSO-*d*_6_, 75 MHz) δ 175.5 (s), 141.8 (d), 141.3 (d), 134.6 (s), 133.5 (s), 131.5 (s), 118.3 (d), 111.0 (d); *m*/*z* (ESI) 227 (^81^Br, MH^+^, 99%), 225 (^79^Br, MH^+^, 100%). Anal. Calcd for C_8_H_5_BrN_2_O: C, 42.70; H, 2.24; N, 12.45. Found: C, 42.83; H, 2.30; N, 12.34.

### Synthesis of Canthin-4-one (stepwise procedure)

#### 4-Bromo-8-methoxy-1,5-naphthyridine (**5**)

8-Bromo-1,5-naphthyridin-4(1*H*)-one (**2**) (269.0 mg, 1.20 mmol) was dissolved in anhydrous DMF (4 mL), and K_2_CO_3_ (198.2 mg, 1.43 mmol) was added in one portion, followed by MeI (90 μL, 1.45 mmol). The mixture was left to stir at ∼20 °C for 14 h. PhMe (20 mL) was added, and DMF was azeotropically distilled off in vacuo. This process was repeated multiple times; additional PhMe (∼20 mL) was added and removed in vacuo until no DMF remained. The solid residue was suspended in DCM, adsorbed onto silica, and chromatographed (95:5 DCM/*t*-BuOMe) to give compound **5** (201.2 mg, 70%) as colorless needles: mp (hot stage) 133.0–134.0 °C (*c-*hexane); *R*_*f*_ = 0.33 (80:20 DCM/*t-*BuOMe); λ_max_ (DCM, nm) 280 inf (log ε = 3.94), 288 (3.99), 299 inf (3.88), 309 inf (3.65); *v*_max_ (ATR, cm^–1^) 3069w and 3024m (aryl C–H), 2945w and 2847w (alkyl C–H), 1593m, 1582m, 1551m, 1497s, 1466m, 1447m, 1393m, 1369m, 1346w, 1302s, 1261w, 1229w, 1194w, 1136m, 1098m, 1007m, 966w, 860m, 839m, 777s, 723w; ^1^H NMR (500 MHz, CDCl_3_) δ 8.92 (1H, d, *J* = 5.5 Hz), 8.69 (1H, d, *J* = 4.5 Hz), 7.99 (1H, d, *J* = 4.5 Hz), 7.05 (1H, d, *J* = 5.0 Hz), 4.15 (3H, s); ^13^C{^1^H} NMR (125 MHz, CDCl_3_) δ 162.3 (s), 152.9 (d), 149.2 (d), 142.8 (s), 137.9 (s), 136.1 (s), 128.9 (d), 104.5 (d), 58.9 (q); *m*/*z* (MS-ESI) 241 (^81^Br, MH^+^, 100%), 239 (^79^Br, MH^+^, 96%). Anal. Calcd for C_9_H_7_BrN_2_O: C, 45.22; H, 2.95; N, 11.72. Found: C, 45.31; H, 3.08; N, 11.56.

#### 4-(2-Chlorophenyl)-8-methoxy-1,5-naphthyridine (**6**)

A mixture of 4-bromo-8-methoxy-1,5-naphthyridine (**5**) (45.0 mg, 0.2 mmol), 2-chlorophenylboronic acid (46.9 mg, 0.3 mmol), K_2_CO_3_ (55.3 mg, 0.4 mmol), and PdCl_2_(dppf)·DCM (2.0 mg, 1.25 mol %) in dioxane/H_2_O (75:25) was heated to ∼88 °C (reflux) for 8 min. After completion of the reaction (as determined by TLC), the mixture was left to cool to ∼20 °C, diluted with DCM (20 mL), dried (Na_2_SO_4_), and filtered, and the volatiles were removed under reduced pressure. The remaining residue was redissolved in DCM (10 mL) and adsorbed onto silica. Chromatography (90:10 DCM/*t*-BuOMe) gave compound **6** as colorless needles (44.1 mg, 74%): mp (hot stage) 158.0–160.0 °C (*c*-hexane/PhMe); *R*_*f*_ = 0.75 (90:10 DCM/*t*-BuOMe); λ_max_ (DCM, nm) 278 inf (log ε = 4.16), 286 (4.21), 293 inf (4.18), 305 inf (4.00); *v*_max_ (ATR, cm^–1^) 3015w (aryl C–H), 2988w and 2938w (alkyl C–H), 1595m, 1580m, 1562m, 1510m, 1481m, 1462m, 1427m, 1400m, 1371m, 1350w, 1304m 1292m, 1246w, 1219m, 1155w, 1177w, 1113m, 1092m, 1059m, 1040m, 993m, 955w, 862m, 835m, 816m, 764s, 735m, 710m; ^1^H NMR (CDCl_3_, 500 MHz) δ 9.01 (1H, d, *J* = 4.5 Hz), 8.81 (1H, d, *J* = 5.5 Hz), 7.59 (1H, d, *J* = 4.0 Hz), 7.56–7.54 (1H, m), 7.43–7.39 (3H, m), 6.99 (1H, d, *J* = 5.0 Hz), 4.16 (3H, s); ^13^C{^1^H} NMR (CDCl_3_, 125 MHz) δ 162.1 (s), 152.2 (d), 149.2 (d), 146.9 (s), 143.0 (s), 137.5 (s), 136.6 (s), 133.4 (s), 131.5 (d), 129.9 (d, 2 × *C*H), 126.7 (d), 125.8 (d), 103.6 (d), 56.6 (q); *m*/*z* (ESI) 273 (^37^Cl, MH^+^, 31%), 271 (^35^Cl, MH^+^, 100%). Anal. Calcd for C_15_H_11_ClN_2_O: C, 66.55; H, 4.10; N, 10.35. Found: C, 66.43; H, 4.15; N, 10.29.

#### 8-(2-Chlorophenyl)-1,5-naphthyridin-4(1*H*)-one (**7**)

To a solution of 4-(2-chlorophenyl)-8-methoxy-1,5-naphthyridine (**6**) (128.3 mg, 0.47 mmol) in dioxane (2.5 mL) was added concentrated HCl/H_2_O (50:50) (3.6 mL), and the mixture was heated to ∼101 °C (reflux) until the starting material had been completely consumed (45 min, as determined by TLC). The mixture was left to cool to ∼20 °C, and the mixture was poured onto crushed ice, neutralized with saturated aqueous NaHCO_3_, and extracted with DCM (10 × 30 mL). The combined organic extracts were dried (Na_2_SO_4_), filtered, evaporated to dryness, and recrystallized to give compound **7** as colorless microcrystalline powder (117.0 mg, 96%): mp (DSC) onset 265.7 °C, peak max 268.0 °C (PhCl); *R*_*f*_ = 0.67 (82:18 DCM-NH_3_/EtOH); λ_max_ (EtOH, nm) 244 inf (log ε = 4.30), 247 (4.31), 332 inf (4.00), 339 (4.03); *v*_max_ (ATR, cm^–1^) 3362–2641w br (N*H*), 1622s, 1601s, 1582m, 1518s, 1477m, 1408m, 1335w, 1290m, 1202s, 1182m, 1159w, 1123w, 1107w, 1065w, 1038m, 910w, 866w, 829s, 760m, 750m; ^1^H NMR (CD_3_OD, 500 MHz) δ 8.79 (1H, d, *J* = 4.0 Hz), 7.87 (1H, d, *J* = 7.0 Hz), 7.65 (1H, dd, *J* = 8.0, 1.0 Hz), 7.59–7.55 (2H, m) 7.53 (1H, ddd, *J* = 7.5, 7.5, 1.0 Hz), 7.47 (1H, dd, *J* = 7.5, 2.0 Hz); ^13^C{^1^H} NMR (CD_3_OD, 125 MHz) δ 179.7 (s), 147.9 (d), 141.9 (d), 141.5 (s), 140.5 (s), 136.4 (s), 134.4 (s), 134.3 (s), 132.5 (d, 2 × *C*H), 131.4 (d), 129.0 (d), 128.9 (d), 112.6 (d); *m*/*z* (ESI) 281 (^37^Cl, MH^+^ + Na, 17%), 279 (^35^Cl, MH^+^ + Na, 49%), 259 (^37^Cl, MH^+^, 33%), 257 (^35^Cl, MH^+^, 100%). Anal. Calcd for C_14_H_9_ClN_2_O: C, 65.51; H, 3.53; N, 10.91. Found: C, 65.72; H, 3.28; N, 10.78.

#### 4H-Indolo[3,2,1-*de*][1,5]naphthyridin-4-one (canthin-4-one) (**8a**)

To a stirred solution of naphthyridinone **7** (77.1 mg, 0.3 mmol) in dioxane/H_2_O (75:15, 3 mL) was added K_2_CO_3_ (83.1 mg, 0.6 mmol). A premixed solution of CuI (5.7 mg, 10 mol %), DMCDA (9.45 μL, 20 mol %), and H_2_O (3 μL, 0.17 mmol) in dioxane (300 μL) was added to the reaction mixture and heated to ∼88 °C (reflux) for 12 h. The reaction mixture was left to cool to ∼20 °C, diluted with DCM, dried (Na_2_SO_4_), filtered, and evaporated to dryness. The remaining residue was redissolved in DCM (10 mL), adsorbed onto silica, and chromatographed (98:2 DCM-NH_3_/*t*-BuOMe) to give compound **8a** as pale yellow needles (50.0 mg, 76%): mp (hot stage) 263.9–265.3 °C (PhMe) [lit.^14^ mp 264 °C (*n*-heptane/DCM)]; *R*_*f*_ = 0.42 (50:50 THF/EtOAc); λ_max_ (DCM, nm) 256 inf (log ε = 4.26), 261 (4.33), 286 (4.31), 292 inf (4.25), 311 inf (3.65), 319 inf (3.60), 388 inf (3.99), 396 (4.00); *v*_max_ (ATR, cm^–1^) 3075w and 3049w (aryl C–H), 1643m, 1620s, 1557s, 1504s, 1470m, 1441m, 1327m, 1294m, 1267m, 1223m, 1194m, 1159m, 1113m, 1078w, 1028m, 976w, 941w, 856m, 826m, 808m, 746s; ^1^H NMR (CDCl_3_, 500 MHz) δ 9.04 (1H, d, *J* = 5.0 Hz), 8.26 (1H, d, *J* = 7.5 Hz), 8.15 (1H, d, *J* = 7.5 Hz), 8.11 (1H, d, *J* = 4.5 Hz), 7.75–7.70 (2H, m), 7.53–7.43 (1H, m), 6.67 (1H, d, *J* = 7.5 Hz); ^13^C{^1^H} NMR (CDCl_3_, 125 MHz) δ 179.4 (s), 147.3 (d), 139.11 (s), 139.08 (s), 135.0 (s), 133.7 (s), 131.5 (d), 131.1 (d), 125.0 (d), 124.7 (s), 124.0 (d), 118.7 (d), 117.6 (d), 111.0 (d); *m*/*z* (MALDI-TOF) 243 (M^+^ + Na, 100%), 221 (M^+^, 22%). Anal. Calcd for C_14_H_8_N_2_O: C, 76.35; H, 3.66; N, 12.72. Found: C, 76.42; H, 3.54; N, 12.81.

### Synthesis of Canthin-4-one **8a** (one-pot procedure)

A mixture of 4-bromo-8-methoxy-1,5-naphthyridine (**5**) (71.7 mg, 0.3 mmol), 2-chlorophenylboronic acid (70.4 mg, 0.45 mmol), K_2_CO_3_ (82.9 mg, 0.6 mmol), and PdCl_2_(dppf)·DCM (3.1 mg, 1.25 mol %) in dioxane/H_2_O (75:25, 6 mL) was heated to ∼88 °C (reflux) for 12 min until starting material **6** had been completely consumed (as determined by TLC). The reaction mixture was left to cool to ∼20 °C; concentrated HCl (300 μL) was added, and the mixture was heated back to ∼88 °C (reflux). After 2 h, the reaction mixture was left to cool to ∼20 °C and K_2_CO_3_ (414.6 mg, 3.00 mmol) was added carefully. The reaction mixture was stirred until the effervescence ceased and the K_2_CO_3_ was completely dissolved. A premixed solution of CuI (5.7 mg, 0.03 mmol, 10 mol %), DMCDA (9.45 μL, 0.06 mmol, 20 mol %), and H_2_O (3 μL, 0.17 mmol) in dioxane (300 μL) was added, and the reaction mixture was heated to ∼88 °C (reflux) for 12 h. The reaction mixture was left to cool to ∼20 °C, diluted (DCM, 15 mL), dried (Na_2_SO_4_), filtered, and evaporated to dryness. The residue was redissolved in DCM (10 mL) and adsorbed onto silica. Chromatography gave 4*H*-indolo[3,2,1-*de*][1,5]naphthyridin-4-one (canthin-4-one) (**8a**) as pale yellow needles (52.9 mg, 80%) identical to those described above.

### Synthesis of 4*H*-Indolo[3,2,1-*ij*][1,6]naphthyridin-4-one (**9a**)

#### 8-Phenyl-1,6-naphthyridin-4(1*H*)-one (**12**)

A mixture of 8-bromo-1,6-naphthyridin-4(1*H*)-one (**11**) (112.5 mg, 0.5 mmol), K_2_CO_3_ (138.5 mg, 1.0 mmol), phenylboronic acid (94.0 mg, 0.75 mmol) and Pd(dppf)Cl_2_·DCM (20.0 mg, 5 mol %) in dioxane/H_2_O (75:25, 5 mL) was heated to ∼88 °C (reflux) for 12 min until the 8-bromo-1,6-naphthyridin-4(1*H*)-one (**11**) had been consumed (as determined by TLC). The reaction mixture was then allowed to cool to ∼20 °C, diluted (DCM, 15 mL), dried (Na_2_SO_4_), filtered, and adsorbed onto silica gel. Chromatography (THF) gave compound **12** as colorless needles (83.9 mg, 75%): mp (DSC) onset 253.6 °C, peak max 254.5 °C (PhCl); *R*_*f*_ = 0.30 (60:40 DCM/THF; λ_max_ (DCM, nm) 280 inf (log ε = 3.82), 289 (3.88), 319 (4.16); *v*_max_ (ATR, cm^–1^) 3250–2650w br (NH), 1641s, 1589s, 1568m, 1493s, 1451w, 1435w, 1310m, 1275w, 1248w, 1209m, 1138m, 1088w, 1043m, 1024m, 916w, 853m, 843m, 829m, 808m, 791m, 770s, 704m; ^1^H NMR (DMSO-*d*_6_, 500 MHz) δ 9.22 (1H, s), 8.48 (1H, s), 7.78 (1H, d, *J* = 7.5 hz), 7.61–7.53 (5H, m), 6.21 (1H, d, *J* = 7.5 Hz); ^13^C{^1^H} NMR (DMSO-*d*_6_, 125 MHz) δ 176.9 (s), 149.6 (d), 148.4 (d), 141.9 (s), 141.4 (d), 133.3 (s), 129.6 (d), 129.2 (d), 128.7 (d), 125.5 (s), 120.3 (s), 112 (d); *m*/*z* (ESI) 261 (M^+^ + K, 16%), 245 (M^+^ + Na, 22%), 223 (MH^+^, 100%). Anal. Calcd for C_14_H_10_N_2_O: C, 75.66; H, 4.54; N, 12.60. Found: C, 75.80; H, 4.32; N, 12.73.

#### 4*H*-Indolo[3,2,1-*ij*][1,6]naphthyridin-4-one (**9a**) (one-pot procedure, MW conditions)

A glass vessel tube used as a microwave reactor was charged with naphthyridinone **11** (45.0 mg, 0.2 mmol), 2-chlorophenylboronic acid (46.9 mg, 0.3 mmol), K_2_CO_3_ (55.3 mg, 0.4 mmol), and PdCl_2_(dppf)·DCM (8.2 mg, 5 mol %) in dioxane/H_2_O (75:25, 2 mL). The tube was capped, and the reaction performed in the microwave reactor at ∼120 °C (*P* = 150 W; pressure = 50 psi) for 45 min. The mixture was left to cool to ∼20 °C, and K_2_CO_3_ (55.3 mg, 0.4 mmol) and a premixed solution of CuI (3.8 mg, 10 mol %) and DMCDA (6.3 μL, 20 mol %) in dioxane/H_2_O (75:25, 300 μL) were added. The tube was capped and placed back in the microwave reactor at 120 °C (*P* = 150 W; pressure = 50 psi) for 6 h. The mixture was left to cool to ∼20 °C, diluted (DCM, 20 mL), dried (Na_2_SO_4_), filtered, and evaporated to dryness. The residue was dissolved in DCM (20 mL) and adsorbed onto silica. Chromatography (50:50 EtOAc/THF) gave compound **9a** as beige needles (30.4 mg, 69%): mp (hot stage) 237.0–238.0 °C (PhMe); *R*_*f*_ = 0.50 (50:50 EtOAc/THF); λ_max_ (DCM, nm) 233 (log ε = 3.29), 263 (4.36), 280 inf (4.08), 285 inf (4.12), 287 (4.14), 292 inf (4,09), 318 (3.63), 359 (4.01), 366 inf (4.00), 369 inf (4.00); *v*_max_ (ATR, cm^–1^) 3100w, 3067w, and 3032w (aryl C–H), 1649s (C=O), 1618m, 1597m, 1562m, 1503m, 1472m, 1454m, 1425w, 1379w, 1337m, 1285m, 1229m, 1200m, 1167m, 1159m, 1125w, 1088m, 1024m, 1016m, 955w, 903w, 872m, 833m, 800m, 789m, 745s; ^1^H NMR (CDCl_3_, 500 MHz) δ 9.46 (1H, s), 9.40 (1H, s), 8.30 (1H, d, *J* = 8.0 Hz), 8.16 (1H, d, *J* = 8.0 Hz), 7.72 (1H, d, *J* = 8.5 Hz), 7.65 (1H, ddd, *J* = 7.8, 7.8, 1.0 Hz), 7.51 (1H, ddd, *J* = 7.8, 7.8, 1.0 Hz), 6.60 (1H, d, *J* = 8.0 Hz); ^13^C{^1^H} NMR (CDCl_3_, 125 MHz) δ 179.2 (s), 146.1 (d), 144.3 (d), 142.2 (s), 138.3 (s), 132.6 (d), 129.1 (d), 125.2 (d), 124.4 (s), 123.0 (d), 121.7 (s), 118.6 (s), 118.1 (d), 110.7 (d); *m*/*z* (MALDI-TOF) 243 (M^+^ + Na, 100%), 221 (MH^+^, 56%), 220 (M^+^, 34%). Anal. Calcd for C_14_H_8_N_2_O: C, 76.35; H, 3.66; N, 12.72. Found: C, 76.49; H, 3.58; N, 12.80.

#### 4*H*-Indolo[3,2,1-*ij*][1,6]naphthyridin-4-one (**9a**) (one-pot procedure, conventional heating)

A mixture of naphthyridinone **11** (225.1 mg, 1.0 mmol), 2-chlorophenylboronic acid (235.0 mg, 1.5 mmol), K_2_CO_3_ (277.0 mg, 2.0 mmol), and PdCl_2_(dppf)·DCM (210 mg, 25 mol %) in dioxane/H_2_O (75:25, 10 mL) was heated to ∼88 °C (reflux) for 10 min until the starting naphthyridinone **11** had been completely consumed (as determined by TLC). The reaction mixture was left to cool to room temperature (rt); K_2_CO_3_ (277.0 mg, 2.0 mmol) was added, followed by a premixed solution of CuI (19 mg, 0.1 mmol, 10 mol %) and DMCDA (31.5 μL, 0.2 mmol, 20 mol %) in dioxane/H_2_O (75:25, 2 mL), and the mixture was heated to ∼88 °C (reflux) for 12 h. The reaction mixture was left cool to rt, diluted (DCM, 50 mL), dried (Na_2_SO_4_), filtered, and evaporated to dryness. The residue was dissolved in DCM and adsorbed onto silica. Chromatography (50:50 EtOAc/THF) gave compound **9a** as beige needles (132.0 mg, 60%) identical to those described above.

### Synthesis of 4*H*-Indolo[3,2,1-*ij*][1,7]naphthyridin-4-one (**10a**)

#### 8-Phenyl-1,7-naphthyridin-4(1*H*)-one (**16**)

A mixture of 8-bromo-1,7-naphthyridin-4(1*H*)-one (**15**) (45.0 mg, 0.2 mmol), K_2_CO_3_ (55.4 mg, 0.4 mmol), phenylboronic acid (36.6 mg, 0.3 mmol), and PdCl_2_(dppf)·DCM (8.2 mg, 5 mol %) in dioxane/H_2_O (75:25, 1 mL) was heated to ∼88 °C (reflux) for 10 min. After the starting material **15** had been completely consumed (as determined by TLC), the reaction mixture was diluted (DCM, 15 mL), dried (Na_2_SO_4_), filtered, and evaporated in vacuo to dryness. The residue was absorbed onto silica and chromatographed (96:4 DCM-NH_3_/EtOH) to give compound **16** (40.4 mg, 91%) as beige prisms: mp (DSC) onset 206.9 °C, peak max 208.3 °C (*n*-pentane/EtOAc); *R*_*f*_ = 0.51 (50:50 EtOAc/THF); λ_max_ (DCM, nm) 282 (log ε = 3.77), 292 (3.83), 339 (4.14), 344 inf (4.14); *v*_max_ (ATR, cm^–1^) 3200–2800w br (N*H*), 1626m, 1607m, 1582m, 1531m, 1514s, 1493m, 1450m, 1441m, 1402w, 1294m, 1267m, 1227w, 1190m, 1072w, 1030w, 1022w, 970w, 908w, 856w, 814m, 768m, 719m; ^1^H NMR (CDCl_3_, 500 MHz) δ 9.18 (1H, br s), 8.57 (1H, d, *J* = 5.5 Hz), 8.06 (1H, d, *J* = 5.0 Hz), 7.68–7.65 (3H, m), 7.55 (2H, dd, *J* = 7.5, 7.5 Hz), 7.49 (1H, t, *J* = 7.4 Hz), 6.29 (1H, d, *J* = 7.5 Hz); ^13^C{^1^H} NMR (CDCl_3_, 125 MHz) δ 177.8 (s), 150.9 (s), 142.9 (d), 138.8 (d), 136.1 (s), 132.9 (s), 131.2 (s), 130.0 (d), 129.7 (d), 129.0 (d), 117.8 (d), 111.5 (d); *m*/*z* (ESI) 261 (M^+^ + K, 38%), 245 (M^+^ + Na, 50%), 223 (M^+^, 100%). Anal. Calcd for C_14_H_10_N_2_O: C, 75.66; H, 4.54; N, 12.60. Found: C, 75.70; H, 4.48; N, 12.53.

#### 4*H*-Indolo[3,2,1-*ij*][1,7]naphthyridin-4-one (**10a**) (one-pot procedure)

A mixture of naphthyridinone **15** (225.1 mg, 1.0 mmol), 2-chlorophenylboronic acid (235.0 mg, 1.5 mmol), K_2_CO_3_ (277.0 mg, 2.0 mmol), and PdCl_2_(dppf)·DCM (5 mol %) in dioxane/H_2_O (75:25, 10 mL) was heated to ∼88 °C (reflux) for 15 min until the starting naphthyridinone **15** had been completely consumed (as determined by TLC). The reaction mixture was left to cool to rt; K_2_CO_3_ (277.0 mg, 2.0 mmol) was added, followed by a premixed solution of CuI (19 mg, 0.1 mmol, 10 mol %), DMCDA (31.5 μL, 0.2 mmol, 20 mol %), and H_2_O (18 μL, 1.0 mmol), in dioxane (1.0 mL), and the mixture was heated to ∼88 °C (reflux) for 12 h. The reaction mixture was left cool to rt, diluted (DCM, 50 mL), dried (Na_2_SO_4_), filtered, and evaporated to dryness. The residue was dissolved in DCM and adsorbed onto silica. Chromatography (70:30 EtOAc/THF) gave compound **10a** as beige needles (170.0 mg, 77%): mp (hot stage) 237.0–238.0 °C (PhMe); *R*_*f*_ = 0.59 (50:50 EtOAc/THF); λ_max_ (DCM, nm) 243 inf (log ε = 4.21), 262 (4.09), 281 inf (4.20), 288 inf (4.31), 292 inf (4.34), 295 (4.36), 332 inf (3.85), 341 (3.88), 389 (4.24); *v*_max_ (ATR, cm^–1^) 3050w (aryl C–H), 1659m, 1643m, 1614s (C=O), 1587m, 1572w, 1541s, 1499s, 1460m, 1433m, 1393w, 1354m, 1317s, 1290m, 1265m, 1202m, 1186m, 1152m, 1111w, 1098m, 1070w, 1030m, 947w, 868w, 824m, 812m, 797m, 712m; ^1^H NMR (CDCl_3_, 500 MHz) δ 8.90 (1H, d, *J* = 5.5 Hz), 8.34 (1H, d, *J* = 8.0 Hz), 8.27 (1H, d, *J* = 8.0 Hz), 7.99 (1H, d, *J* = 5.5 Hz), 7.70 (1H, d, *J* = 8.0 Hz), 6.67 (1H, ddd, *J* = 7.5, 7.5, 1.0 Hz), 7.72 (1H, ddd, *J* = 7.5, 7.5, 1.0 Hz), 6.53 (1H, d, *J* = 7.5 Hz); ^13^C{^1^H} NMR (CDCl_3_, 125 MHz) δ 179.5 (s), 148.4 (s), 146.2 (d), 139.6 (s), 132.6 (d), 131.5 (s), 130.4 (d), 127.1 (s), 125.7 (s), 125.3 (d), 122.8 (d), 116.2 (d), 115.6 (d), 110.7 (d); *m*/*z* (MALDI-TOF) 244 (MH^+^ + Na, 66%), 243 (M^+^ + Na, 95%), 221 (MH^+^, 100%), 220 (M^+^, 81%). Anal. Calcd for C_14_H_8_N_2_O: C, 76.35; H, 3.66; N, 12.72. Found: C, 76.23; H, 3.72; N, 12.85.

### Analogues of Canthin-4-ones **8** and Isocanthin-4-ones **9** and **10**

#### Canthin-4-ones **8** (general procedure)

A mixture of 4-bromo-8-methoxy-1,5-naphthyridine (**5**) (71.7 mg, 0.3 mmol), K_2_CO_3_ (82.8 mg, 0.6 mmol), PdCl_2_(dppf)·DCM (3.0 mg, 1.25 mol %), and the appropriate 2-chlorophenylboronic acid (1.5 or 3 equiv) in dioxane/H_2_O (75:25, 6 mL) was heated to ∼88 °C (reflux) until the starting material had been completely consumed (as determined by TLC). The mixture was left to cool to rt; concentrated HCl (300 μL) was added in one portion, and the mixture heated again to reflux for 2 h. Then the mixture was left to cool to rt, and K_2_CO_3_ (414.6 mg, 3.0 mmol) was added portionwise. The mixture was left to stir for a few minutes until the effervescence ceased and K_2_CO_3_ dissolved; the color changed to deep brown. A premixed mixture of CuI (5.7 mg, 10 mol %) and DMCDA (9.45 μL, 20 mol %) in dioxane/H_2_O (75:25, 1.0 mL) was added, and the reaction mixture heated to ∼88 °C (reflux). After 12 h, the mixture was left to cool to ∼20 °C, diluted (DCM, 50 mL), dried (Na_2_SO_4_), filtered, and evaporated to dryness. The remaining residue was dissolved in DCM, adsorbed onto silica, and chromatographed to give 4*H*-indolo[3,2,1-*de*][1,5]naphthyridin-4-one **8**.

##### 9-Methyl-4*H*-indolo[3,2,1-*de*][1,5]naphthyridin-4-one (**8b**)

2-Chloro-4-methyl-phenylboronic acid (76.8 mg, 0.45 mmol) gave compound **8b** (50:50 EtOAc/THF) as beige needles (52.4 mg, 74%): mp (hot stage) 264–266 °C; mp (DSC) onset 269.1 °C, peak max 271.1 °C (EtOH); *R*_*f*_ = 0.40 (60:40 EtOAc/THF); λ_max_ (DCM, nm) 257 inf (log ε = 4.46), 262 (4.51), 287 inf (4.51), 293 (4.57), 321 (4.04), 329 inf (4.04), 389 (4.25); *v*_max_ (ATR, cm^–1^) 3045w (aryl C–H), 2924w (alkyl C–H), 1620s (C=O), 1557m, 1508s, 1472m, 1431m, 1375w, 1325w, 1290m, 1223m, 1204m, 1161m, 1125m, 1084w, 1024w, 866m, 827m, 812m, 785m, 746w, 731w; ^1^H NMR (CDCl_3_, 500 MHz) δ 9.01 (1H, d, *J* = 4.5 Hz), 8.22 (1H, d, *J* = 8.0 Hz), 8.05 (1H, d, *J* = 5.0 Hz), 8.0 (1H, d, *J* = 7.5 Hz), 7.51 (1H, s), 7.31 (1H, d, *J* = 8.0 Hz), 2.61 (3H, s); ^13^C{^1^H} NMR (CDCl_3_, 125 MHz) δ 179.5 (s), 147.3 (d), 142.3 (s), 139.6 (s), 139.0 (s), 135.3 (s), 133.9 (s), 131.4 (d), 126.2 (d), 123.6 (d), 122.2 (s), 118.3 (d), 117.5 (d), 111.3 (d), 22.5 (q); *m*/*z* (ESI) 257 (M^+^ + Na, 20%), 235 (MH^+^, 100%). Anal. Calcd for C_15_H_10_N_2_O: C, 76.91; H, 4.30; N, 11.96. Found: C, 77.02; H, 4.26; N, 11.74.

##### 9-Methoxy-4*H*-indolo[3,2,1-*de*][1,5]naphthyridin-4-one (**8c**)

2-Chloro-4-methoxyphenylboronic acid (83.9 mg, 0.45 mmol) gave compound **8c** (chromatography eluent, 50:50 EtOAc/THF) as pale yellow needles (26.5 mg, 35%): mp (DSC) onset 298.2 °C, peak max 301.0 °C, decomposition peak max 302.9 °C (EtOH); *R*_*f*_ = 0.18 (THF); λ_max_ (DCM, nm) 259 inf (log ε = 4.26), 264 (4.29), 284 inf (4.28), 293 (4.45), 299 (4.48), 345 (4.10), 373 (4.16); *v*_max_ (ATR, cm^–1^) 3032w (aryl C–H), 1641m, 1607s (C=O), 1557m, 1510s, 1477m, 1470m, 1454m, 1441m, 1431m, 1329m, 1310m, 1275w, 1227s, 1192m, 1175m, 1163m, 1115m, 1069w, 976w, 868m, 853m, 826m, 802m, 793m; ^1^H NMR (CDCl_3_, 500 MHz) δ 8.98 (1H, d, *J* = 4.5 Hz), 8.20 (1H, d, *J* = 8.0 Hz), 8.00 (1H, d, *J* = 8.5 Hz), 7.99 (1H, d, *J* = 4.5 Hz), 7.18 (1H, d, *J* = 2.0 Hz), 7.04 (1H, dd, *J* = 8.5, 2.0 Hz), 6.65 (1H, d, *J* = 8.0 Hz), 3.99 (3H, s); ^13^C{^1^H} NMR (DMSO-*d*_6_, 125 MHz) δ 178.2 (s), 162.3 (s), 146.8 (d), 140.8 (s), 137.9 (s), 135.0 (s), 133.6 (d), 133.3 (s), 124.9 (d), 116.54 (s), 116.47 (d), 112.4 (d), 97.3 (d), 56.1 (q); *m*/*z* (ESI) 273 (M^+^ + Na, 20%), 251 (MH^+^, 100%). Anal. Calcd for C_15_H_10_N_2_O_2_: C, 71.99; H, 4.03; N, 11.19. Found: C, 72.03; H, 4.16; N, 11.12.

##### 9-(Trifluoromethyl)-4*H*-indolo[3,2,1-*de*][1,5]naphthyridin-4-one (**8d**)

2-Chloro-4-(trifluoromethyl)phenylboronic acid (201.9 mg, 0.9 mmol) gave compound **8d** (chromatography eluent, 80:20 DCM/THF) as beige needles (57.9 mg, 67%): mp (DSC) onset 302.8 °C, peak max 303.9 °C (PhCl); *R*_*f*_ = 0.44 (30:70 DCM/EtOAc); λ_max_ (DCM, nm) 255 inf (log ε = 4.28), 258 (4.30), 282 (4.30), 288 (4.30), 297 (4.21), 316 (3.52), 368 inf (3.81), 385 inf (4.06), 398 (4.18); *v*_max_ (ATR, cm^–1^) 3063w and 3034w (aryl C–H), 1649m, 1627m, 1557m, 1510m, 1474m, 1450m, 1435m, 1336s, 1302m, 1225m, 1177s, 1109s, 1061s, 1024m, 999w, 982w, 961w, 908w, 851w, 831m, 818s, 795m, 758w, 741m, 725m; ^1^H NMR (CDCl_3_, 500 MHz) δ 9.13 (1H, d, *J* = 5.0 Hz), 8.34–8.31 (2H, m), 8.22 (1H, d, *J* = 4.5 Hz), 8.018 (1H, s), 7.78 (1H, d, *J* = 8.0 Hz), 6.73 (1H, d, *J* = 7.5 Hz); ^13^C{^1^H} NMR (CDCl_3_, 125 MHz) δ 179.1 (s), 147.5 (d), 139.3 (s), 138.7 (s), 135.6 (s), 133.0 (q, ^2^*J*_CF_ = 32.5 Hz), 132.4 (s), 131.3 (d), 127.5 (s), 124.5 (d), 123.8 (q, ^1^*J*_CF_ = 271.4 Hz), 121.7 (q, ^3^*J*_CF_ = 3.5 Hz), 119.4 (d), 118.3 (d), 108.5 (q, ^3^*J*_CF_ = 3.8 Hz); *m*/*z* (ESI) 311 (M^+^ + Na, 48%), 289 (MH^+^, 100%). Anal. Calcd for C_15_H_7_F_3_N_2_O: C, 62.51; H, 2.45; N, 9.72. Found: C, 62.63; H, 2.57; N, 9.80.

##### 9-Chloro-4*H*-indolo[3,2,1-*de*][1,5]naphthyridin-4-one (**8e**)

2,4-Dichlorophenylboronic acid (85.9 mg, 0.45 mmol) gave compound **8e** (chromatography eluent, 50:50 THF/EtOAc) as yellow plates (26.7 mg, 35%): mp (hot stage) 272.0–274.0 °C (EtOAc); *R*_*f*_ = 0.44 (30:70 DCM/EtOAc); λ_max_ (DCM, nm) 257 inf (log ε = 4.18), 261 (4.23), 288 inf (4.30), 292 (4.33), 318 inf (3.75), 328 (3.97), 364 inf (3.78), 382 inf (3.97), 391 (4.01); *v*_max_ (ATR, cm^–1^) 3084w and 3040w (aryl C–H), 1649m, 1643m, 1620s, 1612s, 1574w, 1555m, 1503s, 1449m, 1431m, 1321m, 1298m, 1279m, 1223m, 1184m, 1159w, 1121m, 1099m, 1076m, 1067m, 1022m, 961w, 935w, 924w, 878m, 827m, 802m, 791m, 745w; ^1^H NMR (CDCl_3_, 500 MHz) δ 9.06 (1H, d, *J* = 4.5 Hz), 8.21 (1H, d, *J* = 8.0 Hz), 8.11 (1H, d, *J* = 4.5 Hz), 8.09 (1H, d, *J* = 8.5 Hz), 7.74 (1H, d, *J* = 1.5 Hz), 7.49 (1H, dd, *J* = 8.5, 1.5 Hz), 6.68 (1H, d, *J* = 8.0 Hz); ^13^C{^1^H} NMR (CDCl_3_, 125 MHz) δ 179.2 (s), 147.5 (d), 139.7 (s), 139.2 (s), 137.2 (s), 135.4 (s), 132.9 (s), 131.2 (d), 125.4 (d), 124.7 (d), 123.2 (s), 118.7 (d), 118.1 (d), 111.7 (d); *m*/*z* (ESI) 257 (^37^Cl, MH^+^, 33%), 255 (^35^Cl, MH^+^, 100%). Anal. Calcd for C_14_H_7_ClN_2_O: C, 66.03; H, 2.77; N, 11.00. Found: C, 66.07; H, 2.69; N, 10.85.

##### 9-Fluoro-4*H*-indolo[3,2,1-*de*][1,5]naphthyridin-4-one (**8f**)

2-Chloro-4-fluorophenylboromic acid (78.5 mg, 0.45 mmol) gave compound **8f** (chromatography eluent, 75:25 DCM/THF) (63.6 mg, 89%) as yellow needles: mp (DSC) onset 309.0 °C, peak max 310.7 °C (PhCl); *R*_*f*_ = 0.39 (50:50 EtOAc/THF); λ_max_ (DCM, nm) 288 inf (log ε = 3.81), 294 (3.89), 303 (3.89), 321 (3.25), 394 inf (3.60), 402 (3.63); *v*_max_ (ATR, cm^–1^) 3034w (aryl C–H), 1641m, 1614m, 1580m, 1555m, 1503s, 1468m, 1443m, 1422m, 1298m, 1321m, 1269m, 1223m, 1159m, 1188m, 1130w, 1101w, 1078m, 1024w, 943m, 878m, 823s, 795m, 758m; ^1^H NMR (CDCl_3_, 500 MHz) δ 8.98 (2H, d, *J* = 5.0 Hz), 8.92 (1H, d, *J* = 8.0 Hz), 8.46 (1H, d, *J* = 5.0 Hz), 8.43 (1H, dd, *J* = 8.5, 5.5 Hz), 8.17 (1H, dd, *J* = 9.5, 2.0 Hz), 7.40 (1H, ddd, *J* = 8.0, 8.0, 2.0 Hz), 6.54 (1H, d, *J* = 8.0 Hz); ^13^C{^1^H} NMR (CDCl_3_, 125 MHz) δ 179.4 (s), 164.8 (d, ^1^*J*_CF_ = 251.9 Hz), 147.6 (s), 140.3 (d, ^3^*J*_CF_ = 12.1 Hz), 139.0 (s), 135.9 (s), 133.2 (s), 125.4 (d, ^3^*J*_CF_ = 10.2 Hz), 121.0 (d, ^4^*J*_CF_ = 2.2 Hz), 118.6 (d), 118.1 (d), 113.0 (d, ^2^*J*_CF_ = 24.1 Hz), 99.3 (d, ^2^*J*_CF_ = 27.9 Hz); *m*/*z* (ESI) 261 (M^+^ + Na, 74%), 239 (MH^+^, 100%). Anal. Calcd for C_14_H_7_FN_2_O: C, 70.59; H, 2.96; N, 11.76. Found: C, 70.47; H, 2.90; N, 11.69.

##### 10-Methoxy-4*H*-indolo[3,2,1-*de*][1,5]naphthyridin-4-one (**8g**)

2-Chloro-5-methoxyphenylboromic acid (83.9 mg, 0.45 mmol) gave compound **8g** (chromatography eluent, THF) as yellow needles (52.1 mg, 69%): mp (DSC) onset 257.0 °C, peak max 258.3 °C (EtOH); *R*_*f*_ = 0.58 (THF); λ_max_ (DCM, nm) 243 (log ε = 3.98), 252 (4.09), 268 (4.30), 275 inf (4.19), 307 inf (4.28), 311 (4.30), 327 inf (3.85), 410 (3.90), 422 inf (3.86); *v*_max_ (ATR, cm^–1^) 3076w (aryl C–H), 1639m, 1632m, 1612s, 1593m, 1553m, 1504s, 1485s, 1429m, 1323m, 1283m, 1223s, 1190m, 1119m, 1036m, 947w, 822m; ^1^H NMR (CDCl_3_, 500 MHz) δ 9.04 (1H, d, *J* = 4.8 Hz), 8.20 (1H, d, *J* = 7.8 Hz), 8.09 (1H, d, *J* = 4.8 Hz), 7.63–7.61 (2H, m), 7.29 (1H, dd, *J* = 8.8, 2.6 Hz), 6.65 (1H, d, *J* = 7.7 Hz), 3.97 (3H, s); ^13^C{^1^H} NMR (CDCl_3_, 125 MHz) δ 179.2 (s), 157.6 (s), 147.1 (d), 139.3 (s), 135.4 (s), 133.8 (s), 133.5 (s), 131.6 (d), 125.8 (s), 118.7 (d), 118.6 (d), 117.2 (d), 111.7 (d), 107.5 (d), 56.2 (q); *m*/*z* (ESI) 273 (M^+^ + Na, 35%), 251 (MH^+^, 100%). Anal. Calcd for C_15_H_10_N_2_O_2_: C, 71.99; H, 4.03; N, 11.19. Found: C, 72.03; H, 3.97; N, 11.05.

##### 10-(Trifluoromethoxy)-4*H*-indolo[3,2,1-*de*][1,5]naphthyridin-4-one (**8h**)

2-Chloro-5-(trifluoromethoxy)phenylboronic acid (216.3 mg, 0.9 mmol) gave compound **8h** (chromatography eluent, EtOAc) as yellow needles (60.9 mg, 67%): mp (DSC) onset 267.2 °C, peak max 267.8 °C (EtOH); *R*_*f*_ = 0.70 (THF); λ_max_ (DCM, nm) 255 inf (log ε = 4.43), 259 (4.47), 285 inf (4.46), 289 (4.49), 295 inf (4.43), 318 inf (3.79), 388 inf (4.22), 397 (4.26); *v*_max_ (ATR, cm^–1^) 3103w and 3038w (aryl C–H), 1643m, 1620m, 1601m, 1553m, 1504m, 1476m, 1449w, 1429m, 1329w, 1265s, 1221m, 1194m, 1153m, 1094w, 1074w, 1051w, 1026w, 962w, 935w, 914w, 875m, 858m, 835m, 793w, 760w, 731w; ^1^H NMR (CDCl_3_, 500 MHz) δ 9.09 (1H, d, *J* = 4.5 Hz), 8.27 (1H, d, *J* = 8.0 Hz), 8.15 (1H, d, *J* = 5.0 Hz), 8.03 (1H, d, *J* = 1.0 Hz), 7.77 (1H, d, *J* = 9.0 Hz), 7.60 (1H, dd, *J* = 8.8, 2.2 Hz), 6.69 (1H, d, *J* = 8.0 Hz); ^13^C{^1^H} NMR (CDCl_3_, 125 MHz) δ 179.1 (s), 147.4 (d), 146.4 (s), 139.3 (s), 137.3 (s), 135.6 (s), 132.7 (s), 131.5 (d), 125.8 (s), 124.4 (d), 120.7 (q, ^1^*J*_CF_ = 258.3 Hz), 119.1 (d), 118.1 (d), 117.0 (d), 112.0 (d); *m*/*z* (ESI) 327 (M^+^ + Na, 25%), 305 (MH^+^, 100%). Anal. Calcd for C_15_H_7_F_3_N_2_O_2_: C, 59.22; H, 2.32; N, 9.21. Found: C, 59.30; H, 2.19; N, 9.34.

##### 10-(Trifluoromethyl)-4*H*-indolo[3,2,1-*de*][1,5]naphthyridin-4-one (**8i**)

2-Chloro-5-(trifluoromethyl)phenylboronic acid (201.9 mg, 0.9 mmol) gave compound **8i** (chromatography eluent, 50:50 EtOAc/THF) as beige needles (86.7 mg, 63%): mp (DSC) onset 311.4 °C, peak max 312.4 °C (EtOH); *R*_*f*_ = 0.44 (50:50 EtOAc/THF); λ_max_ (DCM, nm) 253 inf (log ε = 4.16), 258 (4.20), 282 (4.28), 285 (4.28), 293 inf (4.13), 316 (3.42), 380 inf (4.00), 390 (4.10); *v*_max_ (ATR, cm^–1^) 3059w and 3017w (aryl C–H), 1649m, 1626s, 1560m, 1504m, 147m, 1427m, 1335s, 1298m, 1275m, 1221m, 1198m, 1148m, 1121m, 1078m, 1061m, 1026m, 945m, 899m, 883m, 826m, 723m; ^1^H NMR (CDCl_3_, 500 MHz) δ 9.13 (1H, d, *J* = 4.8 Hz), 8.46 (1H, s), 8.32 (1H, d, *J* = 7.8 Hz), 8.21 (1H, d, *J* = 4.8 Hz), 8.00 (1H, d, *J* = 8.5 Hz), 7.87 (1H, *J* = 8.5 Hz), 6.73 (1H, d, *J* = 7.8 Hz); ^13^C{^1^H} NMR (CDCl_3_, 125 MHz) δ 179.2 (s), 147.6 (d), 140.7 (s), 139.2 (s), 135.6 (s), 132.7 (s), 131.4 (d), 128.1 (q, ^3^*J*_CF_ = 3.6 Hz), 127.4 (q, ^2^*J*_CF_ = 33.1 Hz), 124.8 (s), 124.0 (q, ^1^*J*_CF_ = 272.2 Hz), 121.5 (q, ^3^*J*_CF_ = 3.8 Hz), 119.1 (d), 118.5 (d), 111.5 (d); *m*/*z* (ESI) 327 (M^+^ + K, 8%), 311 (M^+^ + Na, 67%), 289 (MH^+^, 100%). Anal. Calcd for C_15_H_7_F_3_N_2_O: C, 59.22; H, 2.32; N, 9.21. Found: C, 59.30; H, 2.51; N, 9.19.

##### 10-Chloro-4*H*-indolo[3,2,1-*de*][1,5]naphthyridin-4-one (**8j**)^3^

2,5-Dichlorophenylboronic acid (171.7 mg, 0.9 mmol) gave compound **8j** (chromatography eluent, 50:50 EtOAc/THF) as pale yellow needles (53.1 mg, 61%): mp (DSC) onset 321.8 °C, peak max 323.0 (EtOH); *R*_*f*_ = 0.39 (50:50 EtOAc/THF); λ_max_ (DCM, nm) 258 inf (log ε = 4.15), 262 (4.18), 288 inf (4.14), 294 (4.23), 302 (4.22), 304 inf (4.22), 322 (3.58), 403 (3.96); *v*_max_ (ATR, cm^–1^) 3088w and 3028w (aryl C–H), 1649s, 1632m, 1620s, 1614s, 1580m, 1553m, 1505s, 1468m, 1443m, 1422m, 1321m, 1298m, 1271m, 1225m, 1190m, 1159m, 1128m, 1105w, 1080m, 1026m, 941m, 880m, 826s; ^1^H NMR (CDCl_3_, 500 MHz) δ 9.09 (1H, d, *J* = 4.5 Hz), 8.24 (1H, d, *J* = 7.8 Hz), 8.16 (1H, s), 8.13 (1H, d, *J* = 4.6 Hz), 7.71–7.67 (2H, m), 6.70 (1H, d, *J* = 7.7 Hz); ^13^C{^1^H} NMR (CDCl_3_, 125 MHz) δ 179.1 (s), 147.4 (d), 139.2 (s), 137.4 (s), 135.3 (s), 132.7 (s), 131.4 (d), 131.1 (d), 130.8 (s), 126.0 (s), 124.0 (d), 119.0 (d), 117.9 (d), 112.0 (d); *m*/*z* (ESI) 279 (^37^Cl, M^+^ + Na, 37%), 277 (^35^Cl, M^+^ + Na, 100%), 257 (^37^Cl, MH^+^, 10%), 255 (^35^Cl, MH^+^, 28%). Anal. Calcd for C_14_H_7_ClN_2_O: C, 66.03; H, 2.77; N, 11.00. Found: C, 66.11; H, 2.65; N, 10.98.

##### 10-Fluoro-4*H*-indolo[3,2,1-*de*][1,5]naphthyridin-4-one (**8k**)

2-Chloro-5-fluorophenylboronic acid (78.5 mg, 0.45 mmol) gave compound **8k** (chromatography eluent, 50:50 EtOAc/THF) as yellow needles (45.1 mg, 63%): mp (DSC) onset 298.1 °C, peak max 301.6 °C (PhCl); *R*_*f*_ = 0.63 (THF); λ_max_ (DCM, nm) 249 inf (log ε = 4.16), 256 inf (4.28), 261 (4.33), 287 inf (4.26), 300 (4.30), 321 (3.72), 393 inf (4.05), 403 (4.06); *v*_max_ (ATR, cm^–1^) 3049w (aryl C–H), 1643m, 1618m, 1593m, 1555m, 1506m, 1483m, 1445m, 1430m, 1329m, 1300m, 1273m, 1229m, 1190m, 1152m, 1121w, 1084w, 1024m, 953m, 912m, 862m, 849m, 824s, 793w, 756m; ^1^H NMR (CDCl_3_, 500 MHz) δ 9.08 (1H, d, *J* = 5.0 Hz), 8.24 (1H, d, *J* = 7.5 Hz), 8.12 (1H, d, *J* = 5.0 Hz), 7.86 (1H, dd, *J* = 7.5, 2.5 Hz), 7.70 (1H, dd, *J* = 9.0, 4.0 Hz), 7.46 (1H, ddd, *J* = 9.0, 9.0, 2.5 Hz), 6.68 (1H, d, *J* = 8.0 Hz); ^13^C{^1^H} NMR (CDCl_3_, 125 MHz) δ 179.1 (s), 160.2 (d, ^1^*J*_CF_ = 244.7 Hz), 147.3 (d), 139.4 (s), 135.6 (s), 135.4 (s), 133.1 (d, ^4^*J*_CF_ = 3.6 Hz), 131.5 (d), 126.0 (d, ^3^*J*_CF_ = 9.1 Hz), 119.0 (d), 118.6 (d, ^2^*J*_CF_ = 25.7 Hz), 117.7 (d), 112.0 (d, ^3^*J*_CF_ = 9.1 Hz), 110.6 (d, ^2^*J*_CF_ = 24.8 Hz); *m*/*z* (ESI) 261 (M^+^ + Na, 48%), 239 (MH^+^, 100%). Anal. Calcd for C_14_H_7_FN_2_O: C, 70.59; H, 2.96; N, 11.76. Found: C, 70.63; H, 2.70; N, 11.82.

#### 4*H*-Indolo[3,2,1-*ij*][1,6]naphthyridin-4-ones (**9**) (general procedure)

A glass vessel tube used as the microwave reactor was charged with 8-bromo-1,6-naphthyridin-4(1*H*)-one (45.0 mg, 0.2 mmol), K_2_CO_3_ (55.3 mg, 0.4 mmol), PdCl_2_(dppf)·DCM (8.2 mg, 0.01 mmol, 5 mol %), and the appropriate 2-chlorophenylboronic acid (1.5 or 3 equiv) in dioxane/H_2_O (75:25, 2 mL). The tube was capped, and the reaction performed in the microwave reactor at 120 °C (*P* = 150 W; pressure = 50 psi) for 45 min. The mixture was left to cool to rt; then K_2_CO_3_ (55.3 mg, 0.4 mmol) and a premixed solution of CuI (3.8 mg, 10 mol %) and DMCDA (5.4 μL, 20 mol %) in dioxane/H_2_O (75:25, 500 μL) were added to the reaction mixture, and the mixture was placed back into the microwave reactor for 6 h. The mixture was left to cool to rt, diluted (DCM, 20 mL), dried (Na_2_SO_4_), filtered, and evaporated to dryness. The remaining residue was dissolved in DCM and adsorbed onto silica, and chromatography gave 4*H*-indolo[3,2,1-*ij*][1,6]naphthyridin-4-one **9**.

#### 9-Methyl-4*H*-indolo[3,2,1-*ij*][1,6]naphthyridin-4-one (**9b**)

2-Chloro-4-methylphenylboronic acid (51.1 mg, 0.3 mmol) gave compound **9b** (chromatography eluent, 70:30 DCM/THF) as colorless needles (61.9 mg, 88%): mp (hot stage) 253.5–254.5 °C (*c*-hexane/EtOH); *R*_*f*_ = 0.37 (94:6 EtOAc/THF); λ_max_ (DCM, nm) 266 (log ε = 3.62), 293 (3.84), 332 inf (3.66), 236 (4.18), 245 inf (4.21), 358 (3.84), 366 inf (3.89); *v*_max_ (ATR, cm^–1^) 3075w and 3048w (aryl C–H), 1655m, 1649s, 1626s, 1560m, 1541w, 1504s, 1468m, 1418m, 1375m, 1327m, 1288m, 1229m, 1211s, 1179w, 1165m, 1121m, 964w, 907m, 872m, 854m, 843m, 808s, 785w, 721w; ^1^H NMR (CDCl_3_, 500 MHz) δ 9.13 (1H, d, *J* = 4.8 Hz), 8.46 (1H, s), 8.32 (1H, d, *J* = 7.8 Hz), 8.21 (1H, d, *J* = 4.8 Hz), 8.00 (1H, d, *J* = 8.5 Hz), 7.87 (1H, *J* = 8.5 Hz), 6.73 (1H, d, *J* = 7.8 Hz); ^13^C{^1^H} NMR (CDCl_3_, 125 MHz) δ 179.2 (s), 147.6 (d), 140.7 (s), 139.2 (s), 135.6 (s), 132.7 (s), 131.4 (d), 128.1 (q, ^3^*J*_CF_ = 3.6 Hz), 127.4 (q, ^2^*J*_CF_ = 33.1 Hz), 124.8 (s), 124.0 (q, ^1^*J*_CF_ = 272.2 Hz), 121.5 (q, ^3^*J*_CF_ = 3.8 Hz), 119.1 (d), 118.5 (d), 111.5 (d); *m*/*z* (ESI) 273 (M^+^ + K, 5%), 257 (M^+^ + Na, 30%), 235 (MH^+^, 100%). Anal. Calcd for C_15_H_10_N_2_O: C, 76.91; H, 4.30; N, 11.96. Found: C, 77.08; H, 4.12; N, 12.04.

#### 9-Methoxy-4*H*-indolo[3,2,1-*ij*][1,6]naphthyridin-4-one (**9c**)

2-Chloro-4-methoxyphenylboronic acid (111.8 mg, 0.6 mmol) gave compound **9c** (chromatography eluent, EtOAc) as beige microcrystalline powder (51.5 mg, 68%): mp (DSC) 296.3 °C, peak max 297.8 °C (PhMe); *R*_*f*_ = 0.51 (50:50 EtOAc/THF); λ_max_ (DCM, nm) 248 inf (log ε = 4.12), 257 inf (3.99), 269 inf (4.22), 273 (4.26), 297 inf (4.23), 301 (4.27), 352 (4.15); *v*_max_ (ATR, cm^–1^) 3072w and 3034w (aryl C–H), 1657m, 1643m, 1612s, 1597m, 1555m, 1510s, 1474m, 1452m, 1416w, 1377w, 1331w, 1302m, 1226s, 1192m, 1169m, 984w, 903w, 872m, 818m, 745w; ^1^H NMR (DMSO-*d*_6_, 500 MHz) δ 9.44 (1H, s), 9.13 (1H, s), 9.02 (1H, d, *J* = 7.5 Hz), 8.20 (1H, d, *J* = 8.5 Hz), 7.84 (1H, d, *J* = 2.0 Hz), 7.10 (1H, dd, *J* = 8.5, 2.5 Hz), 6.50 (1H, d, *J* = 7.5 Hz), 3.92 (3H, s); ^13^C{^1^H} NMR (CDCl_3_, 125 MHz) δ 178.1 (s), 160.8 (s), 143.4 (d), 143.2 (d), 141.6 (s), 139.9 (s), 135.1 (d), 123.8 (d), 121.5 (s), 117.8 (s), 116.7 (d), 116.2 (s), 112.3 (d), 97.4 (d), 56.0 (q); *m*/*z* (ESI) 289 (M^+^ + K, 10%), 273 (M^+^ + Na, 40%), 251 (MH^+^, 100%). Anal. Calcd for C_15_H_10_N_2_O_2_: C, 71.99; H, 4.03; N, 11.19. Found: C, 72.05; H, 3.86; N, 11.30.

#### 9-(Trifluoromethyl)-4*H*-indolo[3,2,1-*ij*][1,6]naphthyridine-4-one (**9d**)

2-Chloro-4-(trifluoromethyl)phenylboronic acid (67.3 mg, 0.6 mmol) gave compound **9d** (chromatography eluent, 70:30 DCM/EtOAc) as beige needles (28.3 mg, 49%): mp (hot stage) 261.0–263.5 °C (PhMe); *R*_*f*_ = 0.50 (94:6 EtOAc/THF); λ_max_ (DCM, nm) 248 (log ε = 4.16), 260 (4.24), 279 (4.12), 287 (4.14), 293 inf (4.06), 310 inf (3.58), 346 inf (3.81), 355 (3.95), 370 (3.95); *v*_max_ (ATR, cm^–1^) 3066w (aryl C–H), 1665m, 1649m, 1624m, 1585m, 1560, 1504m, 1468m, 1456m, 1416w, 1385w, 1364w, 1337m, 1325m, 1292m, 1267m, 1223m, 1200m, 1163s 1136m, 1121s, 1057s, 1022m, 970, 920m, 891m, 826m; ^1^H NMR (CDCl_3_, 500 MHz) δ 9.54 (2H, br s), 8.36 (1H, d, *J* = 7.5 Hz), 8.33 (1H, d, *J* = 8.5 Hz), 8.01 (1H, s), 7.82 (1H, d, *J* = 8.0 Hz), 6.67 (1H, d, *J* = 7.5 Hz); ^13^C{^1^H} NMR (CDCl_3_, 125 MHz) δ 178.8 (s), 143.0 (s), 137.9 (s), 132.3 (d), 131.3 (q, ^2^*J*_CF_ = 41.7 Hz), 127.3 (s), 123.9 (q, ^1^*J*_CF_ = 275.0 Hz), 123.5 (d), 122.1 (q, ^3^*J*_CF_ = 3.7 Hz), 118.8 (d), 118.7 (s), 108.2 (q, ^3^*J*_CF_ = 4.1 Hz); *m*/*z* (ESI) 289 (MH^+^, 100%). Anal. Calcd for C_15_H_7_F_3_N_2_O: C, 62.51; H, 2.45; F, 19.77; N, 9.72. Found: C, 62.37; H, 2.58; N, 19.63.

#### 9-Fluoro-4*H*-indolo[3,2,1-*ij*][1,6]naphthyridin-4-one (**9e**)

2-Chloro-4-fluorophenylboronic acid (52.3 mg, 0.3 mmol) gave compound **9e** (chromatography eluent. 70:30 DCM/EtOAc) as beige needles (25.9 mg, 36%): mp (DSC) onset 275.6 °C, peak max 276.6 °C (PhCl); *R*_*f*_ = 0.47 (94:6 EtOAc/THF); λ_max_ (DCM, nm) 263 (log ε = 4.34), 286 inf (4.18), 291 (4.20), 328 inf (3.77), 352 (4.07), 359 inf (4.04); *v*_max_ (ATR, cm^–1^) 3086w and 3063w (aryl C–H), 1665s, 1649m, 1620s, 1601m, 1541w, 1505s, 1456m, 1423m, 1375w, 1335m, 1288m, 1267m, 1227m, 1206s, 1173m, 1153m, 1082m, 1022m, 986m, 872m, 845m, 827m, 804m, 783w; ^1^H NMR (CDCl_3_, 500 MHz) δ 8.90 (1H, d, *J* = 5.5 Hz), 8.33 (1H, dd, *J* = 8.5, 5.5 Hz), 8.21 (1H, d *J* = 7.5 Hz), 7.99 (1H, d, *J* = 5.5 Hz), 7.42 (1H, dd, *J* = 8.5, 2.0 Hz), 7.26 (1H, ddd, *J* = 8.5, 8.5, 2.2 Hz), 6.55 (1H, d, *J* = 8.0 Hz); ^13^C{^1^H} NMR (CDCl_3_, 125 MHz) δ 179.2 (s), 164.5 (d, ^1^*J*_CF_ = 250.0 Hz), 147.3 (s), 145.9 (d), 140.7 (d, ^3^*J*_CF_ = 12.5 Hz), 132.4 (d), 132.2 (s), 127.2 (s), 124.6 (d, ^3^*J*_CF_ = 12.5 Hz), 121.6 (s), 116.8 (d), 115.4 (d), 113.2 (d, ^2^*J*_CF_ = 25.0 Hz), 98.0 (d, ^2^*J*_CF_ = 25.0 Hz); *m*/*z* (ESI) 261 (M^+^ + Na, 100%), 239 (MH^+^, 80%). Anal. Calcd for C_14_H_7_FN_2_O: C, 70.59; H, 2.96; N, 11.76. Found: C, 70.63; H, 3.08; N, 11.89.

#### 10-Methoxy-4*H*-indolo[3,2,1-*ij*][1,6]naphthyridin-4-one (**9f**)

2-Chloro-5-methoxyphenylboronic acid (55.9 mg, 0.3 mmol) gave compound **9f** (chromatography eluent, 90:10 EtOAc/THF) as yellow needles (42.7 mg, 85%): mp (hot stage) 229.0–230.5 °C; *R*_*f*_ = 0.32 (94:6 EtOAc/THF); λ_max_ (DCM, nm) 270 (log ε = 3.56), 305 (3.79), 310 inf (3.92), 384 (3.92); *v*_max_ (ATR, cm^–1^) 3360w (aryl C–H), 1657s, 1651m, 1620m, 1614m, 1585s, 1551m, 1503sm 1485s, 1454w, 1435m, 1422m, 1375w, 1337w, 1283s, 1238m, 1215s, 1155m, 1136m, 1119m, 1092m, 1036m, 957m, 91-m, 849m, 820s, 785m, 752w; ^1^H NMR (CDCl_3_, 500 MHz) δ 9.48 (1H, br s), 9.39 (1H, br s), 8.23 (1H, d, *J* = 8.0 Hz), 7.63 (1H, d, *J* = 2.5 Hz), 7.60 (1H, d, *J* = 8.5 Hz), 7.20 (1H, dd, *J* = 9.0, 2.5 Hz), 6.57 (1H, d, *J* = 7.5 Hz), 3.97 (3H, s); ^13^C{^1^H} NMR (CDCl_3_, 125 MHz) δ 179.0 (s), 157.9 (s), 146.2 (d), 144.2 (d), 142.5 (s), 132.69 (d), 132.67 (s), 125.6 (s), 117.7 (d), 116.6 (d), 111.4 (d), 106.8 (d), 56.3 (q), two C (s) resonances missing; *m*/*z* (ESI) 273 (M^+^ + Na, 37%), 251 (MH^+^, 100%). Anal. Calcd for C_15_H_10_N_2_O_2_: C, 71.99; H, 4.03; N, 11.19. Found: C, 72.03; H, 4.11; N, 11.06.

#### 10-(Trifluoromethoxy)-4*H*-indolo[3,2,1-*ij*][1,6]naphthyridin-4-one (**9g**)

2-Chloro-5-(trifluoromethoxy)phenylboronic acid (144.2 mg, 0.6 mmol) gave compound **9g** (chromatography eluent, 80:20 DCM/THF) as beige microcrystalline powder (15.8 mg, 26%): mp (DSC) onset 245.0 °C, peak max 246.9 °C; *R*_*f*_ = 0.52 (94:6 EtOAc/THF); λ_max_ (DCM, nm) 260 (log ε = 3.81), 280 inf (3.76), 284 inf (3.81), 288 (3.88), 294 inf (3.85), 313 inf (3.32), 358 (3.71), 370 (3.70); *v*_max_ (ATR, cm^–1^) 1659s, 1649m, 1628m, 1614m, 1585m, 1562m, 1543w, 1526w, 1501m, 1477m, 1425m, 1300s, 1285s, 1267s, 1227s, 1207s, 1150s, 1138s, 1094m, 1018w, 966m, 945w, 880m, 831m, 812m; ^1^H NMR (CDCl_3_, 500 MHz) δ 9.55 (1H, br s), 9.49 (1H, s), 8.29 (1H, d, *J* = 8.0 Hz), 8.05 (1H, d, *J* = 1.0 Hz), 7.75 (1H, d, *J* = 9.0 Hz), 7.53 (1H, d, *J* = 9.0 Hz), 6.63 (1H, d *J* = 8.0 Hz); ^13^C{^1^H} NMR (CDCl_3_, 125 MHz) δ 178.8 (s), 146.7 (d), 146.54 (s), 146.52 (s), 144.8 (d), 142.9 (s), 136.5 (s), 132.5 (d), 125.6 (s), 122.4 (d), 120.7 (q, ^1^*J*_CF_ = 262.5 Hz), 118.6 (d), 116.1 (d), 111.6 (d), one C (s) resonance missing; *m*/*z* (ESI) 305 (MH^+^, 28%), 295 (100%). Anal. Calcd for C_15_H_7_F_3_N_2_O_2_: C, 59.22; H, 2.32; N, 9.21. Found: C, 59.47; H, 2.18; N, 9.16.

#### 10-Fluoro-4*H*-indolo[3,2,1-*ij*][1,6]naphthyridin-4-one (**9h**)

2-Chloro-5-fluorophenylboronic acid (52.3 mg, 0.3 mmol) gave compound **9h** (chromatography eluent, 80:20 DCM/THF) as microcrystalline powder (11 mg, 23%): mp (DSC) onset 333.7 °C, onset 335.4 °C (PhMe); *R*_*f*_ = 0.50 (94:6 EtOAc/THF); λ_max_ (DCM, nm) 251 inf (log ε = 4.04), 263 (4.27), 284 inf (4.00), 292 (4.12), 300 (4.09), 314 inf (3.59), 350 inf (3.74), 364 (3.93), 372 inf (3.90); *v*_max_ (ATR, cm^–1^) 3045w (aryl C–H), 1655s (C=O), 1651s, 1620m, 1587m, 1560m, 1503s, 1479s, 1439m, 1427m, 1375m, 1337m, 1290m, 1275m, 1229m, 1202s, 1148m, 1111m, 1092m, 1020m, 961w, 926m, 876m, 816m; ^1^H NMR (DMSO-*d*_6_, 500 MHz) δ 9.59 (1H, s), 9.23 (1H, s), 9.02 (1H, d, *J* = 8.0 Hz), 8.26 (1H, dd, *J* = 8.5, 2.5 Hz), 8.20 (1H, dd, *J* = 9.0, 4.5 Hz), 7.58 (1H, ddd, *J* = 9.0, 9.0, 2.5 Hz), 6.51 (1H, d, *J* = 8.0 Hz); ^13^C{^1^H} NMR (DMSO-*d*_6_, 125 MHz) δ 177.8 (s), 159.7 (d, ^1^*J*_CF_ = 275.0 Hz), 145.3 (d), 144.9 (d), 142.0 (s), 135.3 (d), 124.8 (s), 124.9 (d, ^3^*J*_CF_ = 10.7 Hz), 120.9 (s), 117.9 (s), 116.7 (d), 116.3 (d, ^2^*J*_CF_ = 25.0 Hz), 113.5 (d, ^3^*J*_CF_ = 12.5 Hz), 109.7 (d, ^2^*J*_CF_ = 25.0 Hz); *m*/*z* (ESI) 261 (M^+^ + Na, 79%), 239 (MH^+^, 100%). Anal. Calcd for C_14_H_7_FN_2_O: C, 70.59; H, 2.96; N, 11.76. Found: C, 70.64; H, 3.07; N, 11.93.

### 4*H*-Indolo[3,2,1-*ij*][1,7]naphthyridin-4-ones (**10**) (general procedure)

A mixture of 8-bromo-1,7-naphthyridin-4(1*H*)-one (**15**) (67.5 mg, 0.3 mmol), K_2_CO_3_ (83.1 mg, 0.6 mmol), PdCl_2_(dppf)·DCM (12.0 mg, 0.015 mmol, 5 mol %), and the appropriate 2-chlorophenylboronic acid (1.5 or 3 equiv) in dioxane/H_2_O (75:25, 2 mL) was heated at ∼88 °C (reflux) until the starting material had been completely consumed (as determined by TLC) (see Table 1). Then the mixture was left to cool to rt; K_2_CO_3_ (83.1 mg, 0.6 mmol) and a premixed solution of CuI (5.7 mg, 10 mol %) and DMCDA (9.45 μL, 20 mol %) in dioxane/H_2_O (75:25, 300 μL) were added to the reaction mixture, and the mixture was heated back to reflux for 12 h. The mixture was left to cool to rt, diluted (DCM, 20 mL), dried (Na_2_SO_4_), filtered, and evaporated to dryness. The remaining residue was dissolved in DCM, adsorbed onto silica, and chromatographed to give 4*H*-indolo[3,2,1-*ij*][1,7]naphthyridin-4-one **10**.

#### 8-Chloro-4*H*-indolo[3,2,1-*ij*][1,7]naphthyridin-4-one (**10b**)

2,3-Dichlorophenylboronic acid (74.2 mg, 0.45 mmol) gave compound **10b** (chromatography eluent, DCM-NH_3_) as yellow needles (35.7 mg, 47%): mp (hot stage) 235.0–237.0 °C (EtOH); *R*_*f*_ = 0.37 (50:50 DCM/EtOAc); λ_max_ (DCM, nm) 249 (log ε = 3.98), 263 (3.95), 284 (4.11), 293 (4.32), 330 inf (4.38), 337 (3.71), 387 inf (4.12), 395 (4.15); *v*_max_ (ATR, cm^–1^) 3125w and 3084w (aryl C–H), 1657m, 1649m, 1628s, 1574m, 1547m, 1485m, 1441s, 1406m, 1350m, 1335m, 1306m, 1285m, 1271w, 1246m, 1182s, 1159m, 1138m, 1105w, 1076w, 1061m, 1038m, 945m, 899m, 822m, 816m, 810m, 772m, 754m, 739m, 731m; ^1^H NMR (DMSO-*d*_6_, 500 MHz) δ 9.24 (1H, d, *J* = 8.0 Hz), 8.94 (1H, d, *J* = 5.5 Hz), 8.28 (1H, dd, *J* = 7.5, 1.0 Hz), 7.97 (1H, d, *J* = 5.0 Hz), 7.81 (1H, dd, *J* = 8.0, 1.0 Hz), 7.55 (1H, dd, *J* = 8.0, 8.0 Hz), 6.46 (1H, d, *J* = 8.0 Hz); ^13^C{^1^H} NMR (DMSO-*d*_6_, 125 MHz) δ 177.8 (s), 146.40 (s), 146.38 (d), 136.5 (d), 134.9 (s), 131.8 (d), 131.4 (s), 127.6 (s), 126.7 (s), 126.2 (d), 120.8 (d), 118.3 (s), 115.6 (d), 115.5 (d); *m*/*z* (ESI) 257 (^37^Cl, MH^+^, 35%), 255 (^35^Cl, MH^+^, 100%). Anal. Calcd for C_14_H_7_ClN_2_O: C, 66.03; H, 2.77; N, 11.00. Found: C, 66.19; H, 2.56; N, 11.23.

#### 9-Methyl-4*H*-indolo[3,2,1-*ij*][1,7]naphthyridin-4-one (**10c**)

2-Chloro-4-methylphenylboronic acid (76.7 mg, 0.45 mmol) gave compound **10c** (55.1 mg, 78%) as pale yellow needles: mp (hot stage) 249.0–251.0 °C (EtOH); *R*_*f*_ = 0.50 (30:70 DCM/EtOAc); λ_max_ (DCM, nm) 243 inf (log ε = 4.22), 251 inf (4.19), 260 inf (4.05), 291 inf (4.31), 298 (4.46), 343 inf (4.03), 359 inf (4.09), 386 (4.32); *v*_max_ (ATR, cm^–1^) 3105w and 3055w (aryl C–H), 2907w (alkyl C–H), 1661m, 1624m, 1614s, 1585m, 1543s, 1508m, 1477m, 1452m, 1395m, 1350m, 1306m, 1273w, 1217w, 1196s, 1167m, 1132m, 1094m, 1028w, 860w, 802s, 787m, 746w; ^1^H NMR (CDCl_3_, 500 MHz) δ 8.84 (1H, d, *J* = 5.5 Hz), 8.23 (1H, d, *J* = 7.5 Hz), 8.21 (1H, d, *J* = 8.0 Hz), 7.96 (1H, d, *J* = 5.5 Hz), 7.48 (1H, s), 7.32 (1H, d, *J* = 8.0 Hz), 6.51 (1H, d, *J* = 7.5 Hz), 2.60 (3H, s); ^13^C{^1^H} NMR (CDCl_3_, 125 MHz) δ 179.4 (s), 148.3 (s), 145.5 (d), 141.7 (s), 140.1 (s), 132.5 (d), 131.6 (s), 127.1 (s), 126.6 (d), 122.9 (s), 122.7 (d), 116.2 (d), 115.1 (d), 111.1 (d); *m*/*z* (ESI) 257 (M^+^ + Na, 45%), 235 (MH^+^, 100%). Anal. Calcd for C_15_H_10_N_2_O: C, 76.91; H, 4.30; N, 11.96. Found: C, 77.03; H, 4.16; N, 11.78.

#### 9-Methoxy-4*H*-indolo[3,2,1-*ij*][1,7]naphthyridine-4-one (**10d**)

2-Chloro-4-methoxyphenylboronic acid (83.9 mg, 0.45 mmol) gave compound **10d** (61.0 mg, 81%) as yellow needles: mp (hot stage) 269.5–270.0 °C (PhMe); *R*_*f*_ = 0.56 (50:50 DCM/THF); λ_max_ (DCM, nm) 256 (log ε = 4.28), 266 inf (4.23), 288 inf (4.38), 297 (4.52), 303 (4.51), 375 inf (4.54), 383 (4.57); *v*_max_ (ATR, cm^–1^) 3107w, 3061w and 3042w (aryl C–H), 1940w and 2839w (alkyl C–H), 1612s (C=O), 1584m, 1547s, 1504m, 1477m, 1454m, 1435s, 1400m, 1354w, 1287m, 1279m, 1223s, 1194s, 1175m, 1130m, 1096m, 1072w, 1036m, 1016m, 968w, 864m, 843w, 808s, 745w; ^1^H NMR (CDCl_3_, 500 MHz) δ 8.81 (1H, d, *J* = 5.0 Hz), 8.21–8.20 (2H, m), 7.89 (1H, d, *J* = 5.5 Hz), 7.15 (1H, d, *J* = 2.0 Hz), 7.05 (1H, dd, *J* = 8.5, 2.0 Hz), 6.5 (1H, d, *J* = 7.5 Hz); ^13^C{^1^H} NMR (CDCl_3_, 125 MHz) δ 179.6 (s), 162.4 (s), 148.5 (s), 146.0 (d), 141.4 (s), 132.4 (d), 131.7 (s), 126.7 (s), 123.7 (d), 118.7 (s), 116.2 (d), 114.2 (d), 111.9 (d), 96.6 (d), 58.2 (q); *m*/*z* (ESI) 273 (M^+^ + Na, 40%), 251 (MH^+^, 100%). Anal. Calcd for C_15_H_10_N_2_O_2_: C, 71.99; H, 4.03; N, 11.19. Found: C, 72.05; H, 4.14; N, 11.03.

#### 9-(Trifluoromethyl)-4*H*-indolo[3,2,1-*ij*][1,7]naphthyridin-4-one (**10e**)

2-Chloro-4-(trifluoromethyl)phenylboronic acid (201.9 mg, 0.9 mmol) gave compound **10e** (chromatography eluent, 80:20 DCM/THF) as yellow needles (40.6 mg, 47%): mp (DSC) onset 332.3 °C, peak max 333.4 °C (EtOH); *R*_*f*_ = 0.43 (50:50 DCM/EtOAc); λ_max_ (DCM, nm) 263 (log ε = 3.80), 292 (4.29), 296 (4.32), 327 (3.80), 333 (3.81), 365 (3.90), 242 (4.17), 384 (4.19), 395 (4.29); *v*_max_ (ATR, cm^–1^) 3046w (aryl C–H), 1659m, 1649m, 1622m, 1551m, 1504m, 1479m, 1454m, 1439m, 1400m, 1327s, 1283m, 1194m, 1171m, 1159s, 1113s, 1074m, 1055m, 1028w, 968w, 893m, 818s, 725w; ^1^H NMR (DMSO-*d*_6_, 500 MHz) δ 9.17 (1H, d, *J* = 8.0 Hz), 8.99 (1H, d, *J* = 5.5 Hz), 8.77 (1H, s), 8.50 (1H, d, *J* = 8.5 Hz), 8.01 (1H, d, *J* = 5.5 Hz), 7.89 (1H, d, *J* = 8.0 Hz), 6.53 (1H, d, *J* = 7.5 Hz); ^13^C{^1^H} NMR (DMSO-*d*_6_, 125 MHz) δ 178.4 (s), 146.4 (d), 146.2 (s), 139.3 (s), 135.4 (d), 131.8 (s), 130.1 (q, ^2^*J*_CF_ = 29.2 Hz), 127.8 (s), 126.5 (s), 124.2 (q, ^1^*J*_CF_ = 270 Hz), 122.6 (d), 121.3 (q, ^3^*J*_CF_ = 3.6 Hz), 116.1 (d), 115.5 (d), 110.2 (q, ^3^*J*_CF_ = 3.9 Hz); *m*/*z* (ESI) 289 (MH^+^, 100%). Anal. Calcd for C_15_H_7_F_3_N_2_O: C, 62.51; H, 2.45; N, 9.72. Found: C, 63.08; H, 2.67; N, 9.83.

#### 9-Chloro-4*H*-indolo[3,2,1-*ij*][1,7]naphthyridin-4-one (**10f**)

2,4-Dichlorophenylboronic acid (171.7 mg, 0.9 mmol) gave compound **10f** (chromatography eluent, 90:10 EtOAc/THF) as beige cotton fibers (54.5 mg, 71%): mp (DSC) onset 326.5 °C, peak max 327.7 °C (EtOH); *R*_*f*_ = 0.64 (30:70 DCM/EtOAc); λ_max_ (DCM, nm) 242 (log ε = 4.14), 263 (3.88), 283 inf (4.06), 290 inf (4.24), 294 inf (4.29), 298 (4.45), 335 inf (3.89), 347 inf (3.95), 380 inf (4.23), 387 (4.27); *v*_max_ (ATR, cm^–1^) 3055w (aryl C–H), 1663m, 1618m, 1587m, 1545m, 1499m, 1431m, 1395m, 1352m, 1306m, 1279m, 1261w, 1194m, 1123m, 1074m, 1063m, 1030w, 966w, 808s; ^1^H NMR (DMSO-*d*_6_, 500 MHz) δ 8.99 (1H, d, *J* = 7.5 Hz), 8.89 (1H, d, *J* = 5.5 Hz), 8.43 (1H, d, *J* = 2.0 Hz), 8.25 (1H, d, *J* = 8.5 Hz), 7.91 (1H, d, *J* = 5.5 Hz), 7.57 (1H, dd, *J* = 8.0, 2.0 Hz), 8.48 (1H, d, *J* = 8.0 Hz); ^13^C{^1^H} NMR (DMSO-*d*_6_, 125 MHz) δ 178.4 (s), 146.7 (s), 146.1 (d), 140.3 (s), 135.2 (d), 134.8 (s), 131.2 (s), 126.3 (s), 124.9 (d), 123.4 (s), 123.0 (d), 115.5 (d), 115.2 (d), 113.0 (d); *m*/*z* (ESI) 279 (^37^Cl, M^+^ + Na, 11%), 277 (^35^Cl, M^+^ + Na, 24%), 257 (^37^Cl, MH^+^, 40%), 255 (^35^Cl, MH^+^, 100%). Anal. Calcd for C_14_H_7_ClN_2_O: C, 66.03; H, 2.77; N, 11.00. Found: C, 66.16; H, 2.49; N, 11.12.

#### 9-Fluoro-4*H*-indolo[3,2,1-*ij*][1,7]naphthyridin-4-one (**10g**)

2-Chloro-4-fluorophenylboronic acid (78.5 mg, 0.45 mmol) gave compound **10g** (chromatography eluent, 80:20 DCM/THF) as beige needles (47.7 mg, 67%): mp (hot stage) 277.6–279.8 °C (EtOH); *R*_*f*_ = 0.48 (80:20 DCM/THF); λ_max_ (DCM, nm) 242 (log ε = 4.17), 262 (3.96), 286 (4.26), 291 (4.31), 295 (4.44), 353 inf (4.00), 373 inf (4.25), 381 (4.29); *v*_max_ (ATR, cm^–1^) 3055w (aryl C–H), 1659m, 1649m, 1620s, 1587m, 1547m, 1505s, 1477m, 1447m, 1393w, 1350m, 1317m, 1281m, 1196s, 1171m, 1111w, 1090m, 1024w, 980w, 864m, 833m, 806s, 789w; ^1^H NMR (CDCl_3_, 500 MHz) δ 8.90 (2H, d, *J* = 5.5 Hz), 8.32 (1H, dd, *J* = 8.5, 5.5 Hz), 8.21 (1H, d, *J* = 8.0 Hz), 7.98 (1H, d, *J* = 5.5 Hz), 7.41 (1H, dd, *J* = 8.0, 2.0 Hz), 8.25 (1H, ddd, *J* = 8.5, 8.5, 2.0 Hz), 6.55 (1H, d, *J* = 8.0 Hz); ^13^C{^1^H} NMR (CDCl_3_, 125 MHz) δ 179.4 (s), 164.3 (d, ^1^*J*_CF_ = 250 Hz), 147.6 (s), 146.5 (d), 140.6 (d, ^3^*J*_CF_ = 11.5 Hz), 132.3 (d), 132.0 (s), 127.0 (s), 124.2 (d, ^3^*J*_CF_ = 10.4 Hz), 122.0 (s), 116.7 (d), 115.3 (d), 113.1 (d, ^2^*J*_CF_ = 25.0 Hz), 99.0 (d, ^2^*J*_CF_ = 25.0 Hz); *m*/*z* (ESI) 239 (MH^+^, 100%). Anal. Calcd for C_14_H_7_FN_2_O: C, 70.59; H, 2.96; N, 11.76. Found: C, 70.50; H, 2.79; N, 11.63.

#### 10-Methoxy-4*H*-indolo[3,2,1-*ij*][1,7]naphthyridin-4-one (**10h**)

2-Chloro-5-methoxyphenylboronic acid (83.9 mg, 0.45 mmol) gave compound **10h** (chromatography eluent, 70:30 DCM/THF) as yellow needles (50.1 mg, 66%): mp (hot stage) 236.0–237.2 °C (EtOH); *R*_*f*_ = 0.34 (80:20 DCM/THF); λ_max_ (DCM, nm) 243 inf (log ε = 4.28), 271 (4.06), 280 inf (4.06), 291 inf (4.14), 308 inf (4.41), 311 (4.43), 342 (4.17), 407 (4.10); *v*_max_ (ATR, cm^–1^) 3084w and 3024w (aryl C–H), 2955w and 2932w (alkyl C–H), 1647m, 1618m, 1593m, 1541s, 1497m, 1485s, 1464m, 1433m, 1395m, 1356m, 1315m, 1285w, 1263m, 1227s, 1200m, 1188m, 1146m, 1121m, 1096m, 1040m, 943w, 912m, 860w, 810s, 795m; ^1^H NMR (CDCl_3_, 500 MHz) δ 8.88 (1H, d, *J* = 5.5 Hz), 8.21 (1H, d, *J* = 7.5 Hz), 8.00 (1H, d, *J* = 5.5 Hz), 7.83 (1H, d, *J* = 2.5 Hz), 7.59 (1H, d, *J* = 8.5 Hz), 7.23 (1H, dd, *J* = 8.5, 2.5 Hz), 6.50 (1H, d, *J* = 7.5 Hz), 3.96 (3H, s); ^13^C{^1^H} NMR (CDCl_3_, 125 MHz) δ 179.3 (s), 157.9 (s), 148.4 (s), 145.9 (d), 134.0 (s), 132.7 (d), 132.0 (s), 127.3 (s), 126.8 (s), 118.6 (d), 115.8 (d), 115.7 (d), 111.5 (d), 105.7 (d), 56.2 (q); *m*/*z* (ESI) 251 (MH^+^, 100%). Anal. Calcd for C_15_H_10_N_2_O_2_: C, 71.99; H, 4.03; N, 11.19. Found: C, 72.06; H, 3.96; N, 11.22.

#### 10-(Trifluoromethoxy)-4*H*-indolo[3,2,1-*ij*][1,7]naphthyridin-4-one (**10i**)

2-Chloro-5-(trifluoromethoxy)phenylboronic acid (110.4 mg, 0.45 mmol) gave compound **10i** (chromatography eluent, 30:70 DCM/EtOAc) as pale yellow needles (56.7 mg, 62%): mp (hot stage) 197.6–199.0 °C (EtOH); *R*_*f*_ = 0.63 (30:70 DCM/EtOAc); λ_max_ (DCM, nm) 240 inf (log ε = 4.10), 261 (3.83), 291 inf (4.23), 297 (4.29), 330 inf (3.81), 337 (3.82), 385 inf (4.15), 392 (4.16); *v*_max_ (ATR, cm^–1^) 3030w (aryl C–H), 1657m, 1622m, 1589m, 1549m, 1495m, 1464m, 1431w, 1404w, 1354m, 1333m, 1248m, 1194s, 1173s, 1098m, 1024w, 962w, 920w, 876m, 816m; ^1^H NMR (DMSO-*d*_6_, 500 MHz) δ 9.01 (1H, d, *J* = 8.0 Hz), 8.89 (1H, d, *J* = 5.5 Hz), 8.29 (1H, d, *J* = 9.0 Hz), 9.19 (1H, s), 7.92 (1H, d, *J* = 5.0 Hz), 7.78 (1H, d, *J* = 8.0 Hz), 6.45 (1H, d, *J* = 7.5 Hz); ^13^C{^1^H} NMR (DMSO-*d*_6_, 125 MHz) δ 178.2 (s), 146.4 (s), 146.0 (d), 145.1 (s), 138.0 (s), 135.2 (d), 131.6 (s), 126.4 (s), 125.7 (s), 123.5 (d), 120.2 (q, ^1^*J*_CF_ = 258.3 Hz), 115.7 (d), 115.3 (d), 114.6 (d), 113.9 (d); *m*/*z* (ESI) 305 (MH^+^, 100%). Anal. Calcd for C_15_H_7_F_3_N_2_O_2_: C, 59.22; H, 2.32; N, 9.21. Found: C, 59.36; H, 2.19; N, 9.28.

#### 10-Chloro-4*H*-indolo[3,2,1-*ij*][1,7]naphthyridine-4-one (**10j**)

2,5-Dichlorophenylboronic acid (171.7 mg, 0.9 mmol) gave compound **10j** (chromatography eluent, 30:70 DCM/EtOAc) as yellow needles (46.7 mg, 65%): mp (hot stage) 290.0–291.0 °C (EtOH); *R*_*f*_ = 0.59 (30:70 DCM/EtOAc); λ_max_ (DCM, nm) 244 inf (log ε = 3.99), 264 (3.84), 296 inf (4.22), 301 (4.32), 332 inf (3.82), 338 (3.83), 392 (4.10), 396 inf (4.09); *v*_max_ (ATR, cm^–1^) 3086w and 3026w (aryl C–H), 2924w (alkyl C–H), 1657m, 1620s, 1584m, 1549m, 1497m, 1468m, 1452m, 1423m, 1396m, 1352m, 1317m, 1288w, 1269w, 1260w, 1192m, 1157w, 1132w, 1101w, 1080m, 1065w, 1026w, 901m, 883m, 812s; ^1^H NMR (DMSO-*d*_6_, 500 MHz) δ 9.02 (1H, d, *J* = 8.0 Hz), 8.91 (1H, d, *J* = 5.5 Hz), 8.29 (1H, d, *J* = 2.0 Hz), 8.23 (1H, d, *J* = 8.5 Hz), 7.95 (1H, d, *J* = 5.5 Hz), 7.82 (1H, dd, *J* = 8.0, 2.0 Hz), 8.47 (1H, d, *J* = 7.5 Hz); ^13^C{^1^H} NMR (DMSO-*d*_6_, 125 MHz) δ 178.2 (s), 146.4 (s), 146.0 (d), 138.2 (s), 135.2 (d), 131.3 (s), 130.1 (d), 129.3 (s), 126.4 (s), 126.1 (s), 121.3 (d), 115.6 (d), 115.3 (d), 114.0 (d); *m*/*z* (ESI) 255 (MH^+^, 100%). Anal. Calcd for C_14_H_7_ClN_2_O: C, 66.03; H, 2.77; N, 11.00. Found: C, 66.11; H, 2.68; N, 11.09.

#### 10-Fluoro-4*H*-indolo[3,2,1-*ij*][1,7]naphthyridin-4-one (**10k**)

2-Chloro-5-fluorophenylboronic acid (78.5 mg, 0.45 mmol) gave compound **10k** (chromatography eluent, 30:70 DCM/EtOAc) as yellow needles (49.8 mg, 70%): mp (DSC) onset 312.4 °C, peak max 313.0 °C (EtOH); *R*_*f*_ = 0.51 (30:70 DCM/EtOAc); λ_max_ (DCM, nm) 233 inf (log ε = 4.04), 241 inf (4.04), 263 (3.73), 284 inf (3.91), 295 inf (4.10), 301 (4.17), 332 inf (3.77), 336 (3.77), 390 (3.99), 397 (3.98); *v*_max_ (ATR, cm^–1^) 3042w (aryl C–H), 1659m, 1649m, 1618m, 1545m, 1497m, 1479m, 1462m, 1454m, 1429m, 1396w, 1354w, 1337m, 1317m, 1294w, 1283w, 1263m, 1196s, 1144w, 1096w, 1024m, 918m, 905m, 822m, 806s; ^1^H NMR (DMSO-*d*_6_, 500 MHz) δ 9.02 (1H, d, *J* = 7.5 Hz), 8.91 (1H, d, *J* = 5.5 Hz), 8.25 (1H, dd, *J* = 8.0, 4.0 Hz), 8.10 (1H, d, *J* = 8.0 Hz), 7.95 (1H, d, *J* = 5.5 Hz), 7.66 (1H, dd, *J* = 8.0, 8.0 Hz), 8.46 (1H, d, *J* = 8.0 Hz); ^13^C{^1^H} NMR (DMSO-*d*_6_, 125 MHz) δ 178.1 (s), 159.6 (d, ^1^*J*_CF_ = 250.0 Hz), 146.9 (s), 145.8 (d), 136.1 (s), 135.3 (d), 131.6 (s), 126.5 (s), 126.1 (d, ^3^*J*_CF_ = 12.5 Hz), 117.6 (d, ^2^*J*_CF_ = 25.0 Hz), 115.6 (d), 115.0 (d), 114.0 (d, ^3^*J*_CF_ = 12.5 Hz), 108.2 (d, ^2^*J*_CF_ = 25.0 Hz); *m*/*z* (ESI) 239 (MH^+^, 100%). Anal. Calcd for C_14_H_7_FN_2_O: C, 70.59; H, 2.96; N, 11.76. Found: C, 70.50; H, 2.85; N, 11.89.

## Data Availability

The data underlying this study are available in the published article and its Supporting Information.

## Supporting Information Available

Discussion of reaction optimization for the synthesis of compound **2**, other experimental procedures, crystallographic data for **S4**, and NMR spectra for all new compounds (PDF)

## Dedication

This paper is dedicated to the memory of Dr Nikos Chronakis.
