# Supplementary material for: Synthesis of Canthin-4-ones and Isocanthin-4-ones via B Ring Construction
Source: J Org Chem. 2024 Apr 24;89(9):6444–55. doi: 10.1021/acs.joc.4c00440 (PMC11077480; doi:10.1021/acs.joc.4c00440)

Supporting Information

Synthesis of canthin-4-ones and isocanthin-4-ones via B ring construction

Maria Koyioni*, Andreas Kourtellaris, and Panayiotis A. Koutentis

*E-mail: mkogio01@ucy.ac.cy

Department of Chemistry, University of Cyprus, P.O. Box 20537, 1678 Nicosia, Cyprus

Contents

S1	Optimization studies	S3
S1.1	Optimized synthesis of 8-bromo-1,5-naphthyridin-4(1*H*)-one (2)	S3
S1.2	Section references	S5
S2	Experimental section	S6
S2.1	General methods and materials	S6
S2.2	Synthesis of Meldrum’s acid ylidenes	S7
S.2.3.	Synthesis of 8-halonaphthyridones	S9
S2.4	Section references	S9
S3	X-Ray crystallographic studies	S10
S3.1	X-Ray crystallographic method	S10
S3.2	Crystallographic data for 8'-chloro-4*H*-[1,4'-bi(1,5-naphthyridine)]-4,8(5*H*)-dione (S4)	S11
S3.3	Section references	S12
S4	^{1}H- and ^{13}C{^{1}H}-NMR spectra of compounds	S13

S1 Optimization studies

S1.1 Optimized synthesis of 8-bromo-1,5-naphthyridin-4(1*H*)-one (2)

Initial attempts to synthesize the desired starting material, 8-bromo-1,5-naphthyridin-4(1*H*)-one (**2**), under the typical thermolysis conditions used for the synthesis of 8-bromoquinolin-4(1*H*)-one,[1] were not successful. The thermolysis of the structurally similar 4-chloropyridyl ylidene **S1** was reported in the patent literature: Heating ylidene **S1** in Ph_2O (*ca.* 0.09 mol/L) at *ca.* 250 °C gives the chloro analogue naphthyridone **S2** in a moderate 48% yield (Scheme S1).[2] These reaction conditions were notably almost seven times as dilute as our initial conditions (*ca.* 0.62 mol/L).

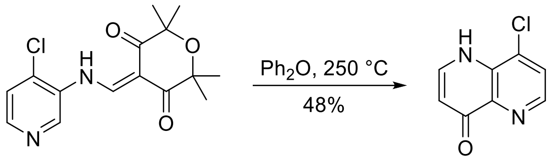

S1 **S2**

Scheme S1. Patented thermolysis of ylidene **S1**

To further investigate this, we looked into the thermolysis of the closely related 5-{[(4-chloropyrid-3-yl)amino]methylene}-2,2-dimethyl-1,3-dioxane-4,6-dione (**S3**). Using approximately the same concentration as in the patent (*ca.* 0.09 mol/L at *ca.* 250 °C) thermolysis of **S3** gave the desired naphthyridone **S2** in 71% yield (Scheme S2). Nevertheless, an unknown side product was also obtained. Based on the spectroscopic data, we initially assigned the structure **S5**, however, X-ray crystallography (see Section **S3**) supported the structure **S4** which also was supported by the available spectroscopic data.

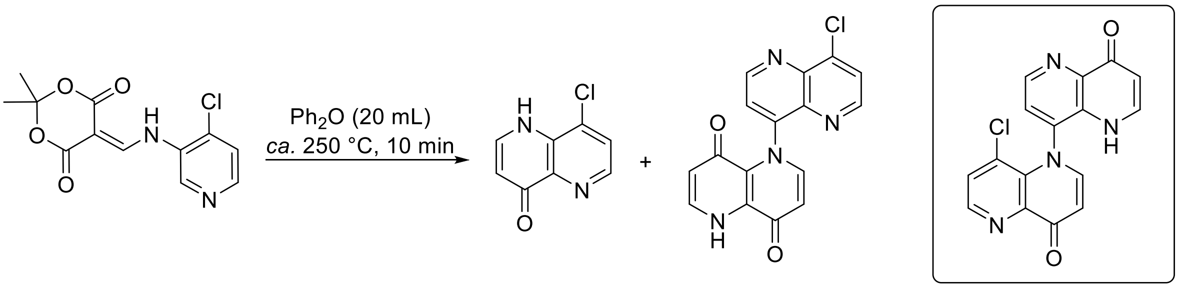

S3 (1 .77 mmol) **S2** (71%) **S4** (8%) **S5**

Scheme S2. Thermolysis of ylidene **S3** to make naphthyridone **S2**.

To rationalize the formation of side product **S4**, we, tentatively, proposed the mechanism in Scheme S2. After initial formation of the 8-chloronaphthyridone **S2**, a sequence of intermolecular and intramolecular reactions between two naphthyridones **S2** can afford the spirocycle **S9**, fragmentation of which via C–O bond breaking gives the thermodynamically stable product **S4** (Scheme S3).

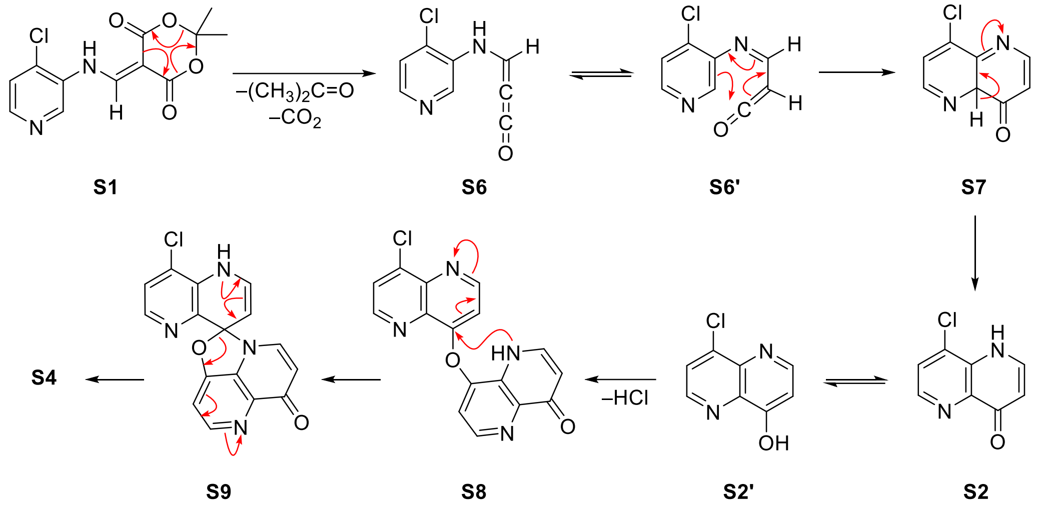

Scheme S3. Speculative mechanism for the formation of side product **S4**

With this result in hand, we reinvestigated the thermolysis of ylidene **1**. Analogously to the chloro derivative the side product **3** along with the naphthyridone **2** was isolated. The yield of the naphthyridone **2** was significantly lower than in the case of the chloro analogue (Table S1, entry 3). This prompted us to investigate the effect of the concentration and reaction time on the outcome of the reaction (Table S1). When the reaction was performed at higher concentrations and extended time, intractable black solids were formed. At high dilution conditions, leaving the reaction for longer also led to formation of black solids that had to be filtered off from the reaction mixture. Both the concentration and the reaction time were crucial for optimum results. The ideal concentration was found to be *ca.* 0.03 mol/L. Further, dilution of the reaction did not have any significant impact on the reaction yield. The external temperature was kept to *ca.* 250 °C and a very short reaction time (0.5 min) was maintained to avoid further reactions. The best yield was obtained at 1.83 mmol scale (52% after recrystallization). We were able to scale up to 2.45 mmol without significant compromise on the yield (46%).

Table S1. Optimization of the thermolysis of ylidene **1** to give the naphthyridone **2**

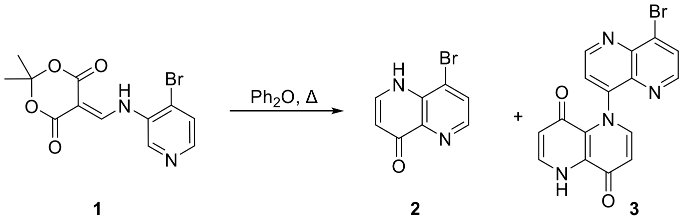

Entry	Scale (mmol)	[1] in Ph_2O (mol/L)	T_{out} (°C)	t (min)	Yields
1	1.53	0.61	230	5	-[a]
2	0.76	0.04	230	8	-[a]
3	1.53	0.08[b]	250	8.5	**2** (12%) + **3** (4%)
4	0.31	0.02	250	3	52%[c]
5	0.61	0.15	250	2	**2** (2%)
6	0.61	0.03	250	2	**2** (40%) + **3** (2%)
7	1.83	0.03	250	0.5	**2** (52%)
8	2.45	0.03	250	0.5	**2** (46%)

[a] Intractable black tar
[b] **1** added portionwise (×5)
[c] Crude mass recovery mixture of **2** & **3** (predominately **2** by ^{1}H-NMR)

S1.2 Section references

1. Broumidis, E.; Koutentis, P. A. A one-pot, two-step synthesis of 3-deazacanthin-4-ones via sequential Pd-catalyzed Suzuki-Miyaura and Cu-catalyzed Buchwald-Hartwig reactions. *Tetrahedron* **2017**, *58*, 2661-2664. DOI: 10.1016/j.tetlet.2017.05.076
2. Wu, H.; Lin, J.; Li, Y.; Wei, C.; Chen, S.; Long, C.; Chen, X.; Liu, Z.; Chen, L. Quinoline derivatives as SMO inhibitors. EP3124482, 2017.

S2 Experimental section

S2.1 General methods and materials

All chemicals were commercially available except those whose synthesis is described. All volatiles were removed under reduced pressure. DCM-NH_3 solvent mixture was prepared by extracting aq. NH_4OH (*ca.* 500 mL) with DCM (4 × 500 mL). The DCM layers were combined, dried (Na_2SO_4), filtered and stored in an amber glass bottle. Dioxane was distilled from CaH_2 before use. The combined DCM extracts were dried (Na_2SO_4) filtered and stored in a glass bottle. All microwave experiments were performed in a CEM Discover Microwave Reactor with an external surface sensor in sealed vials. For conventional heating of the reactions heating blocks were used. All reaction mixtures and column eluents were monitored by TLC using commercial aluminum backed thin layer chromatography (TLC) plates (Kieselgel 60 F_{254}). The plates were observed under UV light at 254 and 365 nm. The technique of dry flash chromatography was used throughout for all non-TLC scale chromatographic separations using silica gel 60 (less than 0.063 mm).[1] Melting points were determined using a PolyTherm-A, Wagner & Munz, Kofler – Hot-stage Microscope apparatus or were determined using a TA Instruments DSC Q1000 with samples hermetically sealed in aluminium pans under an argon atmosphere; using heating rates of 5 °C/min (DSC mp listed by onset and peak values). Solvents used for recrystallization are indicated after the melting points. UV-vis spectra were obtained using a Shimadzu UV-1900 spectrophotometer and inflections are identified by the abbreviation “inf”. IR spectra were recorded on a Shimadzu FTIR-NIR Prestige-21 spectrometer fitted with a Pike Miracle Ge ATR accessory and strong, medium and weak peaks are represented by s, m and w, respectively. ^{1}H and ^{13}C NMR spectra were recorded, as indicated, on a Bruker Avance 300 machine at 300 and 75 MHz, respectively, or on a Bruker Avance 500 machine at 500 and 125 MHz, respectively. Deuterated solvents were used for homonuclear lock, and the signals are referenced to the deuterated solvent peaks. Attached proton test (APT) NMR studies were used for the assignment of the ^{13}C peaks C (quaternary), CH_2, CH_3 and CH_4 as C (s), C (d), C (t) and C (q), respectively. Mass spectrometry was recorded with the Matrix-Assisted Laser Desorption/Ionization Time of Flight (MALDI-TOF) mass spectrometer (+ve mode) on a Bruker Autoflex III Smartbeam instrument or with the Agilent 1260 Infinity II Preparative LC/MSD System. Elemental analysis was performed using a Euro-Vector EA3000 CHN elemental analyzer.

S2.2 Synthesis of Meldrum's acid ylidenes

S2.2.1 5-{[(4-Chloropyrid-3-yl)amino]methylene}-2,2-dimethyl-1,3-dioxane-4,6-dione

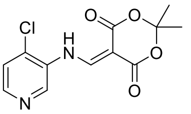

(S1): To a stirred solution of 3-amino-4-chloropyridine (1.0 g, 7.78 mmol) in MeCN (20 mL) was added Meldrum's acid (1.24 g, 8.6 mmol) followed by triethyl orthoformate (2.6 mL, 15.6 mmol). The mixture was heated to *ca.* 82 °C (reflux) for 2 h (until complete consumption of 3-amino-4-chloropyridine by TLC) and then left to cool to rt. The solvent was evaporated to dryness and the residue was recrystallized to give the *title compound* **S1** as beige-brown needles (1.84 g, 83%), mp (DSC) onset: 163.9 °C, peak max: 164.3 °C, decomp. onset: 184.7 °C, peak max: 203.4 °C (EtOH); R_f 0.38 (DCM/*t*-BuOMe, 80:20); Anal. Calcd for $C_{12}H_{11}ClN_2O_4$: C, 50.99; H, 3.92; N, 9.91. Found: C, 50.78; H, 4.01; N, 9.98%; λ_{max}(DCM)/nm 290 (log ε 3.88), 300 (4.06), 325 (4.38); ν_{max}/cm^{-1} (ATR) 3065w (aryl C-H), 3009w & 2994w (alkyl C-H), 1730m (C=O), 1672m, 1607s, 1582w, 1564w, 1501w, 1443m, 1381m, 1329w, 1310s, 1240m, 1225m, 1206m, 1184m, 1167w, 1144m, 1076w, 1022w, 999w, 934m, 891w, 849m, 806m, 791m, 729m; ^{1}H NMR (300 MHz; $CDCl_3$): δ 11.55 (1H, br d, *J* 13.5, N*H*), 8.73 (1H, s, Ar *H*), 8.70 (1H, d, *J* 13.8, C*H*), 8.42 (1H, d, *J* 5.1, Ar *H*), 7.45 (1H, d, *J* 5.1, Ar *H*), 1.77 (6H, s, CH_3); ^{13}C{^{1}H} NMR (75 MHz; $CDCl_3$): δ 165.4 (s), 163.0 (s), 151.9 (d), 147.8 (d), 139.2 (d), 134.2 (s), 132.7 (s), 124.9 (d), 105.8 (s), 90.1 (s), 27.3 (q); *m/z* (ESI) 285 (Cl^{37}: MH^+, 34%), 283 (Cl^{35}: MH^+, 100).

S2.2.2 5-{[(3-Bromopyrid-4-yl)amino]methylene}-2,2-dimethyl-1,3-dioxane-4,6-dione

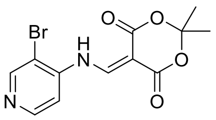

(S10): Prepared according to a literature procedure.[2] A solution of Meldrum's acid (4.32 g, 30.0 mmol) in $CH(OEt)_3$ (30 mL) was heated to *ca.* 146 °C (reflux) for 2 h and then 4-amino-3-bromopyridine (4.33 g, 25.0 mmol) was added and the mixture heated back to *ca.* 146 °C (reflux) for further 2 h. The reaction mixture was left to cool to rt and the precipitated solid was filtered and washed with MeOH and recrystallized to give the *title compound* **9** (4.98 g, 61%) as pale pink needles, mp (DSC) onset: 194.0 °C, peak max: 195.6 °C (EtOH); R_f 0.26 (DCM/*t*-BuOMe, 90:10); Anal. Calcd for $C_{12}H_{11}BrN_2O_4$: C, 44.06; H, 3.39; N, 8.56. Found: C, 44.11; H, 3.28; N, 8.63%; λ_{max}(DCM)/nm 293 (log ε 3.96), 300 (4.51); ν_{max}/cm^{-1} (ATR) 1730m (C=O), 1676m, 1607m, 1582m, 1545m, 1441m, 1416w, 1389w, 1381w, 1369w, 1273s, 1231m, 1207m, 1186w, 1144w, 1030w, 995w, 935w, 864w, 845w, 804m, 727m; ^{1}H NMR (300 MHz; $CDCl_3$): δ 11.64 (1H, br d, *J* 13.5, N*H*), 8.76 (1H, s, Ar *H*), 8.72 (1H, d, *J* 13.5, C*H*), 8.55 (1H, d, *J* 9.0, Ar *H*), 7.30 (1H, d, *J* 9.5, Ar *H*), 1.77 (6H, s, CH_3); ^{13}C{^{1}H} NMR ($CDCl_3$, 75 MHz): δ 164.9 (s), 162.8 (s), 153.5 (d), 150.4 (d), 149.9 (d), 143.0 (s), 111.8 (s), 110.1 (d), 106.0 (s), 91.5 (s), 27.4

(q); *m/z* (ESI) 329 (Br^{81}: MH^+, 64%), 327 (Br^{79}: MH^+, 65), 271 (100), 269 (97), 227 (53), 225 (57).

S2.3 Synthesis of 8-halonaphthyridones

*S2.3.1 Synthesis of 5-{[(4-Chloropyrid-3-yl)amino]methylene}-2,2-dimethyl-1,3-dioxane-4,6-dione (**S2**):* To Ph_2O (5 mL), preheated to *ca.* 250 °C (external temperature), was added in one portion the ylidene **S3** (130 mg, 0.46 mmol) and stirred for 5 min. After this time, the reaction was left to cool to rt, the mixture poured into cold *n*-hexane (*ca.* 200 mL) and the precipitated solid was collected by filtration. The solid was then suspended in hot EtOH and filtered.

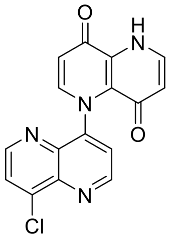

The collected solid was recrystallized to afford *8'-chloro-4*H*-[1,4'-bi(1,5-naphthyridine)]-4,8(5*H*)-dione* (**S4**) as red needles (23.9 mg, 8%), decomp. (DSC) onset: 295.2 °C, peak max: 311.4 °C (EtOH/DMSO); Anal. Calcd for $C_{16}H_9ClN_4O_2$: C, 59.18; H, 2.79; N, 17.25. Found: C, 59.32; H, 2.69; N, 17.30%; λ_{max}(DMSO)/nm 287 inf (log ε 4.05), 302 inf (4.10), 320 inf (4.25), 329 (4.31), 339 (4.34); ν_{max}/cm^{-1} (ATR) 3175w, 3138w & 3090w (aryl C-H), 1601s (C=O), 1574s, 1553m, 1520w, 1491m, 1466m, 1396m, 1387m, 1337w, 1294m, 1275w, 1190s, 1103w, 1086w, 1061w, 1042w, 955m, 868m, 841m, 814m, 791m, 766m, 719w; ^{1}H NMR (DMSO-d_6, 500 MHz): δ 11.97 (1H, br s, N*H*), 9.19 (1H, d, *J* 4.8, Ar *H*), 8.74 (1H, d, *J* 4.8, Ar *H*), 8.05 (1H, d, *J* 4.8, Ar *H*), 8.00 (1H, d, *J* 4.4, Ar *H*), 7.99 (1H, d, *J* 7.8, Ar *H*), 7.77 (1H, br d, *J* 7.2, Ar *H*), 6.46 (1H, d, *J* 7.8, Ar *H*), 6.03 (1H, d, *J* 7.2, Ar *H*); ^{13}C{^{1}H} NMR (DMSO-d_6, 125 MHz): δ 171.8 (s), 171.2 (s), 152.3 (d), 151.0 (d), 149.7 (s), 144.2 (d), 143.0 (s), 141.2 (s), 139.7 (s), 137.7 (d), 132.8 (s), 132.1 (s), 125.2 (d), 121.5 (d), 113.9 (d), 110.9 (d); *m/z* (ESI) 327 (Cl^{37}: M^+, 6%), 325 (Cl^{35}: M^+, 26), 169 (100).

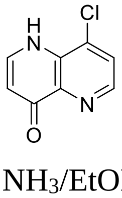

The filtrate was concentrated in vacuo and the residue triturated with *n*-pentane to afford *8-chloro-1,5-naphthyridin-4(1*H*)-one* (**S2**) (59.1 mg, 71%) as beige needles, decomp. (DSC) onset: 149.9 °C, peak max: 154.7 °C (EtOH); R_f 0.11 (DCM-NH_3/EtOH, 82:18); Anal. Calcd for $C_8H_5ClN_2O$: C, 53.21; H, 2.79; N, 15.51. Found: C, 53.18; H, 2.65; N, 15.74%; λ_{max}(EtOH)/nm 235 inf (log ε 4.24), 240 (4.26), 248 (4.21), 268 (3.34), 278 (3.33), 291 (3.29), 320 inf (3.81), 331 (3.95), 340 (3.93); ν_{max}/cm^{-1} (ATR) 3600-2700w br (NH), 1620m (C=O), 1574m, 1557s, 1510s, 1439w, 1404m, 1286m, 1242w, 1217m, 1140w, 1072m, 910w, 856w, 812m; ^{1}H NMR (500 MHz; CD_3OD): δ 8.65 (1H, br s, Ar *H*), 8.07 (1H,

d, *J* 7.4, Ar *H*), 7.90 (1H, d, *J* 4.8, Ar *H*), 6.58 (1H, d, *J* 7.4, Ar *H*); $^{13}C\{^1H\}$ NMR (CD_3OD, 125 MHz): *δ* 178.4 (s), 147.8 (d), 142.4 (d), 141.9 (s), 136.1 (s), 135.9 (s), 128.1 (d), 113.2 (d); *m/z* (ESI) 221 (Cl^{37}: M^++K, 10%), 219 (Cl^{35}: M^++K, 24), 205 (Cl^{37}: M^++Na, 30), 203 (Cl^{35}: M^++Na, 100), 183 (Cl^{37}: MH^+, 19), 181 (Cl^{35}: MH^+, 55).

*S2.3.2 8-Bromo-1,6-naphthyridin-4(1*H*)-one (**11**):* According to a literature procedure,[2] a solution of ylidene **S10** (0.4 g, 1.22 mmol) in Ph_2O (3 mL) was heated to *ca.* 220 °C (external temperature) for 0.5 h. The reaction mixture was cooled to room temperature and poured into *n*-hexane (200 mL). The precipitate was filtered, washed with *n*-hexane, recrystallized and dried in the vacuum oven at *ca.* 100 °C to give the *title compound* **11** as beige needles (200 mg, 72%), mp (DSC) onset: 261.3 °C, peak max: 266.0 °C (PhCl); R_f 0.19 (EtOAc); Anal. Calcd for $C_8H_5BrN_2O$: C, 42.70; H, 2.24; N, 12.45. Found: C, 42.96; H, 2.17; N, 12.52%; λ_{max}(DCM)/nm 215 (log *ε* 4.55), 242 (4.14), 251 inf (4.10), 275 inf (3.91), 279 (3.93), 290 (3.95), 310 inf (3.98), 319 (4.07), 331 (4.05), 351 inf (3.52), 371 inf (3.14), 389 inf (2.77); ν_{max}/cm^{-1} (ATR) 3280-2920w br (NH), 1643m, 1626s, 1599s, 1578m, 1547s, 1493s, 1443w, 1406w, 1391w, 1315s, 1254w, 1219m, 1198s, 1101m, 1057m, 910w, 895w, 837m, 799m, 789m, 773m; ^1H NMR (DMSO-d_6, 500 MHz): *δ* 11.50 (1H, br s, N*H*), 9.12 (1H, s, Ar *H*), 8.84 (1H, s, Ar *H*), 7.91 (1H, d, *J* 7.5, Ar *H*), 6.26 (1H, d, *J* 7.5, Ar *H*); $^{13}C\{^1H\}$ NMR (DMSO-d_6, 125 MHz): *δ* 176.5 (s), 151.4 (d), 148.2 (d), 142.5 (s), 141.4 (d), 121.6 (s), 113.1 (d), 109.3 (s); *m/z* (ESI) 249 (Br^{81}: M^++Na, 98%), 247 (Br^{79}: M^++Na, 100), 226 (Br^{81}: MH^+, 64), 224 (Br^{79}: MH^+, 63).

S2.5 Section references

1. Hardwood, L. M. “Dry-Column” Flash Chromatography. *Aldrichimica Acta* **1985**, *18*, 25-25.
2. Wu, H.; Lin, J.; Li, Y.; Wei, C.; Chen, S.; Long, C.; Chen, X.; Liu, Z.; Chen, L. Quinoline derivatives as SMO inhibitors. EP 3124482 A1, 2017.

S3 X-Ray crystallographic studies

S3.1 X-Ray crystallographic method

Data for compound **S4** was collected on an Oxford-Diffraction Supernova diffractometer, equipped with a CCD area detector utilizing Mo Kα radiation (λ = 0.71073Å). Suitable crystals were attached to glass fibers using paratone-N oil and transferred to a goniostat where they were cooled for data collection. Unit cell dimensions were determined and refined. Empirical absorption corrections (multi-scan based on symmetry-related measurements) were applied using CrysAlis RED software.[1] The structure **S4** was solved by direct method and refined on F2 using full-matrix least squares using SHELXL97.[2] Software packages used: CrysAlis CCD1 for data collection, CrysAlis[1] for cell refinement and data reduction, WINGX for geometric calculations,[3] and DIAMOND[4] for molecular graphics. The non-H atoms were treated anisotropically. The hydrogen atoms were placed in calculated, ideal positions and refined as riding on their respective carbon atoms.

Crystallographic data for **S4** has been deposited with the Cambridge Crystallographic Data Centre with deposit numbers CCDC-2325378. The data can be obtained free of charge via www.ccdc.cam.ac.uk/data_request/cif (or from the Cambridge Crystallographic Data Centre, 12 Union Road, Cambridge CB2 1EZ, UK; fax: +44 1223 336033; or e-mail: deposit@ccdc.cam.ac.uk).

S3.2 Crystallographic data for 8'-chloro-4*H*-[1,4'-bi(1,5-naphthyridine)]-4,8(5*H*)-dione (S4)

**Table S2.** Crystal data and structure refinement for **S4** at 100(2) K.	
Empirical formula	C_{16} H_9Cl N_4 O_2
Formula weight	324.72
Temperature	100(2) K
Wavelength	0.71073 Å
Crystal system	Triclinic
Space group	P -1
Unit cell dimensions	a = 7.2763(14) Å, α = 89.292(13)°<br>b = 8.5693(10) Å, β = 79.474(18)°<br>c = 10.988(3) Å, γ = 82.863(13)°
Volume	668.3(2) $Å^3$
Z	2
Density (calculated)	1.614 g/cm^3
Absorption coefficient	0.303 mm^{-1}
F(000)	332
Crystal size	0.19 × 0.05 × 0.04 mm^3
θ range for data collection	3.503 to 29.007°
Index ranges	-6<=h<=9, -11<=k<=11, -14<=l<=14
Reflections collected	4989
Independent reflections	3065 [R_{int} = 0.0590]
Completeness to θ = 25.242°	99.8%
Refinement method	Full-matrix least-squares on F^2
Data / restraints / parameters	3065 / 0 / 208
Goodness-of-fit	0.995
Final R indices [I > 2σ(I)]	R_{obs} = 0.0837, wR_{obs} = 0.1899
R indices [all data]	R_{all} = 0.1500, wR_{all} = 0.2571
Extinction coefficient	.
Largest diff. peak and hole	0.530 and -0.645 e·$Å^{-3}$

$R = \Sigma||F_o|-|F_c|| / \Sigma|F_o|$, $wR = \{\Sigma[w(|F_o|^2 - |F_c|^2)^2] / \Sigma[w(|F_o|^4)]\}^{1/2}$ and $w=1/[\sigma^2(Fo^2)+(0.1058P)^2]$ where $P=(Fo^2+2Fc^2)/3$

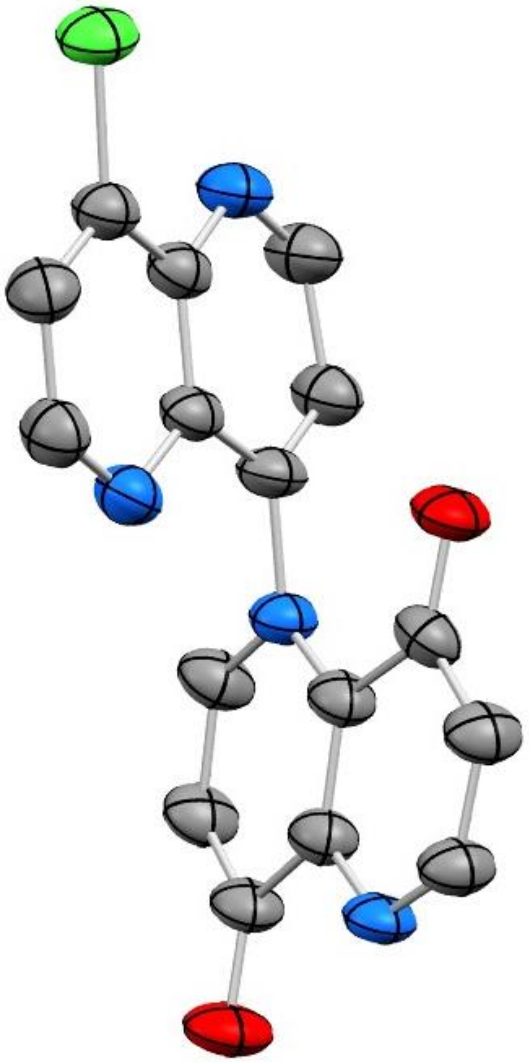

Figure S1. Thermal ellipsoid (50%) representation of the X-ray structure of 8'-chloro-4*H*-[1,4'-bi(1,5-naphthyridine)]-4,8(5*H*)-dione (**S4**). Hydrogens are omitted for clarity.

S3.2 Section references

1. CrysAlis CCD and CrysAlis RED, version 1.171.32.15; Oxford Diffraction Ltd, Abingdon, Oxford, England, 2008.
2. Sheldrick, G. M. SHELXL-97: A program for the refinement of crystal structure; University of Göttingen: Göttingen, Germany, 1997.
3. Farrugia, L. J. *WinGX* suite for small-molecule single-crystal crystallography. *J. Appl. Crystallogr.* **1999**, *32*, 837-838. DOI: 10.1107/S0021889899006020
4. Brandenburg, K. DIAMOND, version 3.1d; Crystal Impact GbR, Bonn, Germany, 2006.

S4 ^{1}H- and $^{13}C\{^{1}H\}$-NMR spectra of compounds

5-{[(4-Chloropyrid-3-yl)amino]methylene}-2,2-dimethyl-1,3-dioxane-4,6-dione (**S1**), ^{1}H NMR in $CDCl_3$, 300 MHz

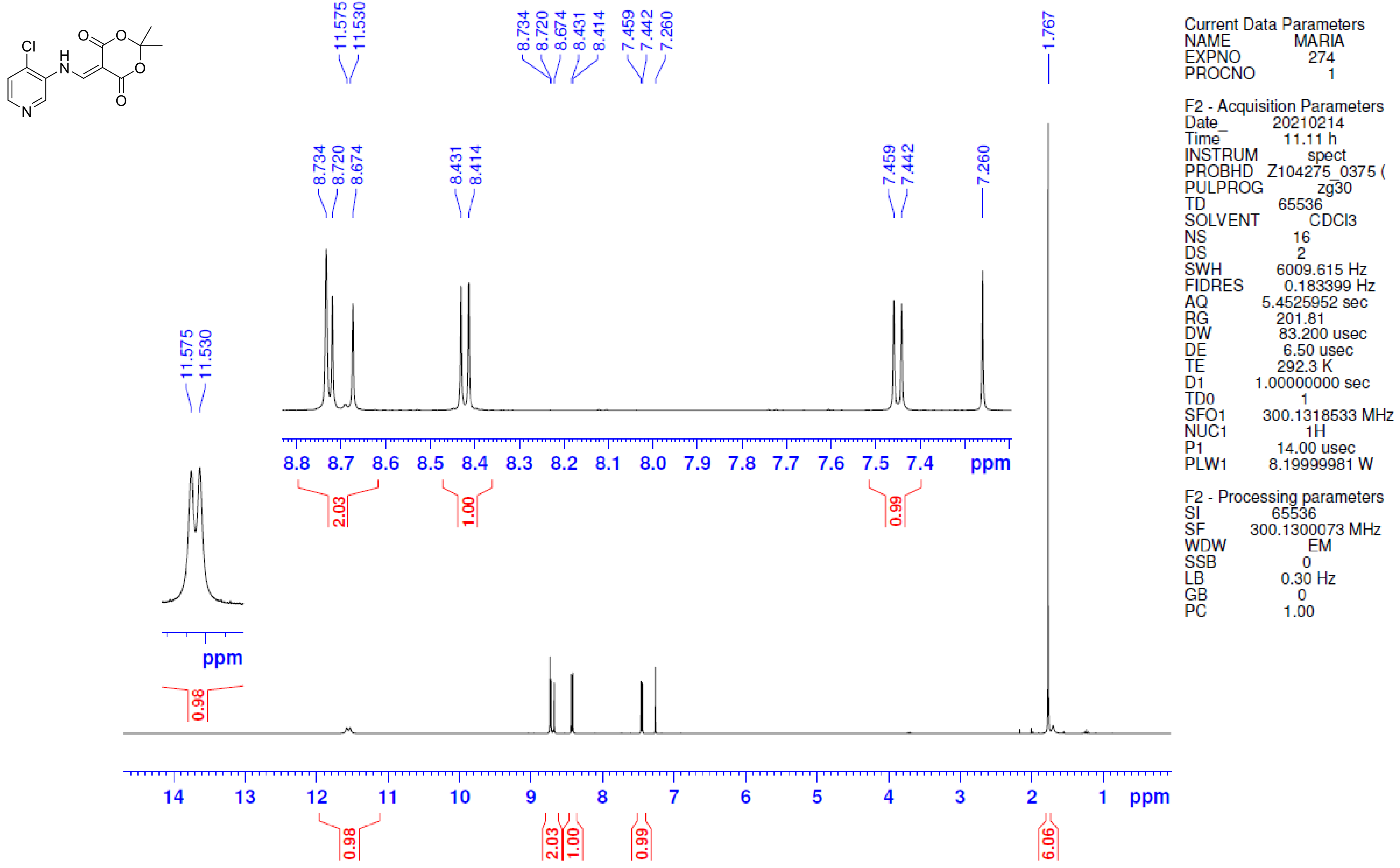

5-{[(4-Chloropyrid-3-yl)amino]methylene}-2,2-dimethyl-1,3-dioxane-4,6-dione (**S1**), ^{13}C NMR in $CDCl_3$, 75 MHz

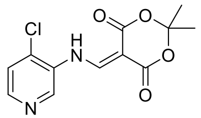

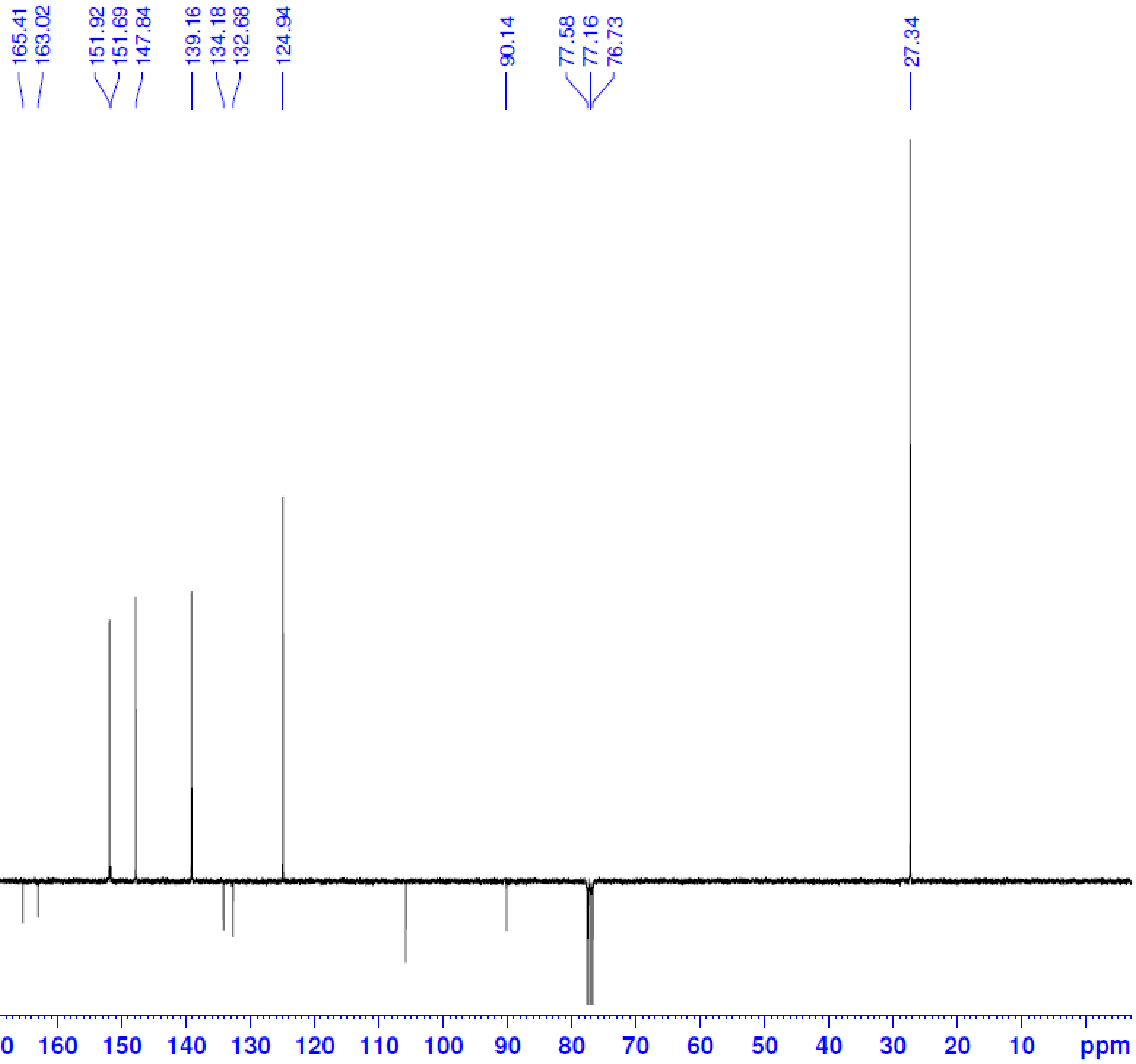

Current Data Parameters
NAME MARIA
EXPNO 275
PROCNO 1

F2 - Acquisition Parameters
Date_ 20210214
Time 18.42 h
INSTRUM spect
PROBHD Z104275_0375 (
PULPROG jmod
TD 65536
SOLVENT CDCl3
NS 6970
DS 4
SWH 18115.941 Hz
FIDRES 0.552855 Hz
AQ 1.8087935 sec
RG 201.81
DW 27.600 usec
DE 6.50 usec
TE 294.9 K
CNST2 145.0000000
CNST11 1.0000000
D1 2.00000000 sec
D20 0.00689655 sec
TD0 1
SFO1 75.4752953 MHz
NUC1 13C
P1 10.00 usec
P2 20.00 usec
PLW1 41.00000000 W
SFO2 300.1312005 MHz
NUC2 1H
CPDPRG[2 waltz16
PCPD2 80.00 usec
PLW2 8.19999981 W
PLW12 0.25112000 W

F2 - Processing parameters
SI 32768
SF 75.4677404 MHz
WDW EM
SSB 0
LB 1.00 Hz
GB 0
PC 1.40

5-{[(4-Chloropyrid-3-yl)amino]methylene}-2,2-dimethyl-1,3-dioxane-4,6-dione (**S2**), ^{1}H NMR in MeOH-d_4, 500 MHz

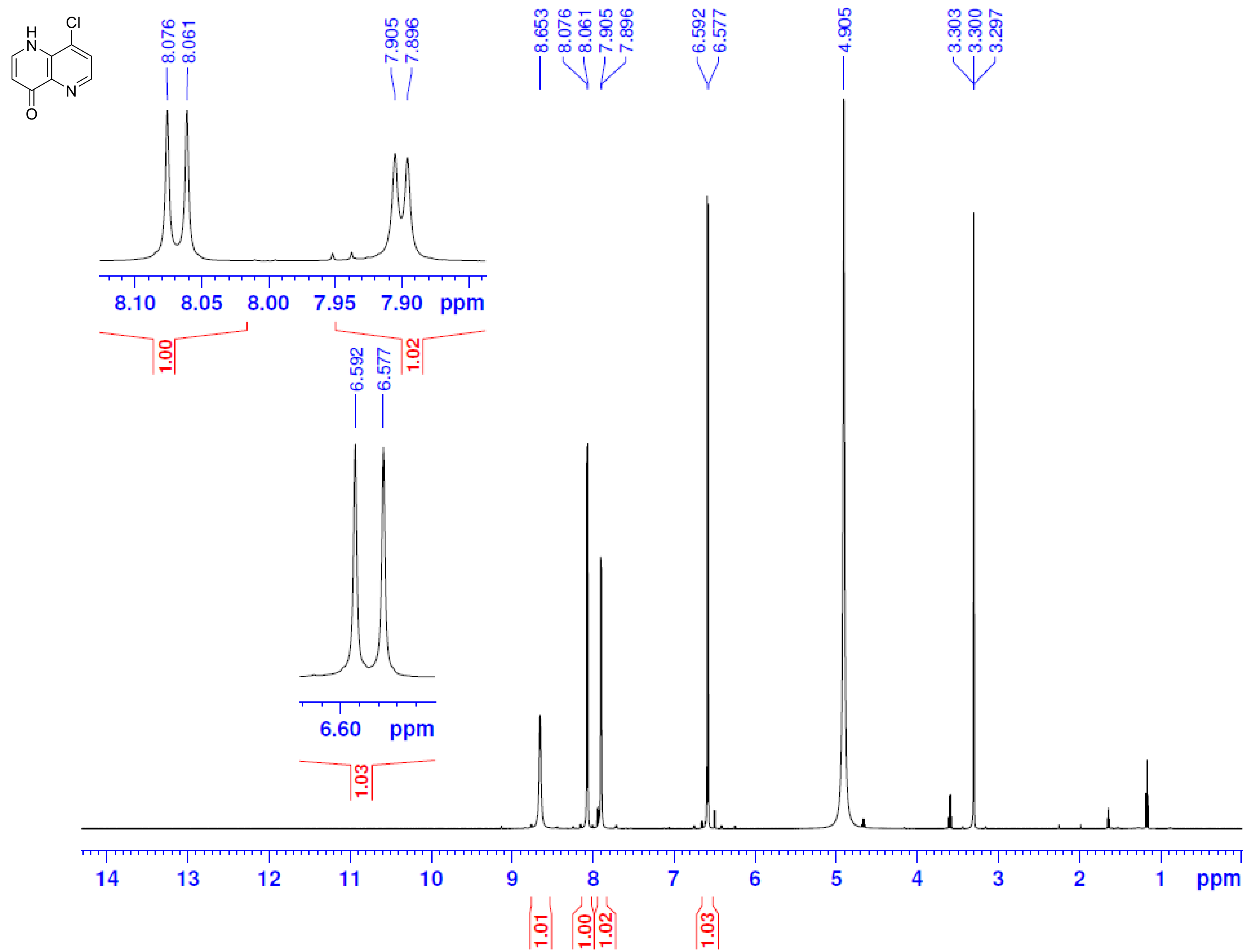

Current Data Parameters
NAME CANTHIN4ONE
EXPNO 1089
PROCNO 1

F2 - Acquisition Parameters
Date_ 20210518
Time 20.45
INSTRUM spect
PROBHD 5 mm PABBO BB-
PULPROG zg30
TD 65536
SOLVENT CD3OD_SPE
NS 16
DS 2
SWH 10000.000 Hz
FIDRES 0.152588 Hz
AQ 3.2767999 sec
RG 114
DW 50.000 usec
DE 6.50 usec
TE 294.8 K
D1 1.00000000 sec
TD0 1

======== CHANNEL f1 ========
SFO1 500.0361158 MHz
NUC1 1H
P1 12.00 usec
PLW1 14.50000000 W

F2 - Processing parameters
SI 65536
SF 500.0330427 MHz
WDW EM
SSB 0
LB 0.30 Hz
GB 0
PC 1.00

5-{[(4-Chloropyrid-3-yl)amino]methylene}-2,2-dimethyl-1,3-dioxane-4,6-dione (**S2**), ^{13}C NMR in MeOH-d_4, 125 MHz

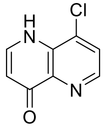

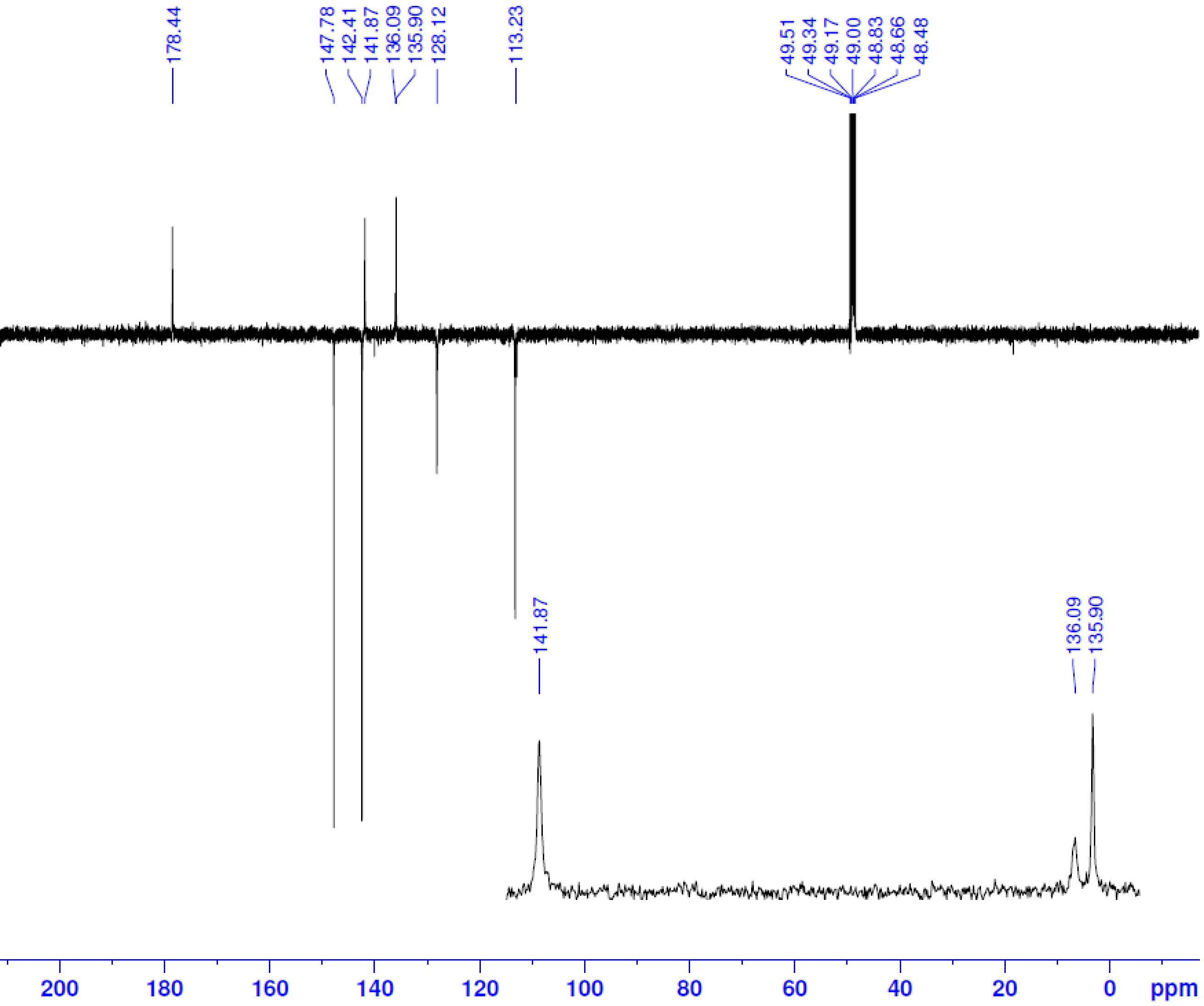

Current Data Parameters
NAME CANTHIN4ONE
EXPNO 1090
PROCNO 1

F2 - Acquisition Parameters
Date_ 20210518
Time 21.32
INSTRUM spect
PROBHD 5 mm PABBO BB-
PULPROG jmod
TD 65536
SOLVENT CD3OD_SPE
NS 2966
DS 4
SWH 29761.904 Hz
FIDRES 0.454131 Hz
AQ 1.1010048 sec
RG 1820
DW 16.800 usec
DE 6.50 usec
TE 296.3 K
CNST2 145.0000000
CNST11 1.0000000
D1 2.00000000 sec
D20 0.00689655 sec
TD0 1

======== CHANNEL f1 ======
SFO1 125.7459782 MHz
NUC1 13C
P1 9.20 usec
P2 18.40 usec
PLW1 140.00000000 W

======== CHANNEL f2 ======
SFO2 500.0350280 MHz
NUC2 1H
CPDPRG[2 waltz16
PCPD2 80.00 usec
PLW2 14.50000000 W
PLW12 0.32624999 W

F2 - Processing parameters
SI 32768
SF 125.7332297 MHz
WDW EM
SSB 0
LB 1.00 Hz
GB 0
PC 1.40

8'-Chloro-4*H*-[1,4'-bi(1,5-naphthyridine)]-4,8(5*H*)-dione (**S4**), ^{1}H NMR in DMSO-d_6, 500 MHz

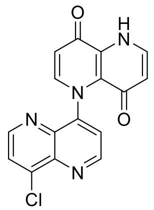

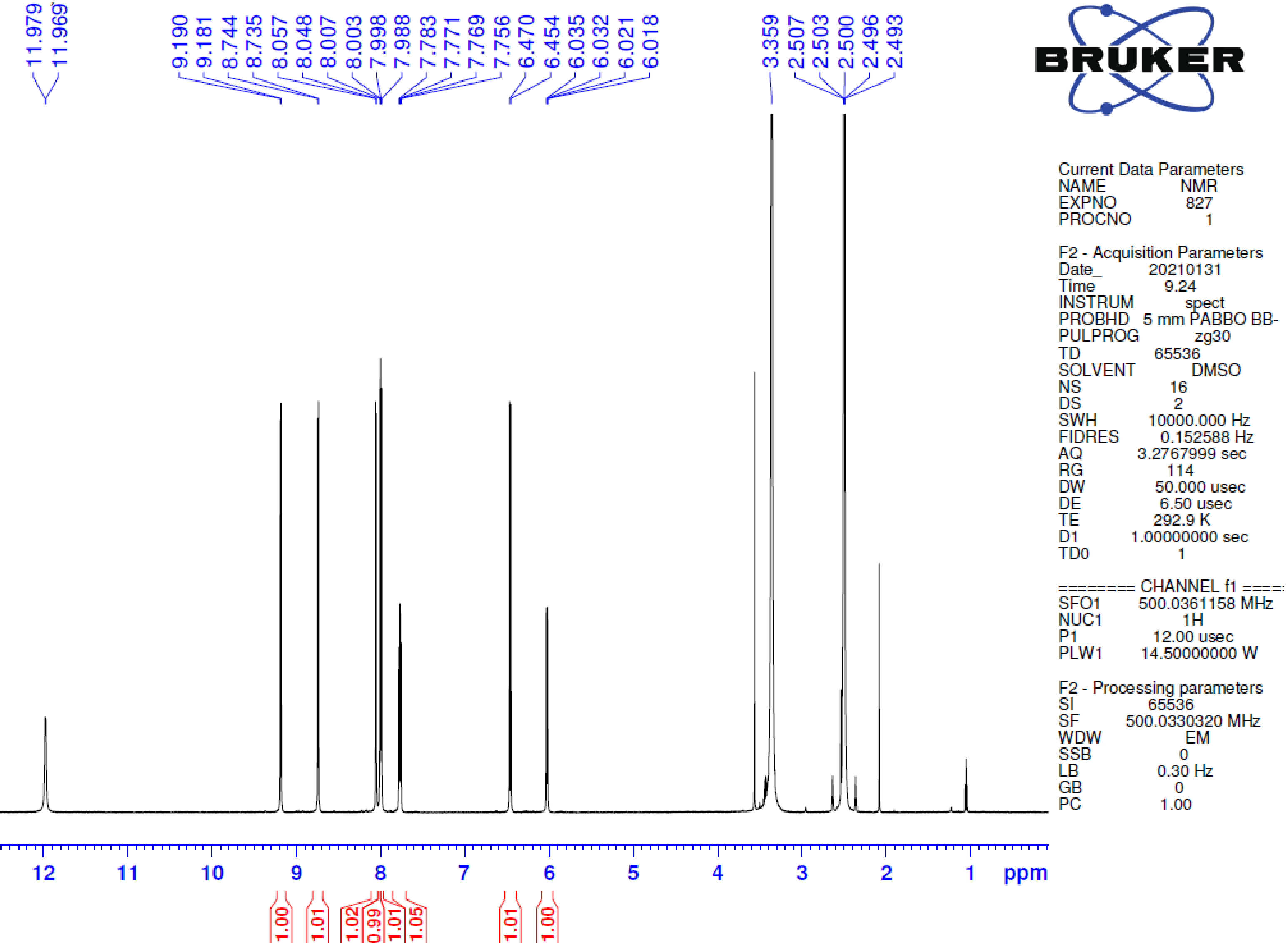

8'-Chloro-4*H*-[1,4'-bi(1,5-naphthyridine)]-4,8(5*H*)-dione (**S4**), ^{13}C NMR in DMSO-d_6, 75 MHz

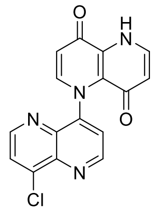

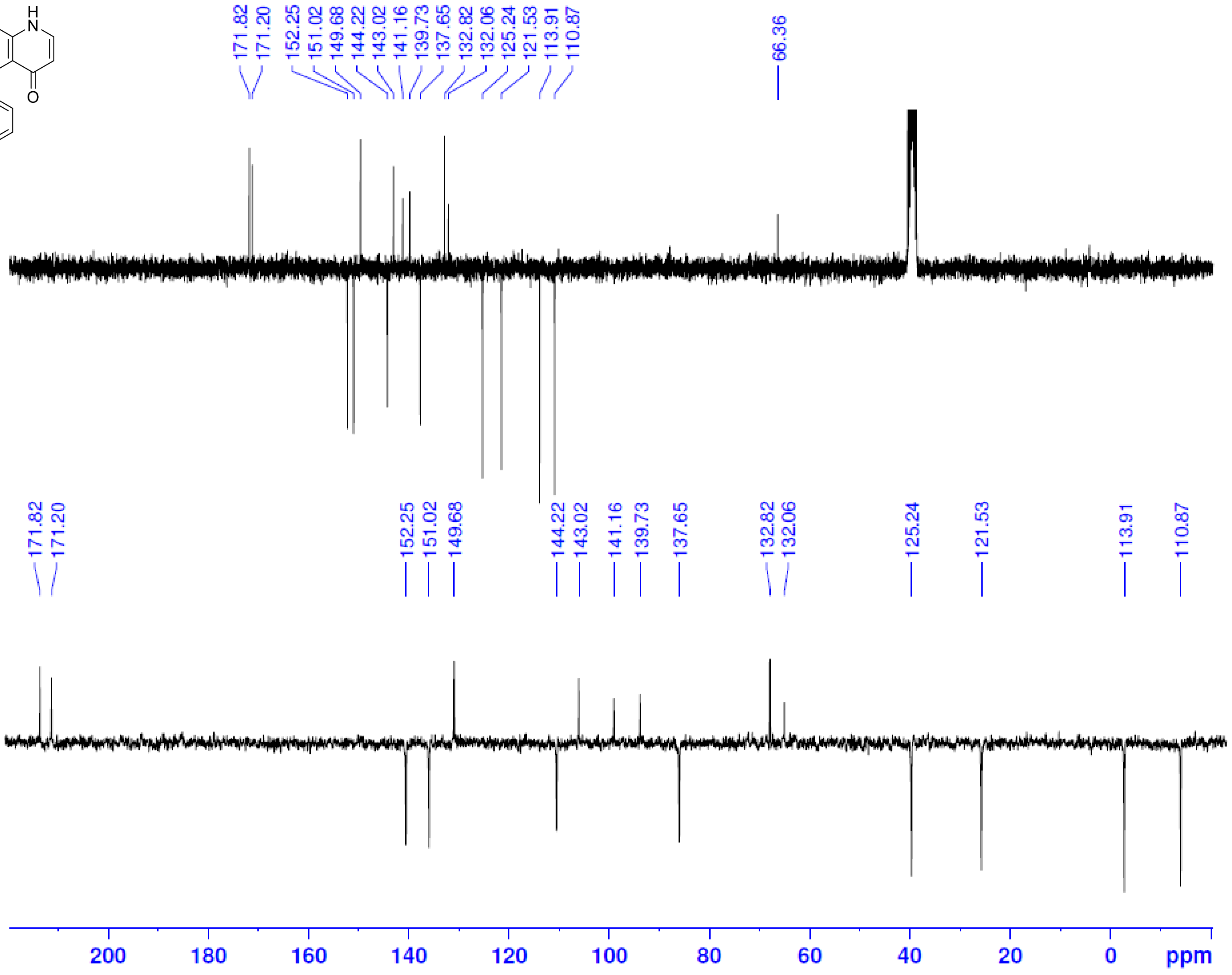

Current Data Parameters
NAME MARIA
EXPNO 242
PROCNO 1

F2 - Acquisition Parameters
Date_ 20210115
Time 15.01 h
INSTRUM spect
PROBHD Z104275_0375 (
PULPROG jmod
TD 65536
SOLVENT DMSO
NS 16384
DS 4
SWH 18115.941 Hz
FIDRES 0.552855 Hz
AQ 1.8087935 sec
RG 201.81
DW 27.600 usec
DE 6.50 usec
TE 294.6 K
CNST2 145.0000000
CNST11 1.0000000
D1 2.00000000 sec
D20 0.00689655 sec
TD0 1
SFO1 75.4752953 MHz
NUC1 13C
P1 10.00 usec
P2 20.00 usec
PLW1 41.00000000 W
SFO2 300.1312005 MHz
NUC2 1H
CPDPRG[2 waltz16
PCPD2 80.00 usec
PLW2 8.19999981 W
PLW12 0.25112000 W

F2 - Processing parameters
SI 32768
SF 75.4677831 MHz
WDW EM
SSB 0
LB 1.00 Hz
GB 0
PC 1.40

5-{[(3-Bromopyrid-4-yl)amino]methylene}-2,2-dimethyl-1,3-dioxane-4,6-dione (**S10**), ^{1}H NMR in $CDCl_3$, 300 MHz

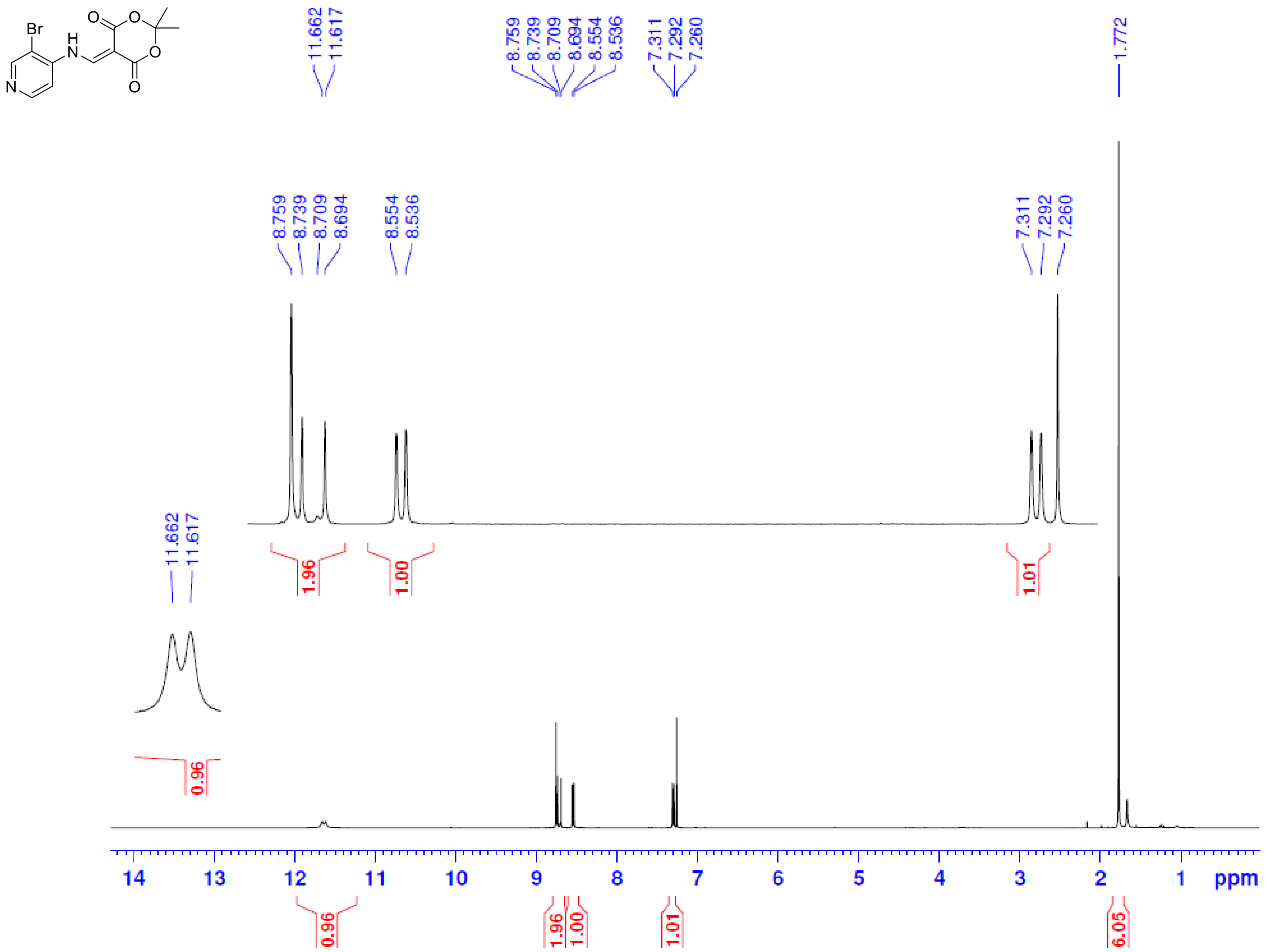

Current Data Parameters
NAME MARIA
EXPNO 276
PROCNO 1

F2 - Acquisition Parameters
Date_ 20210214
Time 18.49 h
INSTRUM spect
PROBHD Z104275_0375 (
PULPROG zg30
TD 65536
SOLVENT CDCl3
NS 16
DS 2
SWH 6009.615 Hz
FIDRES 0.183399 Hz
AQ 5.4525952 sec
RG 201.81
DW 83.200 usec
DE 6.50 usec
TE 293.7 K
D1 1.00000000 sec
TD0 1
SFO1 300.1318533 MHz
NUC1 1H
P1 14.00 usec
PLW1 8.19999981 W

F2 - Processing parameters
SI 65536
SF 300.1300073 MHz
WDW EM
SSB 0
LB 0.30 Hz
GB 0
PC 1.00

5-{[(3-Bromopyrid-4-yl)amino]methylene}-2,2-dimethyl-1,3-dioxane-4,6-dione (**S10**), ^{13}C NMR in $CDCl_3$, 125 MHz

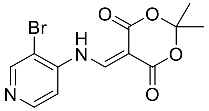

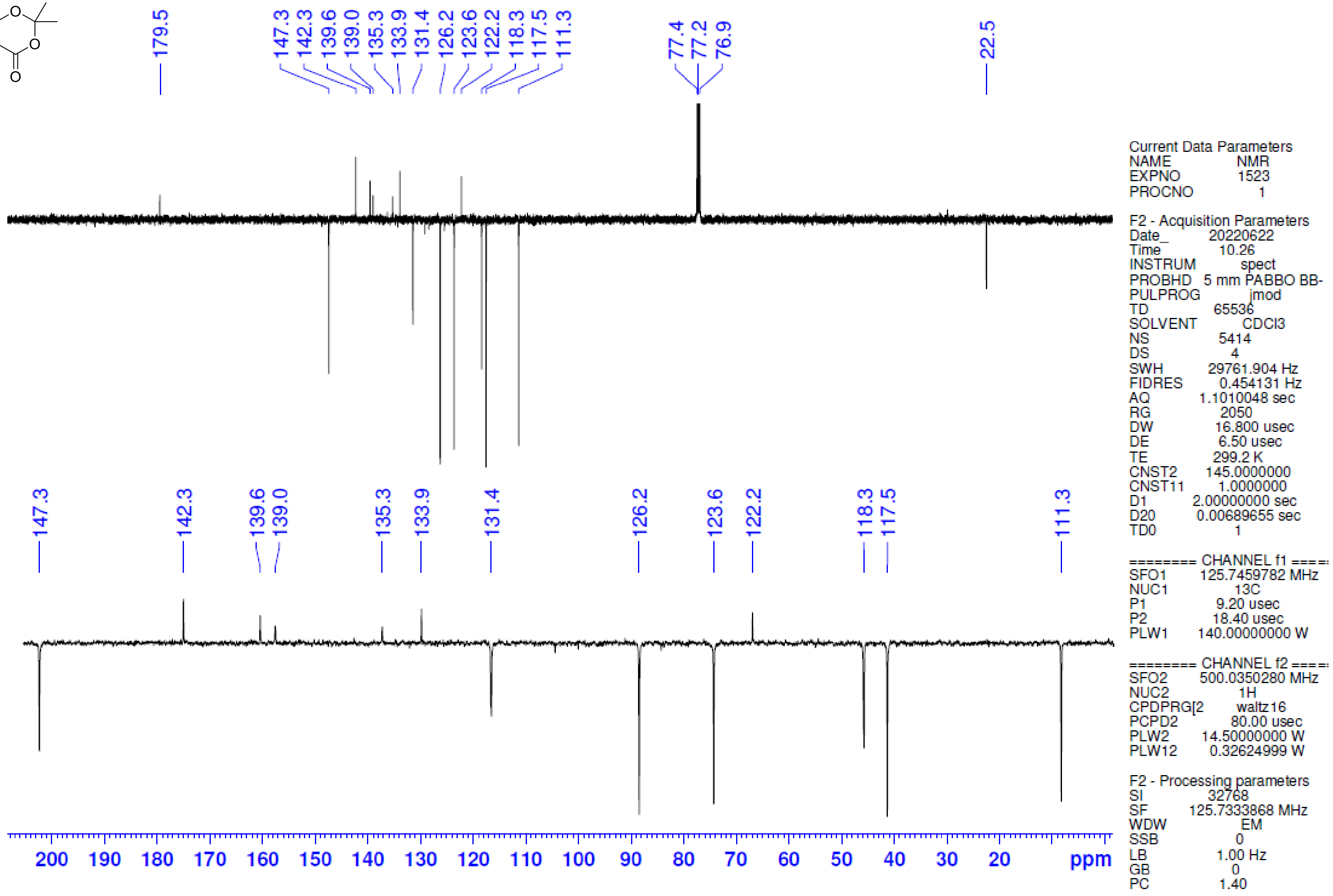

5-{[(4-Bromopyrid-3-yl)amino]methylene}-2,2-dimethyl-1,3-dioxane-4,6-dione (**1**), ^{1}H NMR in $CDCl_3$, 500 MHz

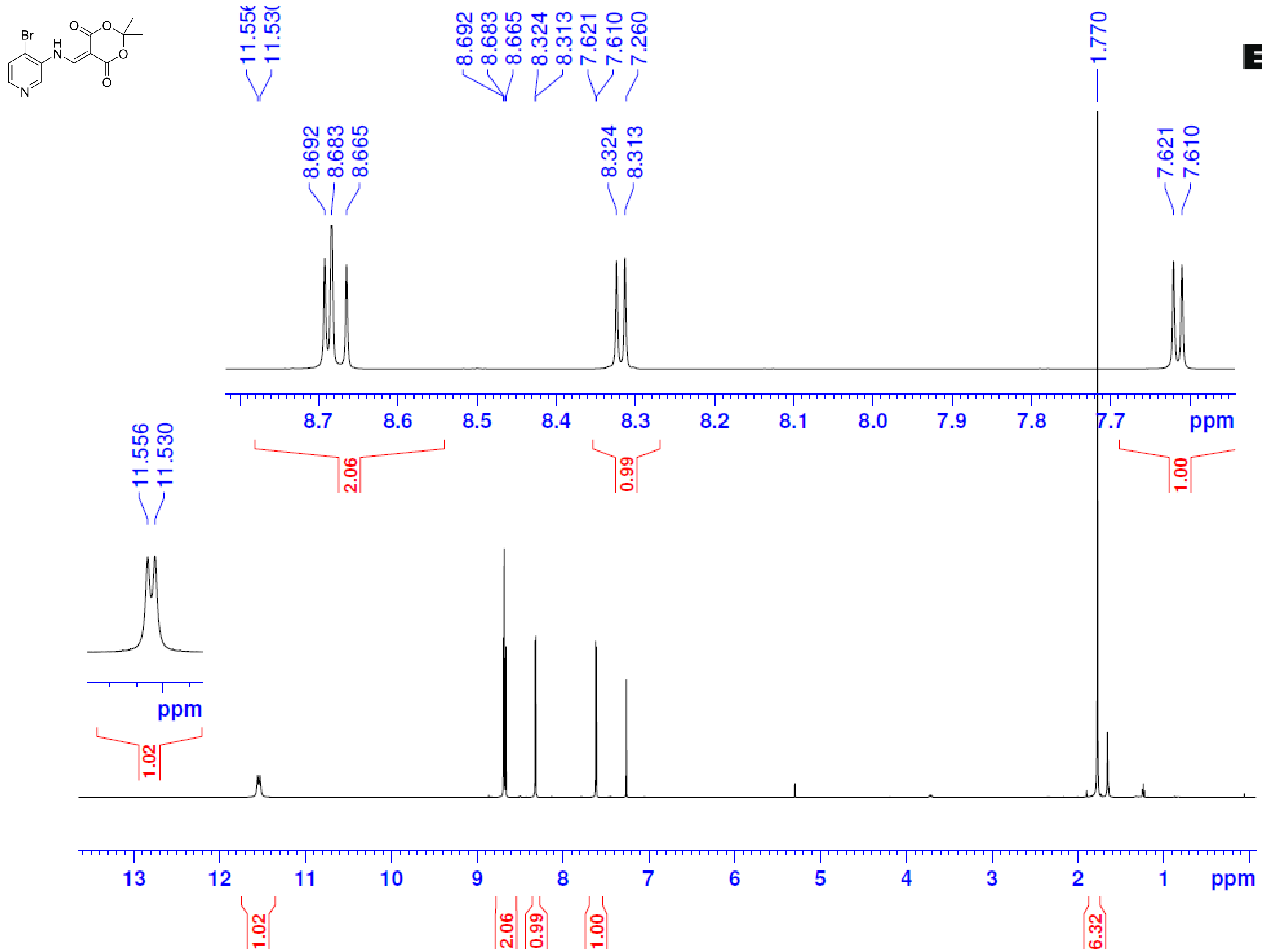

Current Data Parameters
NAME NMR
EXPNO 122
PROCNO 1

F2 - Acquisition Parameters
Date_ 20200130
Time 10.14
INSTRUM spect
PROBHD 5 mm PABBO BB-
PULPROG zg30
TD 65536
SOLVENT CDCl3
NS 16
DS 2
SWH 10000.000 Hz
FIDRES 0.152588 Hz
AQ 3.2767999 sec
RG 144
DW 50.000 usec
DE 6.50 usec
TE 295.0 K
D1 1.00000000 sec
TD0 1

======== CHANNEL f1 =====
SFO1 500.0361158 MHz
NUC1 1H
P1 12.00 usec
PLW1 14.50000000 W

F2 - Processing parameters
SI 65536
SF 500.0330409 MHz
WDW EM
SSB 0
LB 0.30 Hz
GB 0
PC 1.00

5-{[(4-Bromopyrid-3-yl)amino]methylene}-2,2-dimethyl-1,3-dioxane-4,6-dione (**1**), ^{13}C NMR in $CDCl_3$, 125 MHz

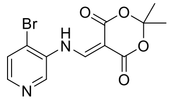

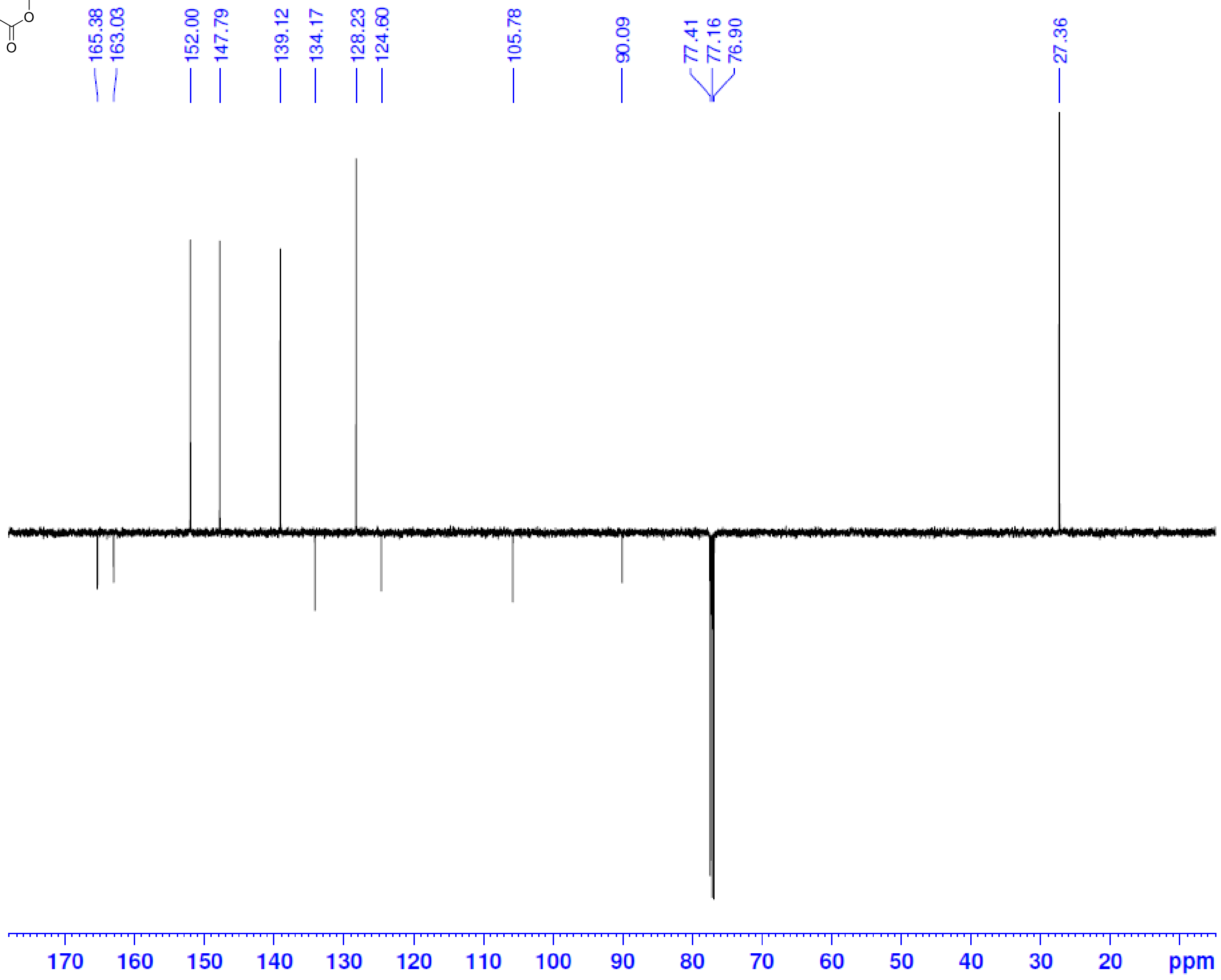

Current Data Parameters
NAME CANTHIN4ONE
EXPNO 123
PROCNO 1

F2 - Acquisition Parameters
Date_ 20200130
Time 11.15
INSTRUM spect
PROBHD 5 mm PABBO BB-
PULPROG jmod
TD 65536
SOLVENT CDCl3
NS 1001
DS 4
SWH 29761.904 Hz
FIDRES 0.454131 Hz
AQ 1.1010048 sec
RG 2050
DW 16.800 usec
DE 6.50 usec
TE 296.4 K
CNST2 145.0000000
CNST11 1.0000000
D1 2.00000000 sec
D20 0.00689655 sec
TD0 1

======== CHANNEL f1 ========
SFO1 125.7459782 MHz
NUC1 13C
P1 9.20 usec
P2 18.40 usec
PLW1 140.00000000 W

======== CHANNEL f2 ========
SFO2 500.0350280 MHz
NUC2 1H
CPDPRG[2 waltz16
PCPD2 80.00 usec
PLW2 14.50000000 W
PLW12 0.32624999 W

F2 - Processing parameters
SI 32768
SF 125.7333902 MHz
WDW EM
SSB 0
LB 1.00 Hz
GB 0
PC 1.40

8-Bromo-1,5-naphthyridin-4(1*H*)-one (**2**), ^{1}H NMR in MeOH-d_4, 500 MHz

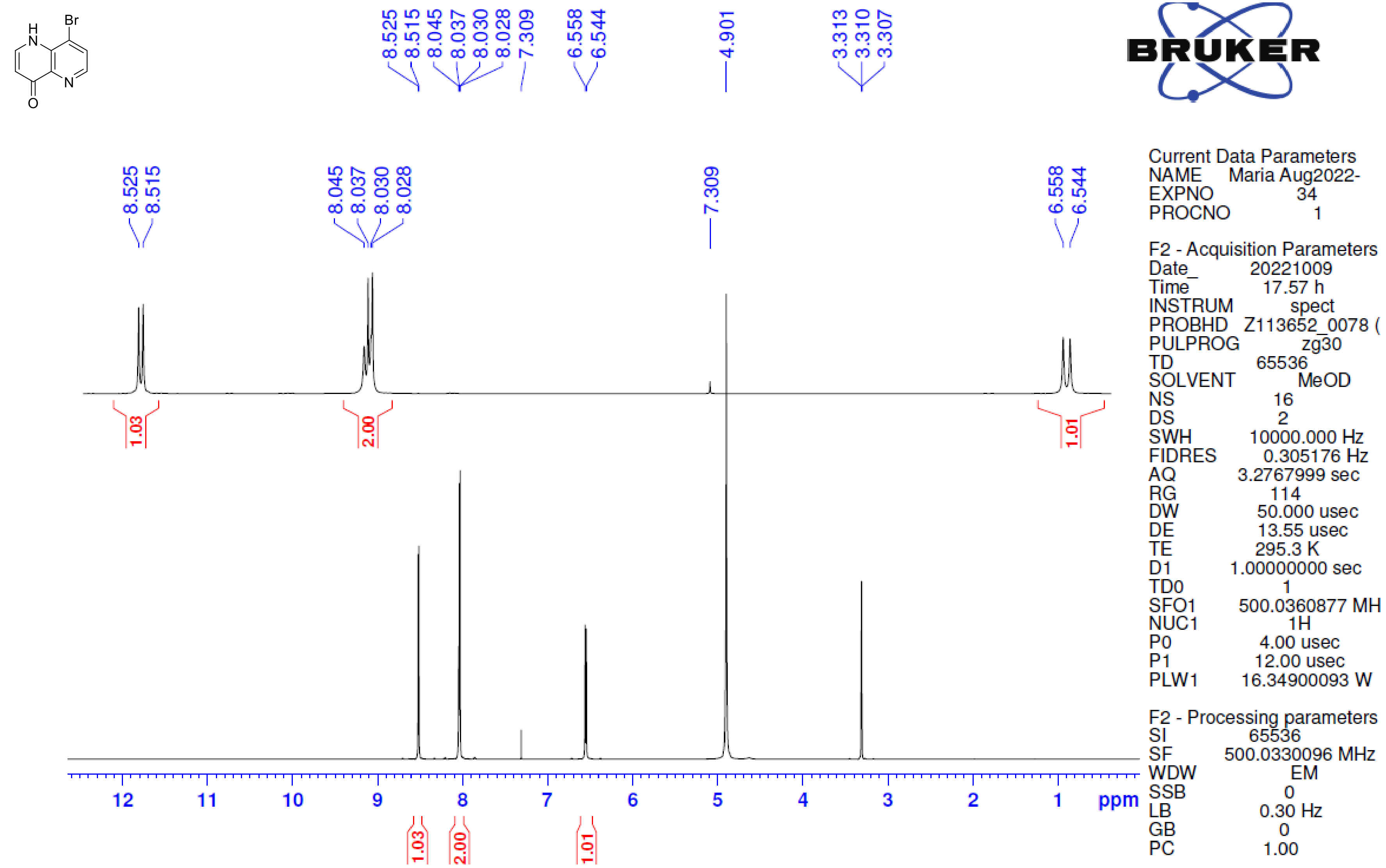

8-Bromo-1,5-naphthyridin-4(1*H*)-one (**2**), ^{13}C NMR in MeOH-d_4, 125 MHz

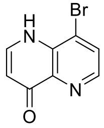

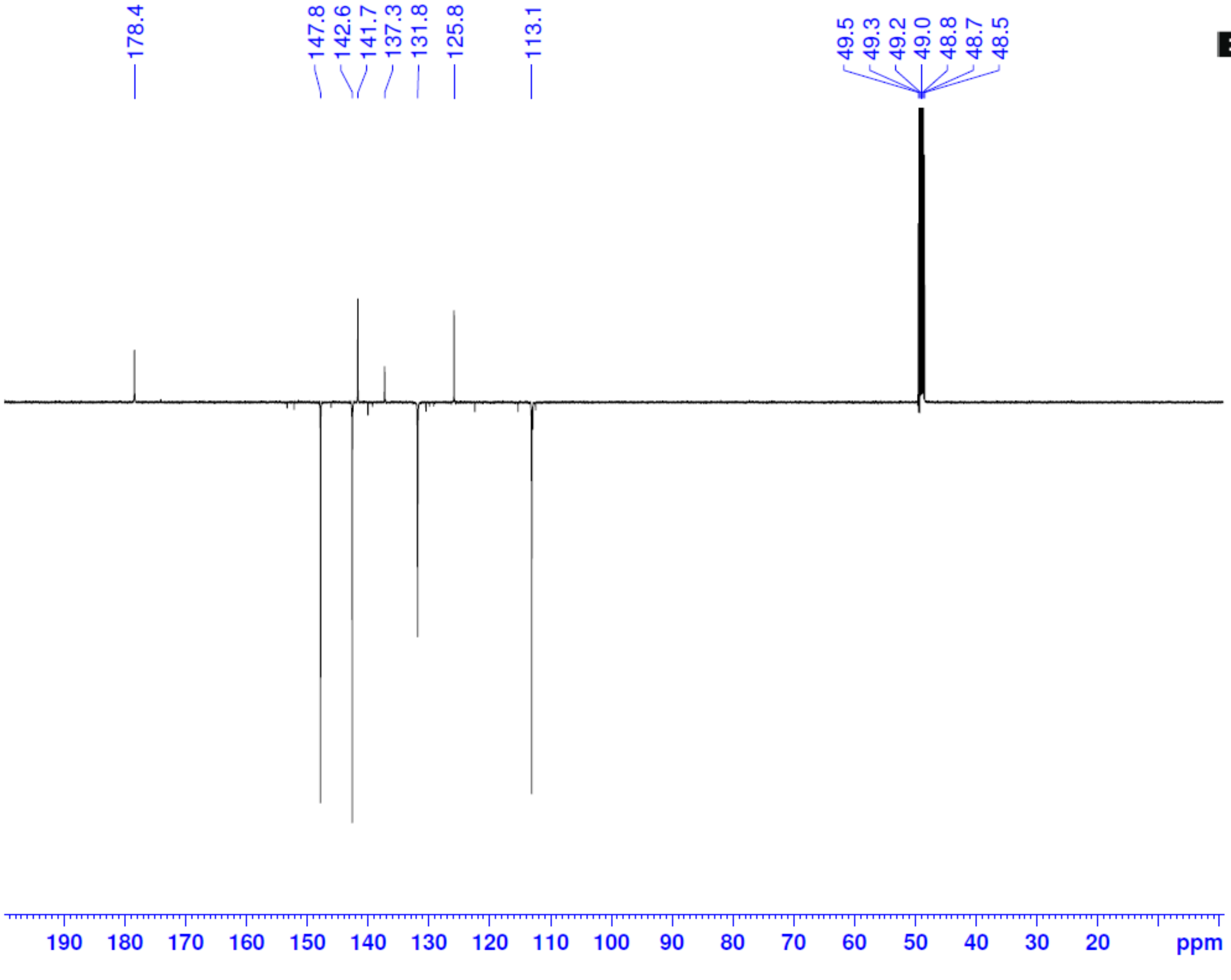

Current Data Parameters
NAME Maria Aug2022-
EXPNO 144
PROCNO 1

F2 - Acquisition Parameters
Date_ 20230917
Time 13.03 h
INSTRUM spect
PROBHD Z113652_0078 (
PULPROG jmod
TD 65536
SOLVENT CD3OD_SPE
NS 22402
DS 4
SWH 29761.904 Hz
FIDRES 0.908261 Hz
AQ 1.1010048 sec
RG 2050
DW 16.800 usec
DE 6.50 usec
TE 298.8 K
CNST2 145.0000000
CNST11 1.0000000
D1 2.00000000 sec
D20 0.00689655 sec
TD0 1
SFO1 125.7459712 MHz
NUC1 13C
P1 10.00 usec
P2 20.00 usec
PLW1 121.36000061 W
SFO2 500.0350001 MHz
NUC2 1H
CPDPRG[2 waltz65
PCPD2 80.00 usec
PLW2 16.34900093 W
PLW12 0.34647381 W

F2 - Processing parameters
SI 32768
SF 125.7332243 MHz
WDW EM
SSB 0
LB 1.00 Hz
GB 0
PC 1.40

8'-Bromo-4*H*-[1,4'-bi(1,5-naphthyridine)]-4,8(5*H*)-dione (**3**), ^{1}H NMR in DMSO-d_6, 500 MHz

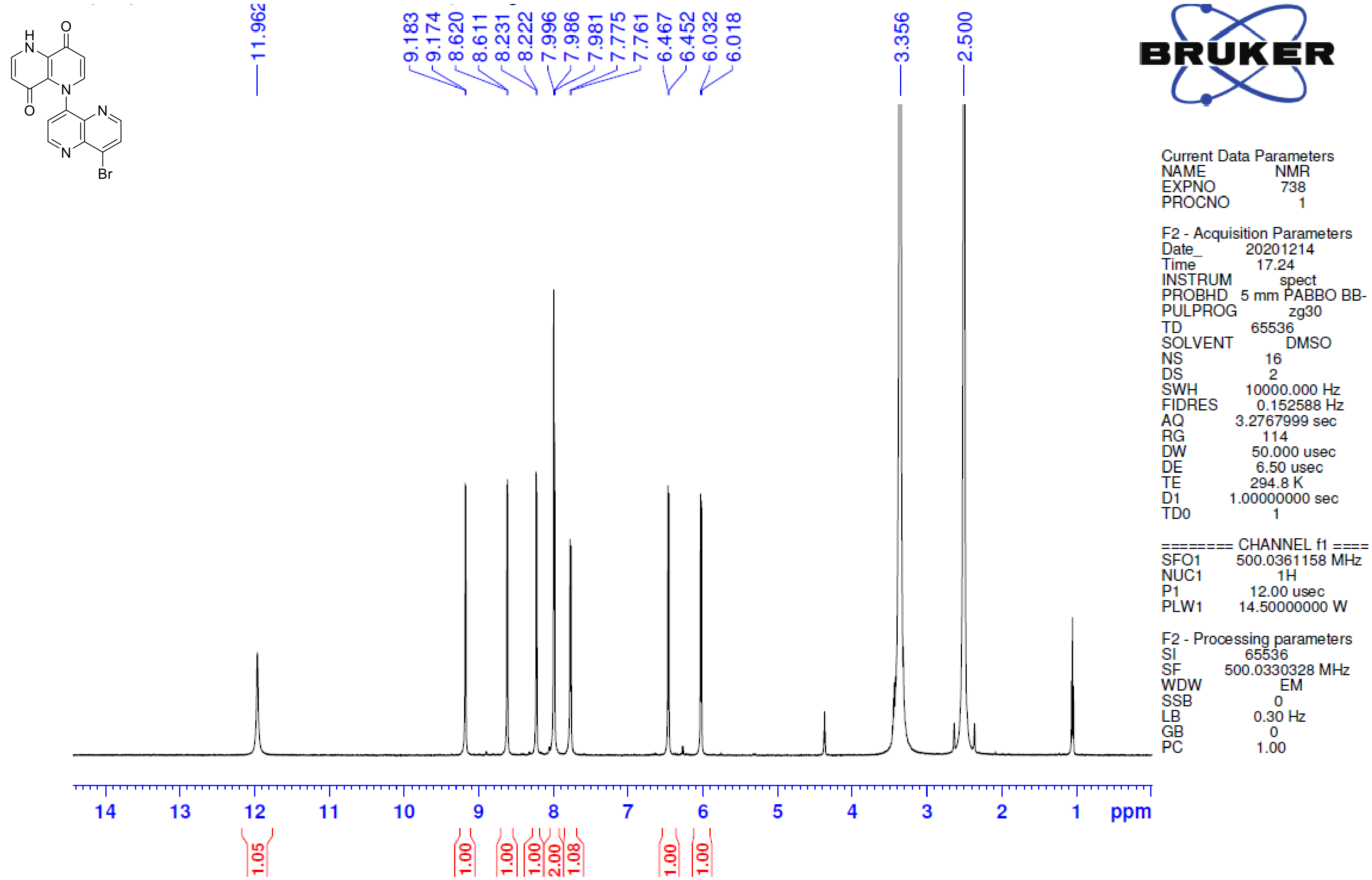

Current Data Parameters
NAME NMR
EXPNO 738
PROCNO 1

F2 - Acquisition Parameters
Date_ 20201214
Time 17.24
INSTRUM spect
PROBHD 5 mm PABBO BB-
PULPROG zg30
TD 65536
SOLVENT DMSO
NS 16
DS 2
SWH 10000.000 Hz
FIDRES 0.152588 Hz
AQ 3.2767999 sec
RG 114
DW 50.000 usec
DE 6.50 usec
TE 294.8 K
D1 1.00000000 sec
TD0 1

======== CHANNEL f1 ====
SFO1 500.0361158 MHz
NUC1 1H
P1 12.00 usec
PLW1 14.50000000 W

F2 - Processing parameters
SI 65536
SF 500.0330328 MHz
WDW EM
SSB 0
LB 0.30 Hz
GB 0
PC 1.00

8'-Bromo-4*H*-[1,4'-bi(1,5-naphthyridine)]-4,8(5*H*)-dione (**3**), ^{13}C NMR in DMSO-d_6, 125 MHz

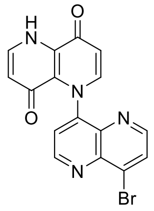

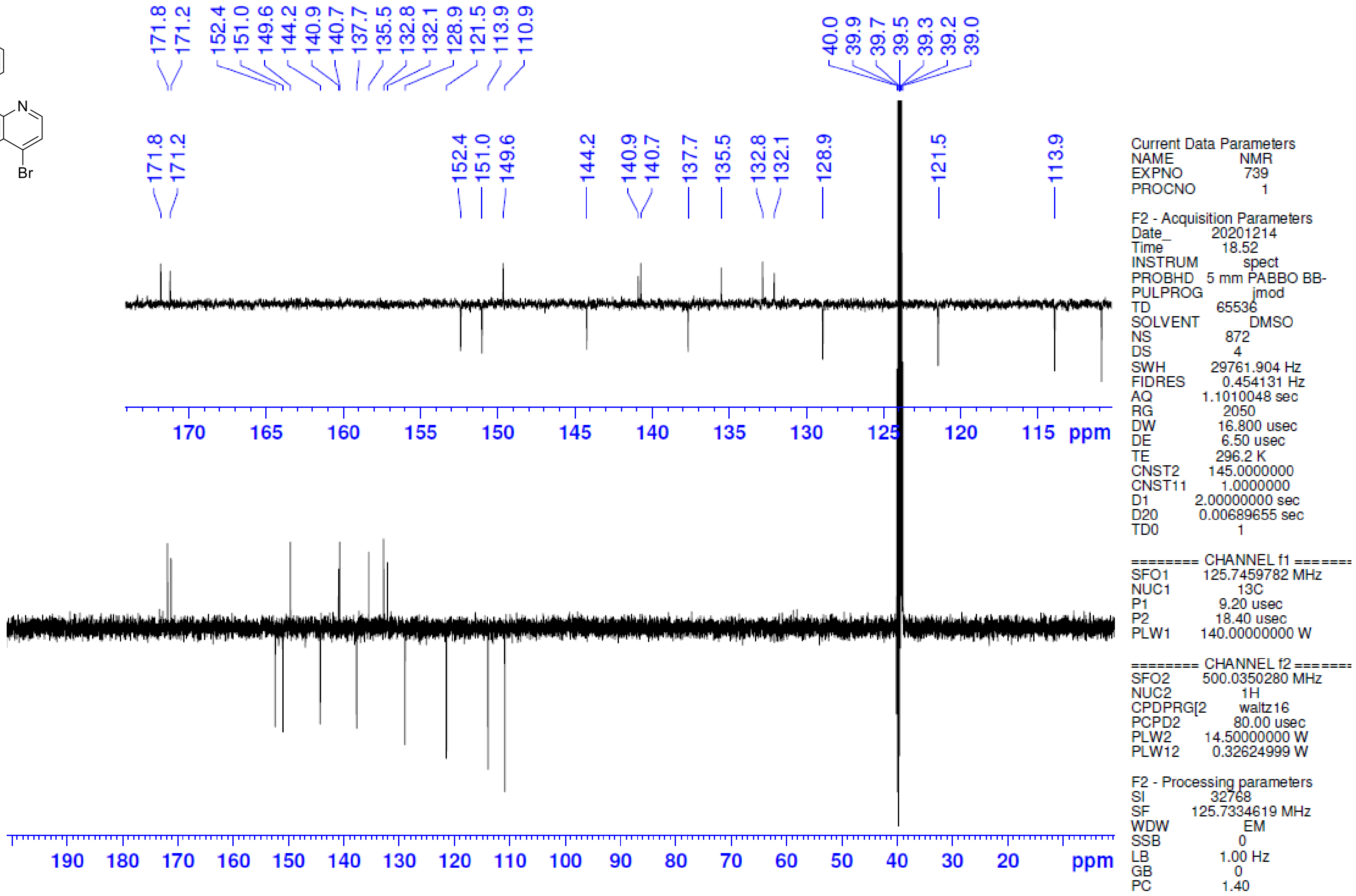

4-Bromo-8-methoxy-1,5-naphthyridine (**5**), ^{1}H NMR in $CDCl_3$, 500 MHz

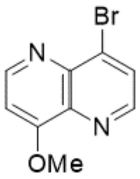

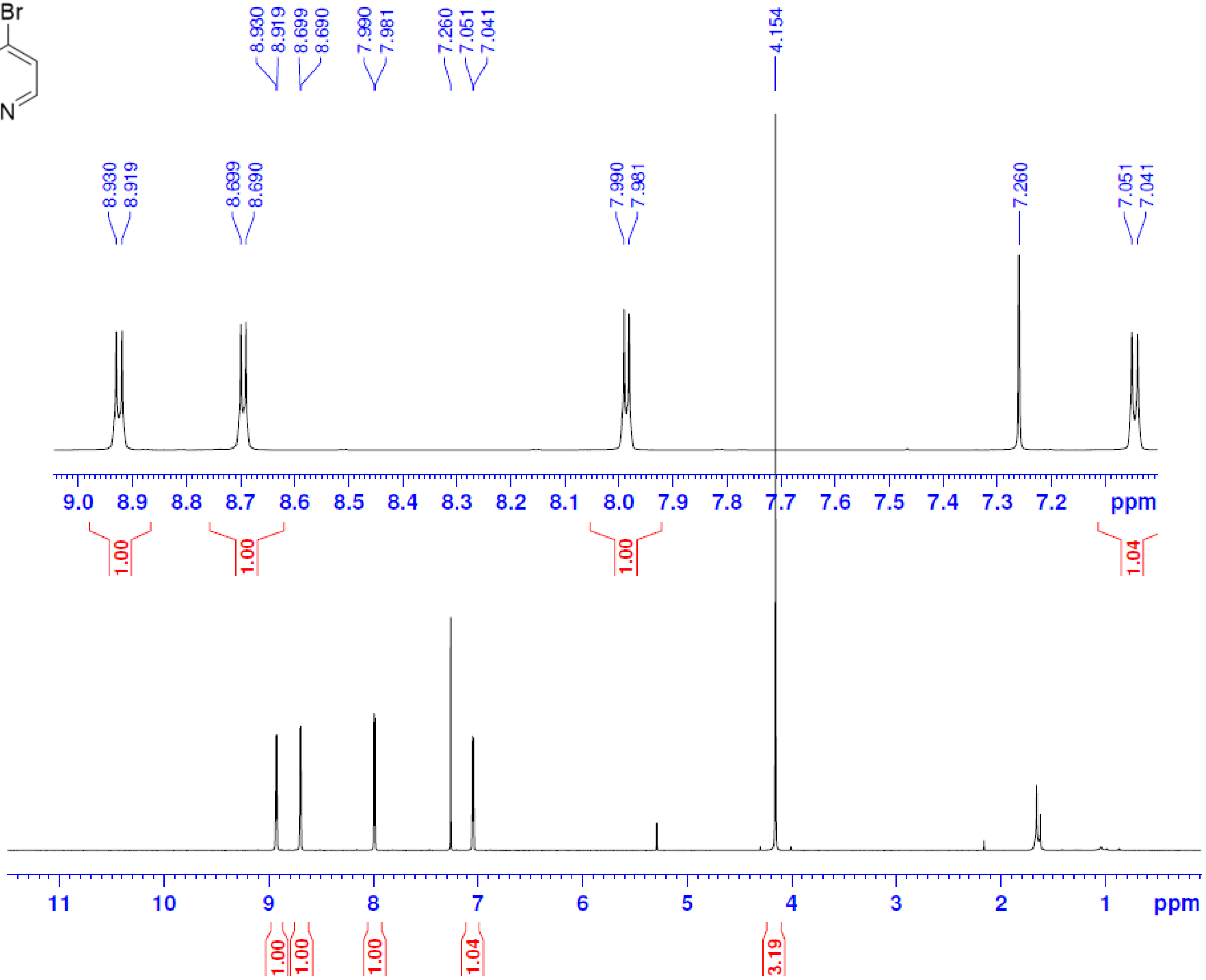

Current Data Parameters
NAME CANTHIN4ONE
EXPNO 910
PROCNO 1

F2 - Acquisition Parameters
Date_ 20210303
Time 11.27
INSTRUM spect
PROBHD 5 mm PABBO BB-
PULPROG zg30
TD 65536
SOLVENT CDCl3
NS 16
DS 2
SWH 10000.000 Hz
FIDRES 0.152588 Hz
AQ 3.2767999 sec
RG 144
DW 50.000 usec
DE 6.50 usec
TE 295.3 K
D1 1.00000000 sec
TD0 1

======== CHANNEL f1 ====
SFO1 500.0361158 MHz
NUC1 1H
P1 12.00 usec
PLW1 14.50000000 W

F2 - Processing parameters
SI 65536
SF 500.0330405 MHz
WDW EM
SSB 0
LB 0.30 Hz
GB 0
PC 1.00

4-Bromo-8-methoxy-1,5-naphthyridine (**5**), ^{13}C NMR in $CDCl_3$, 125 MHz

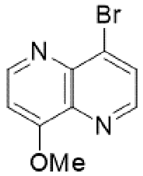

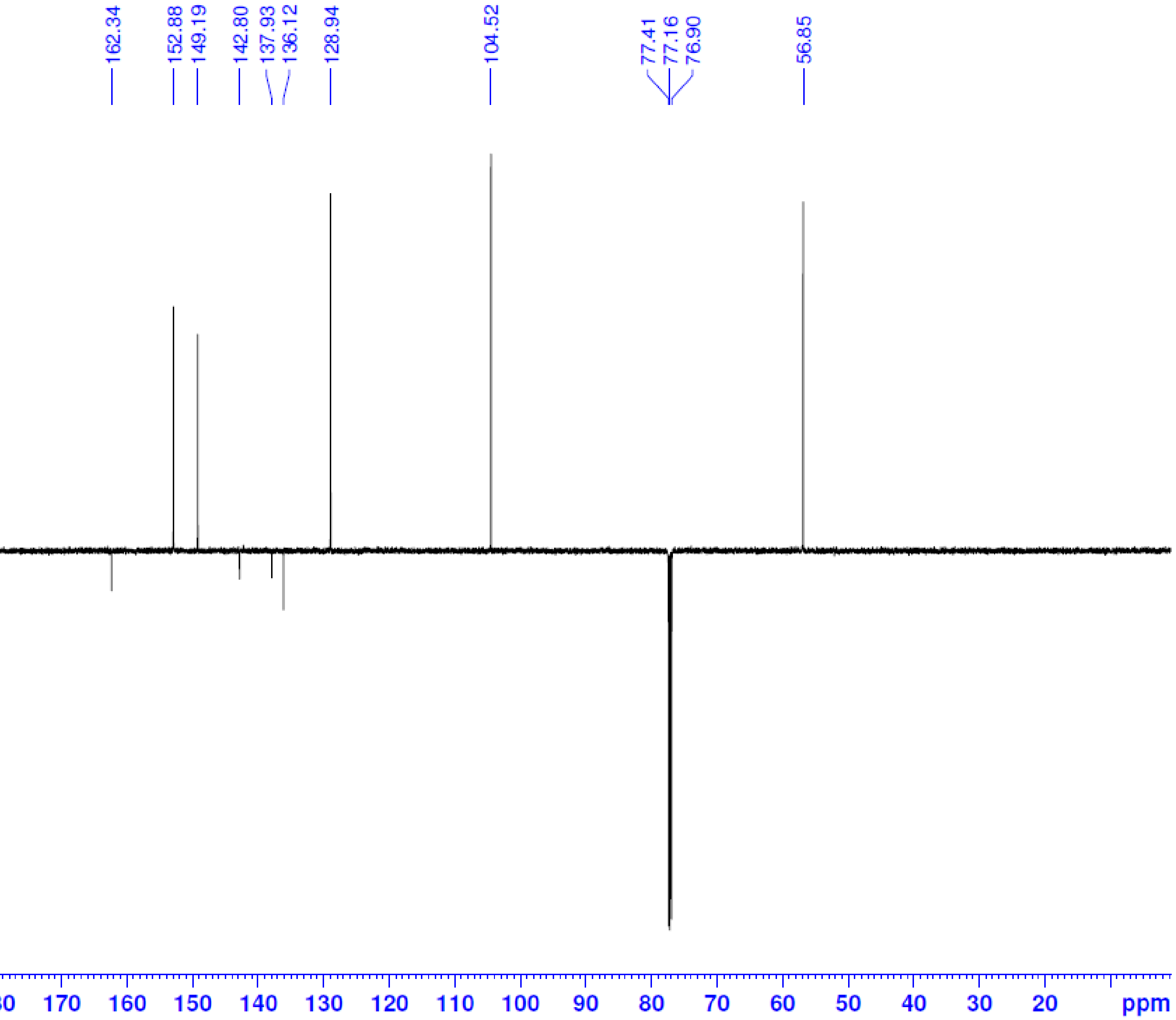

Current Data Parameters
NAME CANTHIN4ONE
EXPNO 918
PROCNO 1

F2 - Acquisition Parameters
Date_ 20210303
Time 18.34
INSTRUM spect
PROBHD 5 mm PABBO BB-
PULPROG jmod
TD 65536
SOLVENT CDCl3
NS 2560
DS 4
SWH 29761.904 Hz
FIDRES 0.454131 Hz
AQ 1.1010048 sec
RG 2050
DW 16.800 usec
DE 6.50 usec
TE 296.5 K
CNST2 145.0000000
CNST11 1.0000000
D1 2.00000000 sec
D20 0.00689655 sec
TD0 1

======== CHANNEL f1 =====
SFO1 125.7459782 MHz
NUC1 13C
P1 9.20 usec
P2 18.40 usec
PLW1 140.00000000 W

======== CHANNEL f2 =====
SFO2 500.0350280 MHz
NUC2 1H
CPDPRG[2 waltz16
PCPD2 80.00 usec
PLW2 14.50000000 W
PLW12 0.32624999 W

F2 - Processing parameters
SI 32768
SF 125.7333898 MHz
WDW EM
SSB 0
LB 1.00 Hz
GB 0
PC 1.40

4-(2-Chlorophenyl)-8-methoxy-1,5-naphthyridine (**6**), ^{1}H NMR in $CDCl_3$, 500 MHz

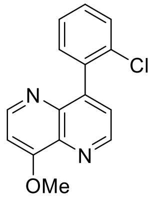

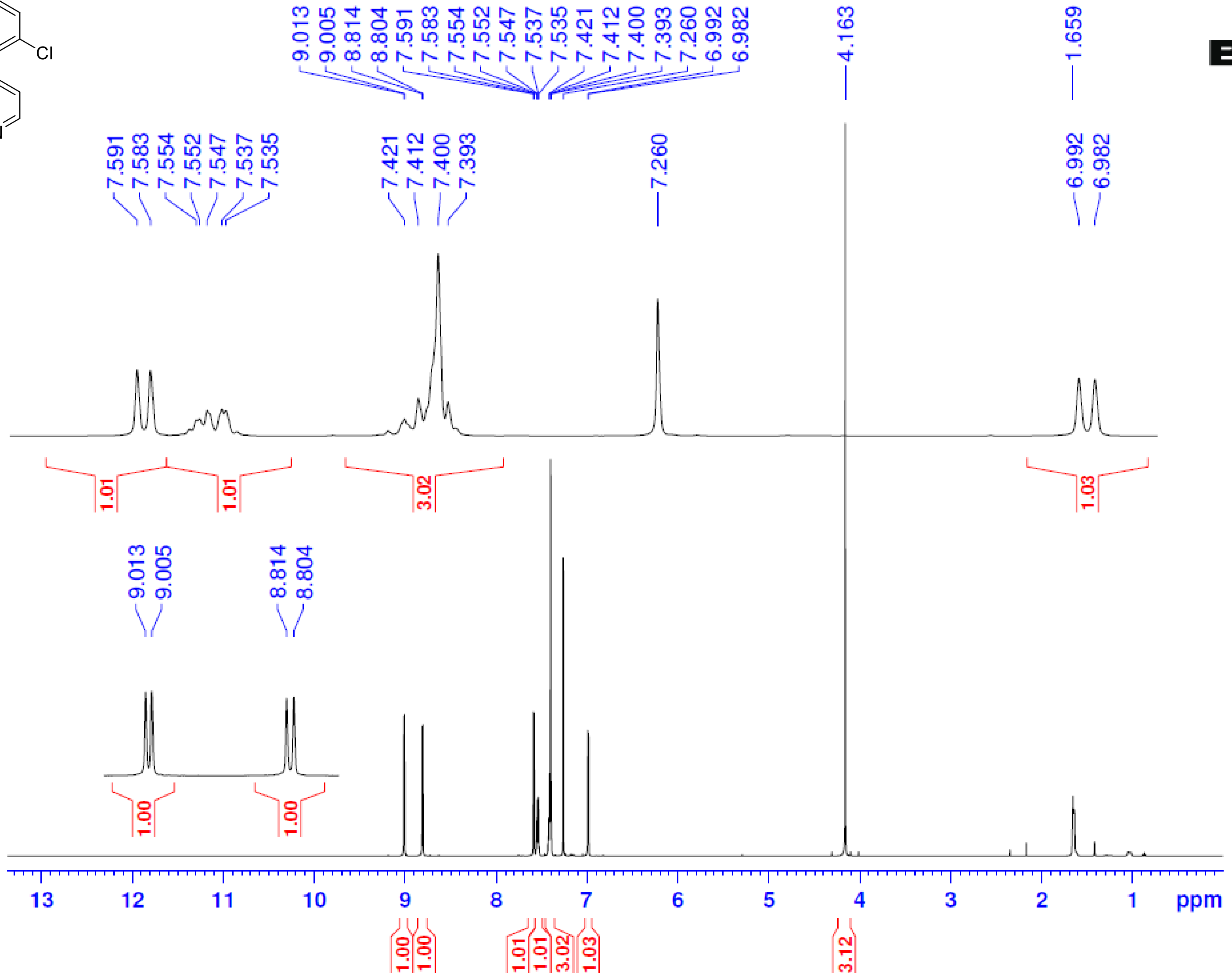

Current Data Parameters
NAME Maria
EXPNO 1008
PROCNO 1

F2 - Acquisition Parameters
Date_ 20210328
Time 18.23
INSTRUM spect
PROBHD 5 mm PABBO BB-
PULPROG zg30
TD 65536
SOLVENT CDCl3
NS 16
DS 2
SWH 10000.000 Hz
FIDRES 0.152588 Hz
AQ 3.2767999 sec
RG 144
DW 50.000 usec
DE 6.50 usec
TE 293.5 K
D1 1.00000000 sec
TD0 1

======= CHANNEL f1 ====
SFO1 500.0361158 MHz
NUC1 1H
P1 12.00 usec
PLW1 14.50000000 W

F2 - Processing parameters
SI 65536
SF 500.0330406 MHz
WDW EM
SSB 0
LB 0.30 Hz
GB 0
PC 1.00

4-(2-Chlorophenyl)-8-methoxy-1,5-naphthyridine (**6**), ^{13}C NMR in $CDCl_3$, 125 MHz

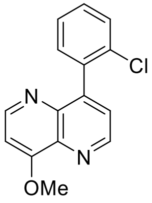

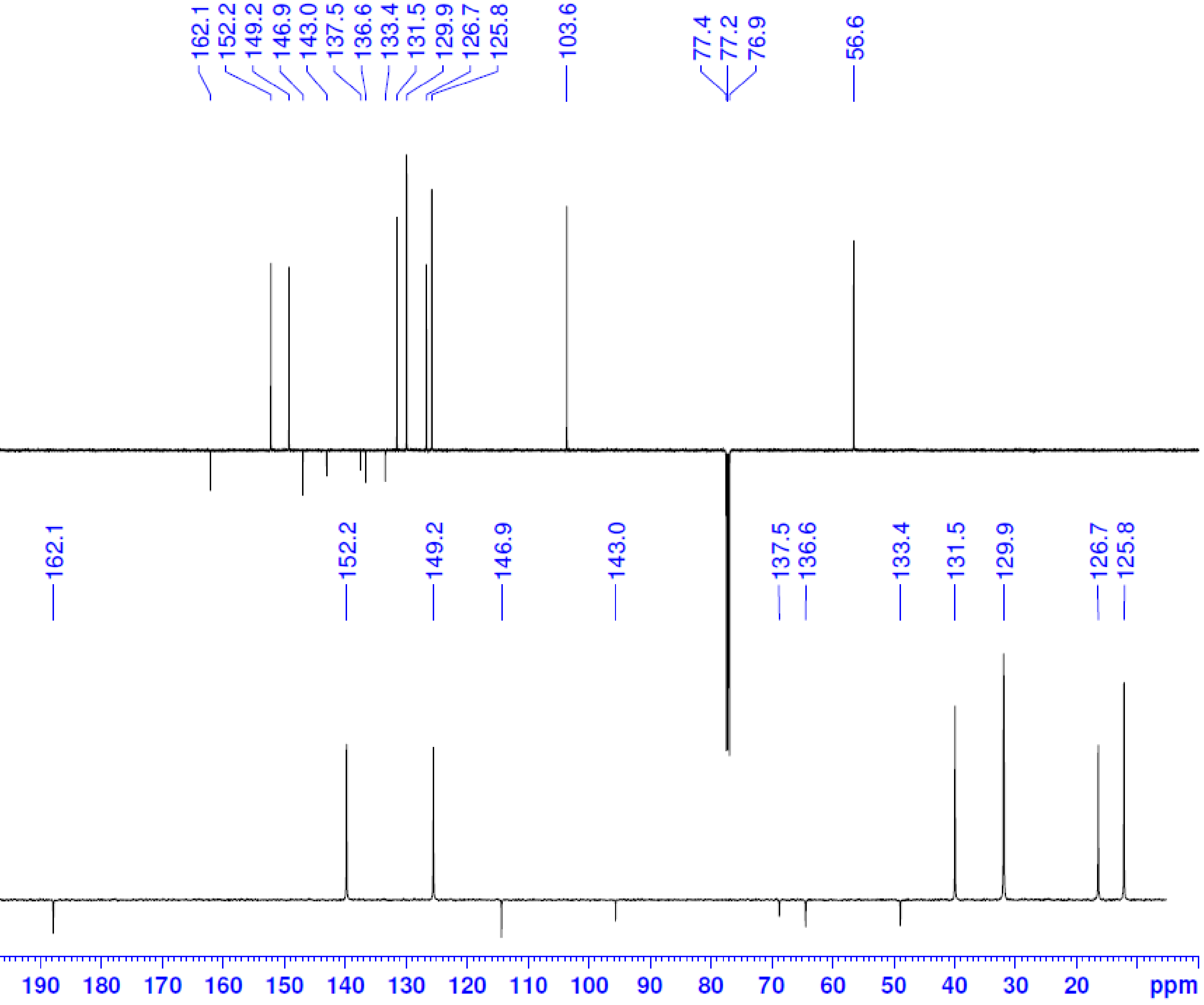

Current Data Parameters
NAME Maria
EXPNO 1009
PROCNO 1

F2 - Acquisition Parameters
Date_ 20210328
Time 23.46
INSTRUM spect
PROBHD 5 mm PABBO BB-
PULPROG jmod
TD 65536
SOLVENT CDCl3
NS 6096
DS 4
SWH 29761.904 Hz
FIDRES 0.454131 Hz
AQ 1.1010048 sec
RG 2050
DW 16.800 usec
DE 6.50 usec
TE 294.1 K
CNST2 145.0000000
CNST11 1.0000000
D1 2.00000000 sec
D20 0.00689655 sec
TD0 1

======== CHANNEL f1 ======
SFO1 125.7459782 MHz
NUC1 13C
P1 9.20 usec
P2 18.40 usec
PLW1 140.00000000 W

======== CHANNEL f2 ======
SFO2 500.0350280 MHz
NUC2 1H
CPDPRG[2 waltz16
PCPD2 80.00 usec
PLW2 14.50000000 W
PLW12 0.32624999 W

F2 - Processing parameters
SI 32768
SF 125.7333906 MHz
WDW EM
SSB 0
LB 1.00 Hz
GB 0
PC 1.40

8-(2-Chlorophenyl)-1,5-naphthyridin-4(1*H*)-one (**7**), ^{1}H NMR in MeOH-d_4, 500 MHz

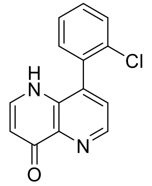

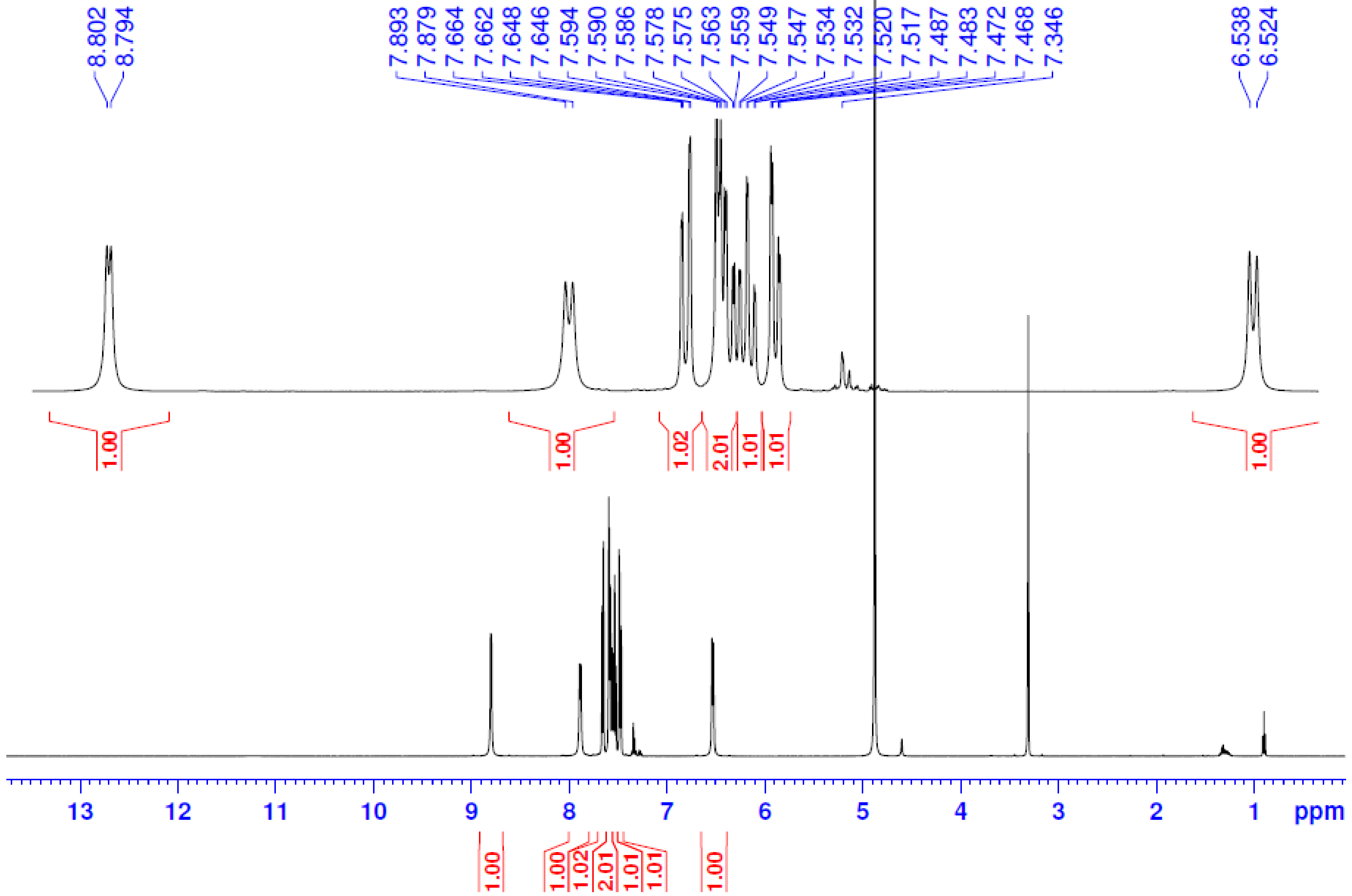

Current Data Parameters
NAME Maria
EXPNO 1098
PROCNO 1

F2 - Acquisition Parameters
Date_ 20210529
Time 15.46
INSTRUM spect
PROBHD 5 mm PABBO BB-
PULPROG zg30
TD 65536
SOLVENT CD3OD_SPE
NS 16
DS 2
SWH 10000.000 Hz
FIDRES 0.152588 Hz
AQ 3.2767999 sec
RG 114
DW 50.000 usec
DE 6.50 usec
TE 297.4 K
D1 1.00000000 sec
TD0 1

======== CHANNEL f1 ====
SFO1 500.0361158 MHz
NUC1 1H
P1 12.00 usec
PLW1 14.50000000 W

F2 - Processing parameters
SI 65536
SF 500.0330377 MHz
WDW EM
SSB 0
LB 0.30 Hz
GB 0
PC 1.00

8-(2-Chlorophenyl)-1,5-naphthyridin-4(1*H*)-one (**7**), ^{13}C NMR in MeOH-d_4, 125 MHz

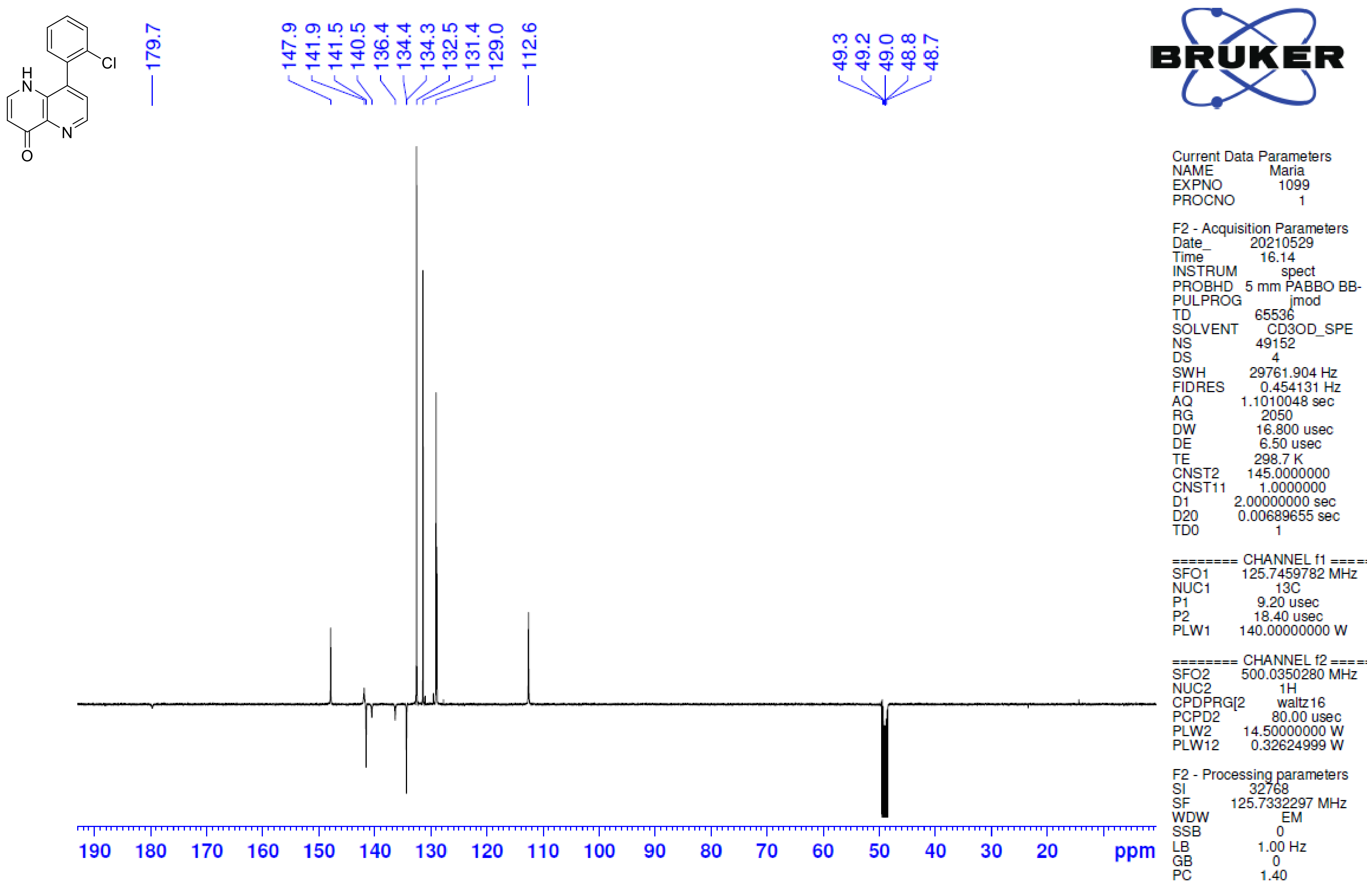

8-(2-Chlorophenyl)-1,5-naphthyridin-4(1*H*)-one (**7**), 2D NMR ^{1}H-^{13}C HSQC in MeOH-d_4, 500 MHz

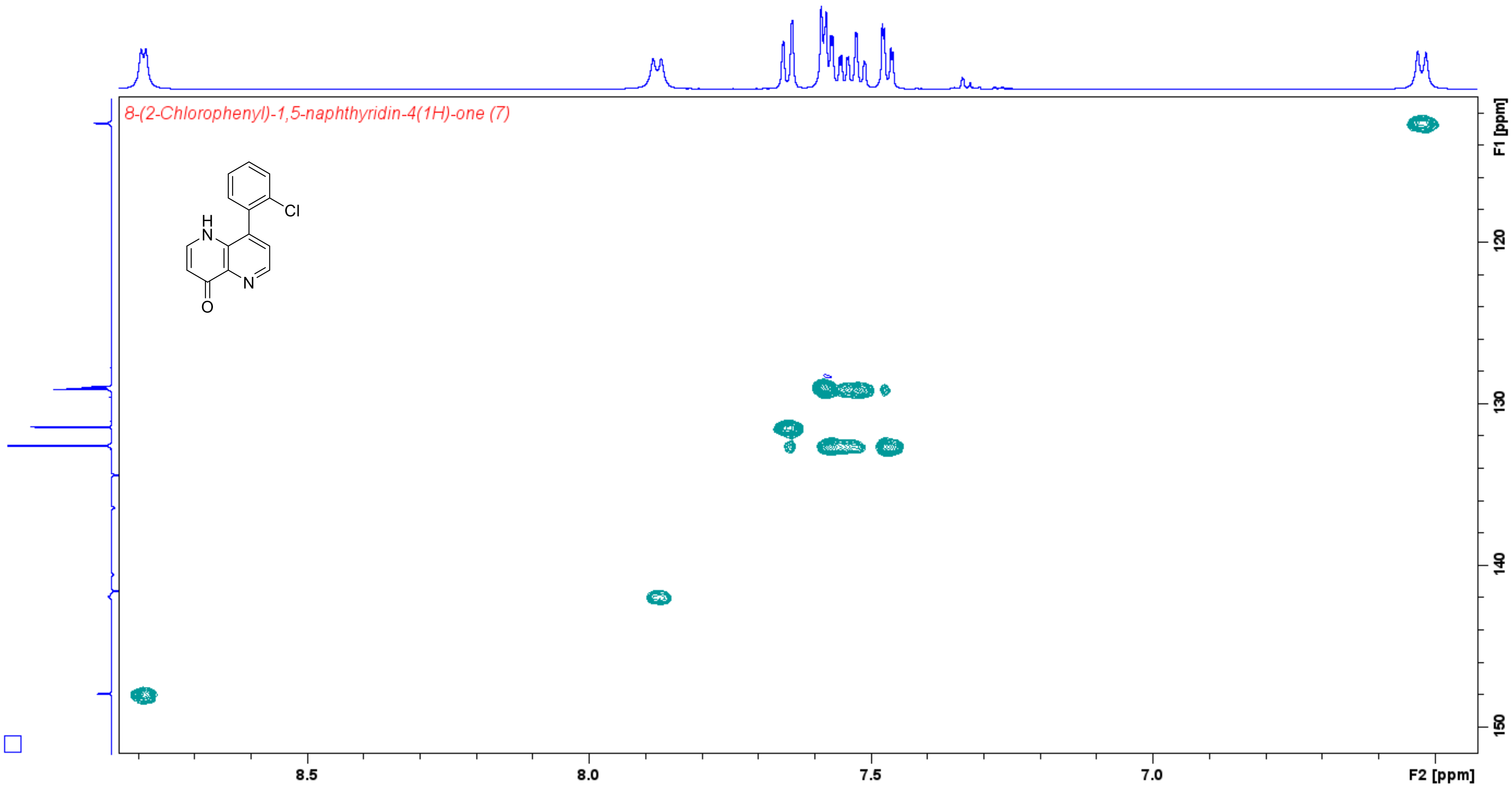

8-(2-Chlorophenyl)-1,5-naphthyridin-4(1*H*)-one (**7**), 2D NMR ^{1}H-^{13}C HSQC in MeOH-d_4, 500 MHz

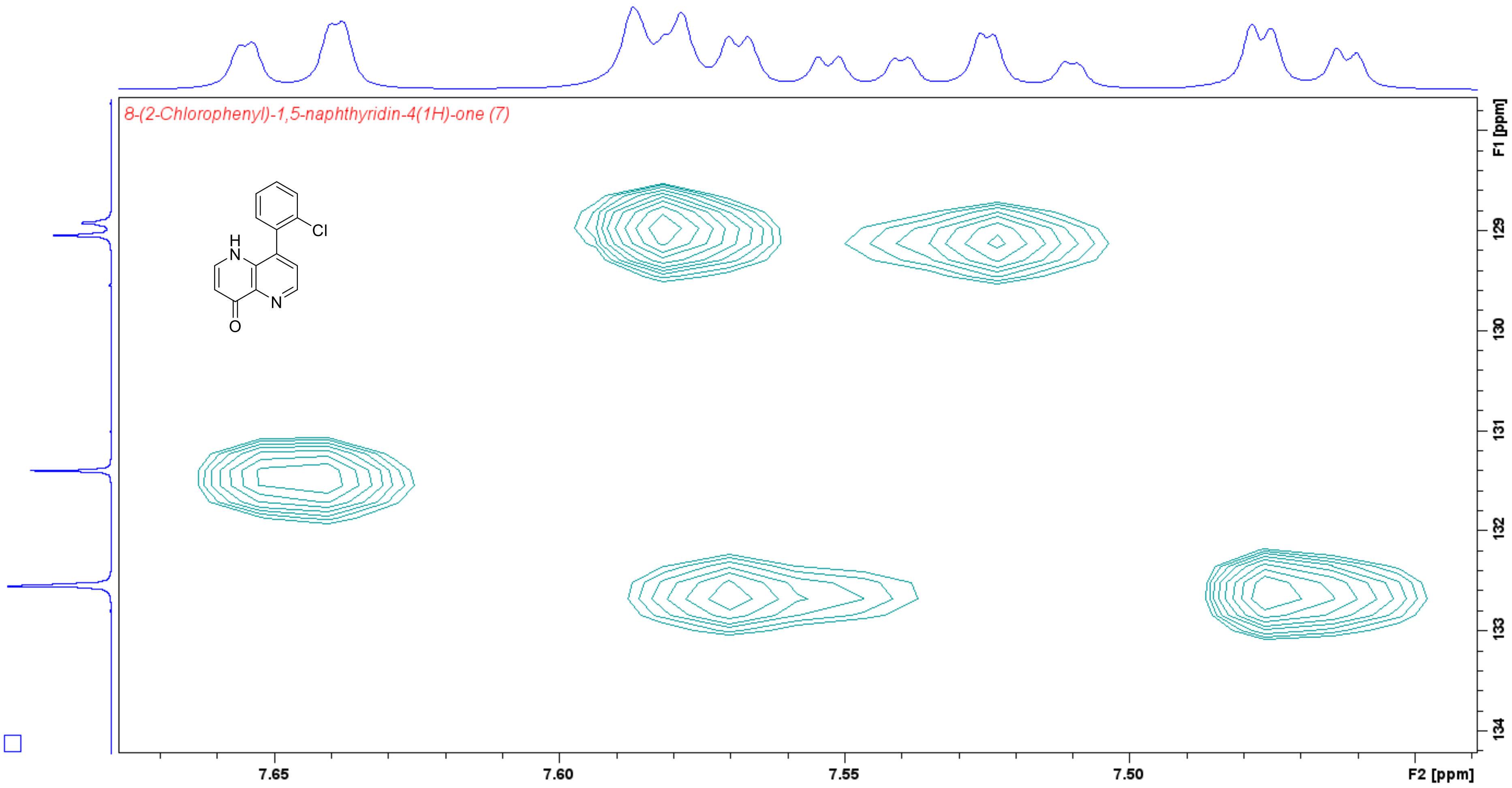

4*H*-Indolo[3,2,1-*de*][1,5]naphthyridin-4-one (canthin-4-one) (**8a**), ^{1}H NMR in $CDCl_3$, 500 MHz

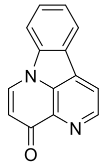

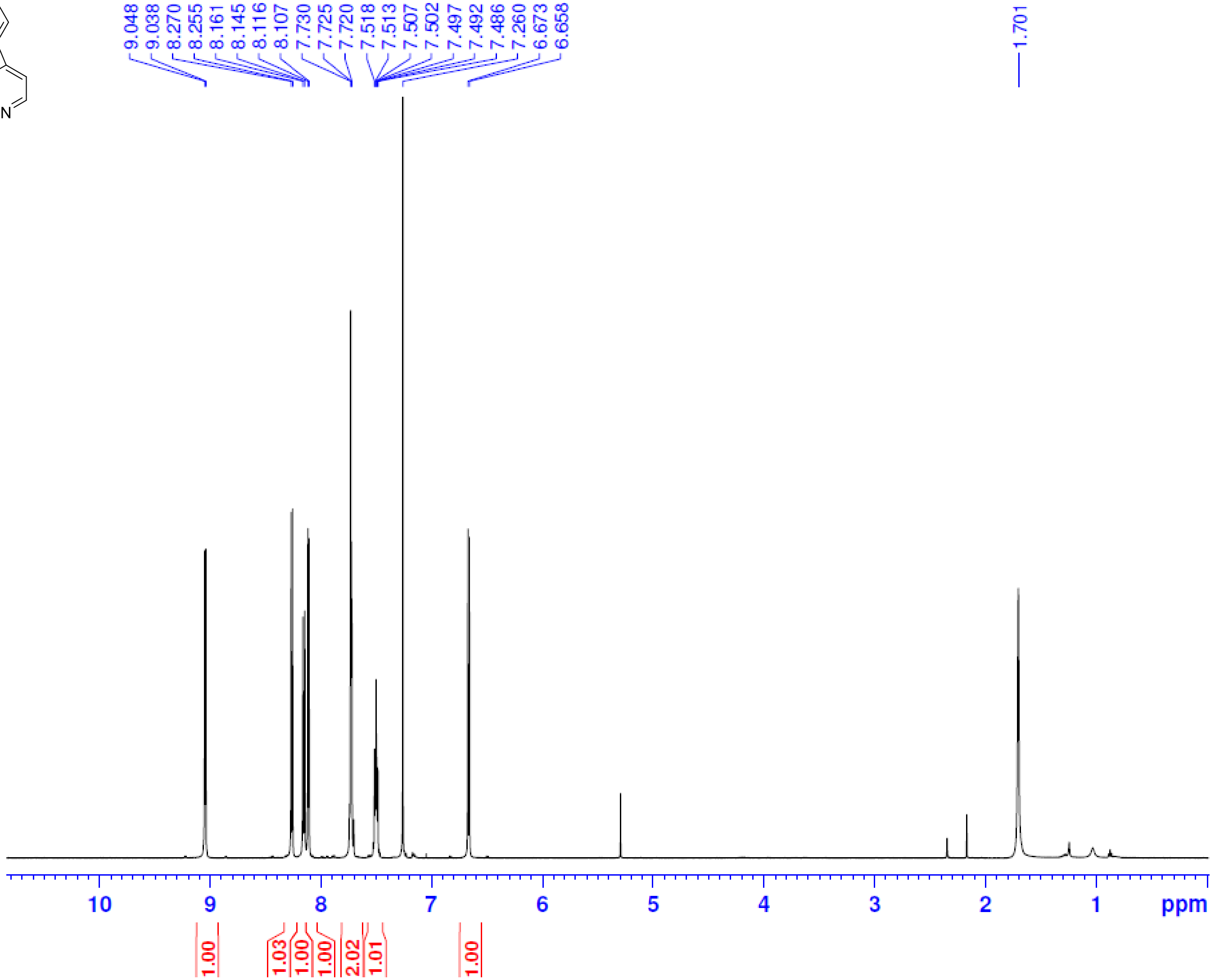

Current Data Parameters
NAME CANTHIN4ONE
EXPNO 1074
PROCNO 1

F2 - Acquisition Parameters
Date_ 20210506
Time 20.39
INSTRUM spect
PROBHD 5 mm PABBO BB-
PULPROG zg30
TD 65536
SOLVENT CDCl3
NS 16
DS 2
SWH 10000.000 Hz
FIDRES 0.152588 Hz
AQ 3.2767999 sec
RG 161
DW 50.000 usec
DE 6.50 usec
TE 295.7 K
D1 1.00000000 sec
TD0 1

======== CHANNEL f1 ====
SFO1 500.0361158 MHz
NUC1 1H
P1 12.00 usec
PLW1 14.50000000 W

F2 - Processing parameters
SI 65536
SF 500.0330403 MHz
WDW EM
SSB 0
LB 0.30 Hz
GB 0
PC 1.00

4*H*-Indolo[3,2,1-*de*][1,5]naphthyridin-4-one (canthin-4-one) (**8a**), ^{13}C NMR in $CDCl_3$, 125 MHz

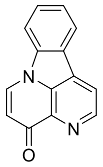

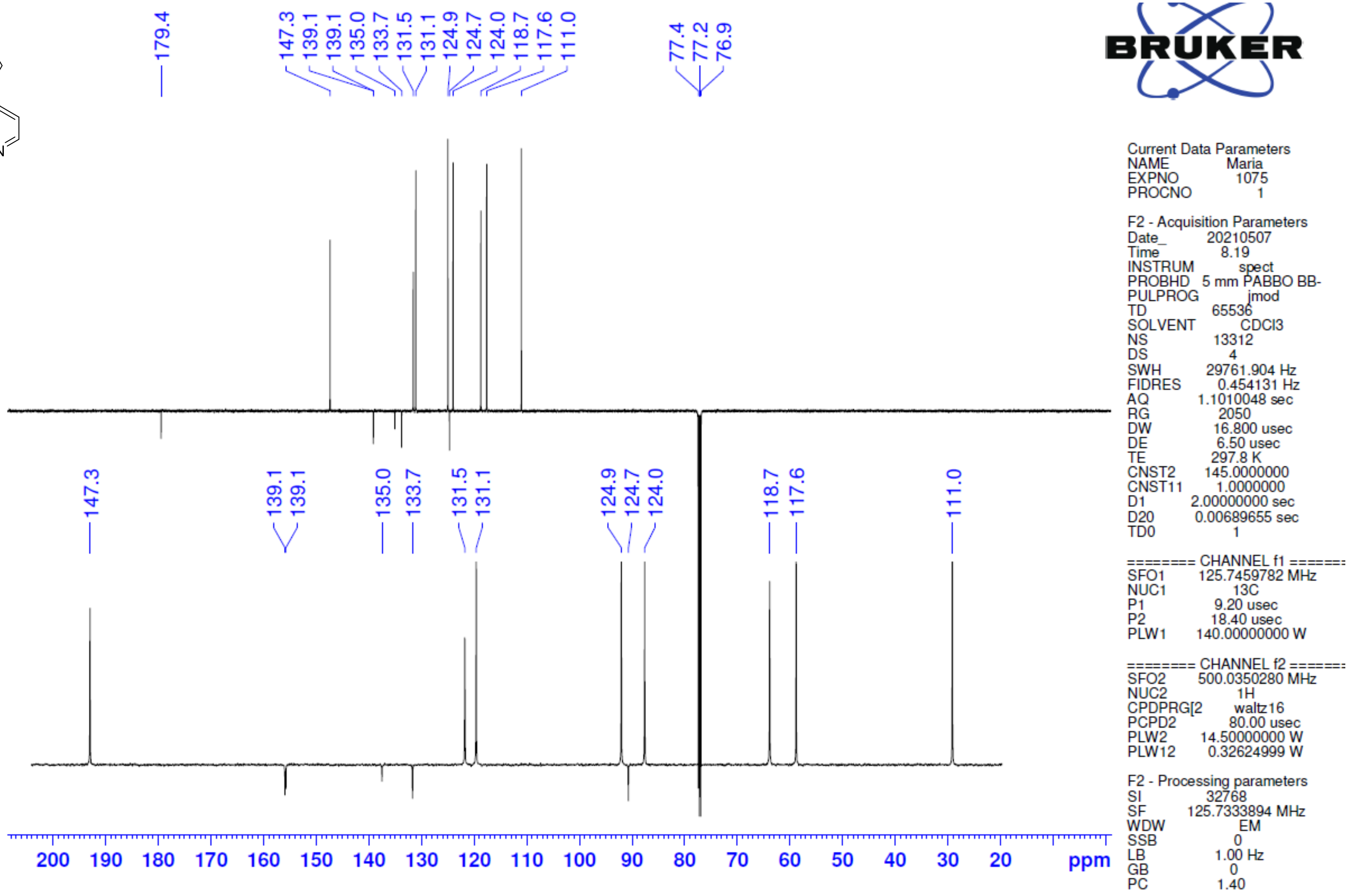

9-Methyl-4*H*-indolo[3,2,1-*de*][1,5]naphthyridin-4-one (**8b**), ^{1}H NMR in $CDCl_3$, 500 MHz

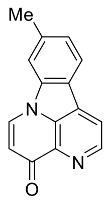

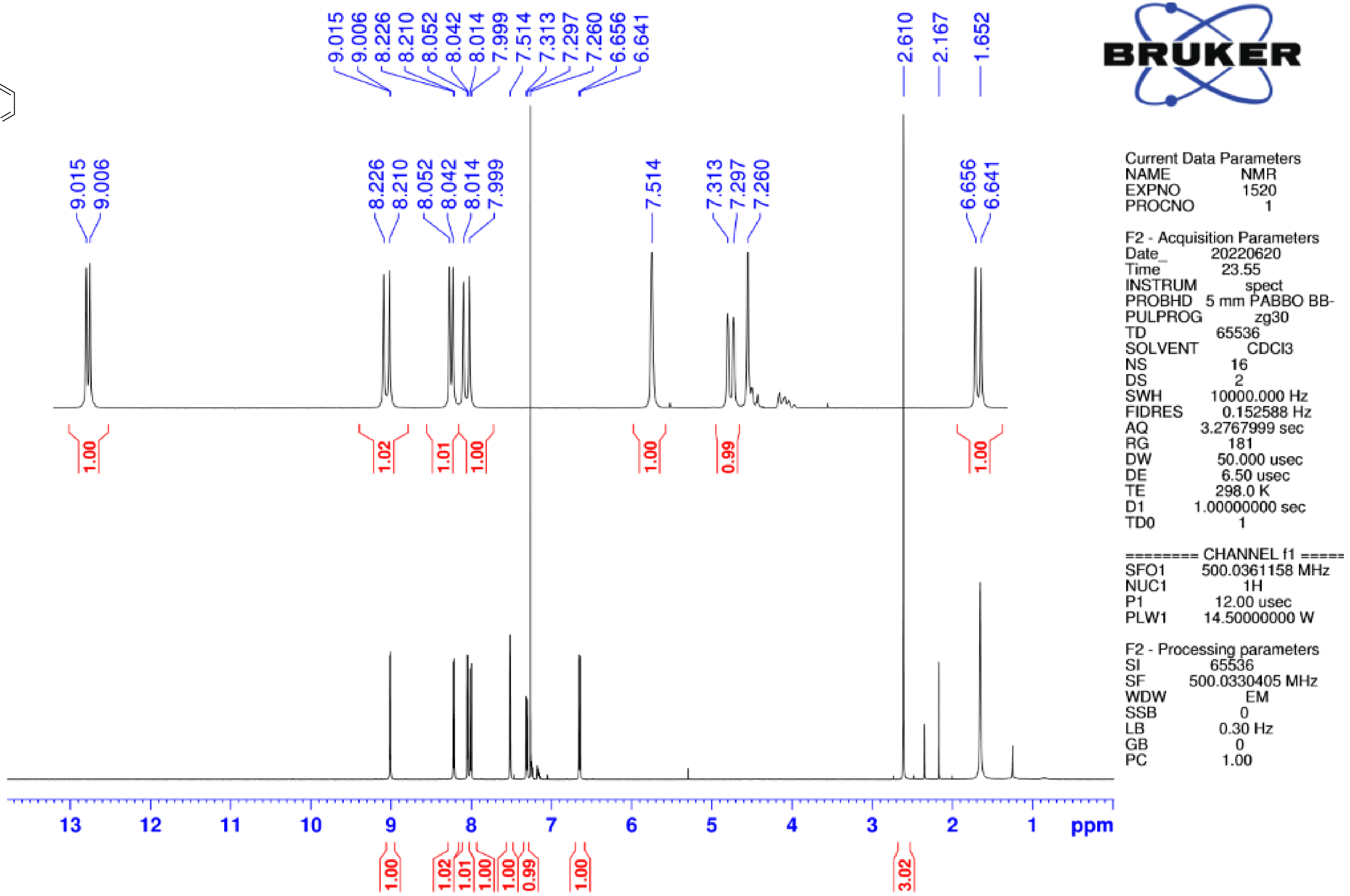

9-Methyl-4*H*-indolo[3,2,1-*de*][1,5]naphthyridin-4-one (**8b**), ^{13}C NMR in $CDCl_3$, 125 MHz

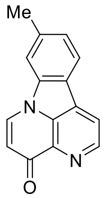

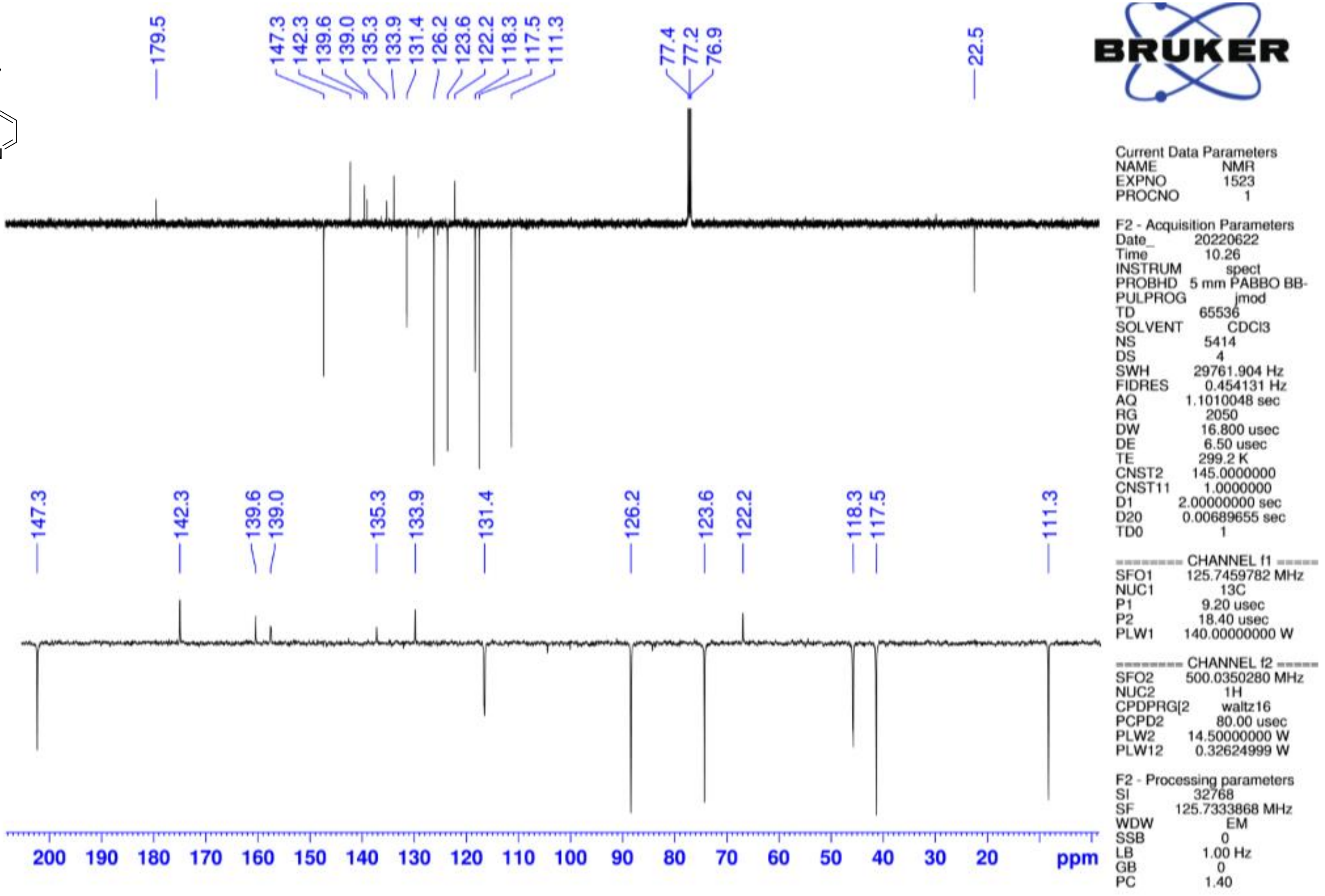

9-Methoxy-4*H*-indolo[3,2,1-*de*][1,5]naphthyridin-4-one (**8c**), ^{1}H NMR in $CDCl_3$, 500 MHz

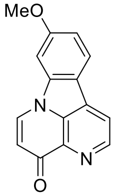

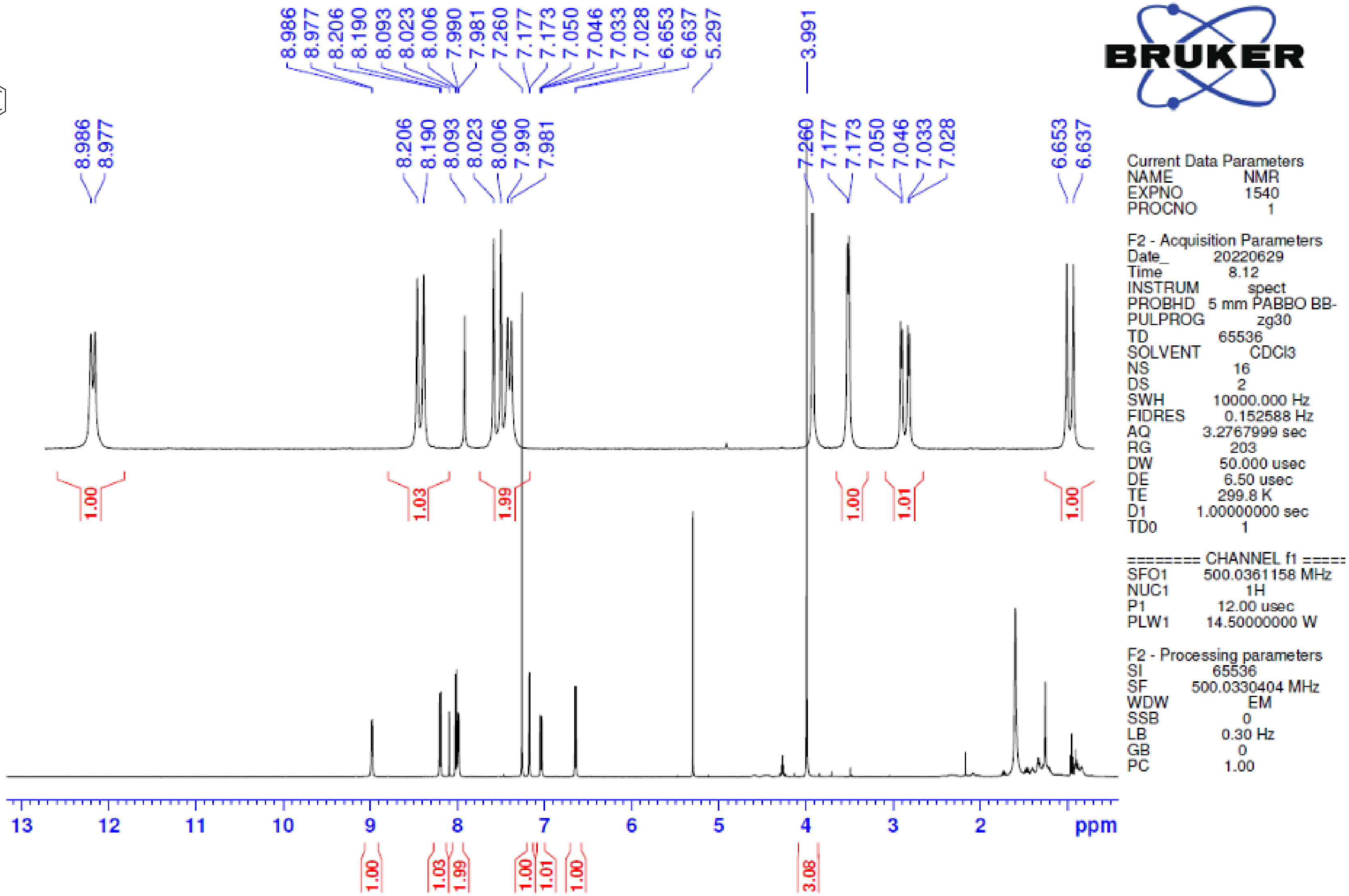

9-Methoxy-4*H*-indolo[3,2,1-*de*][1,5]naphthyridin-4-one (**8c**), ^{13}C NMR in $CDCl_3$, 125 MHz

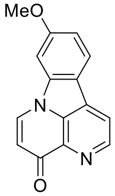

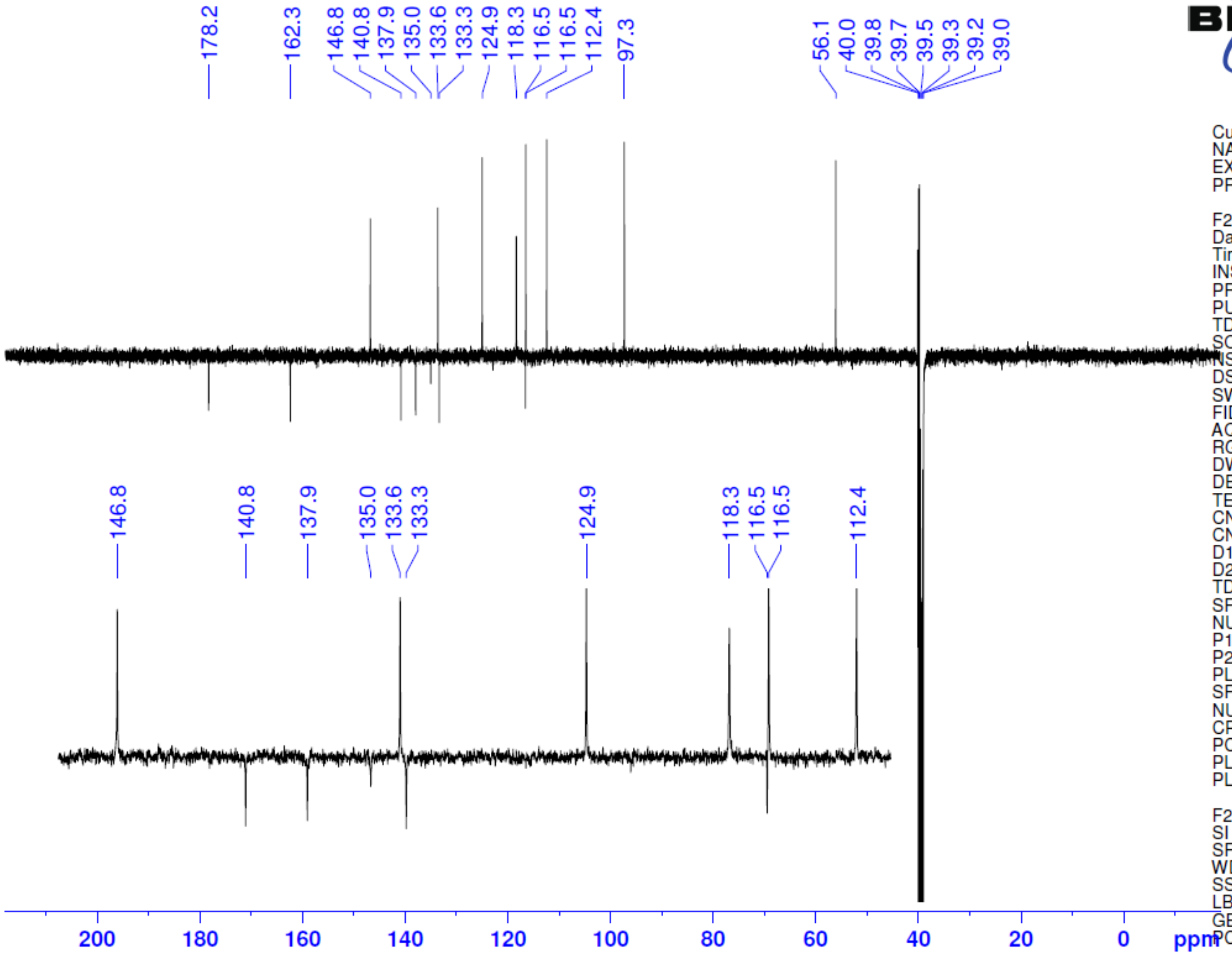

Current Data Parameters
NAME Maria Aug2022-
EXPNO 138
PROCNO 1

F2 - Acquisition Parameters
Date_ 20230829
Time 12.28 h
INSTRUM spect
PROBHD Z113652_0078 (
PULPROG jmod
TD 65536
SOLVENT DMSO
NS 20480
DS 4
SWH 29761.904 Hz
FIDRES 0.908261 Hz
AQ 1.1010048 sec
RG 2050
DW 16.800 usec
DE 6.50 usec
TE 300.4 K
CNST2 145.0000000
CNST11 1.0000000
D1 2.00000000 sec
D20 0.00689655 sec
TD0 1
SFO1 125.7459712 MHz
NUC1 13C
P1 10.00 usec
P2 20.00 usec
PLW1 121.36000061 W
SFO2 500.0350001 MHz
NUC2 1H
CPDPRG[2 waltz65
PCPD2 80.00 usec
PLW2 16.34900093 W
PLW12 0.34647381 W

F2 - Processing parameters
SI 32768
SF 125.7334600 MHz
WDW EM
SSB 0
LB 1.00 Hz
GB 0
PC 1.40

9-(Trifluoromethyl)-4*H*-indolo[3,2,1-*de*][1,5]naphthyridin-4-one (**8d**), ^{1}H NMR in $CDCl_3$, 500 MHz

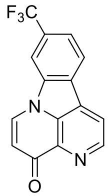

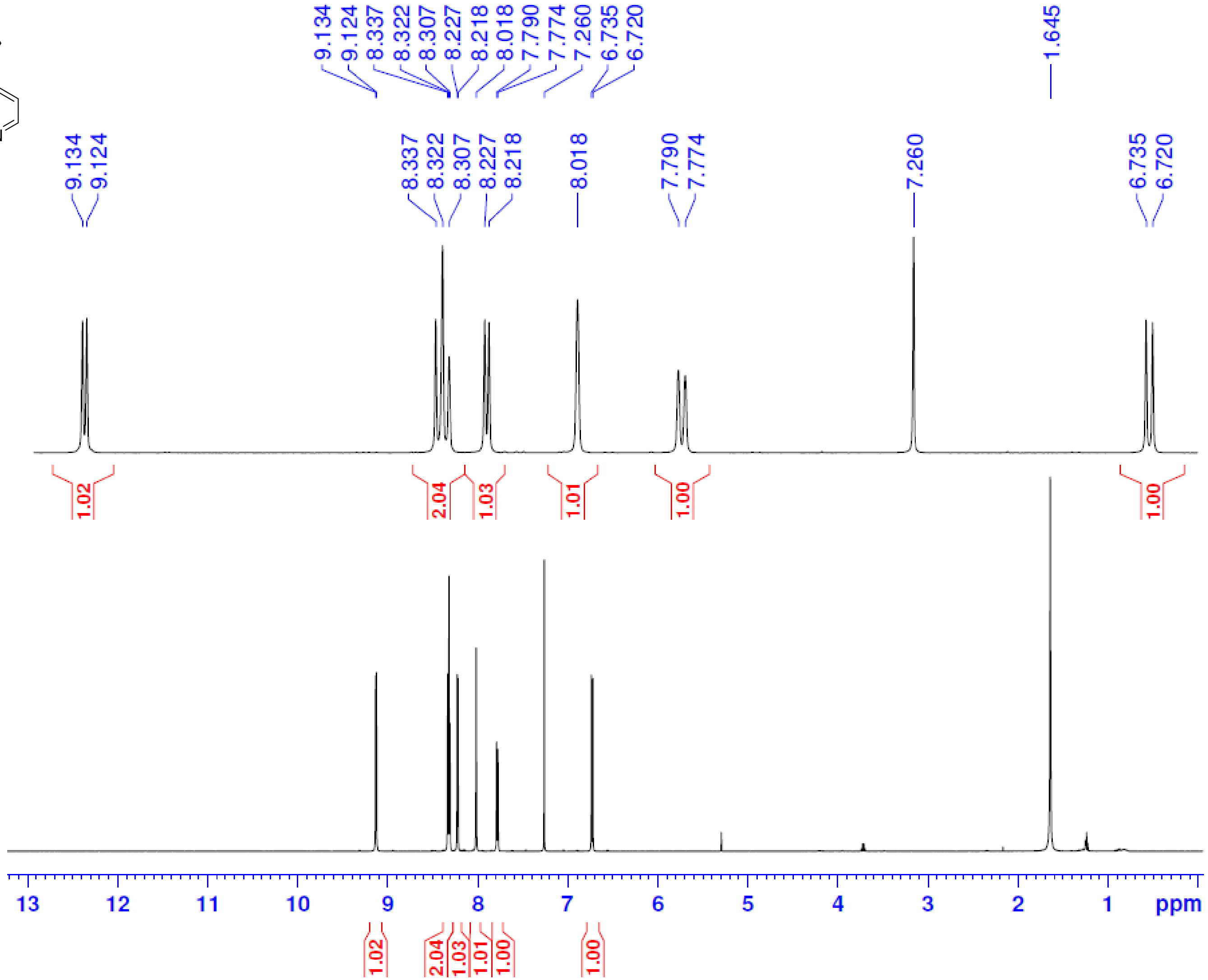

Current Data Parameters
NAME Maria Aug2022-
EXPNO 54
PROCNO 1

F2 - Acquisition Parameters
Date_ 20221203
Time 18.50 h
INSTRUM spect
PROBHD Z113652_0078 (
PULPROG zg30
TD 65536
SOLVENT CDCl3
NS 16
DS 2
SWH 10000.000 Hz
FIDRES 0.305176 Hz
AQ 3.2767999 sec
RG 228
DW 50.000 usec
DE 13.55 usec
TE 294.6 K
D1 1.00000000 sec
TD0 1
SFO1 500.0360877 MHz
NUC1 1H
P0 4.00 usec
P1 12.00 usec
PLW1 16.34900093 W

F2 - Processing parameters
SI 65536
SF 500.0330122 MHz
WDW EM
SSB 0
LB 0.30 Hz
GB 0
PC 1.00

9-(Trifluoromethyl)-4*H*-indolo[3,2,1-*de*][1,5]naphthyridin-4-one (**8d**), ^{13}C NMR in $CDCl_3$, 125 MHz

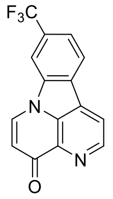

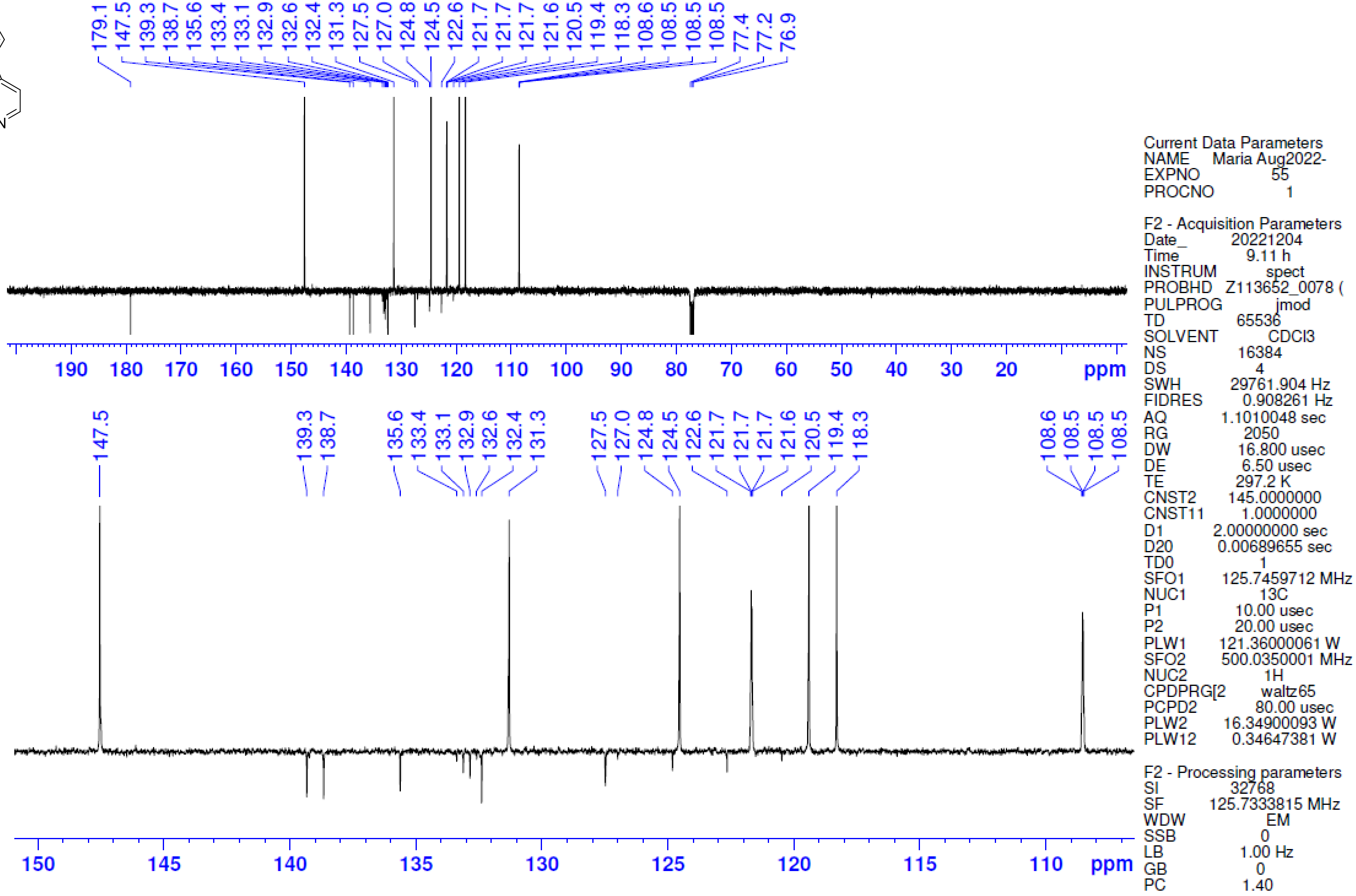

9-Chloro-4*H*-indolo[3,2,1-*de*][1,5]naphthyridin-4-one (**8e**), ^{1}H NMR in $CDCl_3$, 500 MHz

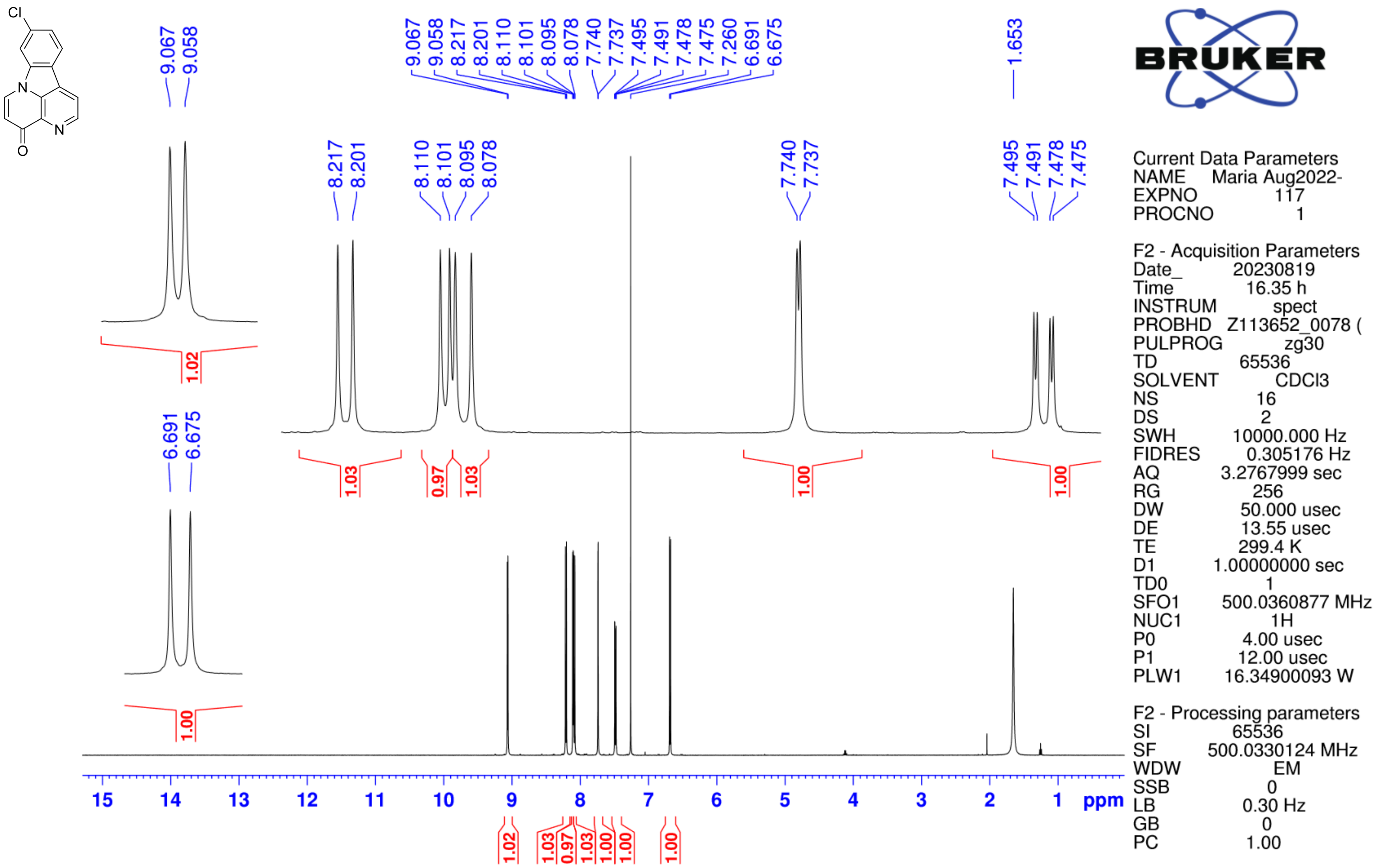

9-Chloro-4*H*-indolo[3,2,1-*de*][1,5]naphthyridin-4-one (**8e**), ^{13}C NMR in $CDCl_3$, 125 MHz

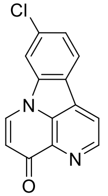

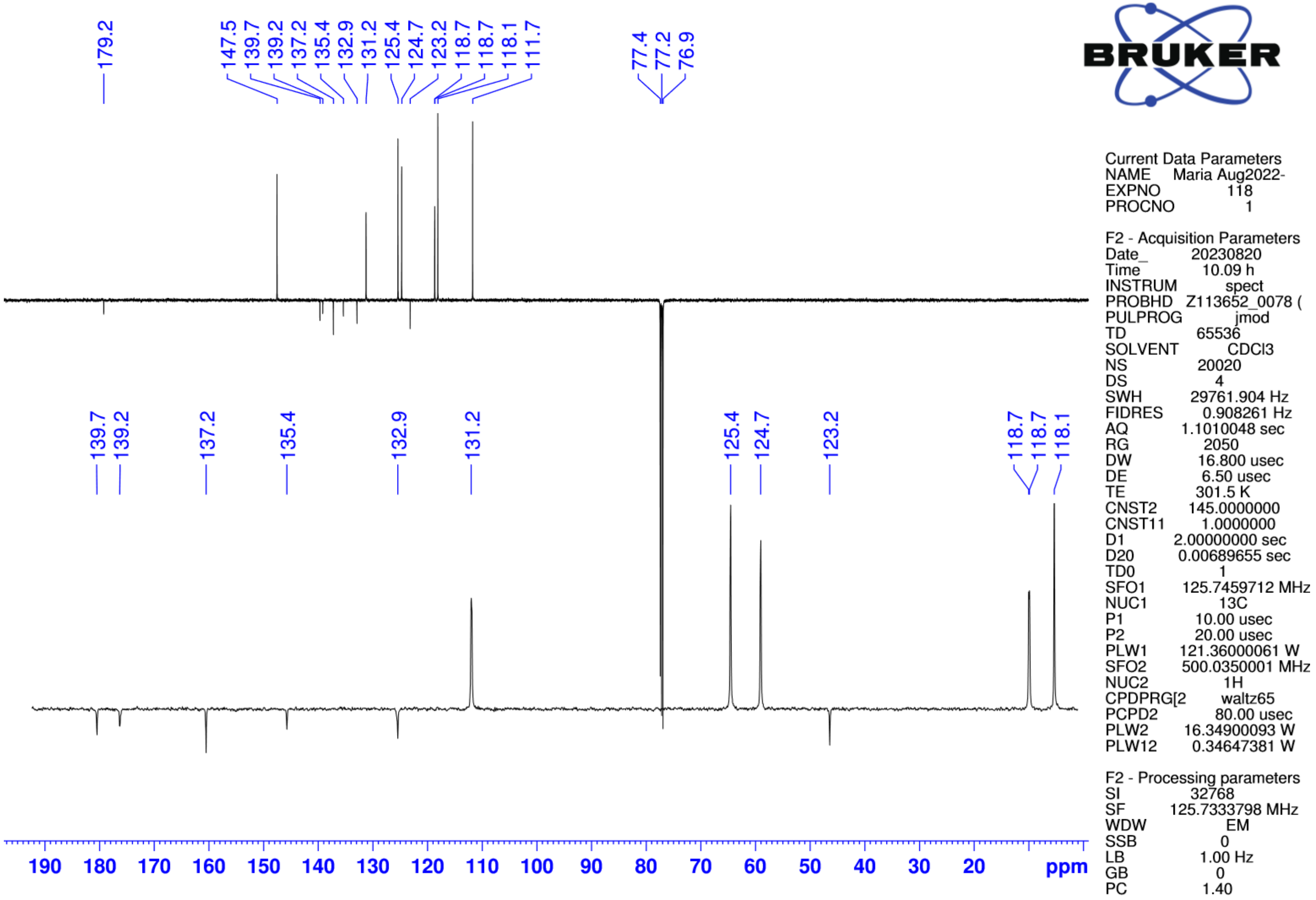

9-Fluoro-4*H*-indolo[3,2,1-*de*][1,5]naphthyridin-4-one (**8f**), ^{1}H NMR in $CDCl_3$, 500 MHz

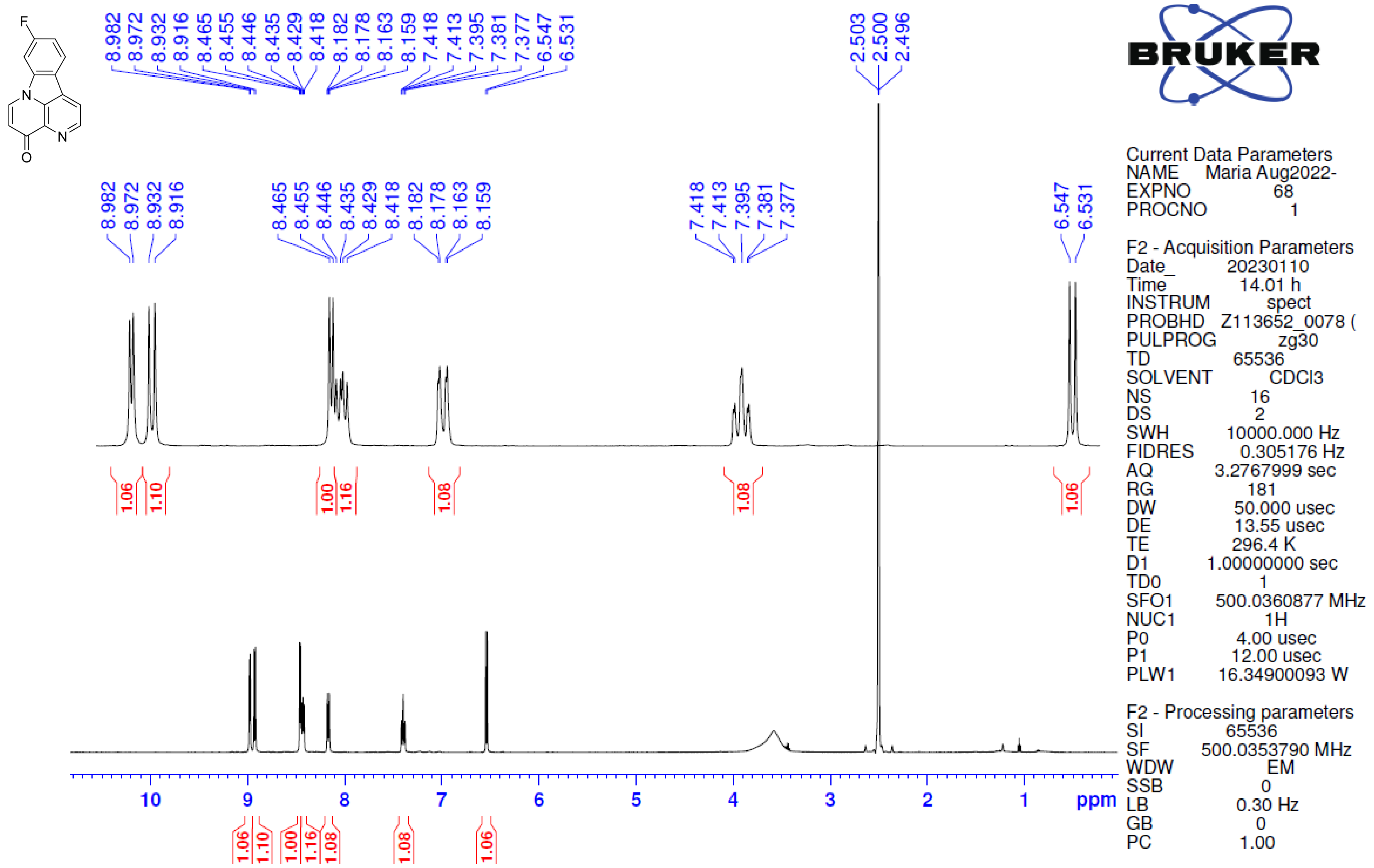

9-Fluoro-4*H*-indolo[3,2,1-*de*][1,5]naphthyridin-4-one (**8f**), ^{13}C NMR in $CDCl_3$, 125 MHz

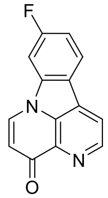

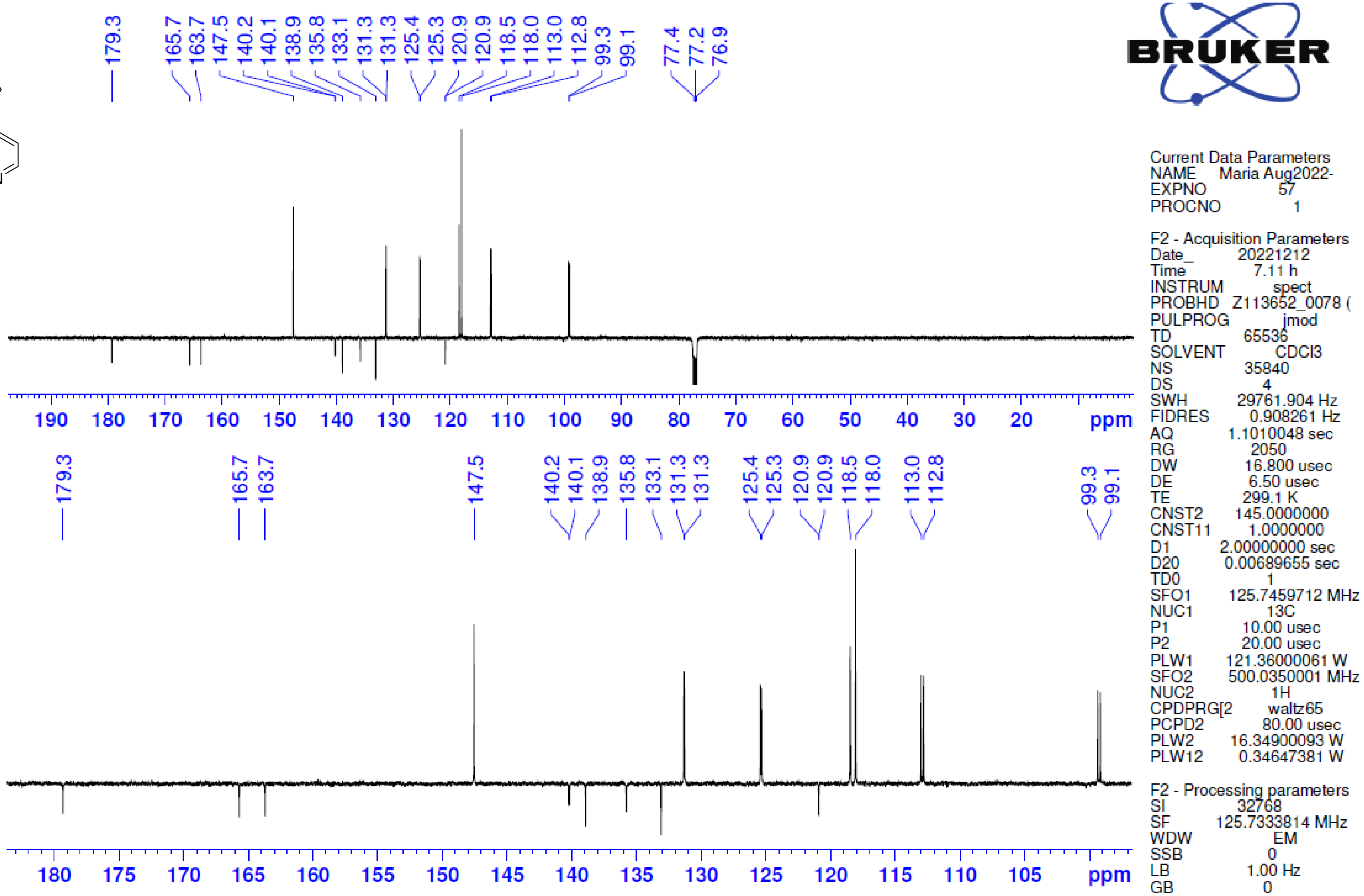

Current Data Parameters
NAME Maria Aug2022-
EXPNO 57
PROCNO 1

F2 - Acquisition Parameters
Date_ 20221212
Time 7.11 h
INSTRUM spect
PROBHD Z113652_0078 (
PULPROG jmod
TD 65536
SOLVENT CDCl3
NS 35840
DS 4
SWH 29761.904 Hz
FIDRES 0.908261 Hz
AQ 1.1010048 sec
RG 2050
DW 16.800 usec
DE 6.50 usec
TE 299.1 K
CNST2 145.0000000
CNST11 1.0000000
D1 2.00000000 sec
D20 0.00689655 sec
TD0 1
SFO1 125.7459712 MHz
NUC1 13C
P1 10.00 usec
P2 20.00 usec
PLW1 121.36000061 W
SFO2 500.0350001 MHz
NUC2 1H
CPDPRG[2 waltz65
PCPD2 80.00 usec
PLW2 16.34900093 W
PLW12 0.34647381 W

F2 - Processing parameters
SI 32768
SF 125.7333814 MHz
WDW EM
SSB 0
LB 1.00 Hz
GB 0
PC 1.40

10-Methoxy-4*H*-indolo[3,2,1-*de*][1,5]naphthyridin-4-one (**8g**), ^{1}H NMR in $CDCl_3$, 500 MHz

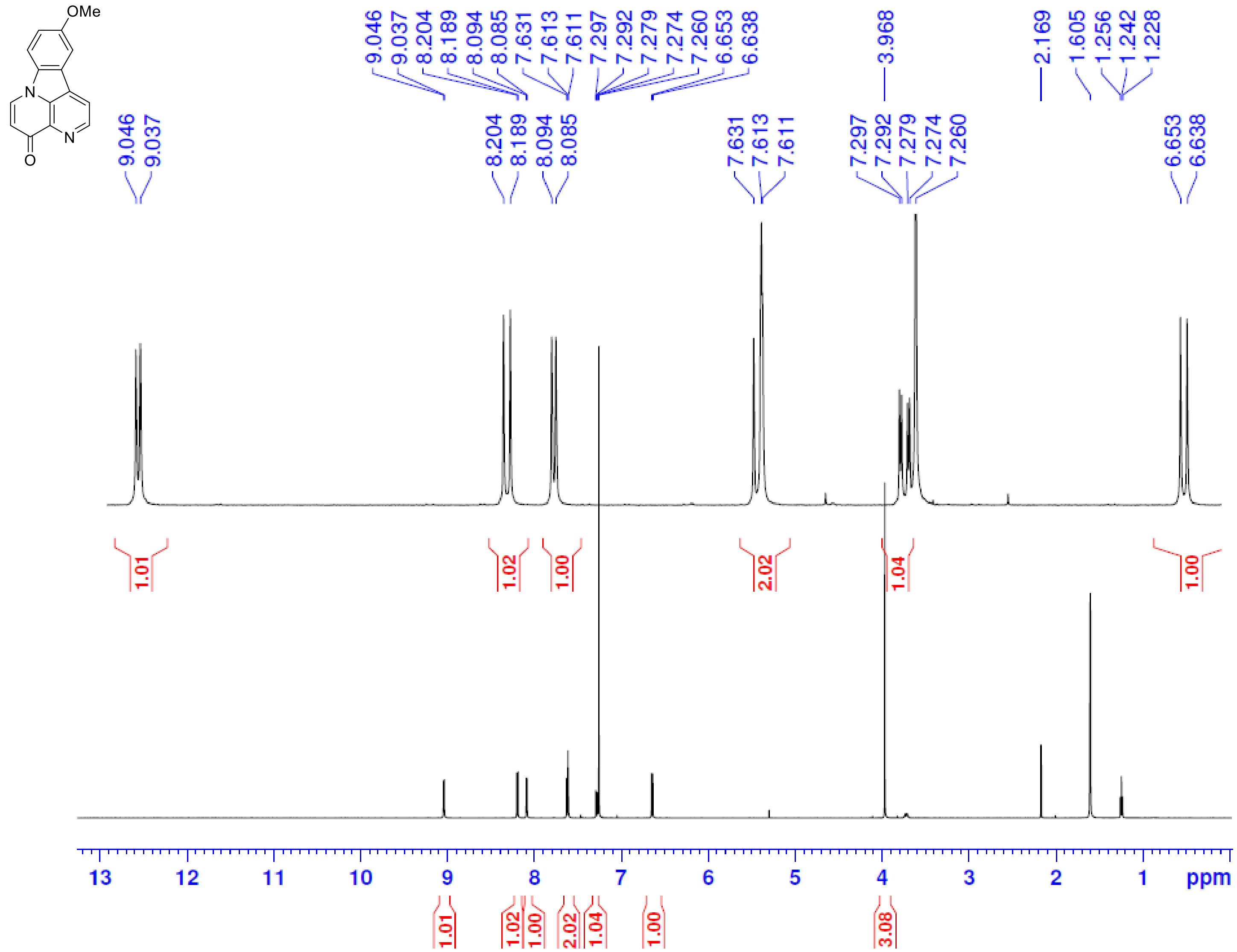

Current Data Parameters
NAME Maria
EXPNO 1521
PROCNO 1

F2 - Acquisition Parameters
Date_ 20220621
Time 0
INSTRUM spect
PROBHD 5 mm PABBO BB-
PULPROG zg30
TD 65536
SOLVENT CDCl3
NS 16
DS 2
SWH 10000.000 Hz
FIDRES 0.152588 Hz
AQ 3.2767999 sec
RG 203
DW 50.000 usec
DE 6.50 usec
TE 298.1 K
D1 1.00000000 sec
TD0 1

======== CHANNEL f1 ====
SFO1 500.0361158 MHz
NUC1 1H
P1 12.00 usec
PLW1 14.50000000 W

F2 - Processing parameters
SI 65536
SF 500.0330405 MHz
WDW EM
SSB 0
LB 0.30 Hz
GB 0
PC 1.00

10-Methoxy-4*H*-indolo[3,2,1-*de*][1,5]naphthyridin-4-one (**8g**), ^{13}C NMR in $CDCl_3$, 125 MHz

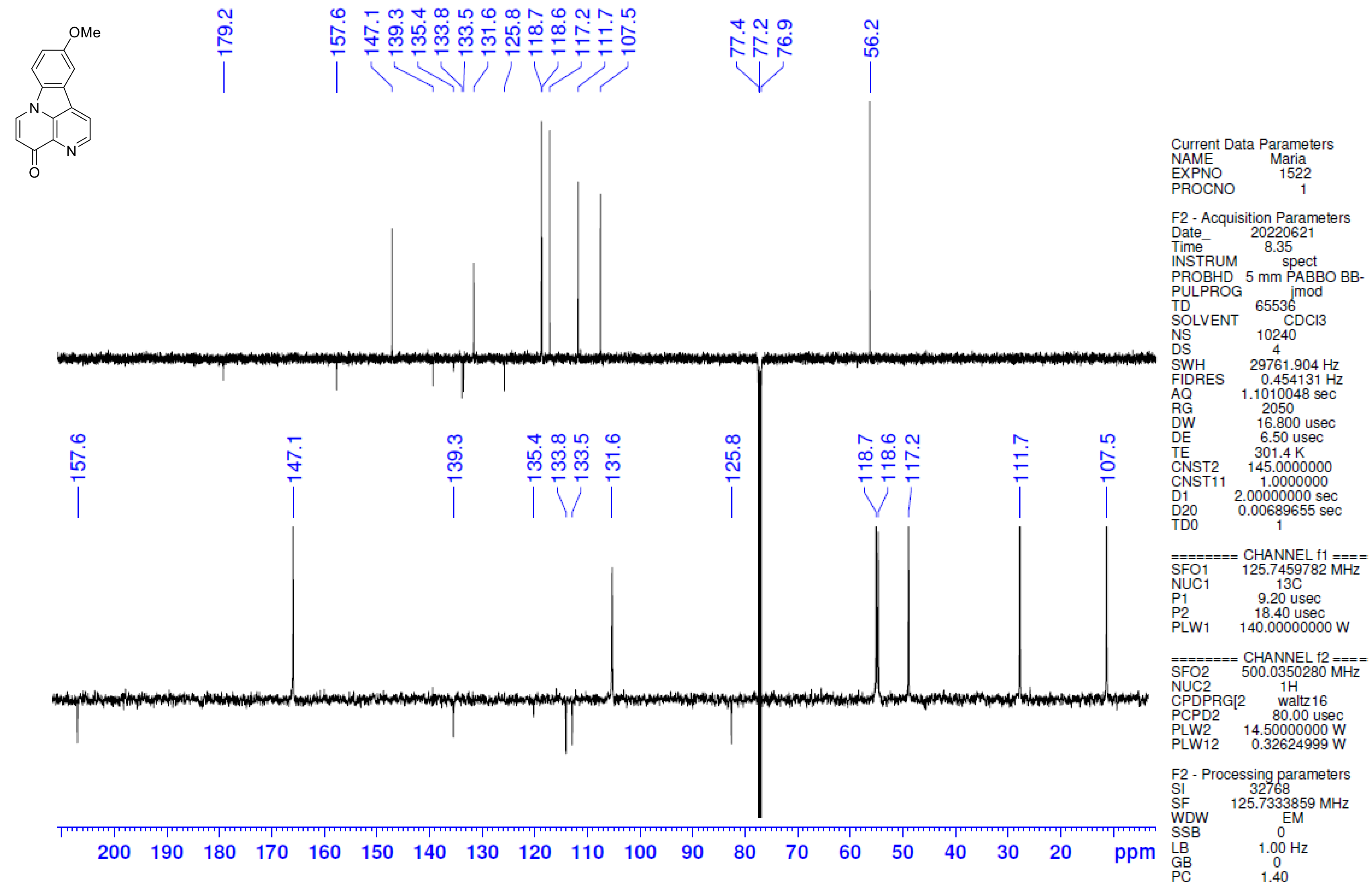

10-(Trifluoromethoxy)-4*H*-indolo[3,2,1-*de*][1,5]naphthyridin-4-one (**8h**), ^{1}H NMR in $CDCl_3$, 500 MHz

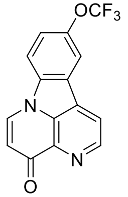

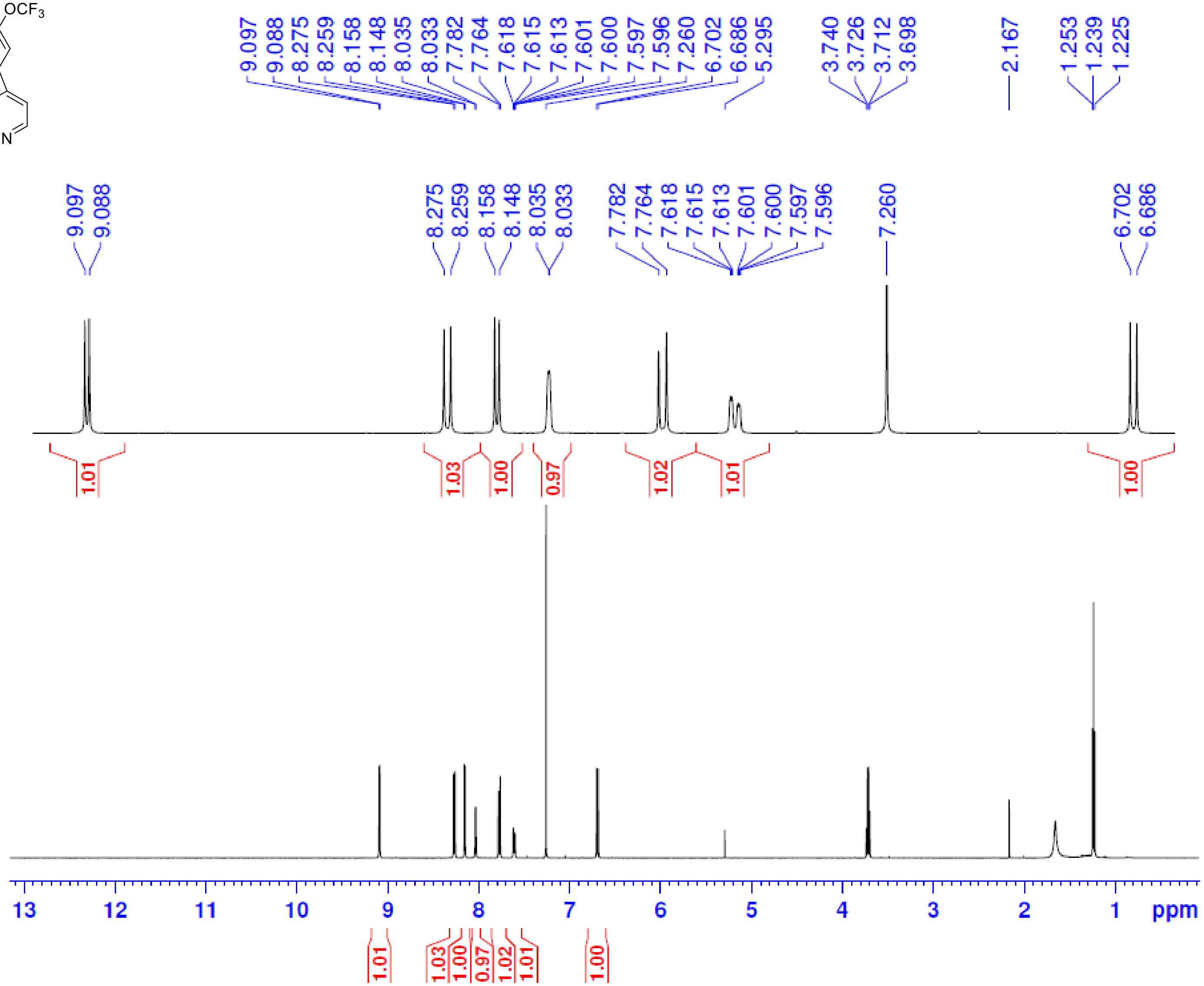

Current Data Parameters
NAME Maria
EXPNO 1507
PROCNO 1

F2 - Acquisition Parameters
Date_ 20220619
Time 17.43
INSTRUM spect
PROBHD 5 mm PABBO BB-
PULPROG zg30
TD 65536
SOLVENT CDCl3
NS 16
DS 2
SWH 10000.000 Hz
FIDRES 0.152588 Hz
AQ 3.2767999 sec
RG 161
DW 50.000 usec
DE 6.50 usec
TE 296.2 K
D1 1.00000000 sec
TD0 1

======== CHANNEL f1 ====
SFO1 500.0361158 MHz
NUC1 1H
P1 12.00 usec
PLW1 14.50000000 W

F2 - Processing parameters
SI 65536
SF 500.0330404 MHz
WDW EM
SSB 0
LB 0.30 Hz
GB 0
PC 1.00

10-(Trifluoromethoxy)-4*H*-indolo[3,2,1-*de*][1,5]naphthyridin-4-one (**8h**), ^{13}C NMR in $CDCl_3$, 125 MHz

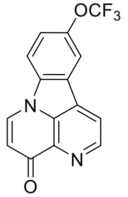

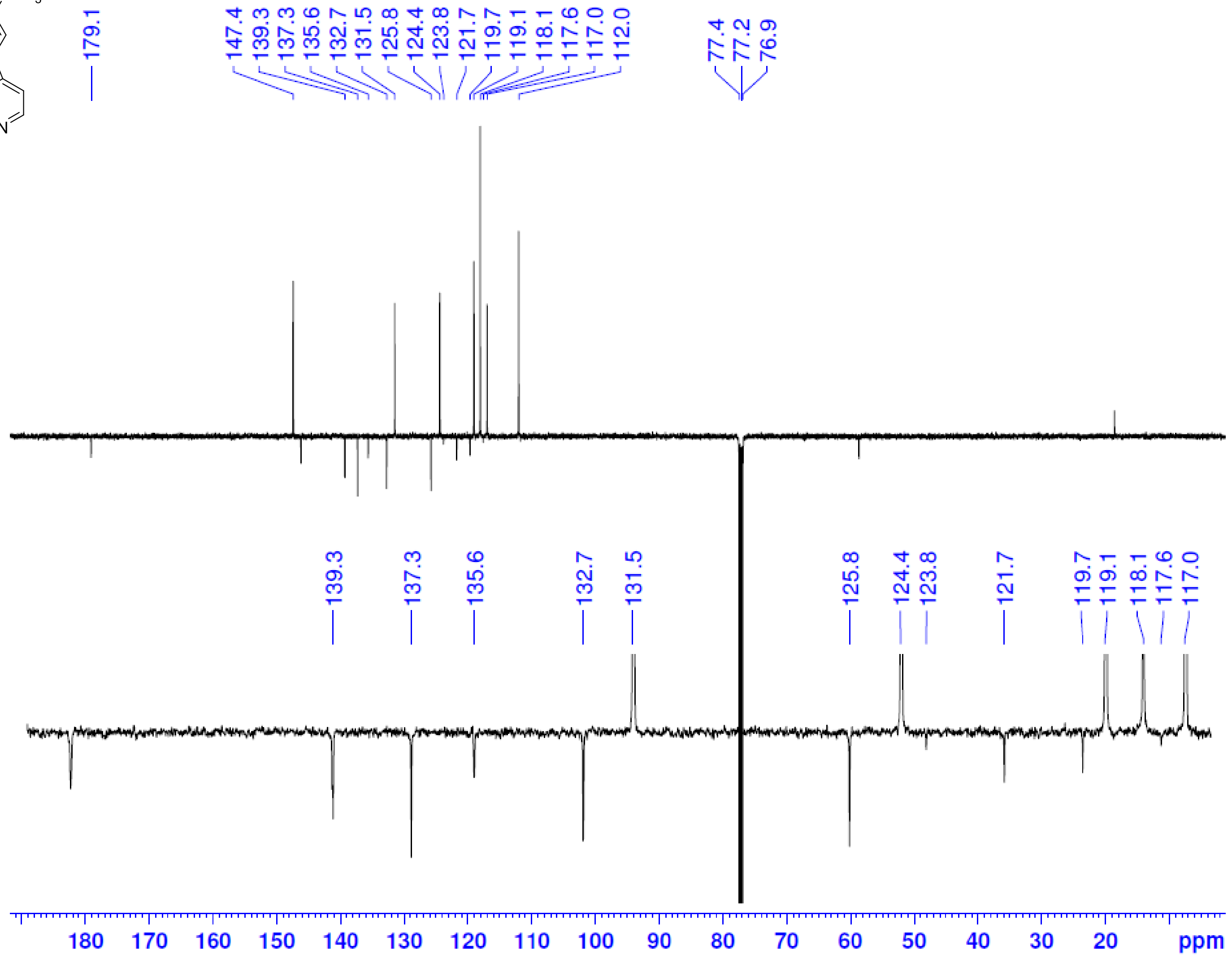

Current Data Parameters
NAME Maria
EXPNO 1508
PROCNO 1

F2 - Acquisition Parameters
Date_ 20220619
Time 21.06
INSTRUM spect
PROBHD 5 mm PABBO BB-
PULPROG jmod
TD 65536
SOLVENT CDCl3
NS 18130
DS 4
SWH 29761.904 Hz
FIDRES 0.454131 Hz
AQ 1.1010048 sec
RG 2050
DW 16.800 usec
DE 6.50 usec
TE 298.0 K
CNST2 145.0000000
CNST11 1.0000000
D1 2.00000000 sec
D20 0.00689655 sec
TD0 1

======== CHANNEL f1 =====
SFO1 125.7459782 MHz
NUC1 13C
P1 9.20 usec
P2 18.40 usec
PLW1 140.00000000 W

======== CHANNEL f2 =====
SFO2 500.0350280 MHz
NUC2 1H
CPDPRG[2 waltz16
PCPD2 80.00 usec
PLW2 14.50000000 W
PLW12 0.32624999 W

F2 - Processing parameters
SI 32768
SF 125.7333876 MHz
WDW EM
SSB 0
LB 1.00 Hz
GB 0
PC 1.40

10-(Trifluoromethyl)-4*H*-indolo[3,2,1-*de*][1,5]naphthyridin-4-one (**8i**), ^{1}H NMR in $CDCl_3$, 500 MHz

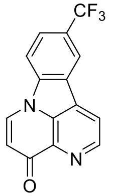

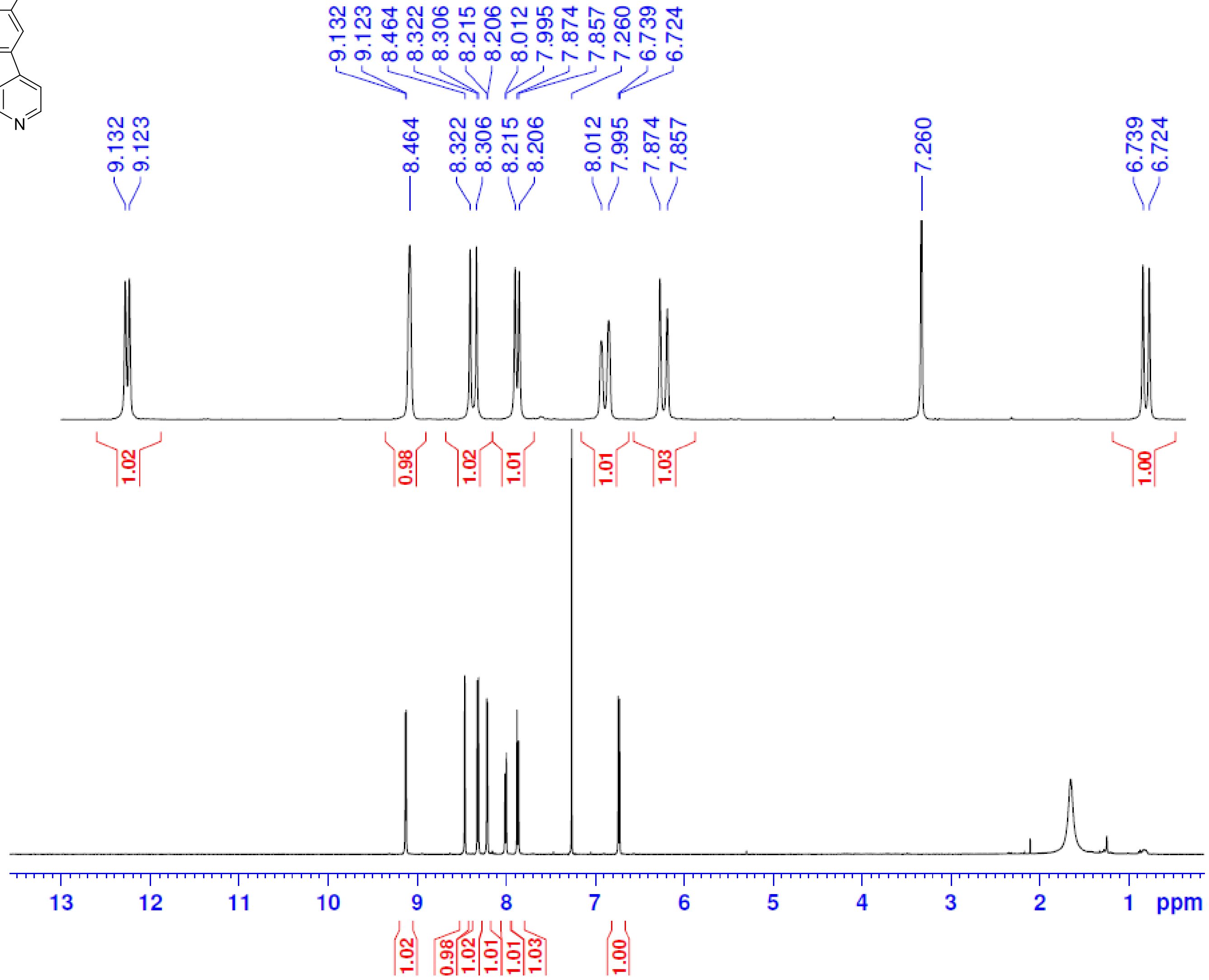

Current Data Parameters
NAME Maria Aug2022-
EXPNO 42
PROCNO 1

F2 - Acquisition Parameters
Date_ 20221029
Time 19.19 h
INSTRUM spect
PROBHD Z113652_0078 (
PULPROG zg30
TD 65536
SOLVENT CDCl3
NS 16
DS 2
SWH 10000.000 Hz
FIDRES 0.305176 Hz
AQ 3.2767999 sec
RG 228
DW 50.000 usec
DE 13.55 usec
TE 294.1 K
D1 1.00000000 sec
TD0 1
SFO1 500.0360877 MHz
NUC1 1H
P0 4.00 usec
P1 12.00 usec
PLW1 16.34900093 W

F2 - Processing parameters
SI 65536
SF 500.0330121 MHz
WDW EM
SSB 0
LB 0.30 Hz
GB 0
PC 1.00

10-(Trifluoromethyl)-4*H*-indolo[3,2,1-*de*][1,5]naphthyridin-4-one (**8i**), ^{13}C NMR in $CDCl_3$, 125 MHz

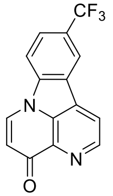

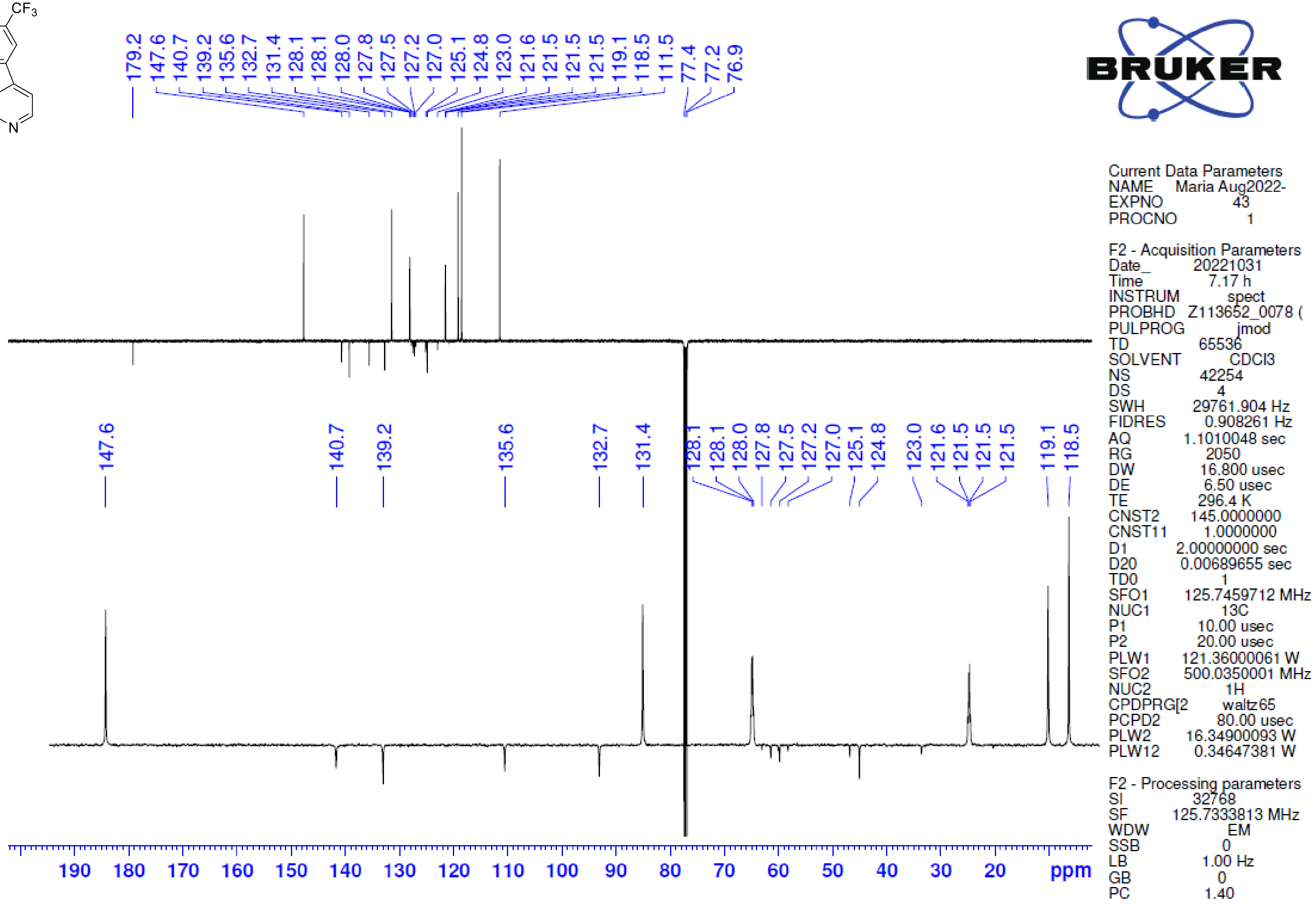

10-Chloro-4*H*-indolo[3,2,1-*de*][1,5]naphthyridin-4-one (**8j**), ^{1}H NMR in $CDCl_3$, 500 MHz

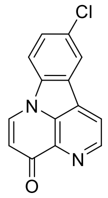

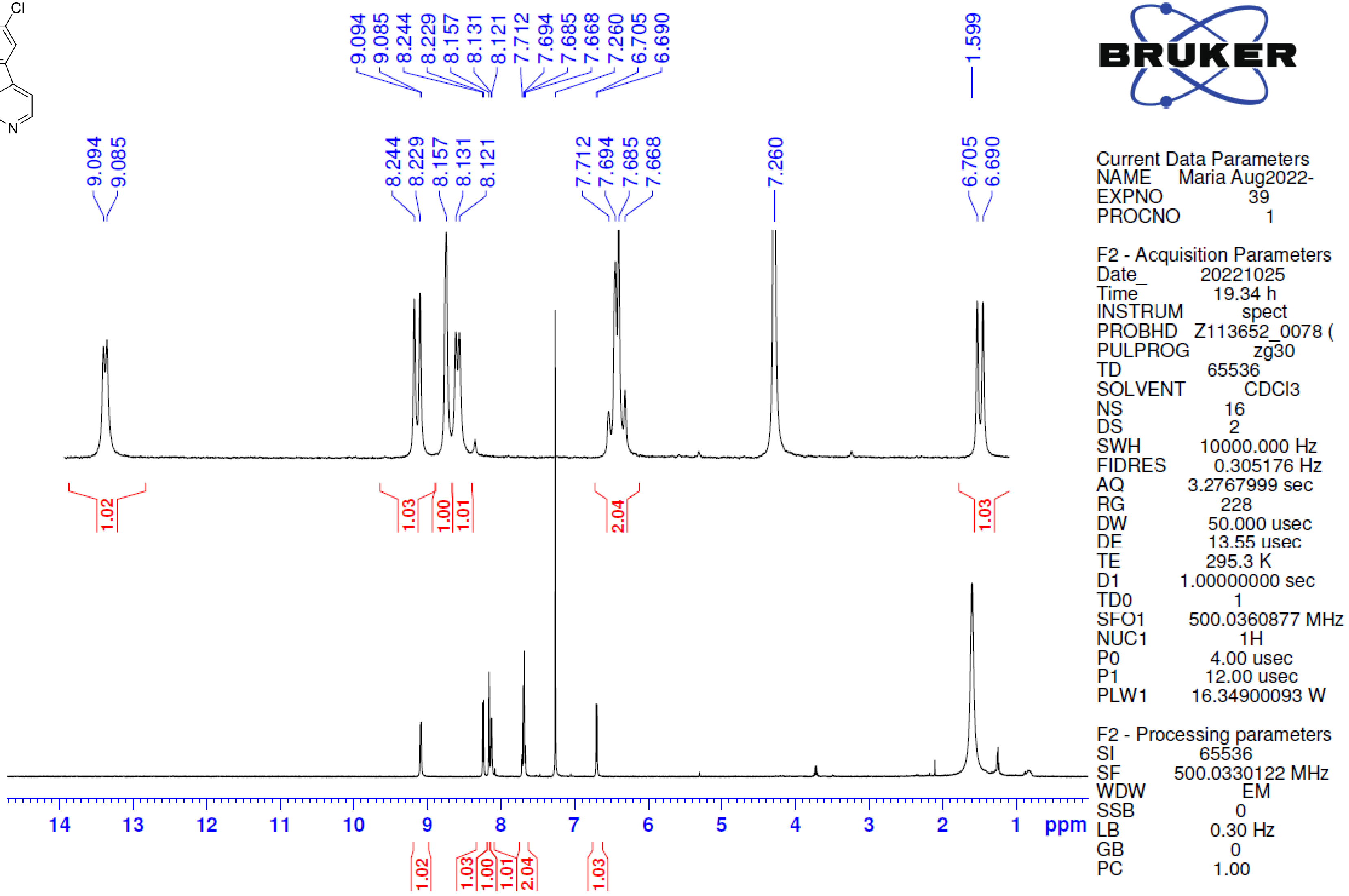

10-Chloro-4*H*-indolo[3,2,1-*de*][1,5]naphthyridin-4-one (**8j**), ^{13}C NMR in $CDCl_3$, 125 MHz

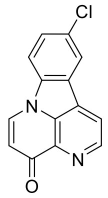

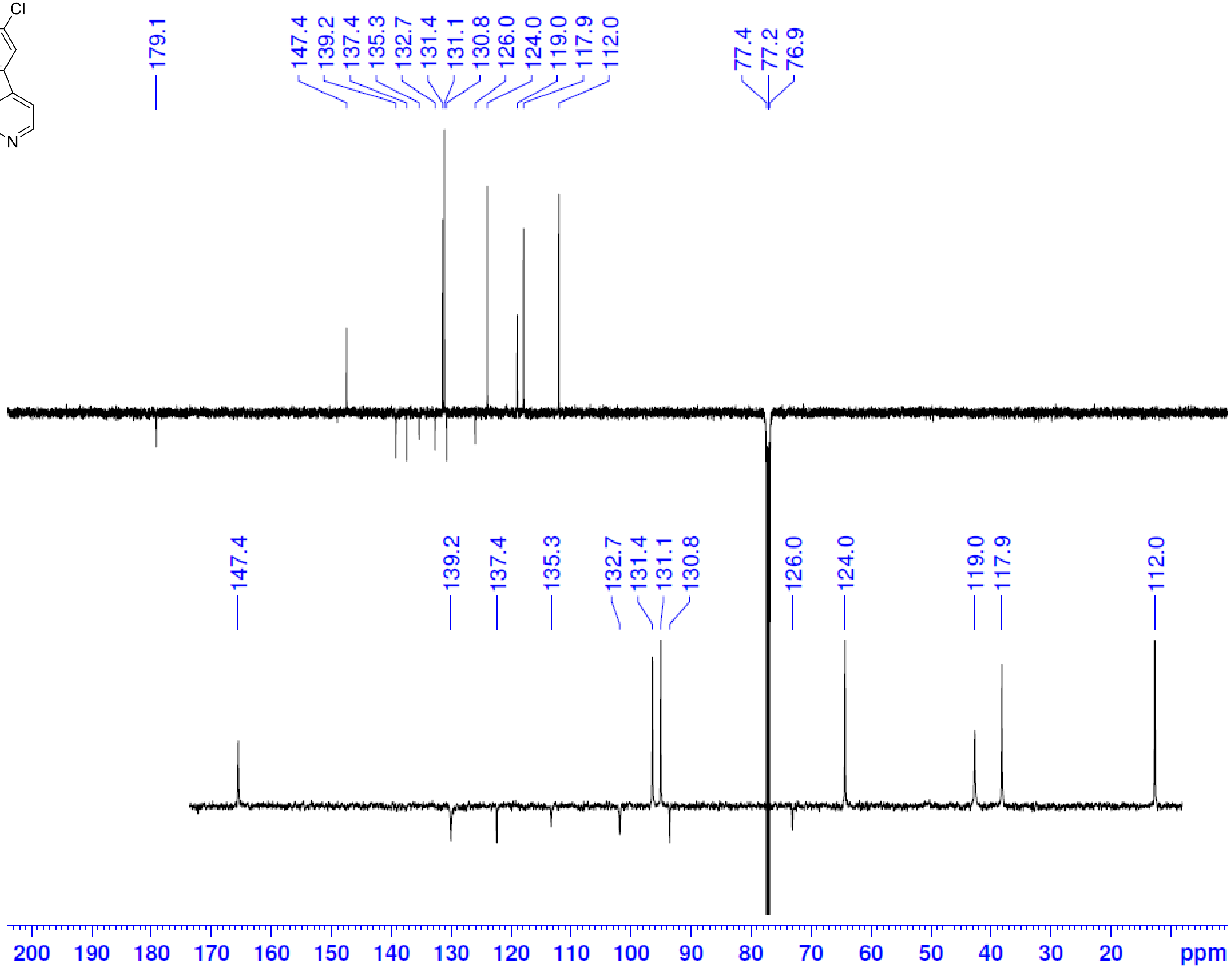

Current Data Parameters
NAME Maria Aug2022-
EXPNO 41
PROCNO 1

F2 - Acquisition Parameters
Date_ 20221028
Time 17.47 h
INSTRUM spect
PROBHD Z113652_0078 (
PULPROG jmod
TD 65536
SOLVENT CDCl3
NS 40960
DS 4
SWH 29761.904 Hz
FIDRES 0.908261 Hz
AQ 1.1010048 sec
RG 2050
DW 16.800 usec
DE 6.50 usec
TE 294.9 K
CNST2 145.0000000
CNST11 1.0000000
D1 2.00000000 sec
D20 0.00689655 sec
TD0 1
SFO1 125.7459712 MHz
NUC1 13C
P1 10.00 usec
P2 20.00 usec
PLW1 121.36000061 W
SFO2 500.0350001 MHz
NUC2 1H
CPDPRG[2 waltz65
PCPD2 80.00 usec
PLW2 16.34900093 W
PLW12 0.34647381 W

F2 - Processing parameters
SI 32768
SF 125.7333810 MHz
WDW EM
SSB 0
LB 1.00 Hz
GB 0
PC 1.40

10-Fluoro-4*H*-indolo[3,2,1-*de*][1,5]naphthyridin-4-one (**8k**), ^{1}H NMR in $CDCl_3$, 500 MHz

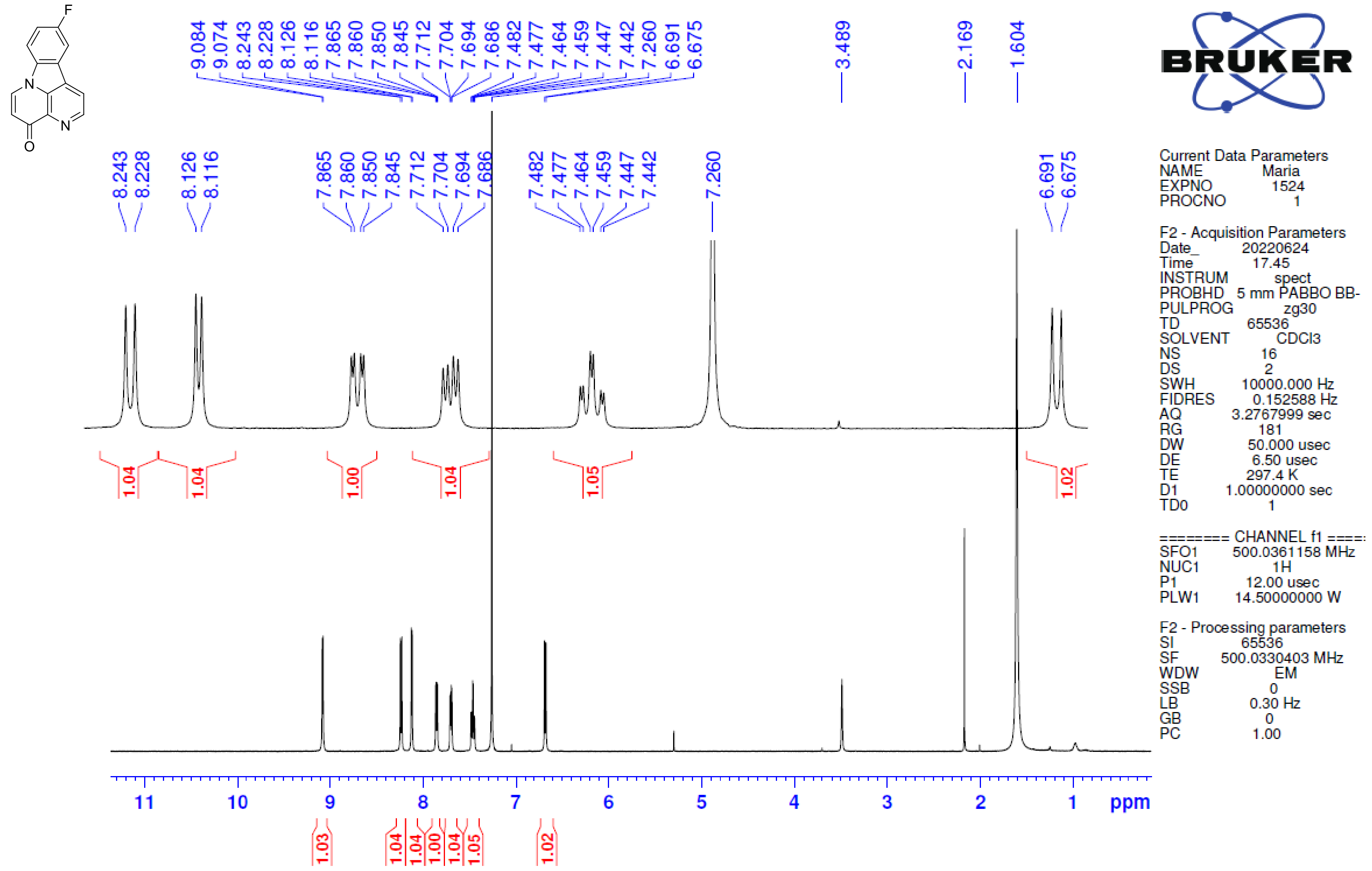

10-Fluoro-4*H*-indolo[3,2,1-*de*][1,5]naphthyridin-4-one (**8k**), ^{13}C NMR in $CDCl_3$, 125 MHz

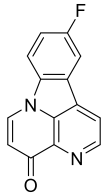

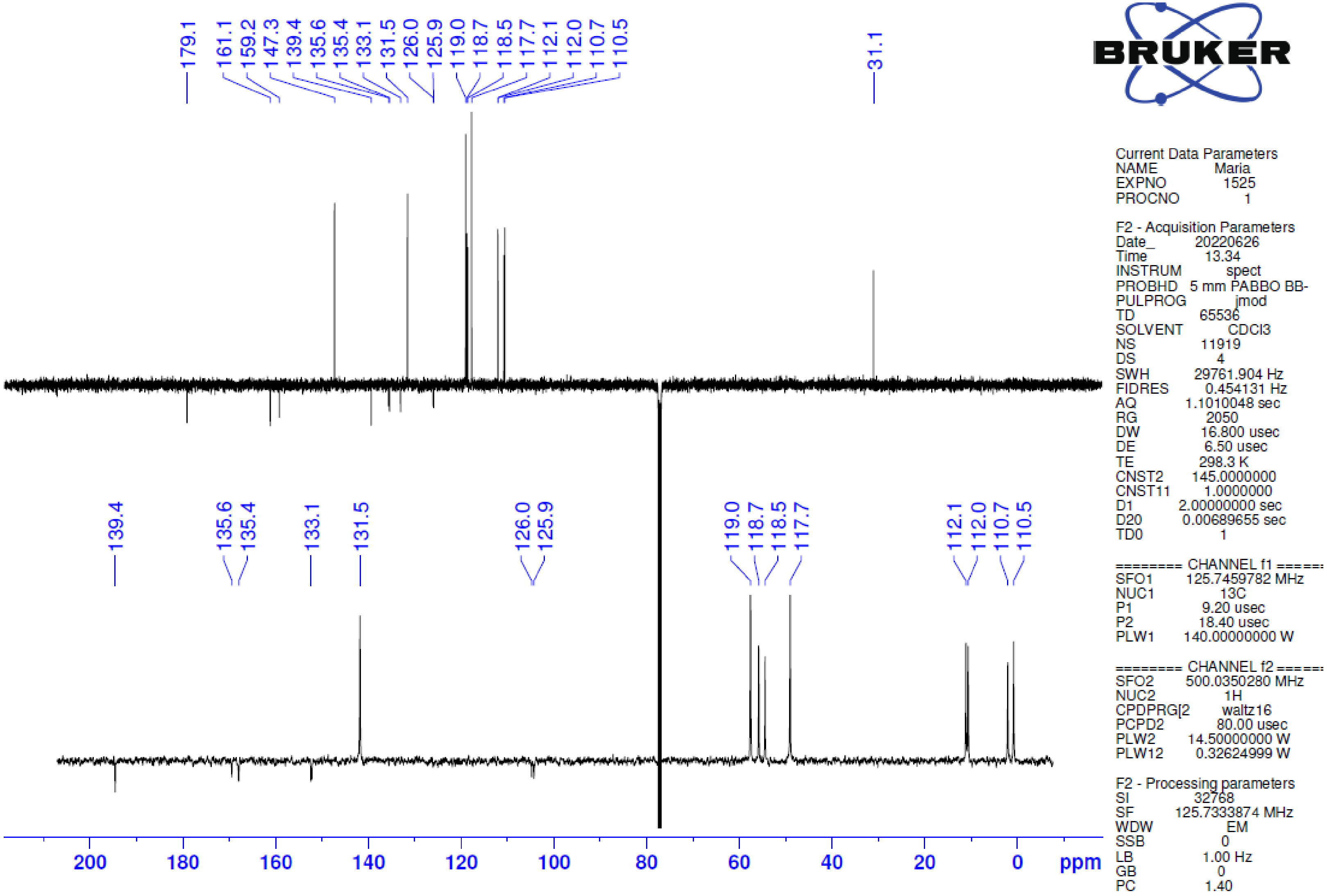

4*H*-Indolo[3,2,1-*ij*][1,6]naphthyridin-4-one (**9a**), ^{1}H NMR in $CDCl_3$, 500 MHz

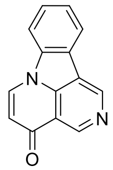

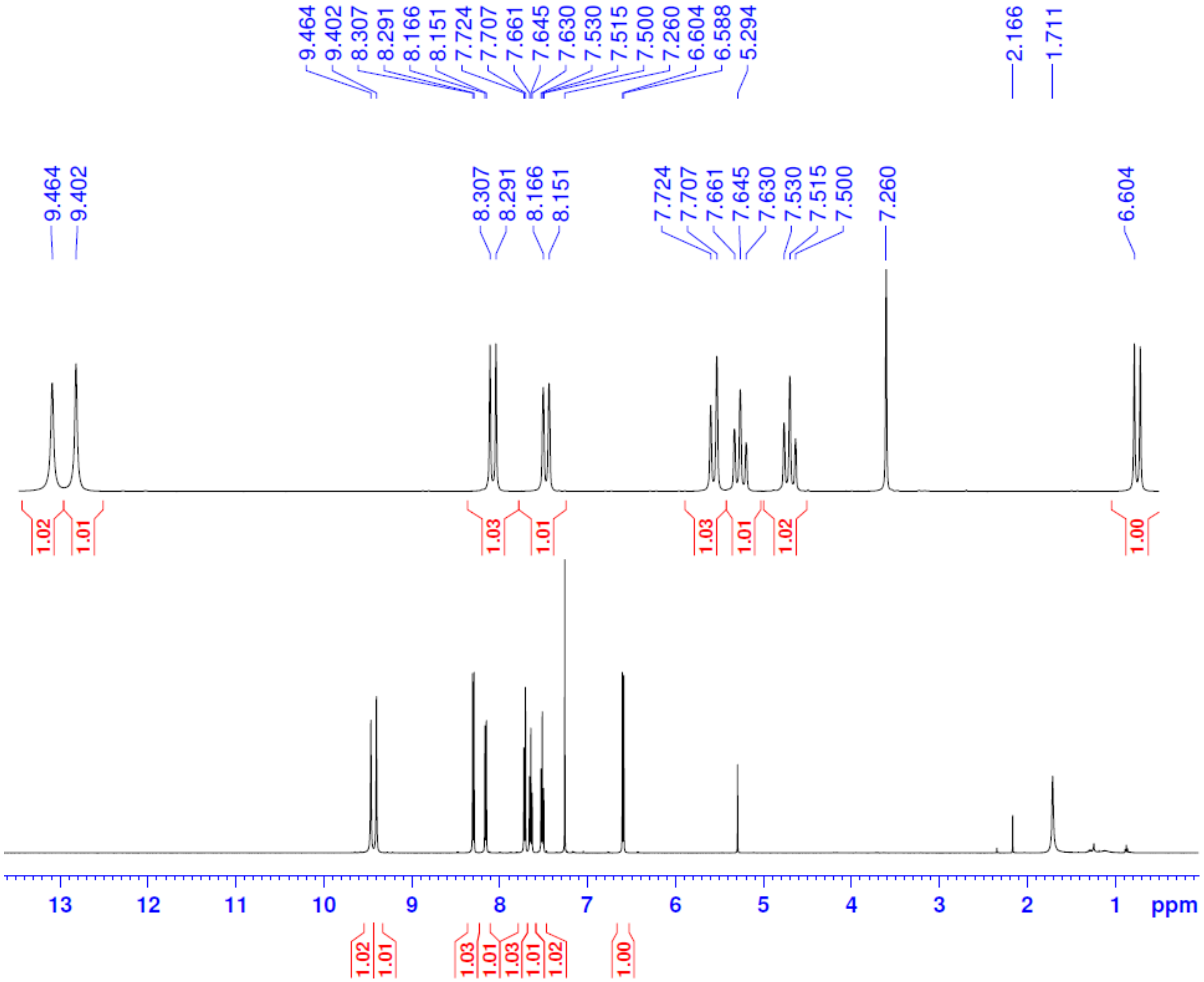

Current Data Parameters
NAME Maria
EXPNO 991
PROCNO 1

F2 - Acquisition Parameters
Date_ 20210325
Time 5.21
INSTRUM spect
PROBHD 5 mm PABBO BB-
PULPROG zg30
TD 65536
SOLVENT CDCl3
NS 16
DS 2
SWH 10000.000 Hz
FIDRES 0.152588 Hz
AQ 3.2767999 sec
RG 161
DW 50.000 usec
DE 6.50 usec
TE 296.6 K
D1 1.00000000 sec
TD0 1

======== CHANNEL f1 ====
SFO1 500.0361158 MHz
NUC1 1H
P1 12.00 usec
PLW1 14.50000000 W

F2 - Processing parameters
SI 65536
SF 500.0330404 MHz
WDW EM
SSB 0
LB 0.30 Hz
GB 0
PC 1.00

4*H*-Indolo[3,2,1-*ij*][1,6]naphthyridin-4-one (**9a**), ^{13}C NMR in $CDCl_3$, 125 MHz

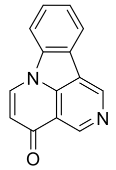

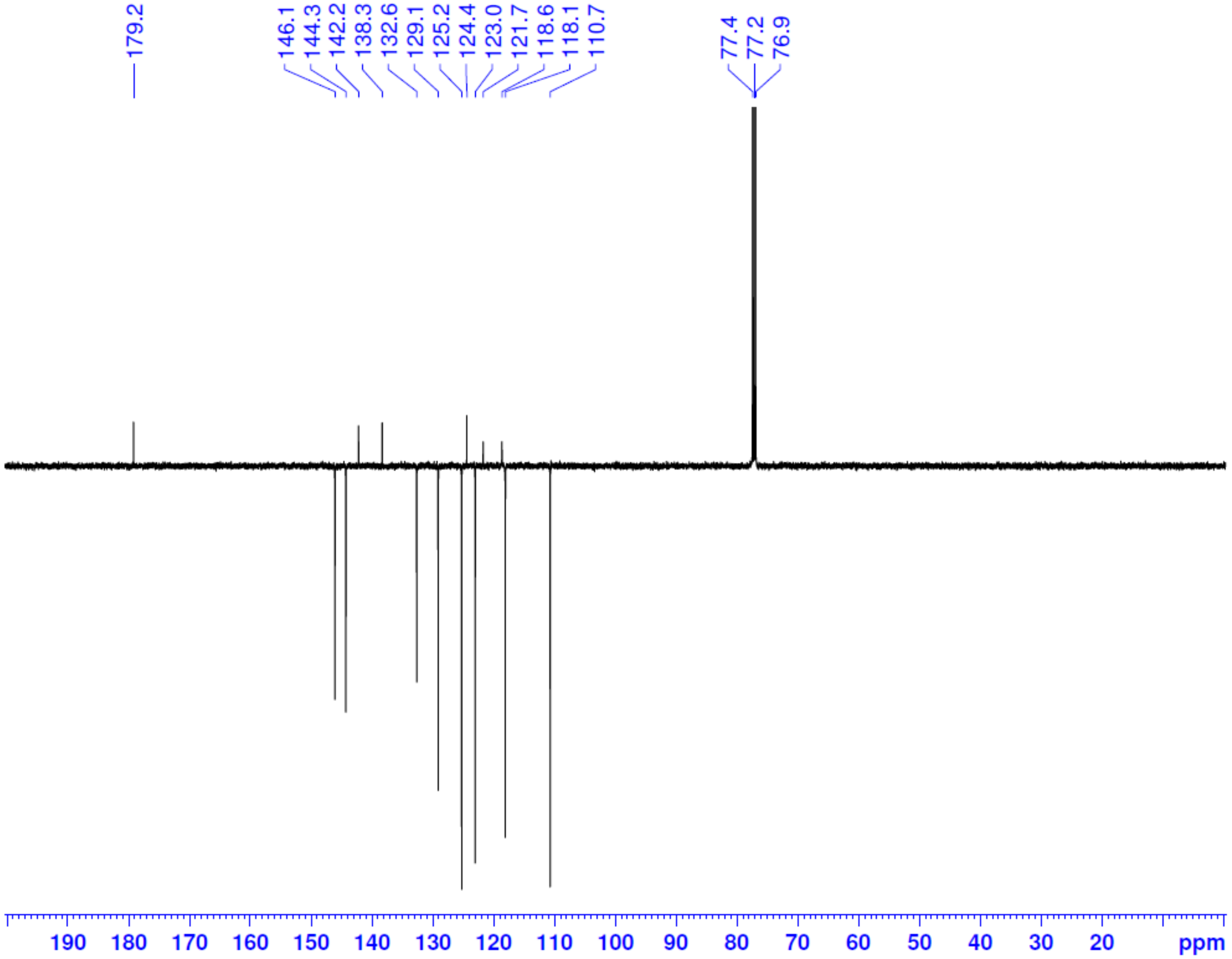

Current Data Parameters
NAME Maria
EXPNO 992
PROCNO 1

F2 - Acquisition Parameters
Date_ 20210325
Time 8.58
INSTRUM spect
PROBHD 5 mm PABBO BB-
PULPROG jmod
TD 65536
SOLVENT CDCl3
NS 4096
DS 4
SWH 29761.904 Hz
FIDRES 0.454131 Hz
AQ 1.1010048 sec
RG 2050
DW 16.800 usec
DE 6.50 usec
TE 297.6 K
CNST2 145.0000000
CNST11 1.0000000
D1 2.00000000 sec
D20 0.00689655 sec
TD0 1

======== CHANNEL f1 ========
SFO1 125.7459782 MHz
NUC1 13C
P1 9.20 usec
P2 18.40 usec
PLW1 140.00000000 W

======== CHANNEL f2 ========
SFO2 500.0350280 MHz
NUC2 1H
CPDPRG[2 waltz16
PCPD2 80.00 usec
PLW2 14.50000000 W
PLW12 0.32624999 W

F2 - Processing parameters
SI 32768
SF 125.7333892 MHz
WDW EM
SSB 0
LB 1.00 Hz
GB 0
PC 1.40

9-Methyl-4*H*-indolo[3,2,1-*ij*][1,6]naphthyridin-4-one (**9b**), ^{1}H NMR in $CDCl_3$, 500 MHz

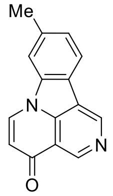

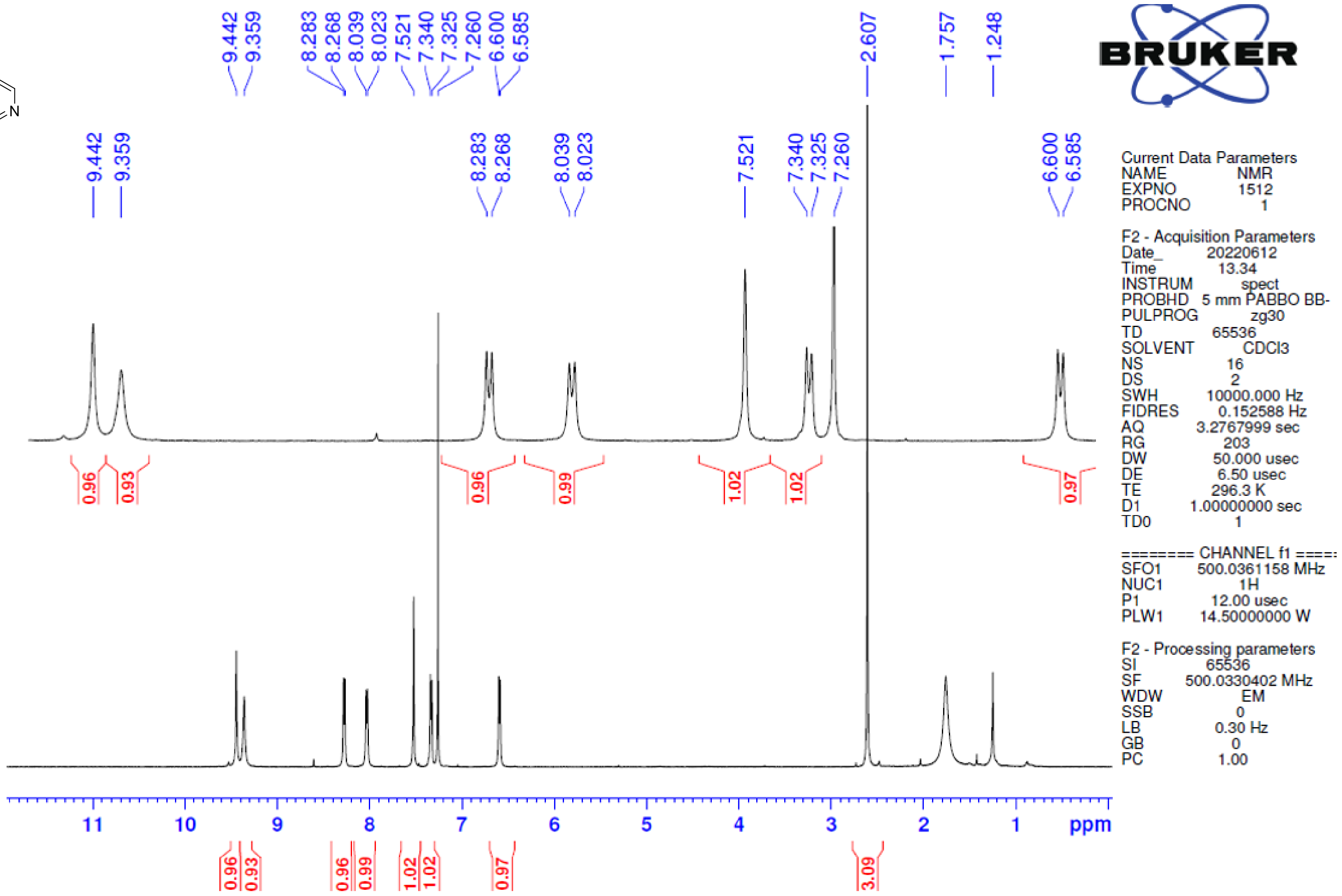

9-Methyl-4*H*-indolo[3,2,1-*ij*][1,6]naphthyridin-4-one (**9b**), ^{13}C NMR in $CDCl_3$, 125 MHz

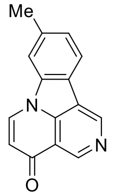

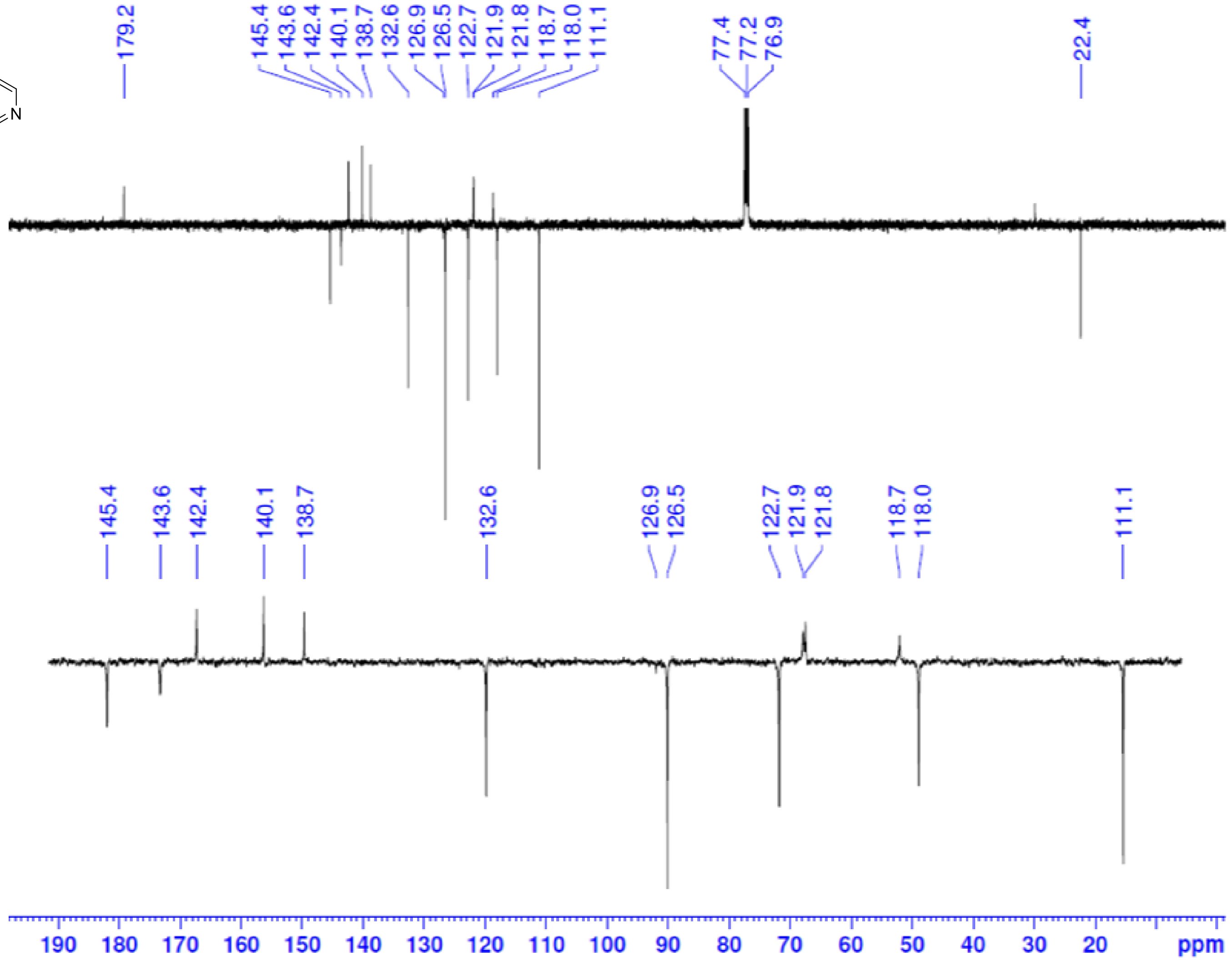

Current Data Parameters
NAME NMR
EXPNO 1513
PROCNO 1

F2 - Acquisition Parameters
Date_ 20220612
Time 14.23
INSTRUM spect
PROBHD 5 mm PABBO BB-
PULPROG jmod
TD 65536
SOLVENT CDCl3
NS 10240
DS 4
SWH 29761.904 Hz
FIDRES 0.454131 Hz
AQ 1.1010048 sec
RG 2050
DW 16.800 usec
DE 6.50 usec
TE 297.5 K
CNST2 145.0000000
CNST11 1.0000000
D1 2.00000000 sec
D20 0.00689655 sec
TD0 1

======== CHANNEL f1 ========
SFO1 125.7459782 MHz
NUC1 13C
P1 9.20 usec
P2 18.40 usec
PLW1 140.00000000 W

======== CHANNEL f2 ========
SFO2 500.0350280 MHz
NUC2 1H
CPDPRG[2 waltz16
PCPD2 80.00 usec
PLW2 14.50000000 W
PLW12 0.32624999 W

F2 - Processing parameters
SI 32768
SF 125.7333879 MHz
WDW EM
SSB 0
LB 1.00 Hz
GB 0
PC 1.40

9-Methoxy-4*H*-indolo[3,2,1-*ij*][1,6]naphthyridin-4-one (**9c**), ^{1}H NMR in DMSO-d_6, 500 MHz

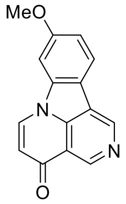

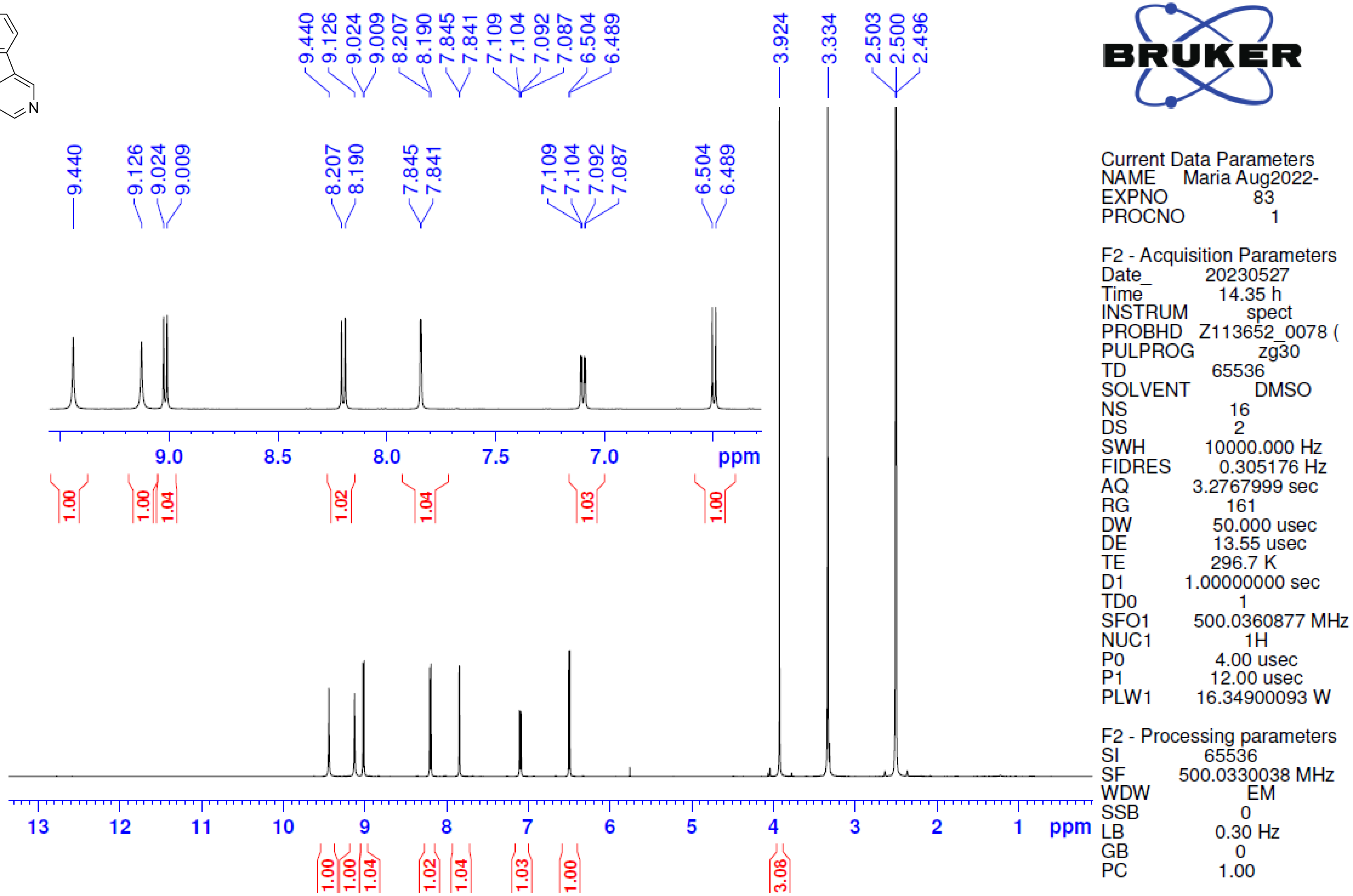

9-Methoxy-4*H*-indolo[3,2,1-*ij*][1,6]naphthyridin-4-one (**9c**), ^{13}C NMR in DMSO-d_6, 125 MHz

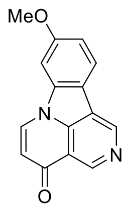

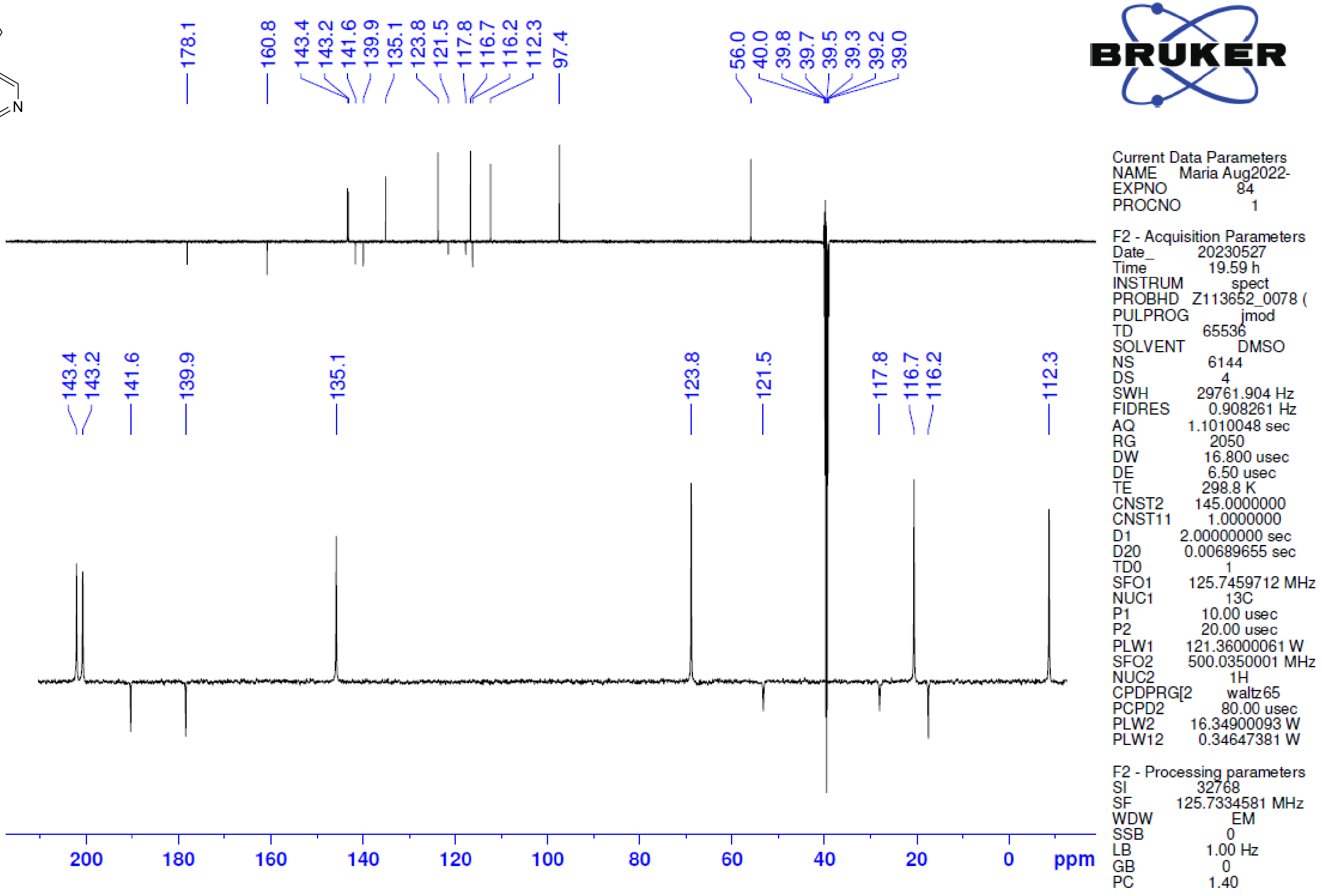

9-(Trifluoromethyl)-4*H*-indolo[3,2,1-*ij*][1,6]naphthyridin-4-one (**9d**), ^{1}H NMR in $CDCl_3$, 500 MHz

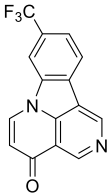

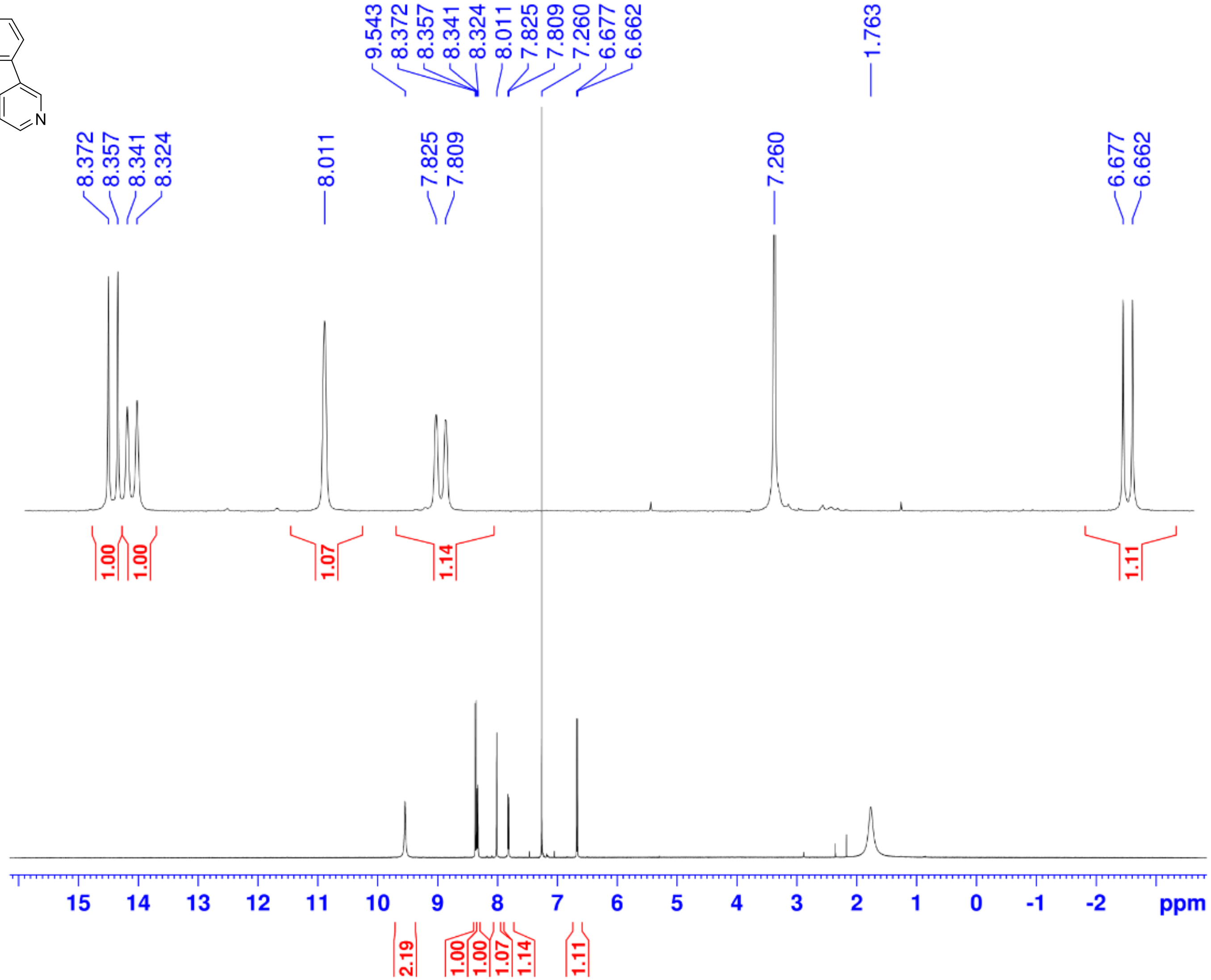

9-(Trifluoromethyl)-4*H*-indolo[3,2,1-*ij*][1,6]naphthyridin-4-one (**9d**), ^{13}C NMR in $CDCl_3$, 125 MHz

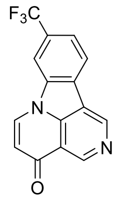

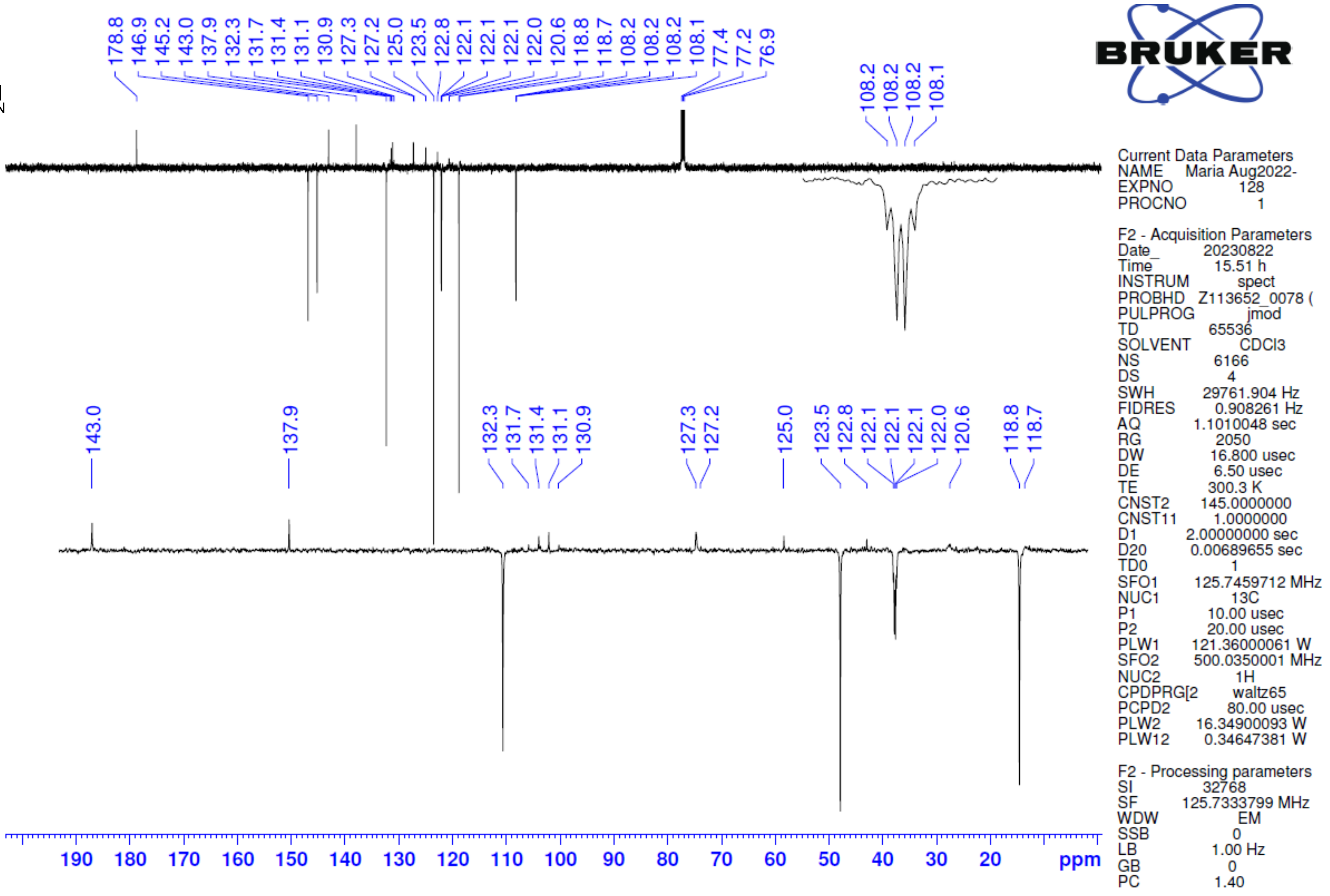

9-Fluoro-4H-indolo[3,2,1-*ij*][1,6]naphthyridin-4-one (**9e**), ^{1}H NMR in $CDCl_3$, 500 MHz

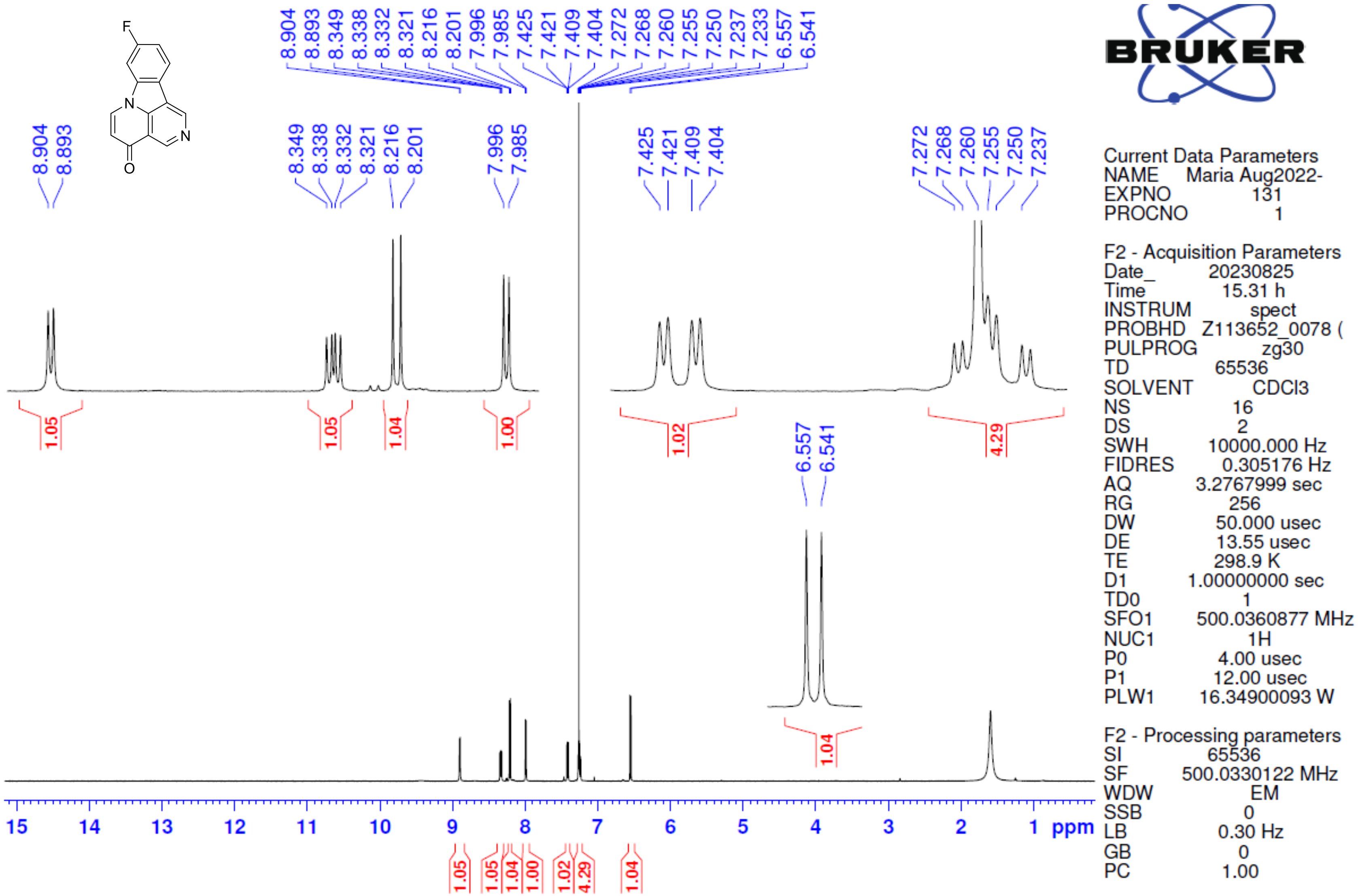

9-Fluoro-4*H*-indolo[3,2,1-*ij*][1,6]naphthyridin-4-one (**9e**), ^{13}C NMR in $CDCl_3$, 125 MHz

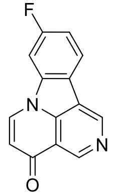

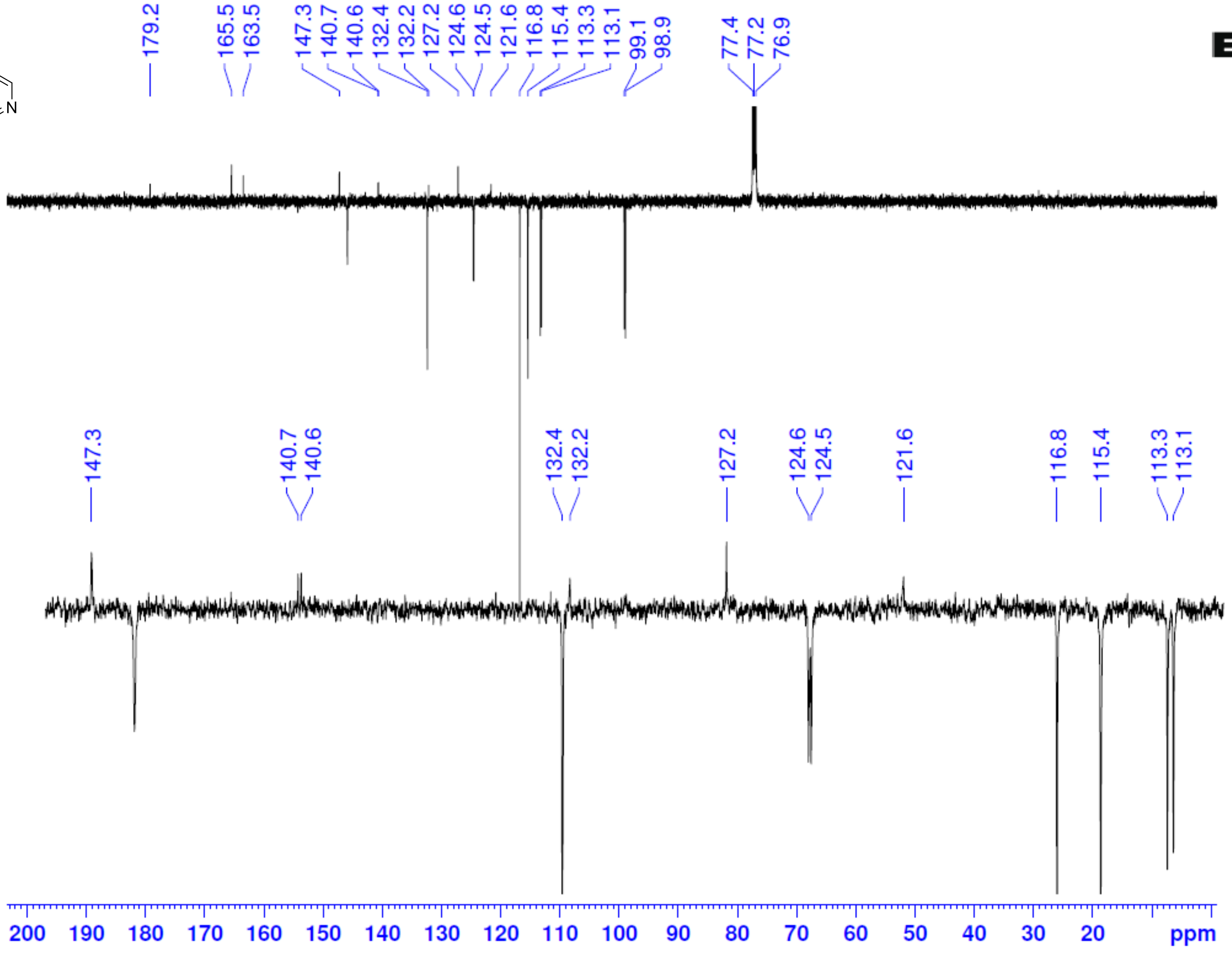

Current Data Parameters
NAME Maria Aug2022-
EXPNO 133
PROCNO 1

F2 - Acquisition Parameters
Date_ 20230827
Time 10.12 h
INSTRUM spect
PROBHD Z113652_0078 (
PULPROG jmod
TD 65536
SOLVENT CDCl3
NS 48691
DS 4
SWH 29761.904 Hz
FIDRES 0.908261 Hz
AQ 1.1010048 sec
RG 2050
DW 16.800 usec
DE 6.50 usec
TE 299.6 K
CNST2 145.0000000
CNST11 1.0000000
D1 2.00000000 sec
D20 0.00689655 sec
TD0 1
SFO1 125.7459712 MHz
NUC1 13C
P1 10.00 usec
P2 20.00 usec
PLW1 121.36000061 W
SFO2 500.0350001 MHz
NUC2 1H
CPDPRG[2 waltz65
PCPD2 80.00 usec
PLW2 16.34900093 W
PLW12 0.34647381 W

F2 - Processing parameters
SI 32768
SF 125.7333789 MHz
WDW EM
SSB 0
LB 1.00 Hz
GB 0
PC 1.40

10-Methoxy-4*H*-indolo[3,2,1-*ij*][1,6]naphthyridin-4-one (**9f**), ^{1}H NMR in $CDCl_3$, 500 MHz

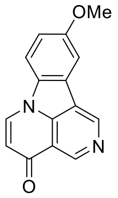

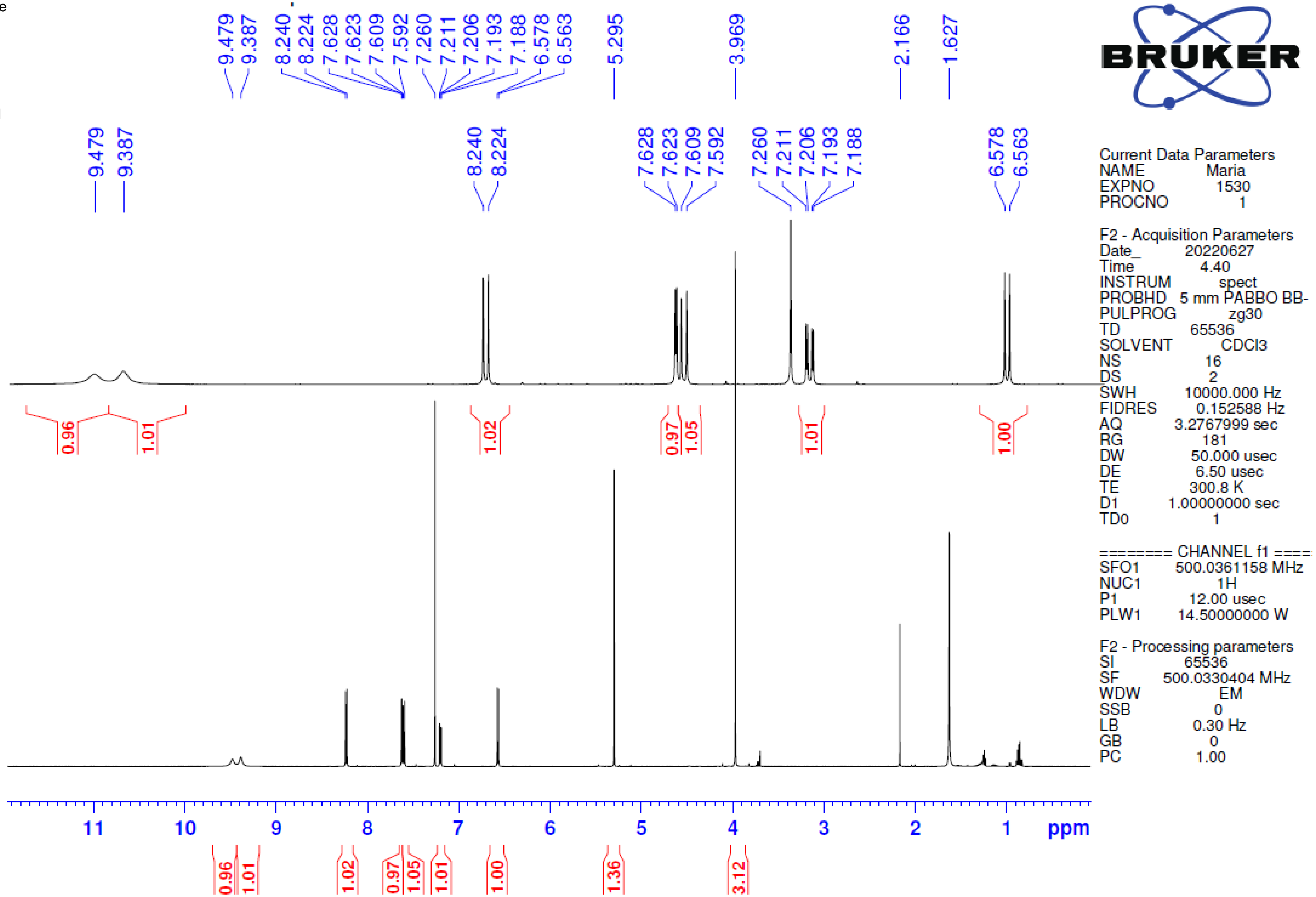

10-Methoxy-4*H*-indolo[3,2,1-*ij*][1,6]naphthyridin-4-one (**9f**), ^{13}C NMR in $CDCl_3$, 125 MHz

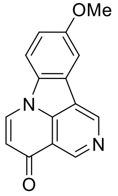

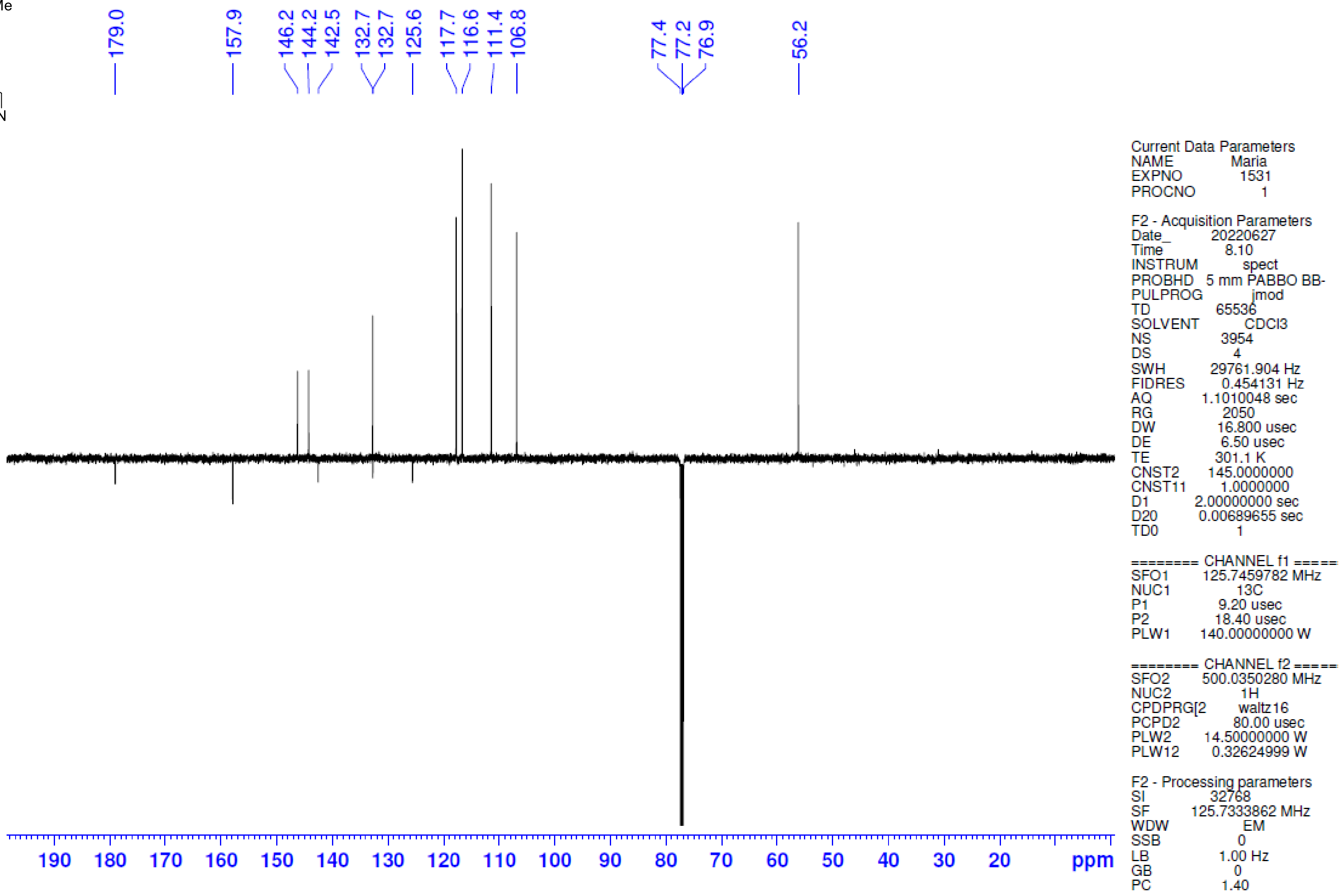

10-(Trifluoromethoxy)-4*H*-indolo[3,2,1-*ij*][1,6]naphthyridin-4-one (**9g**), ^{1}H NMR in $CDCl_3$, 500 MHz

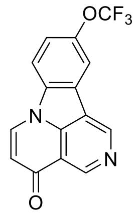

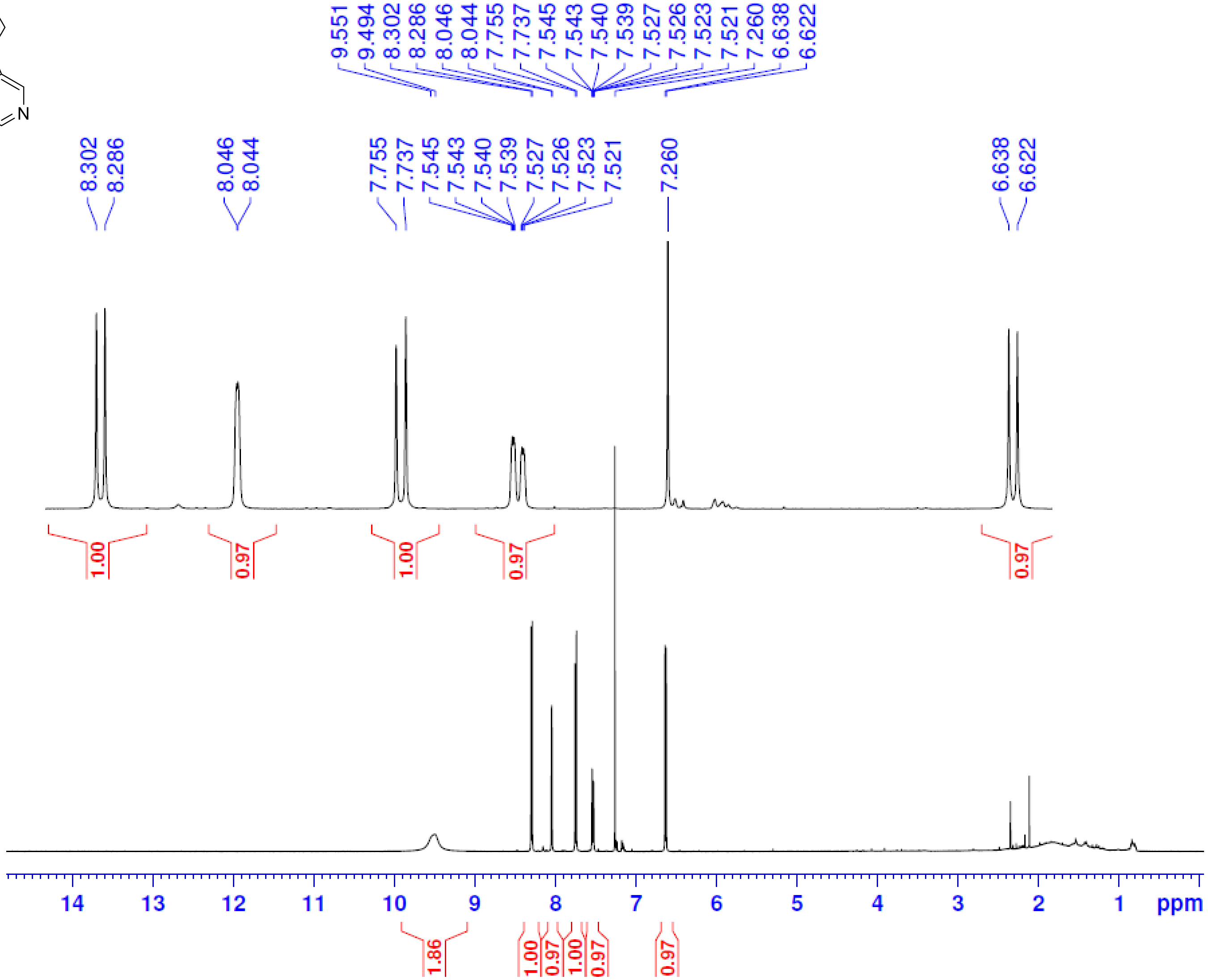

Current Data Parameters
NAME Maria Aug2022-
EXPNO 90
PROCNO 1

F2 - Acquisition Parameters
Date_ 20230611
Time 11.47 h
INSTRUM spect
PROBHD Z113652_0078 (
PULPROG zg30
TD 65536
SOLVENT CDCl3
NS 16
DS 2
SWH 10000.000 Hz
FIDRES 0.305176 Hz
AQ 3.2767999 sec
RG 203
DW 50.000 usec
DE 13.55 usec
TE 298.8 K
D1 1.00000000 sec
TD0 1
SFO1 500.0360877 MHz
NUC1 1H
P0 4.00 usec
P1 12.00 usec
PLW1 16.34900093 W

F2 - Processing parameters
SI 65536
SF 500.0330123 MHz
WDW EM
SSB 0
LB 0.30 Hz
GB 0
PC 1.00

10-(Trifluoromethoxy)-4*H*-indolo[3,2,1-*ij*][1,6]naphthyridin-4-one (**9g**), ^{13}C NMR in $CDCl_3$, 125 MHz

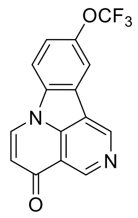

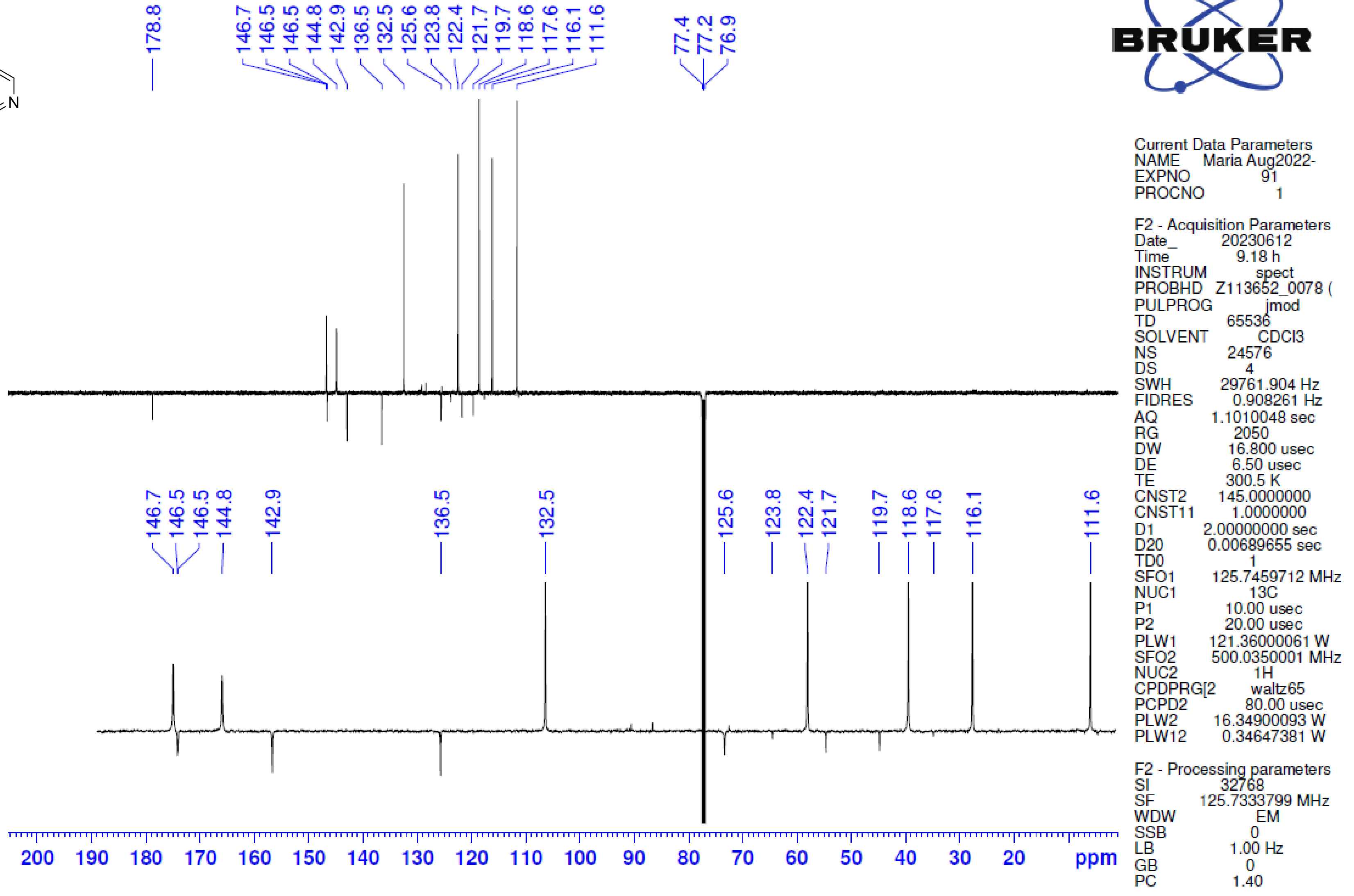

10-Fluoro-4*H*-indolo[3,2,1-*ij*][1,6]naphthyridin-4-one (**9h**), ^{1}H NMR in DMSO-d_6, 500 MHz

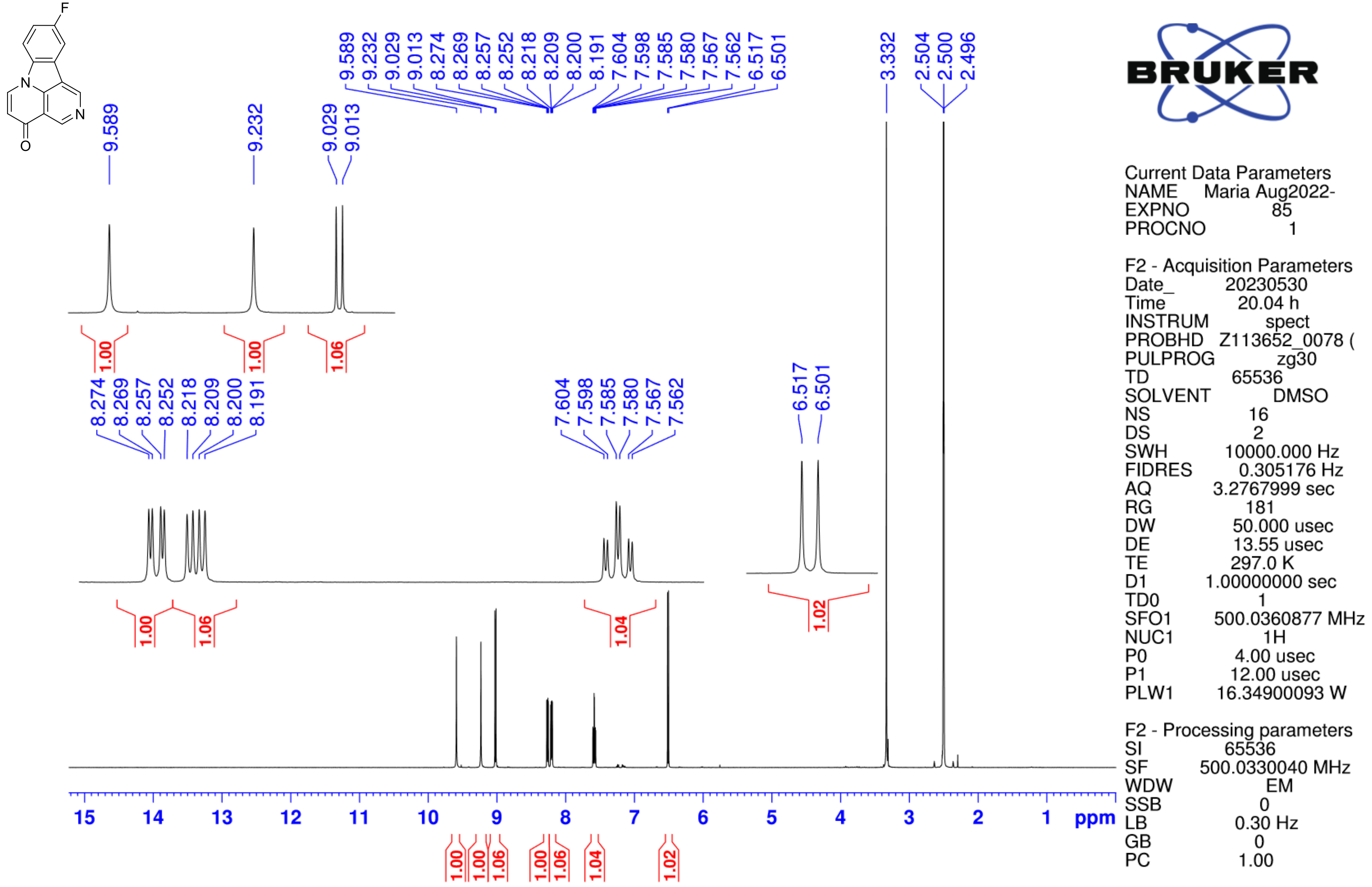

10-Fluoro-4*H*-indolo[3,2,1-*ij*][1,6]naphthyridin-4-one (**9h**), ^{13}C NMR in DMSO-d_6, 125 MHz

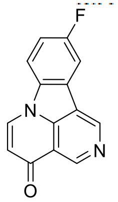

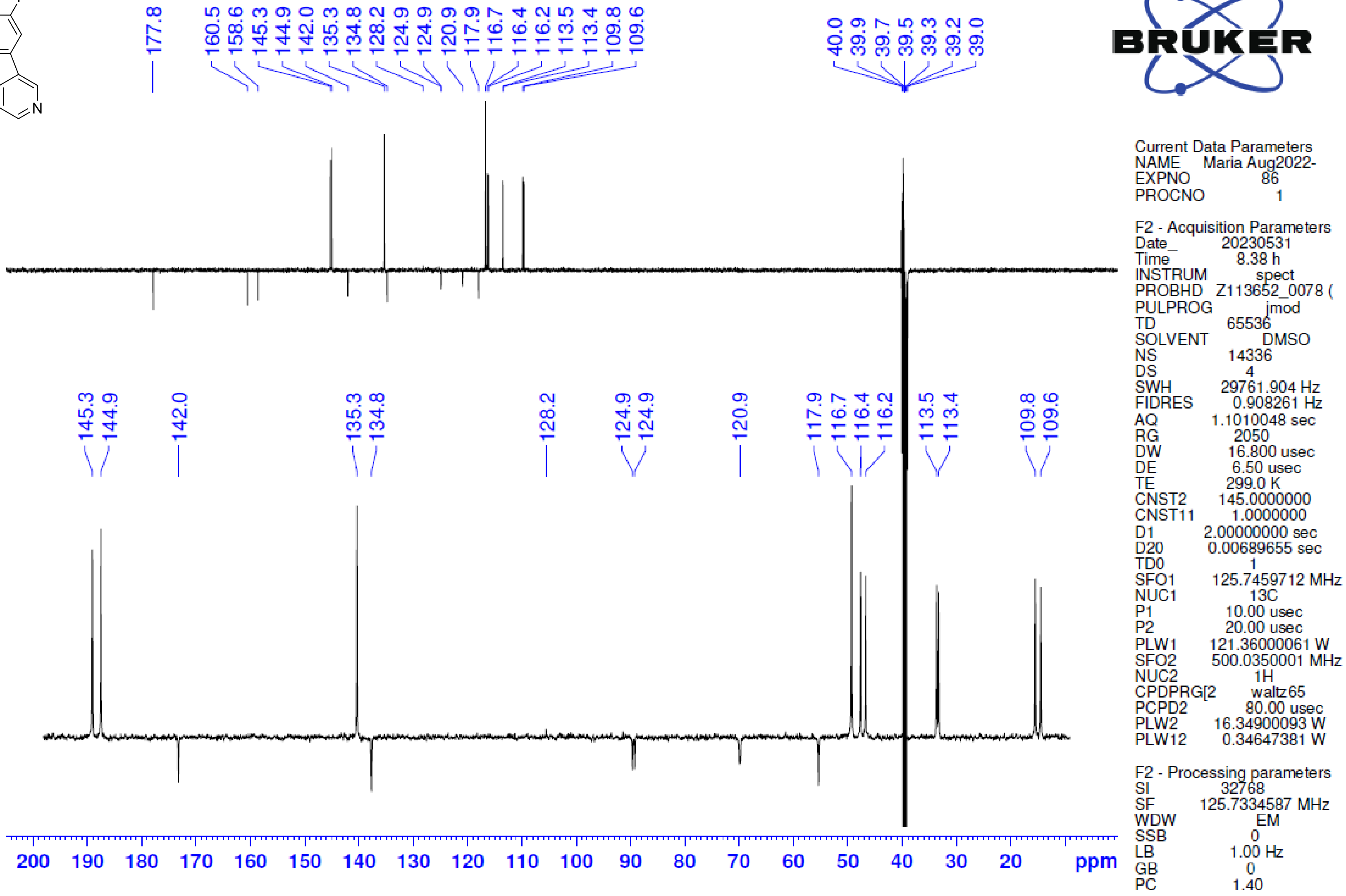

4*H*-Indolo[3,2,1-*ij*][1,7]naphthyridin-4-one (**10a**), ^{1}H NMR in $CDCl_3$, 500 MHz

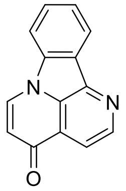

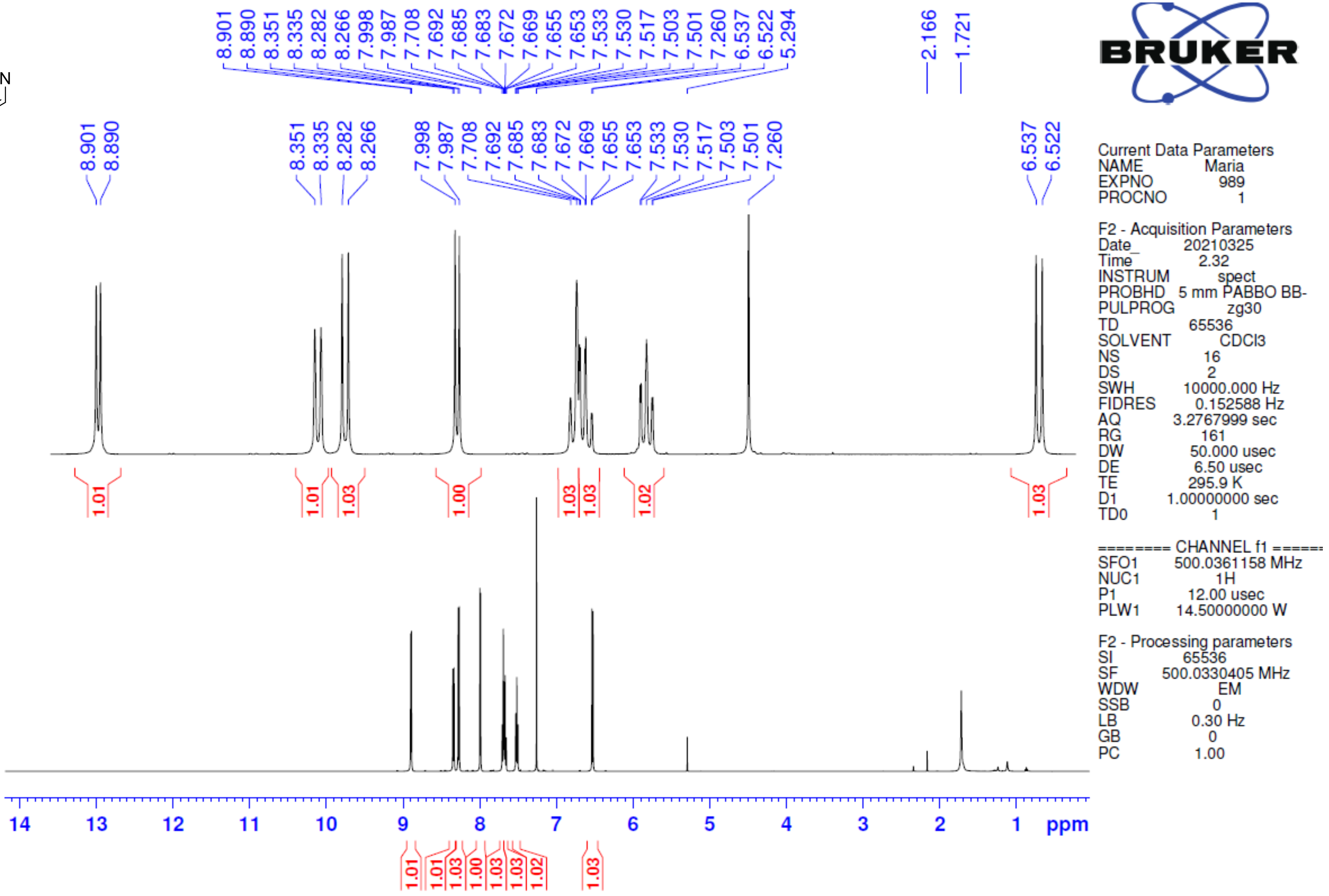

4*H*-Indolo[3,2,1-*ij*][1,7]naphthyridin-4-one (**10a**), ^{13}C NMR in $CDCl_3$, 125 MHz

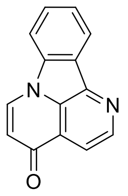

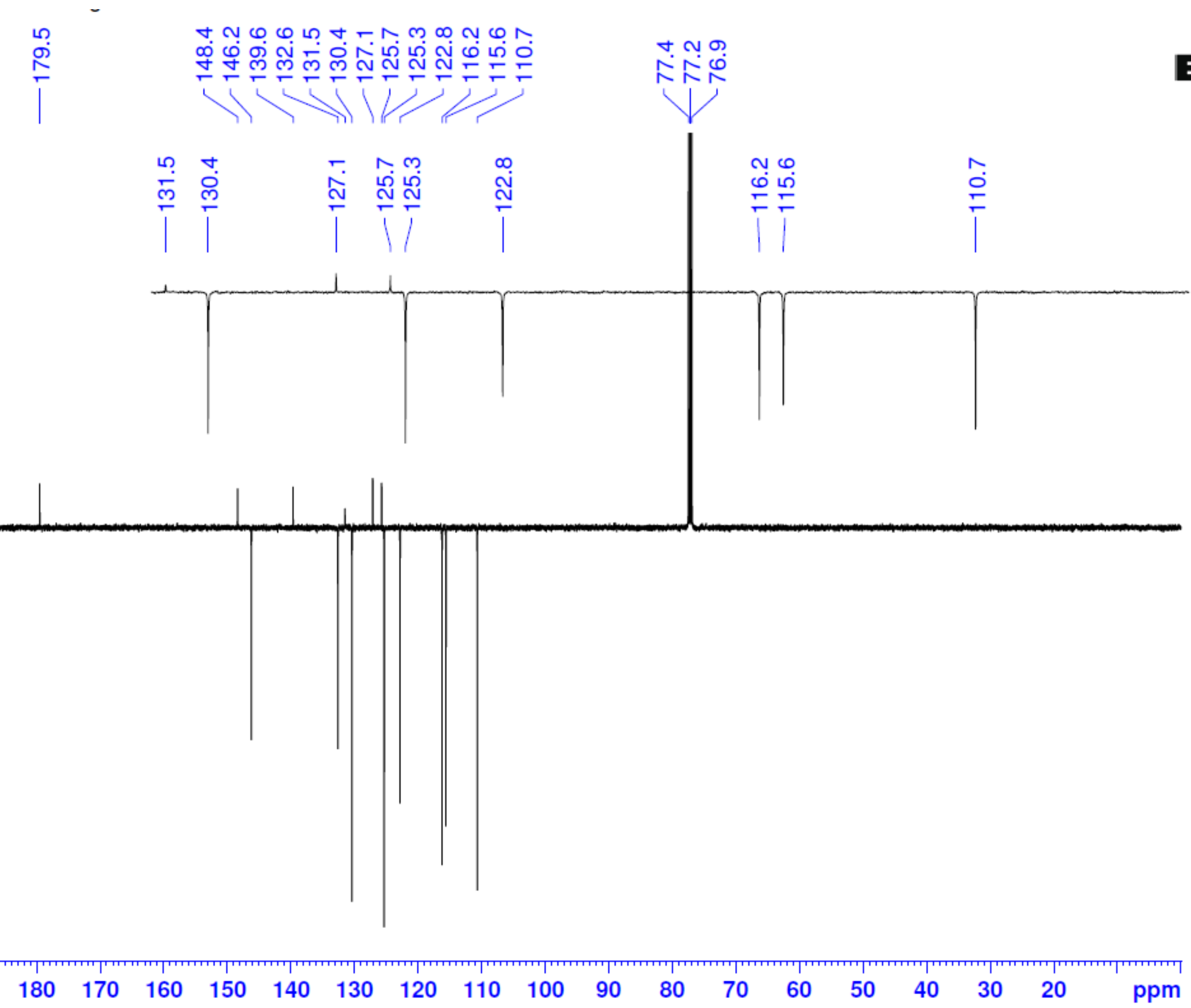

Current Data Parameters
NAME Maria
EXPNO 990
PROCNO 1

F2 - Acquisition Parameters
Date_ 20210325
Time 5.16
INSTRUM spect
PROBHD 5 mm PABBO BB-
PULPROG jmod
TD 65536
SOLVENT CDCl3
NS 3072
DS 4
SWH 29761.904 Hz
FIDRES 0.454131 Hz
AQ 1.1010048 sec
RG 2050
DW 16.800 usec
DE 6.50 usec
TE 297.7 K
CNST2 145.0000000
CNST11 1.0000000
D1 2.00000000 sec
D20 0.00689655 sec
TD0 1

======== CHANNEL f1 ====
SFO1 125.7459782 MHz
NUC1 13C
P1 9.20 usec
P2 18.40 usec
PLW1 140.00000000 W

======== CHANNEL f2 ====
SFO2 500.0350280 MHz
NUC2 1H
CPDPRG[2 waltz16
PCPD2 80.00 usec
PLW2 14.50000000 W
PLW12 0.32624999 W

F2 - Processing parameters
SI 32768
SF 125.7333892 MHz
WDW EM
SSB 0
LB 1.00 Hz
GB 0
PC 1.40

8-Chloro-4*H*-indolo[3,2,1-*ij*][1,7]naphthyridin-4-one (**10b**), ^{1}H NMR in DMSO-d_6, 500 MHz

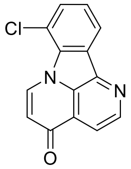

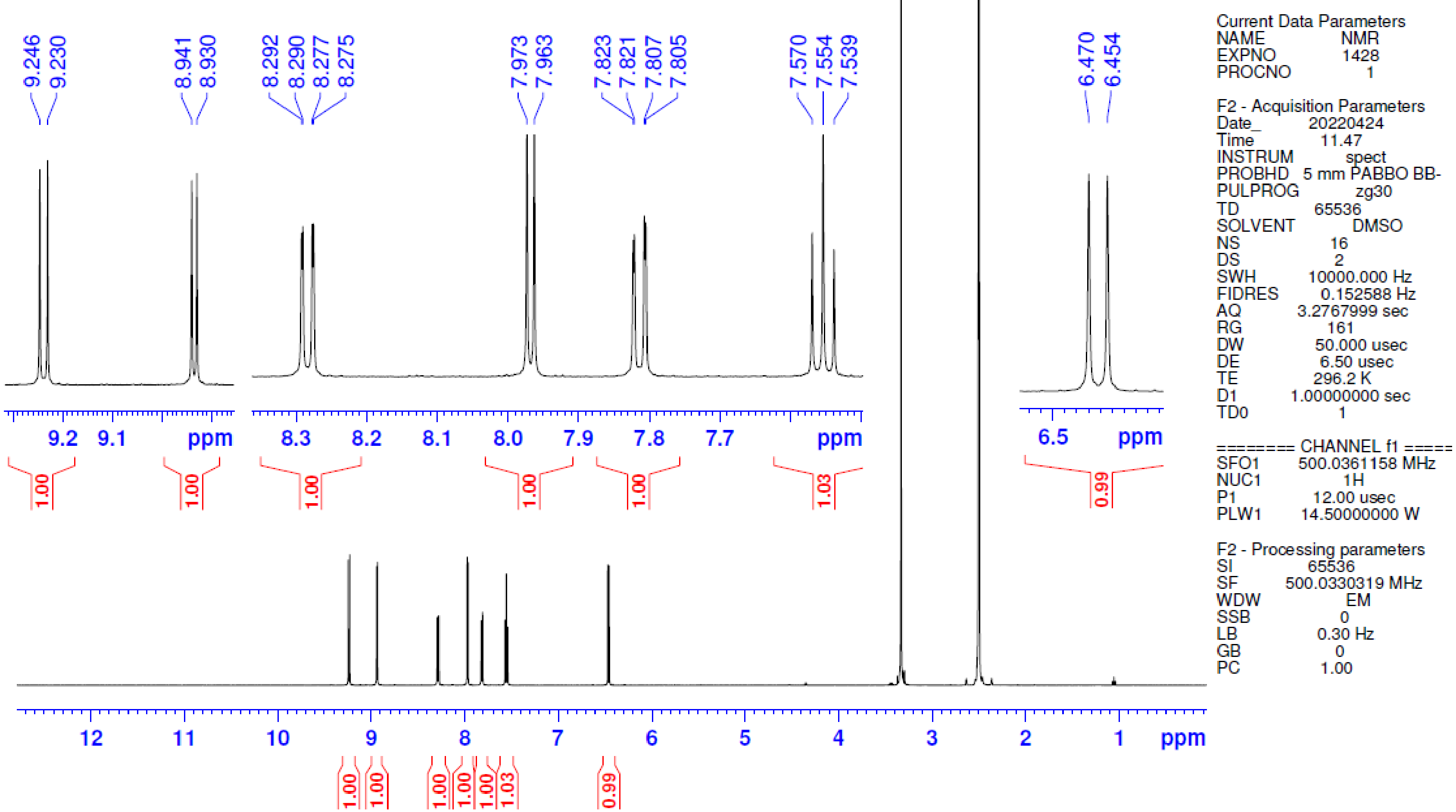

8-Chloro-4*H*-indolo[3,2,1-*ij*][1,7]naphthyridin-4-one (**10b**), ^{13}C NMR in DMSO-d_6, 125 MHz

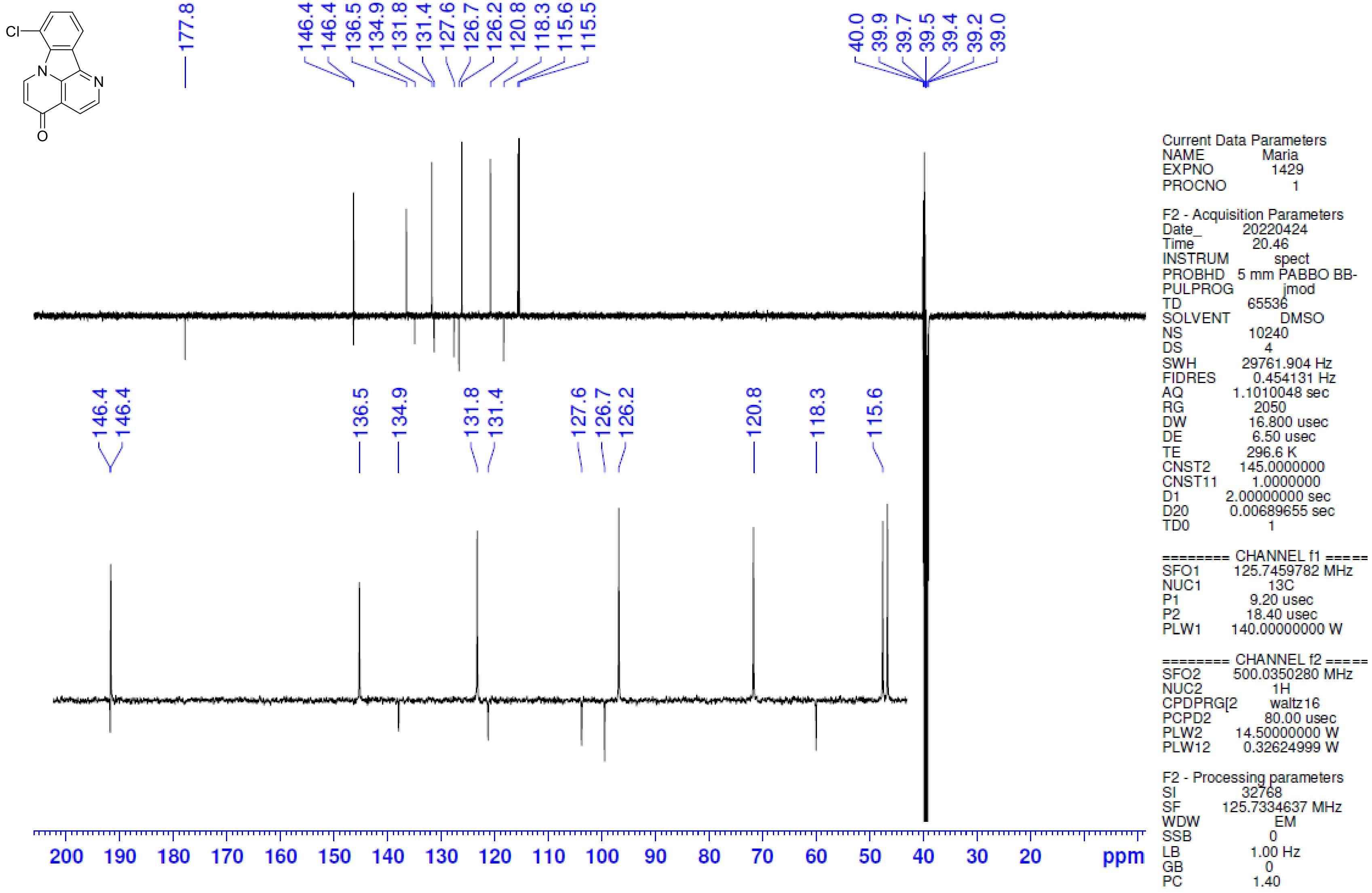

9-Methyl-4*H*-indolo[3,2,1-*ij*][1,7]naphthyridin-4-one (**10c**), ^{1}H NMR in $CDCl_3$, 500 MHz

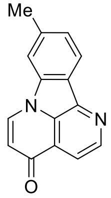

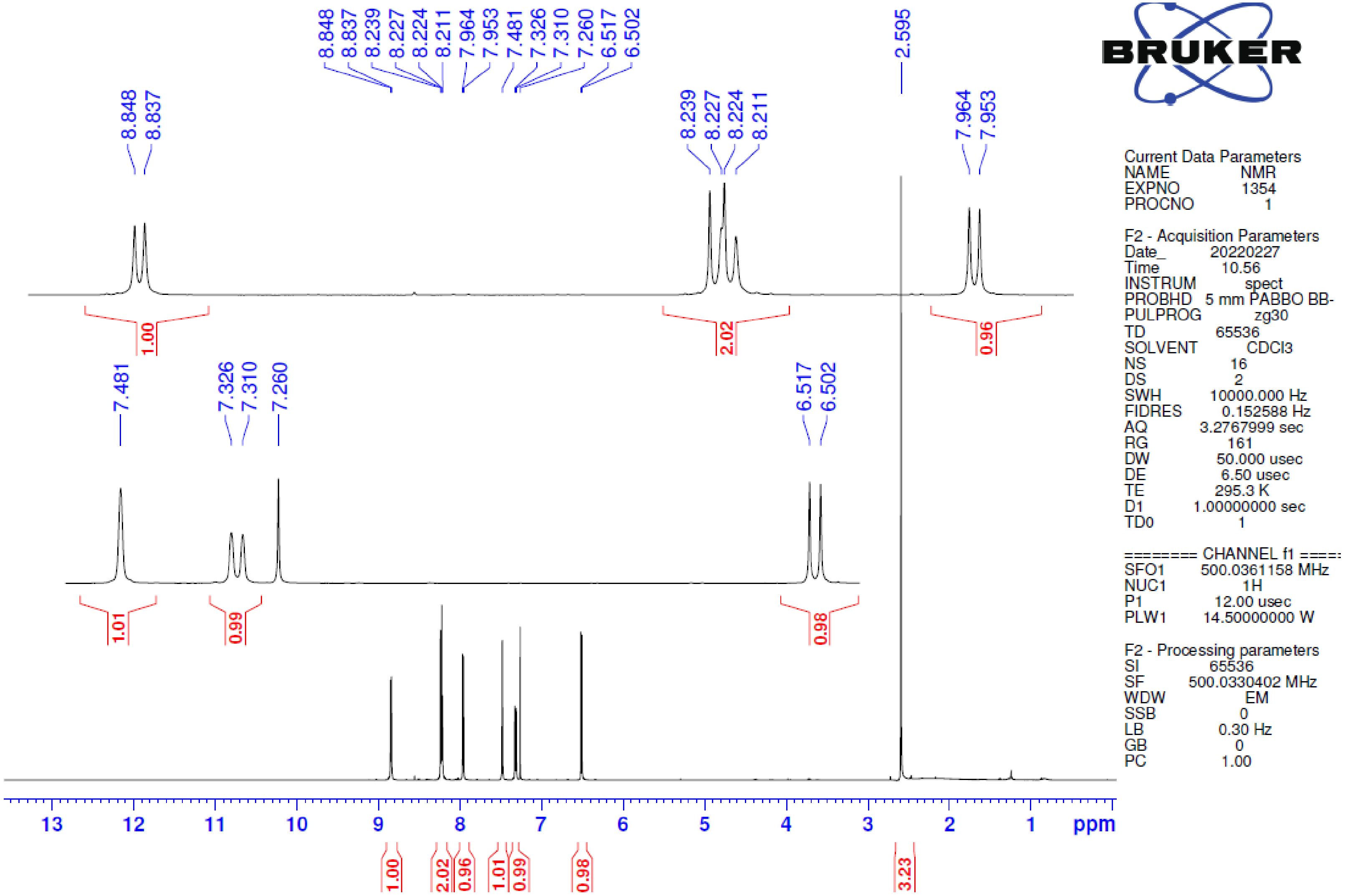

9-Methyl-4*H*-indolo[3,2,1-*ij*][1,7]naphthyridin-4-one (**10c**), ^{13}C NMR in $CDCl_3$, 500 MHz

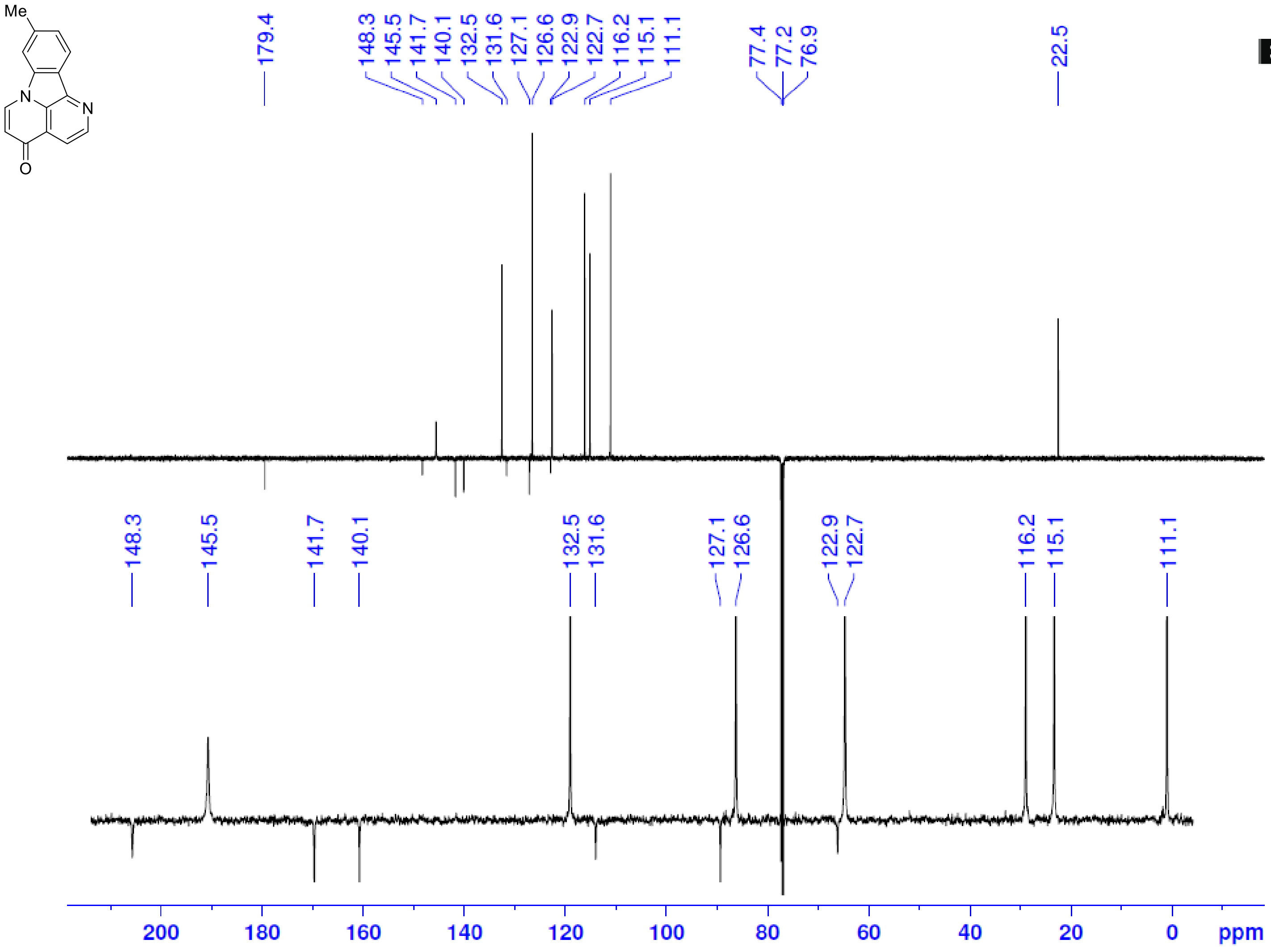

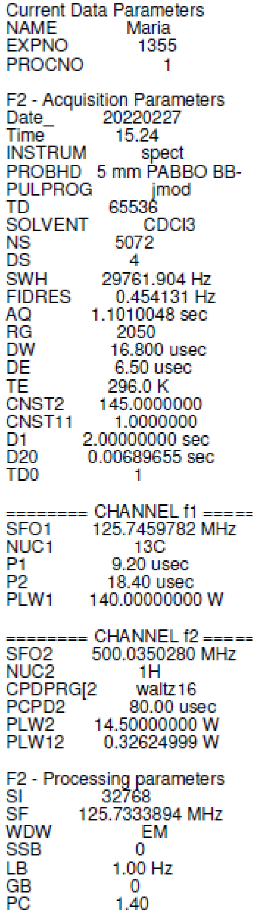

9-Methoxy-4*H*-indolo[3,2,1-*ij*][1,7]naphthyridin-4-one (**10d**), ^{1}H NMR in $CDCl_3$, 500 MHz

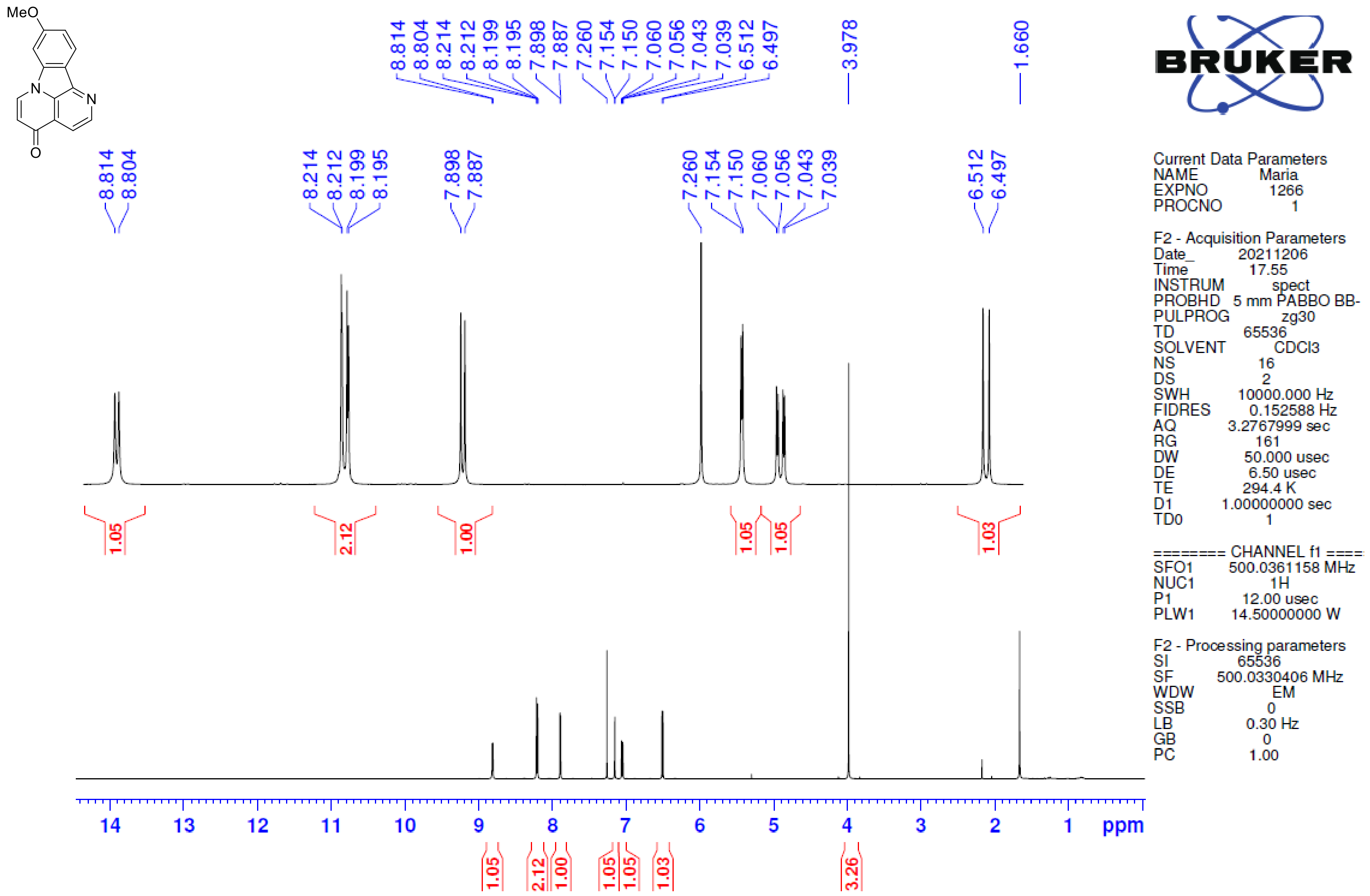

9-Methoxy-4*H*-indolo[3,2,1-*ij*][1,7]naphthyridin-4-one (**10d**), ^{13}C NMR in $CDCl_3$, 125 MHz

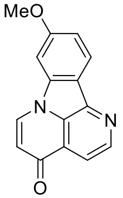

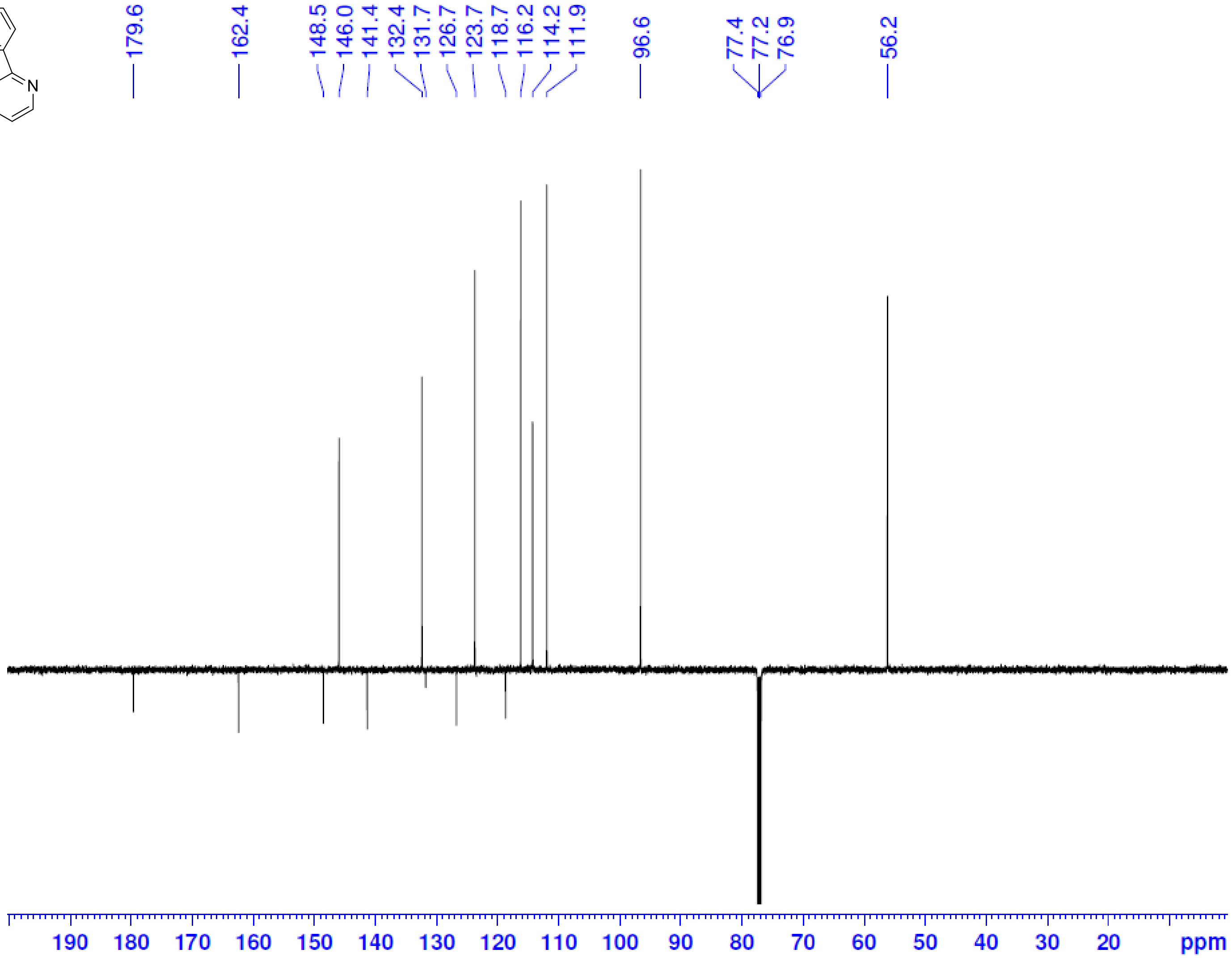

Current Data Parameters
NAME Maria
EXPNO 1268
PROCNO 1

F2 - Acquisition Parameters
Date_ 20211208
Time 2.19
INSTRUM spect
PROBHD 5 mm PABBO BB-
PULPROG jmod
TD 65536
SOLVENT CDCl3
NS 7168
DS 4
SWH 29761.904 Hz
FIDRES 0.454131 Hz
AQ 1.1010048 sec
RG 2050
DW 16.800 usec
DE 6.50 usec
TE 294.5 K
CNST2 145.0000000
CNST11 1.0000000
D1 2.00000000 sec
D20 0.00689655 sec
TD0 1

======== CHANNEL f1 ======
SFO1 125.7459782 MHz
NUC1 13C
P1 9.20 usec
P2 18.40 usec
PLW1 140.00000000 W

======== CHANNEL f2 ======
SFO2 500.0350280 MHz
NUC2 1H
CPDPRG[2 waltz16
PCPD2 80.00 usec
PLW2 14.50000000 W
PLW12 0.32624999 W

F2 - Processing parameters
SI 32768
SF 125.7333893 MHz
WDW EM
SSB 0
LB 1.00 Hz
GB 0
PC 1.40

9-(Trifluoromethyl)-4*H*-indolo[3,2,1-*ij*][1,7]naphthyridin-4-one (**10e**), ^{1}H NMR in DMSO-d_6, 500 MHz

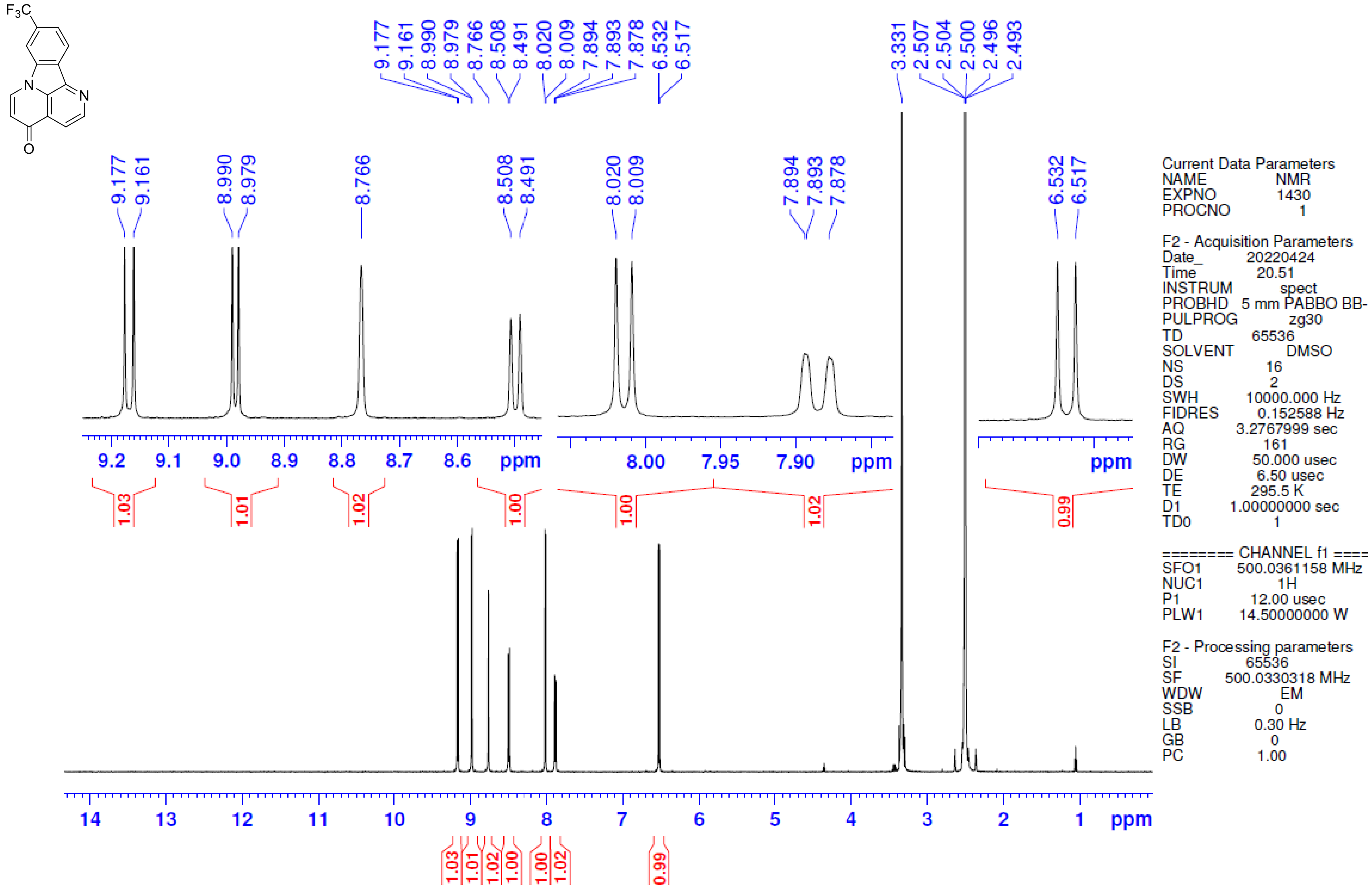

9-(Trifluoromethyl)-4*H*-indolo[3,2,1-*ij*][1,7]naphthyridin-4-one (**10e**), ^{13}C NMR in DMSO-d_6, 125 MHz

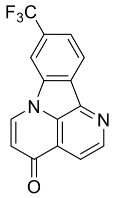

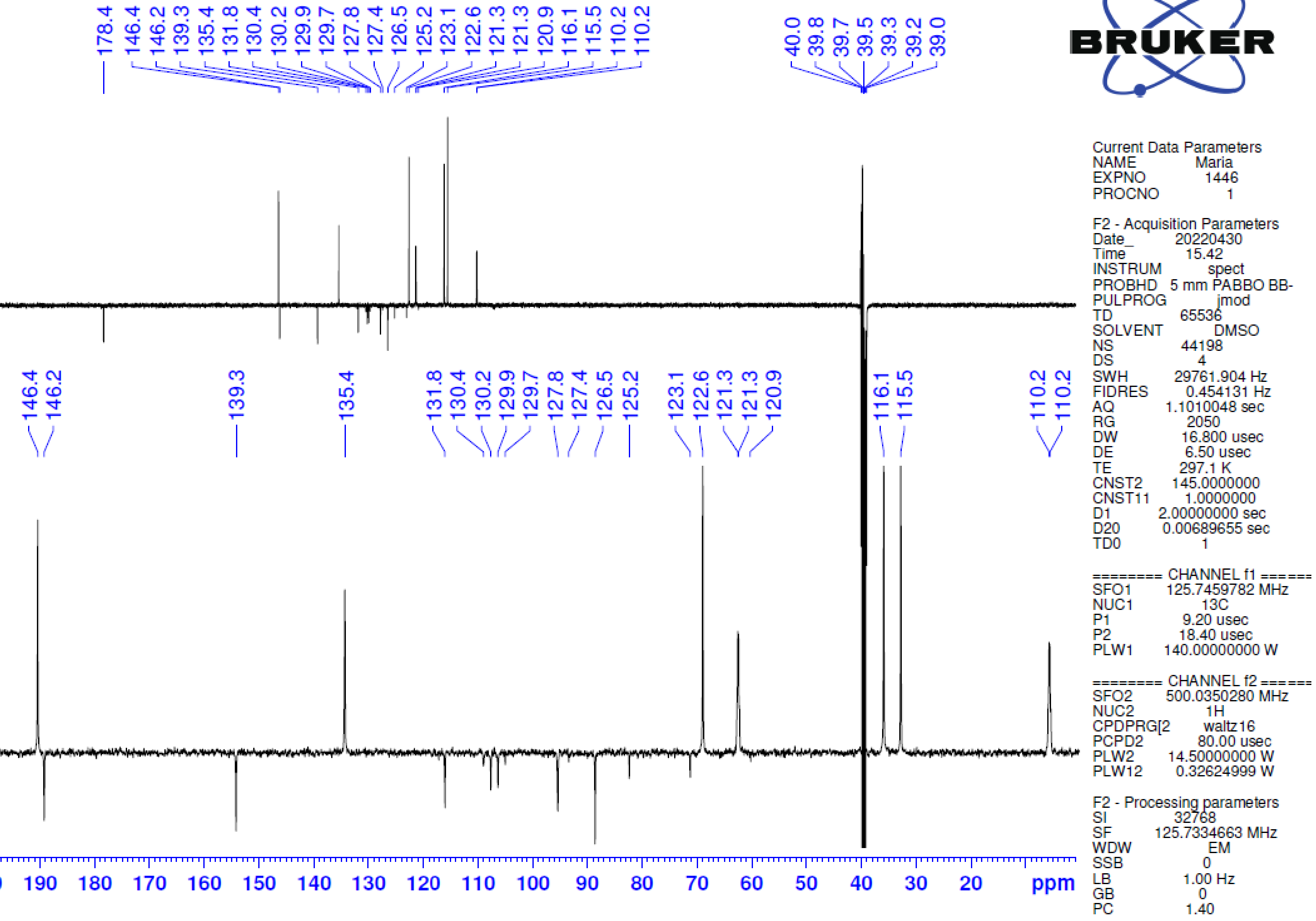

9-Chloro-4*H*-indolo[3,2,1-*ij*][1,7]naphthyridin-4-one (**10f**), ^{1}H NMR in DMSO-d_6, 500 MHz

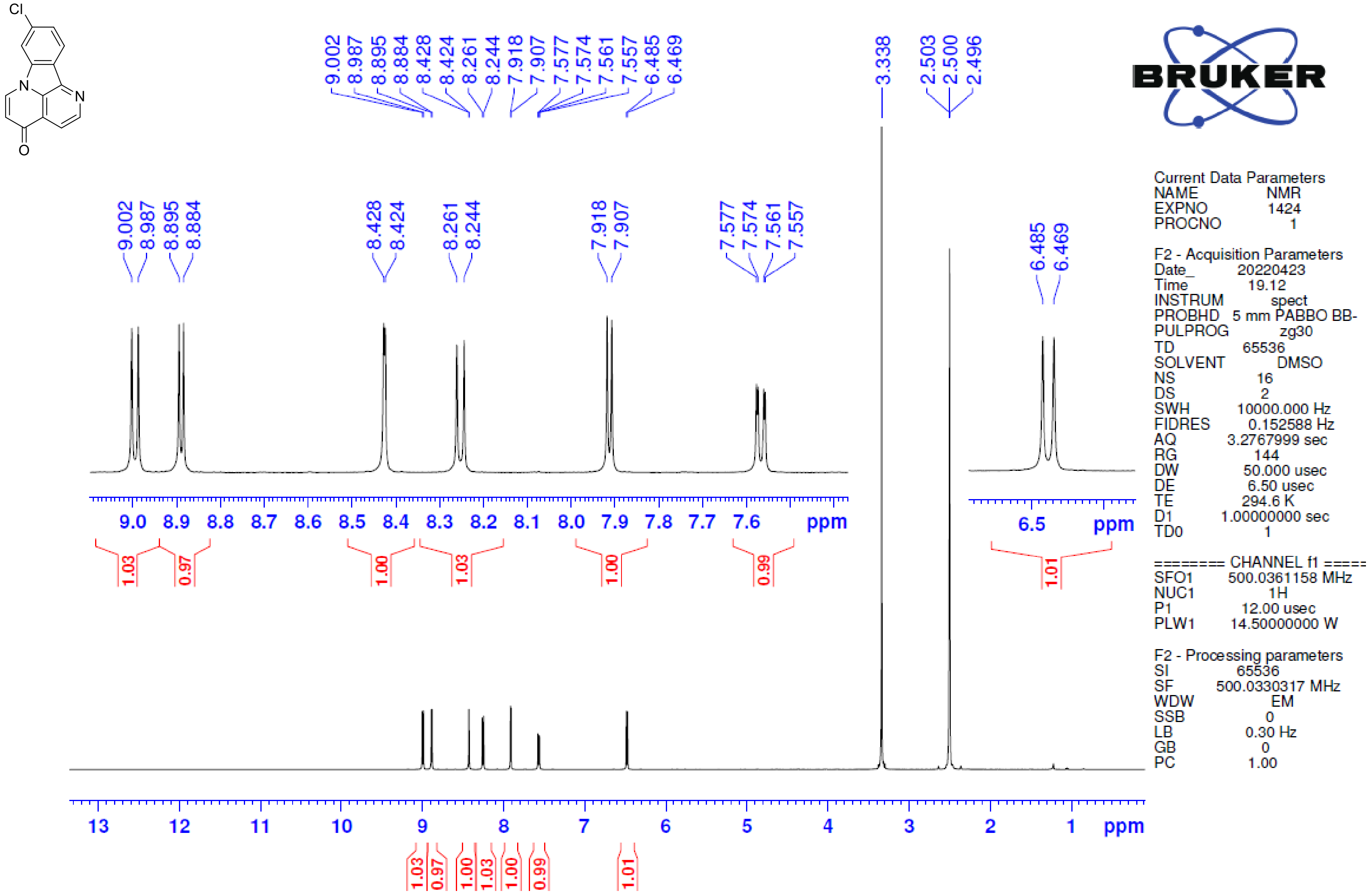

9-Chloro-4*H*-indolo[3,2,1-*ij*][1,7]naphthyridin-4-one (**10f**), ^{13}C NMR in DMSO-d_6, 125 MHz

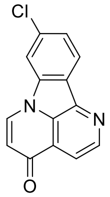

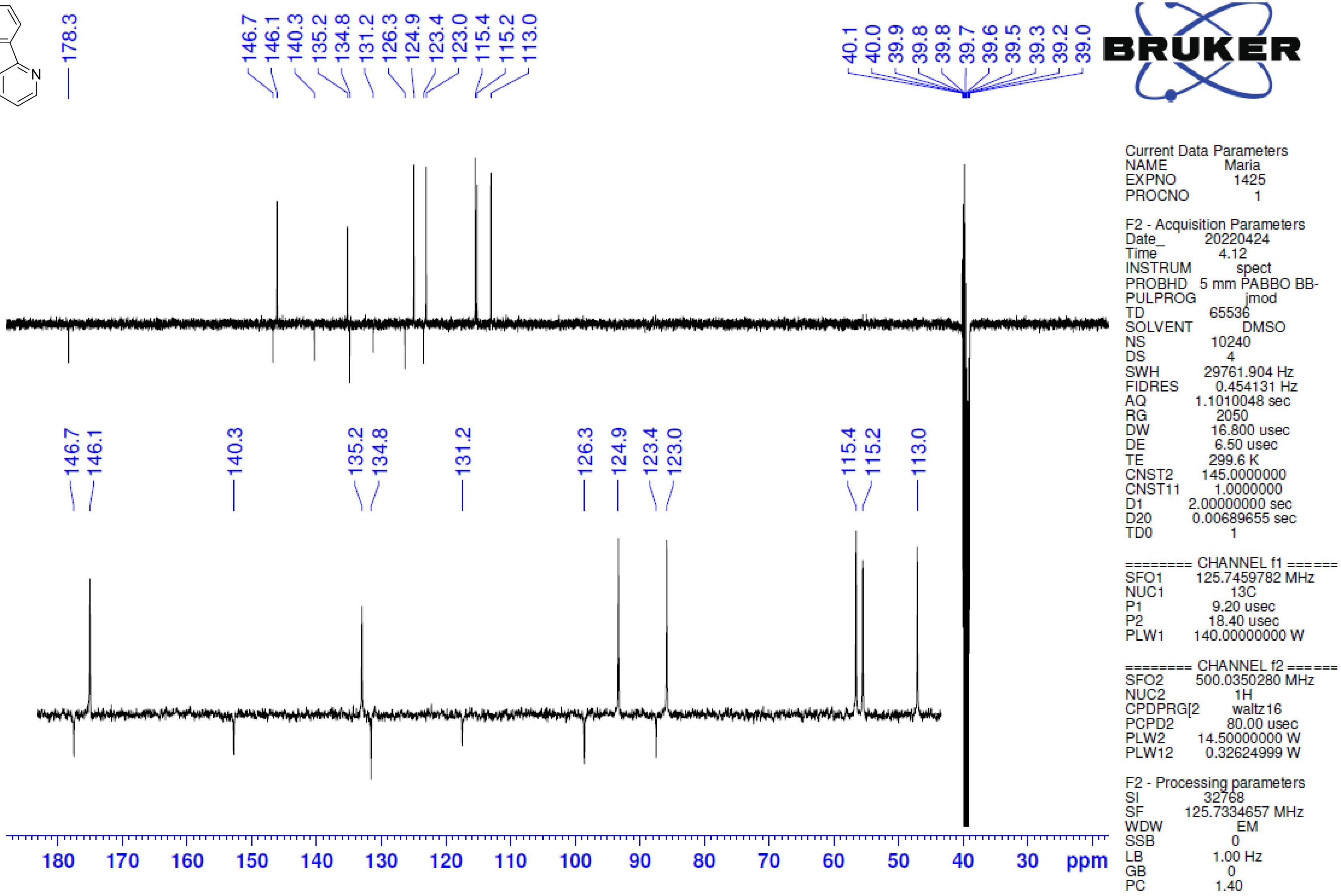

9-Fluoro-4*H*-indolo[3,2,1-*ij*][1,7]naphthyridin-4-one (**10g**), ^{1}H NMR in $CDCl_3$, 500 MHz

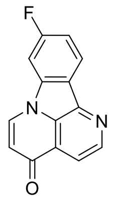

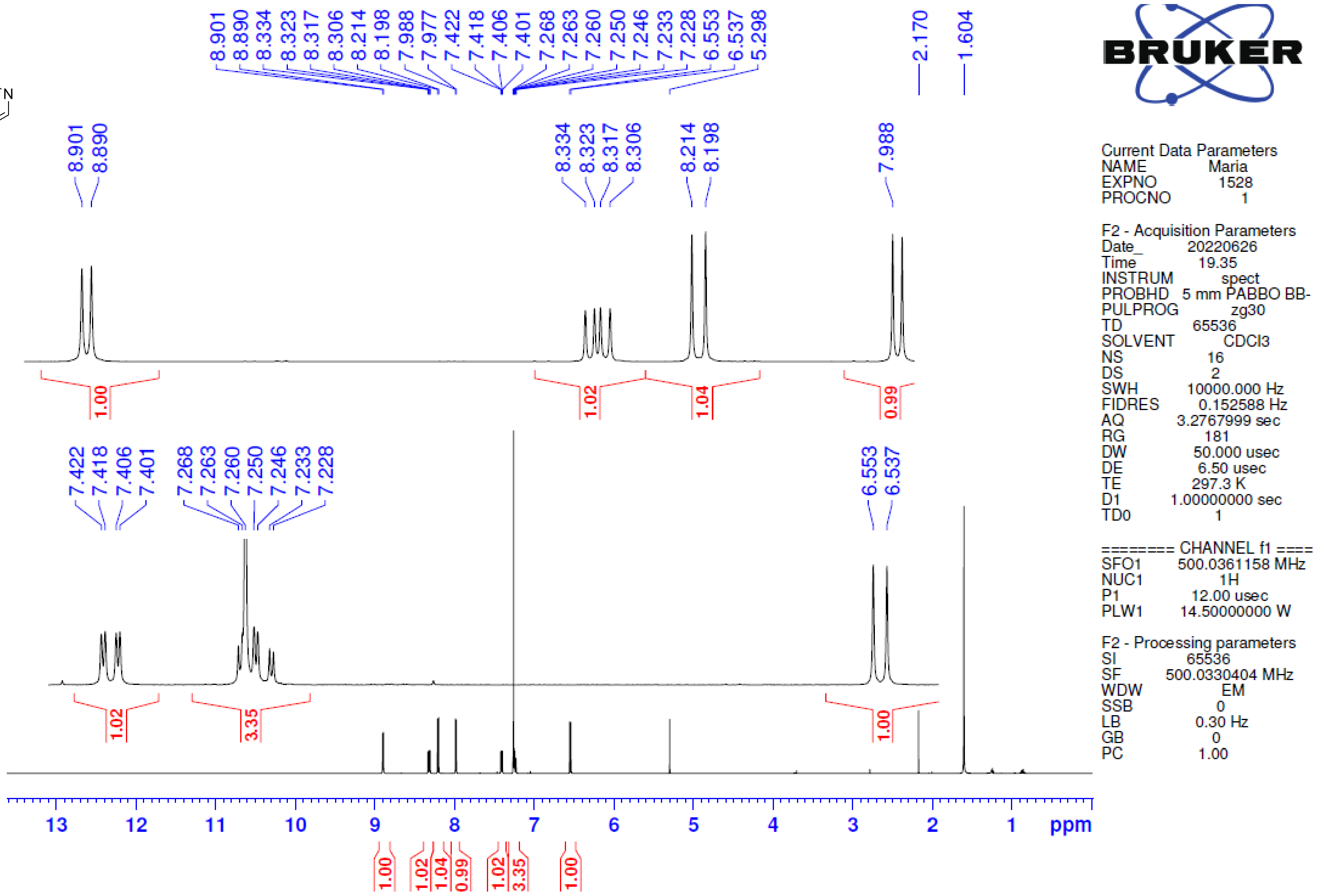

9-Fluoro-4*H*-indolo[3,2,1-*ij*][1,7]naphthyridin-4-one (**10g**), ^{13}C NMR in $CDCl_3$, 125 MHz

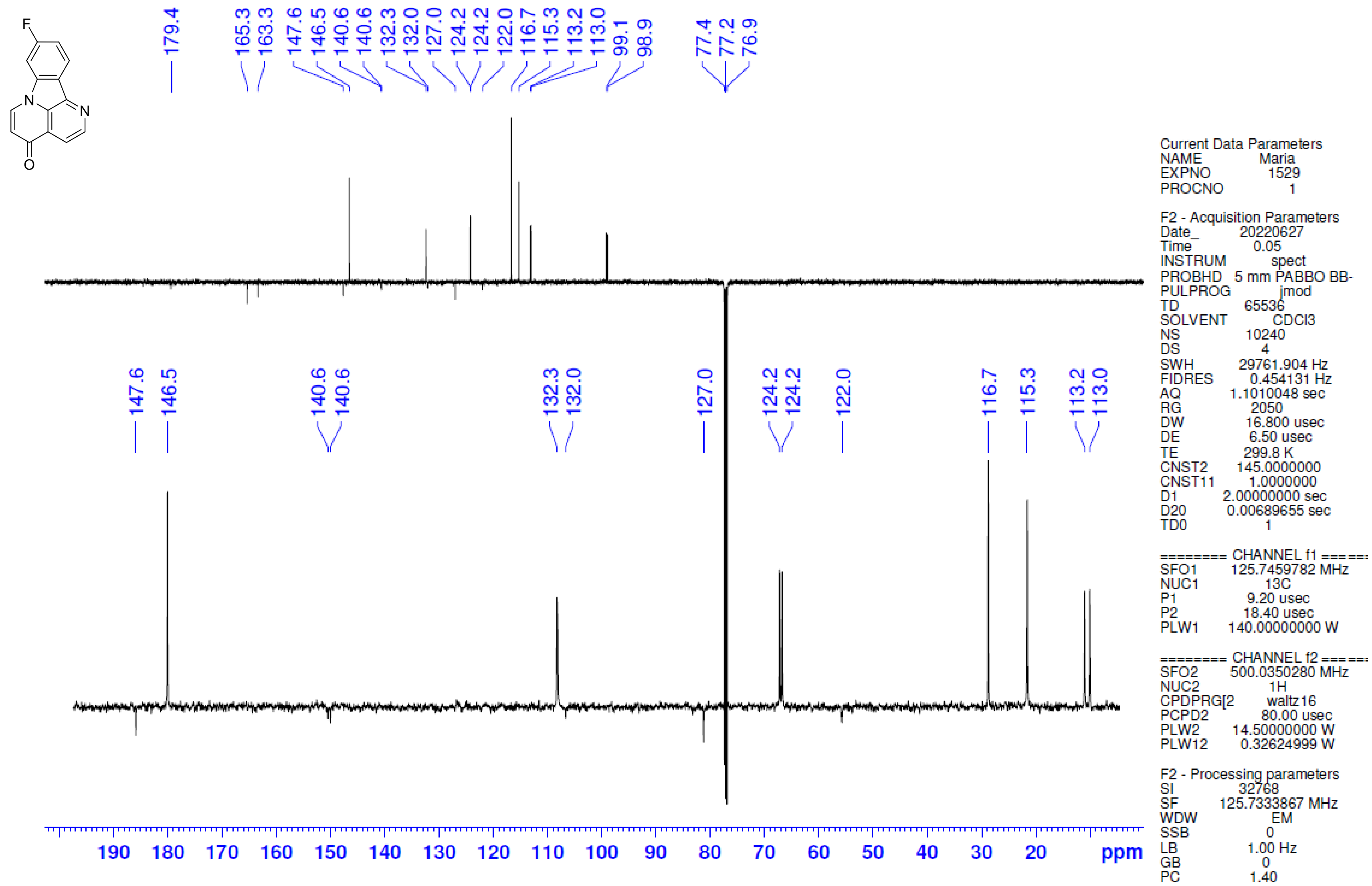

10-Methoxy-4*H*-indolo[3,2,1-*ij*][1,7]naphthyridin-4-one (**10h**), ^{1}H NMR in $CDCl_3$, 500 MHz

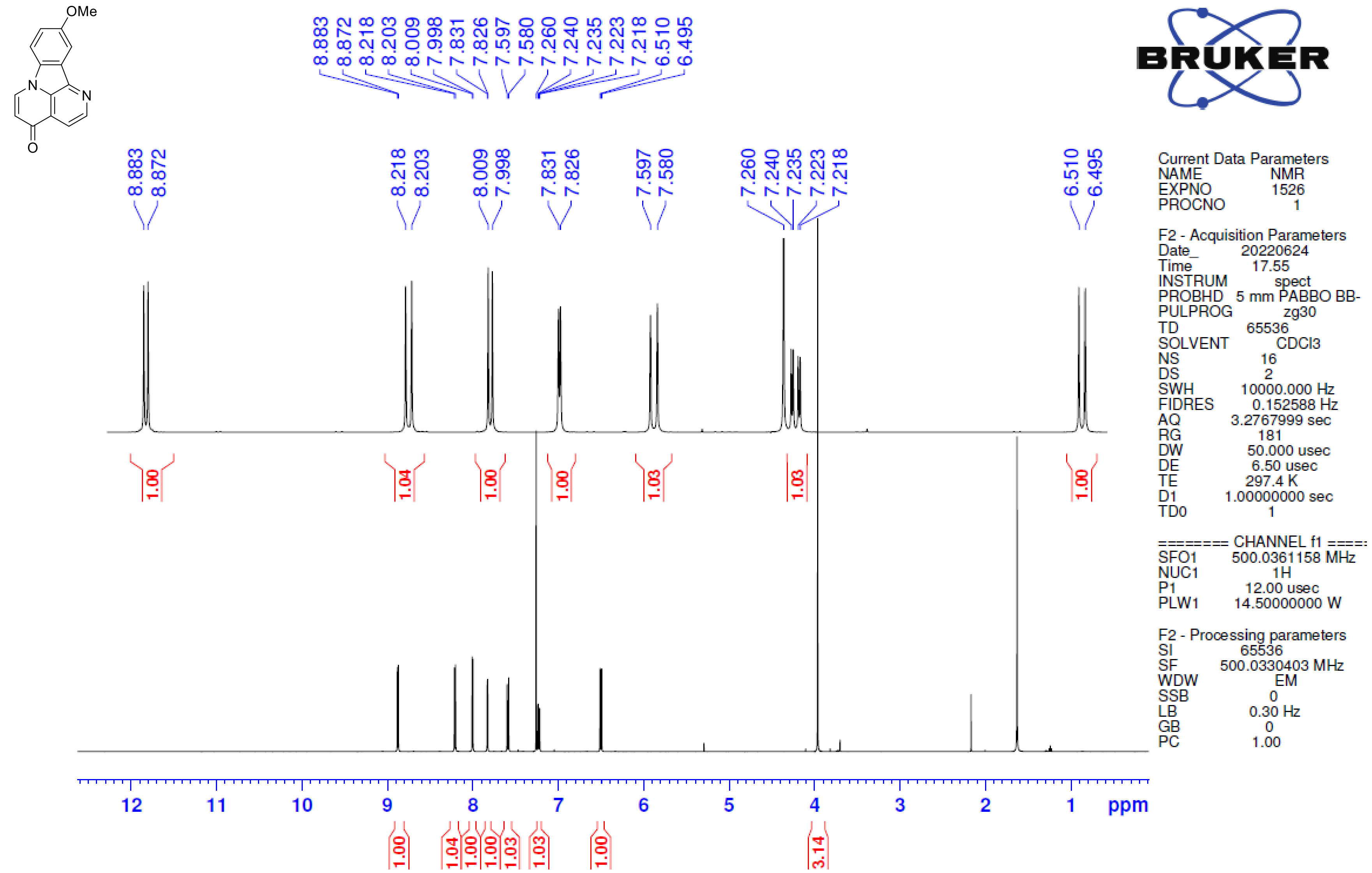

10-Methoxy-4*H*-indolo[3,2,1-*ij*][1,7]naphthyridin-4-one (**10h**), ^{13}C NMR in $CDCl_3$, 125 MHz

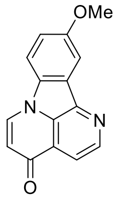

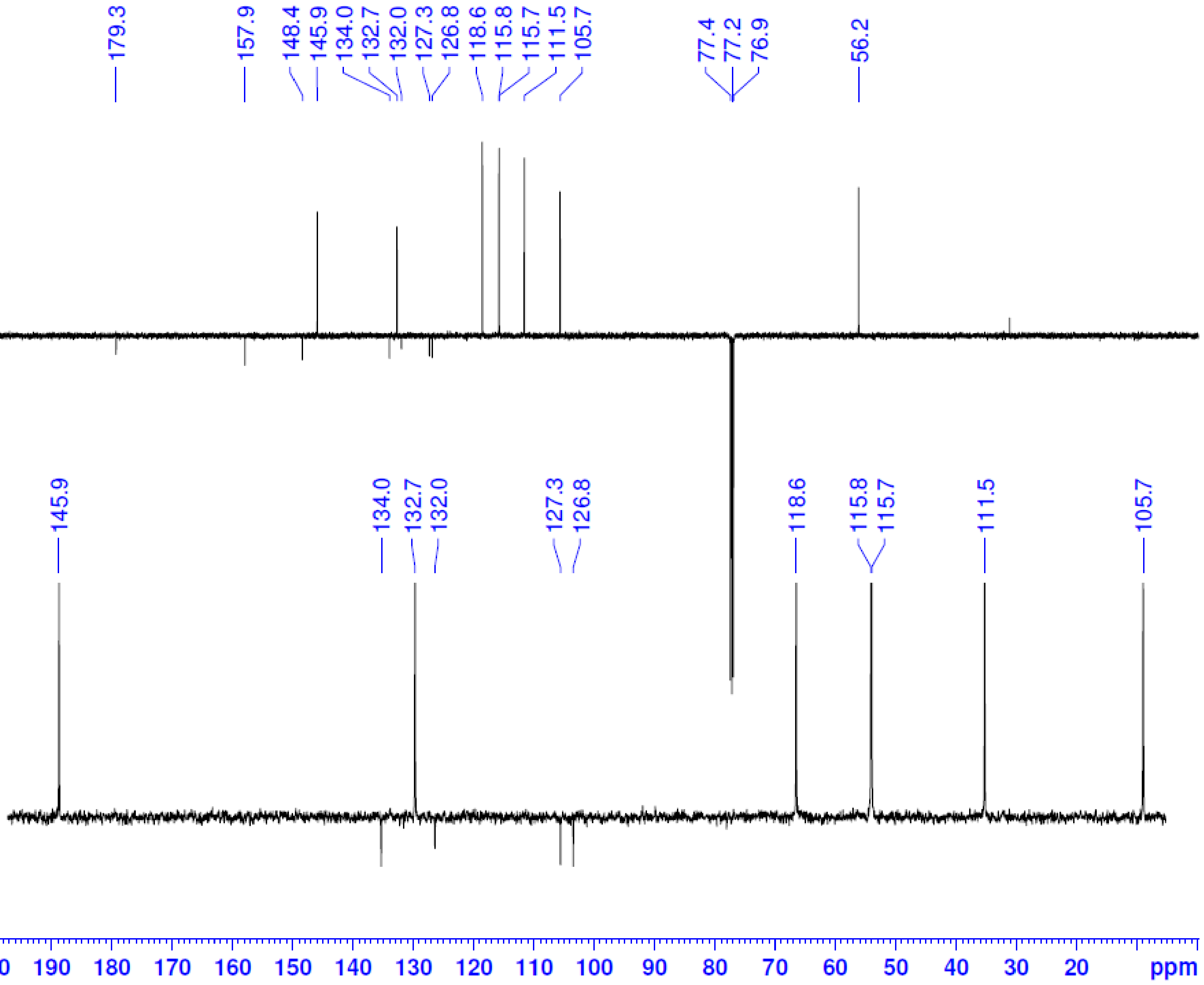

Current Data Parameters
NAME Maria
EXPNO 1527
PROCNO 1

F2 - Acquisition Parameters
Date_ 20220627
Time 10.08
INSTRUM spect
PROBHD 5 mm PABBO BB-
PULPROG jmod
TD 65536
SOLVENT CDCl3
NS 2118
DS 4
SWH 29761.904 Hz
FIDRES 0.454131 Hz
AQ 1.1010048 sec
RG 2050
DW 16.800 usec
DE 6.50 usec
TE 299.3 K
CNST2 145.0000000
CNST11 1.0000000
D1 2.00000000 sec
D20 0.00689655 sec
TD0 1

======== CHANNEL f1 ======
SFO1 125.7459782 MHz
NUC1 13C
P1 9.20 usec
P2 18.40 usec
PLW1 140.00000000 W

======== CHANNEL f2 ======
SFO2 500.0350280 MHz
NUC2 1H
CPDPRG[2 waltz16
PCPD2 80.00 usec
PLW2 14.50000000 W
PLW12 0.32624999 W

F2 - Processing parameters
SI 32768
SF 125.7333872 MHz
WDW EM
SSB 0
LB 1.00 Hz
GB 0
PC 1.40

10-(Trifluoromethoxy)-4*H*-indolo[3,2,1-*ij*][1,7]naphthyridin-4-one (**10i**), ^{1}H NMR in DMSO-d_6, 500 MHz

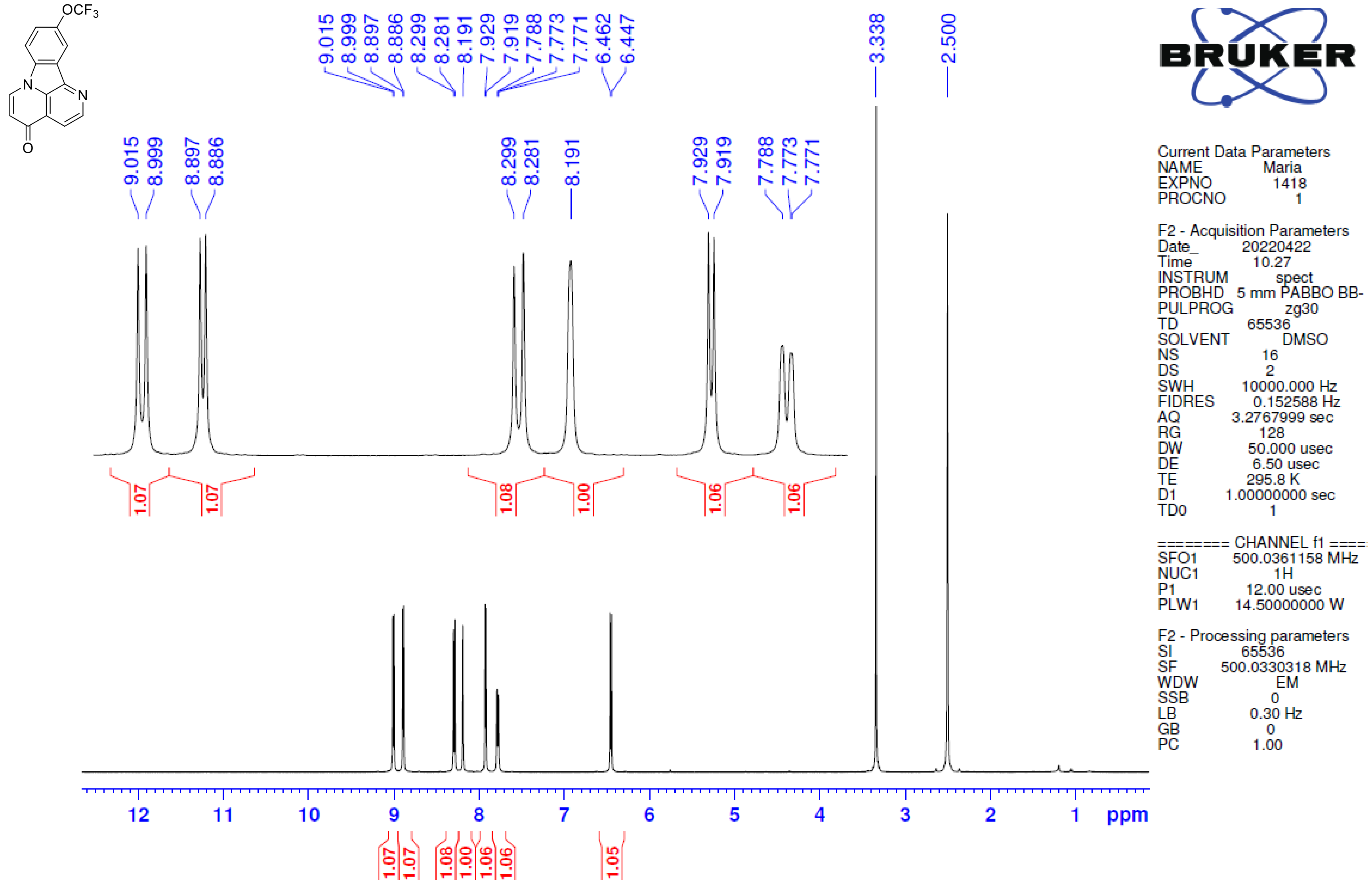

10-(Trifluoromethoxy)-4*H*-indolo[3,2,1-*ij*][1,7]naphthyridin-4-one (**10i**), ^{13}C NMR in DMSO-d_6, 125 MHz

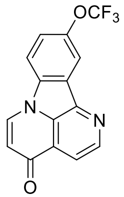

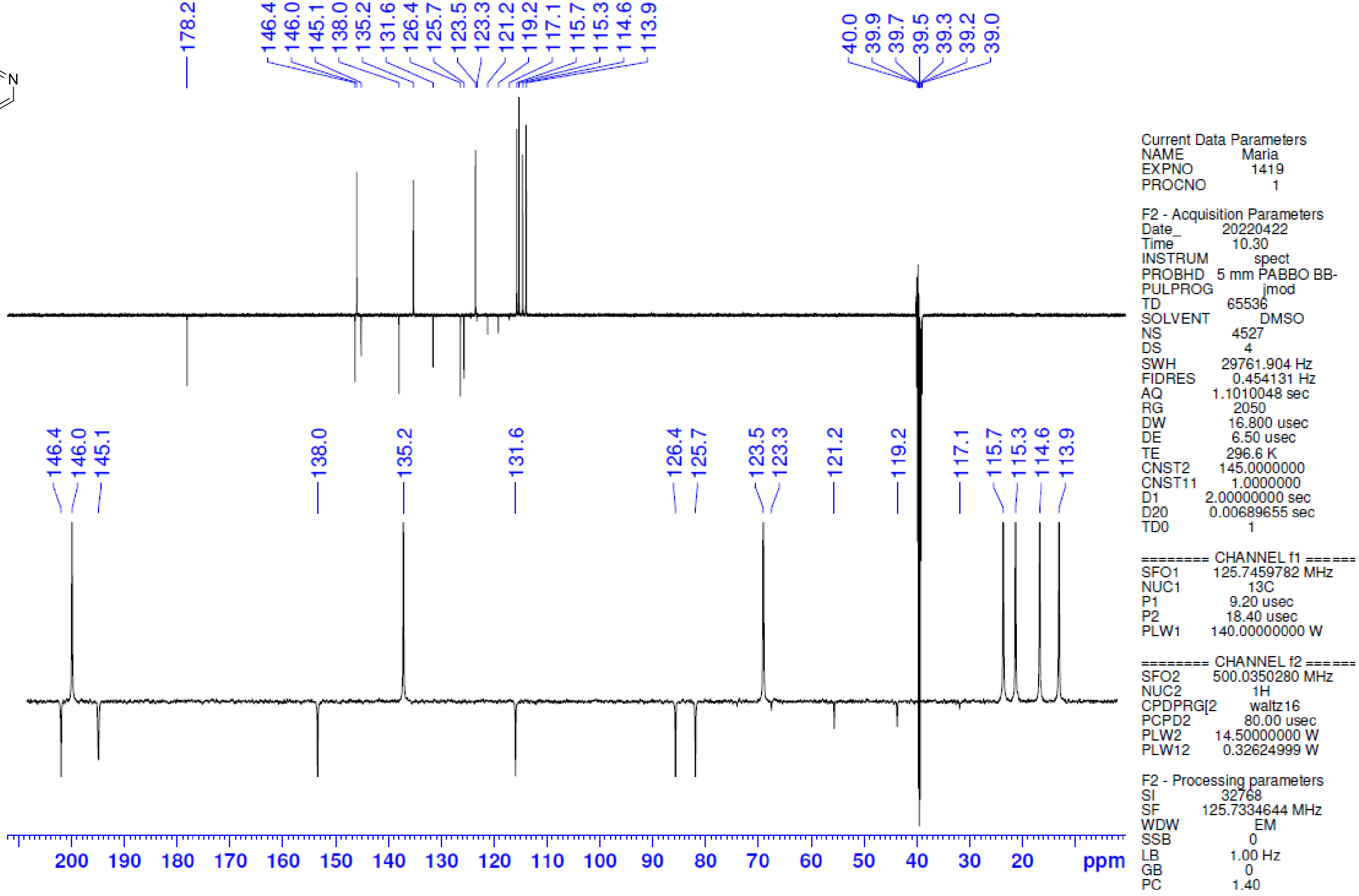

10-Chloro-4*H*-indolo[3,2,1-*ij*][1,7]naphthyridin-4-one (**10j**), ^{1}H NMR in DMSO-d_6, 500 MHz

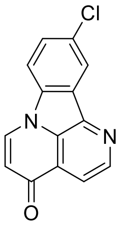

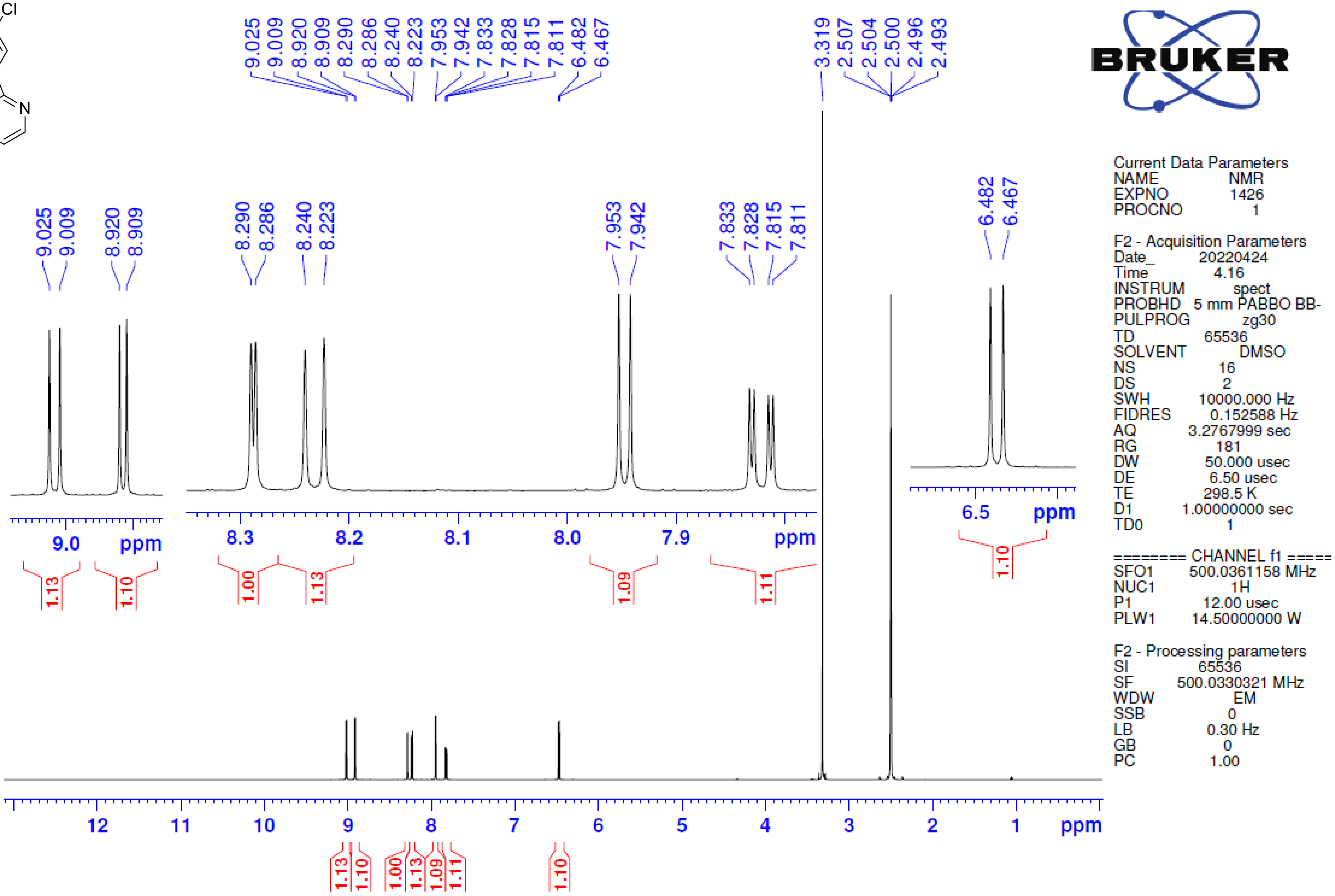

10-Chloro-4*H*-indolo[3,2,1-*ij*][1,7]naphthyridin-4-one (**10j**), ^{13}C NMR in DMSO-d_6, 125 MHz

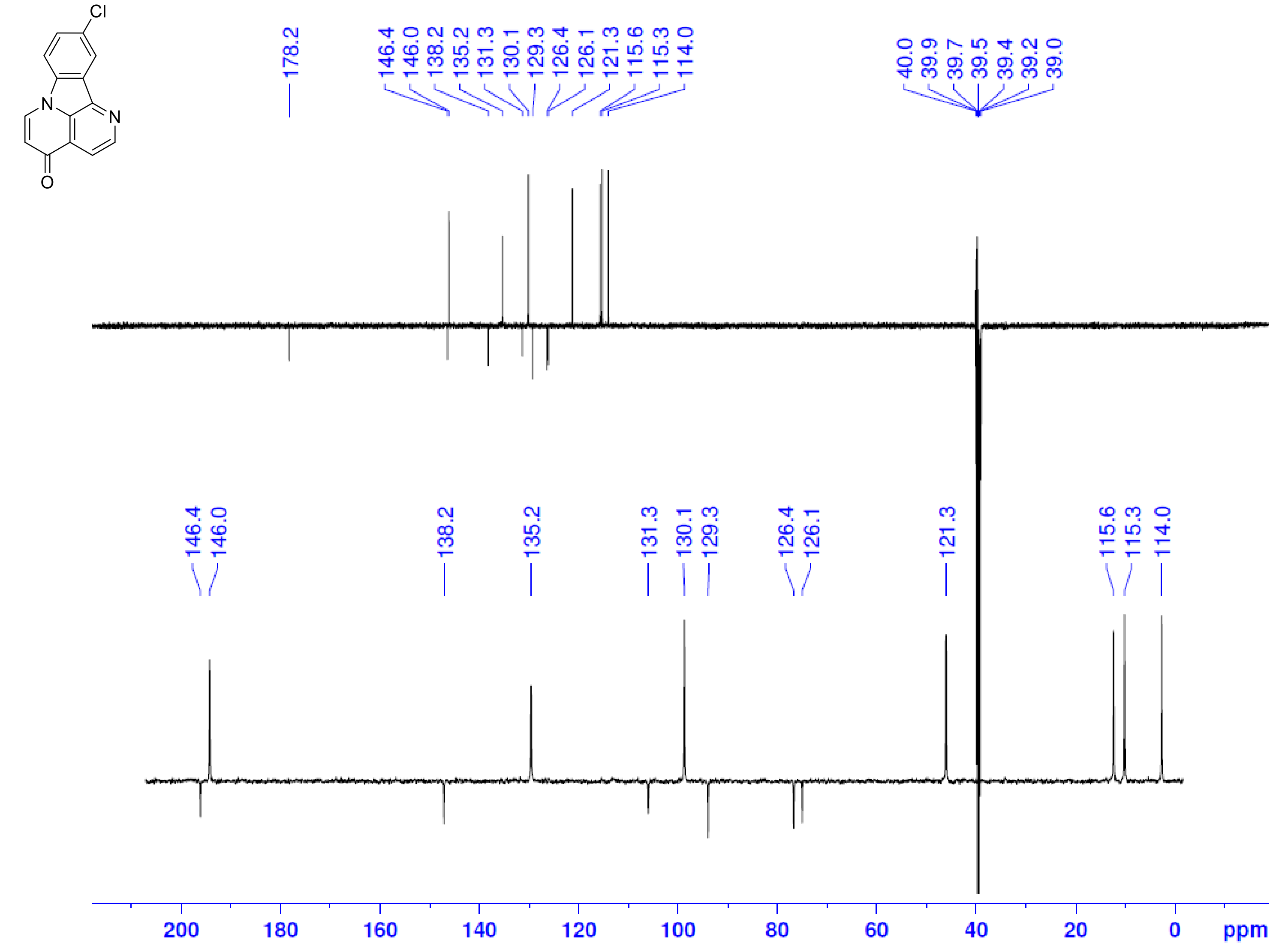

Current Data Parameters
NAME Maria
EXPNO 1427
PROCNO 1

F2 - Acquisition Parameters
Date_ 20220424
Time 11.42
INSTRUM spect
PROBHD 5 mm PABBO BB-
PULPROG jmod
TD 65536
SOLVENT DMSO
NS 8451
DS 4
SWH 29761.904 Hz
FIDRES 0.454131 Hz
AQ 1.1010048 sec
RG 2050
DW 16.800 usec
DE 6.50 usec
TE 297.3 K
CNST2 145.0000000
CNST11 1.0000000
D1 2.00000000 sec
D20 0.00689655 sec
TD0 1

======== CHANNEL f1 ======
SFO1 125.7459782 MHz
NUC1 13C
P1 9.20 usec
P2 18.40 usec
PLW1 140.00000000 W

======== CHANNEL f2 ======
SFO2 500.0350280 MHz
NUC2 1H
CPDPRG[2 waltz16
PCPD2 80.00 usec
PLW2 14.50000000 W
PLW12 0.32624999 W

F2 - Processing parameters
SI 32768
SF 125.7334659 MHz
WDW EM
SSB 0
LB 1.00 Hz
GB 0
PC 1.40

10-Fluoro-4*H*-indolo[3,2,1-*ij*][1,7]naphthyridin-4-one (**10k**), ^{1}H NMR in DMSO-d_6, 500 MHz

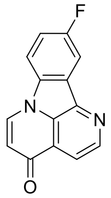

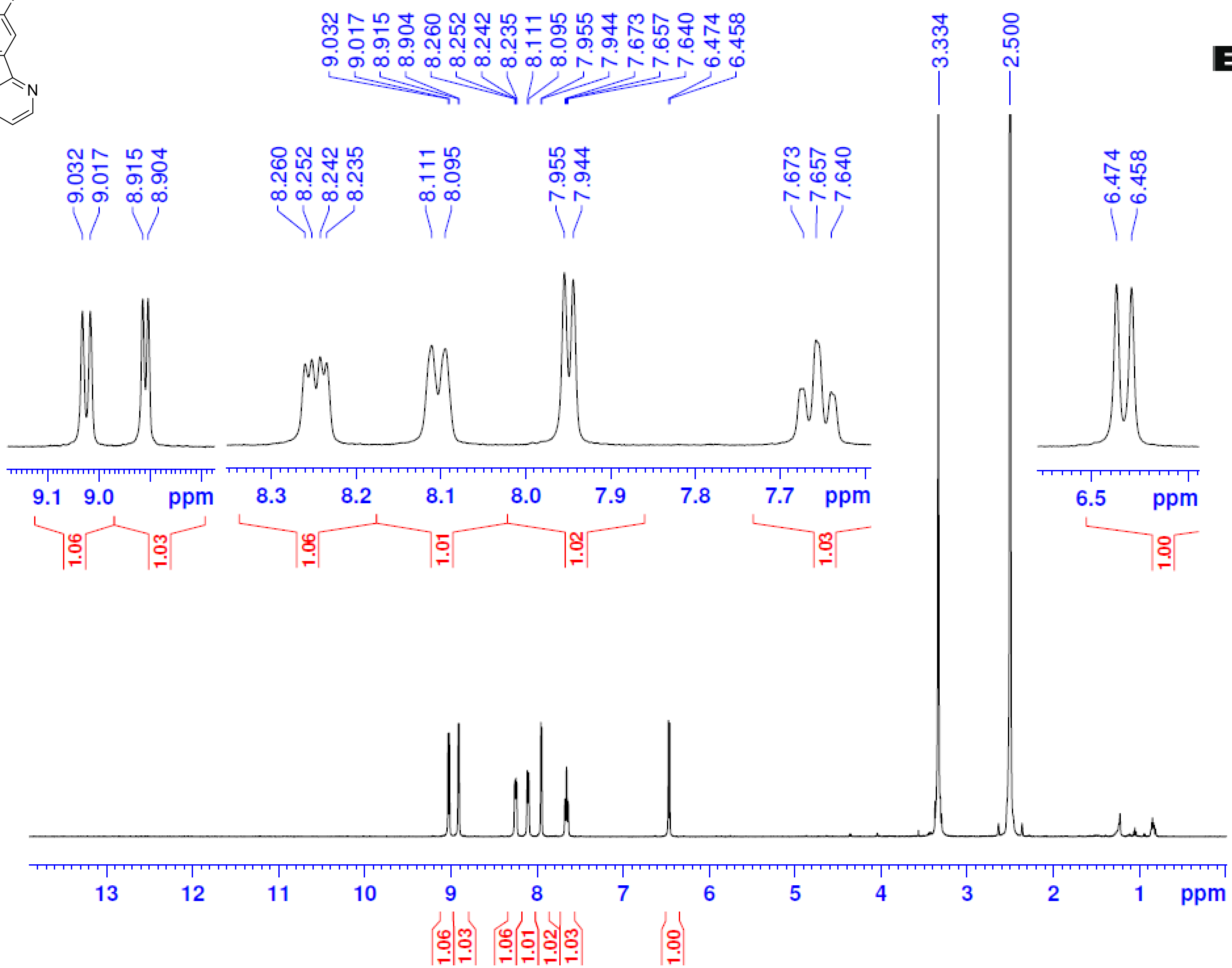

Current Data Parameters
NAME NMR
EXPNO 1416
PROCNO 1

F2 - Acquisition Parameters
Date_ 20220421
Time 20.59
INSTRUM spect
PROBHD 5 mm PABBO BB-
PULPROG zg30
TD 65536
SOLVENT DMSO
NS 16
DS 2
SWH 10000.000 Hz
FIDRES 0.152588 Hz
AQ 3.2767999 sec
RG 144
DW 50.000 usec
DE 6.50 usec
TE 295.5 K
D1 1.00000000 sec
TD0 1

======== CHANNEL f1 ========
SFO1 500.0361158 MHz
NUC1 1H
P1 12.00 usec
PLW1 14.50000000 W

F2 - Processing parameters
SI 65536
SF 500.0330317 MHz
WDW EM
SSB 0
LB 0.30 Hz
GB 0
PC 1.00

10-Fluoro-4*H*-indolo[3,2,1-*ij*][1,7]naphthyridin-4-one (**10k**), ^{13}C NMR in DMSO-d_6, 125 MHz

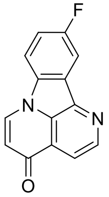

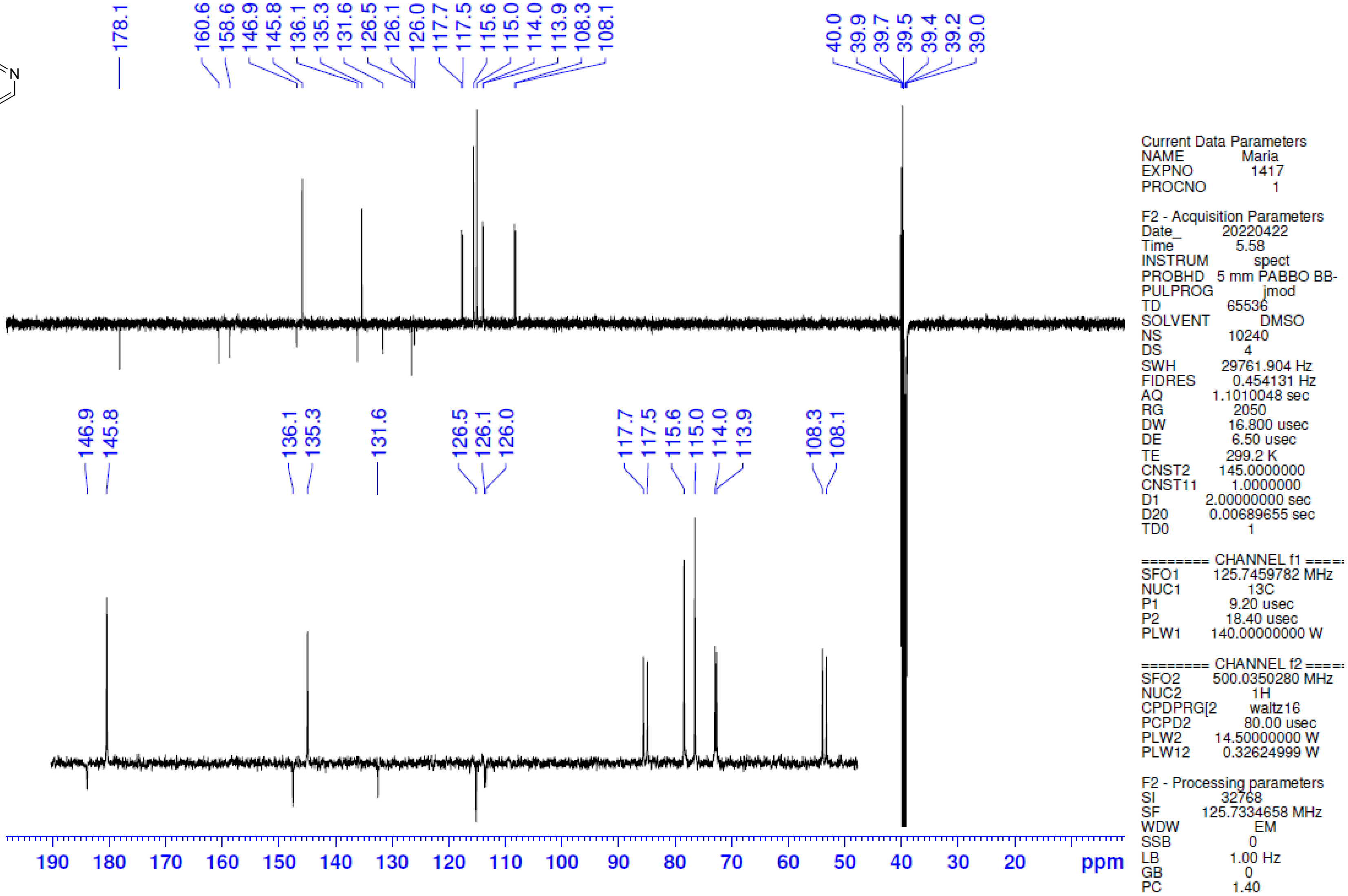

8-Bromo-1,6-naphthyridin-4(1*H*)-one (**11**), ^{1}H NMR in DMSO-d_6, 500 MHz

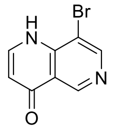

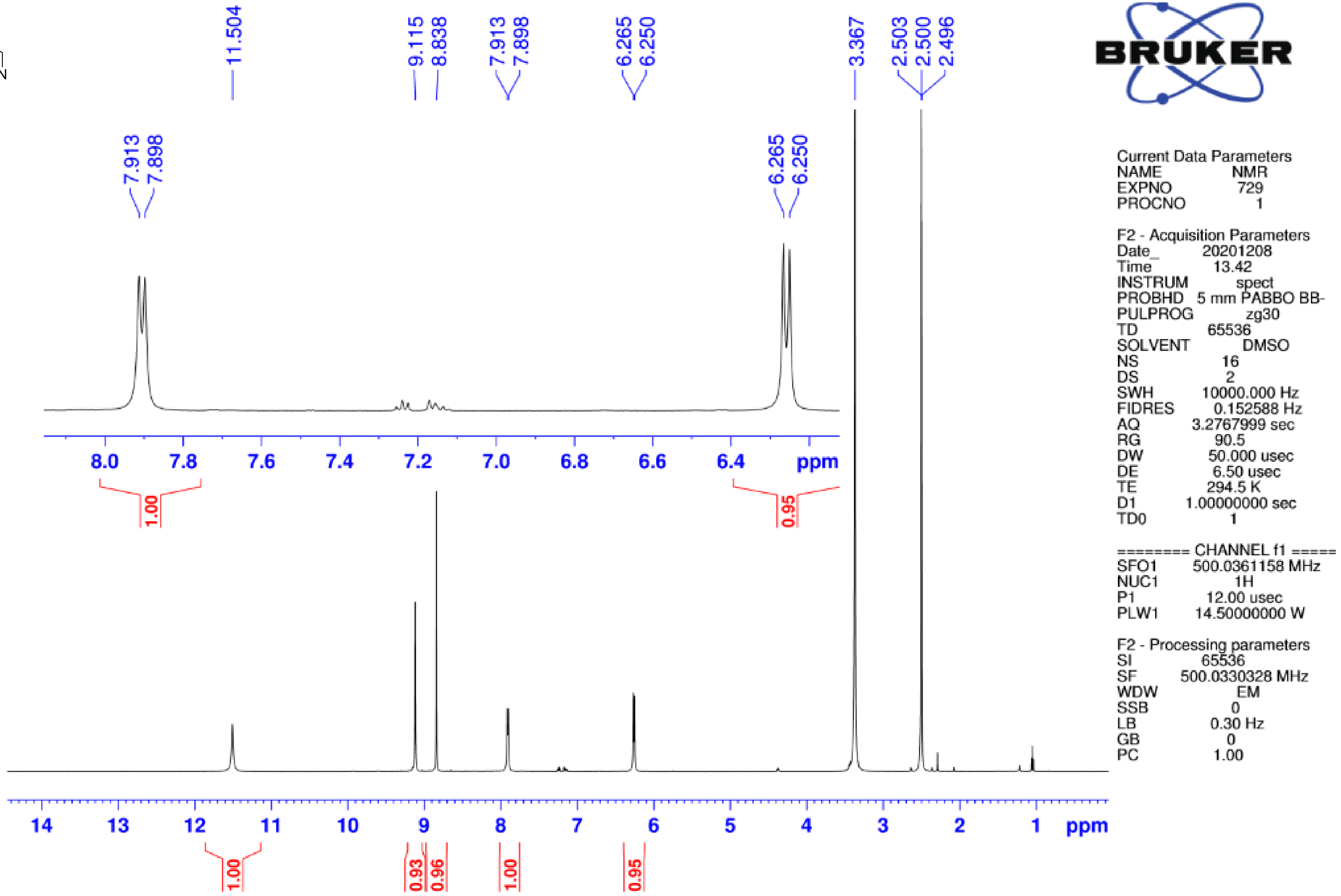

8-Bromo-1,6-naphthyridin-4(1*H*)-one (**11**), ^{13}C NMR in DMSO-d_6, 125 MHz

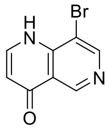

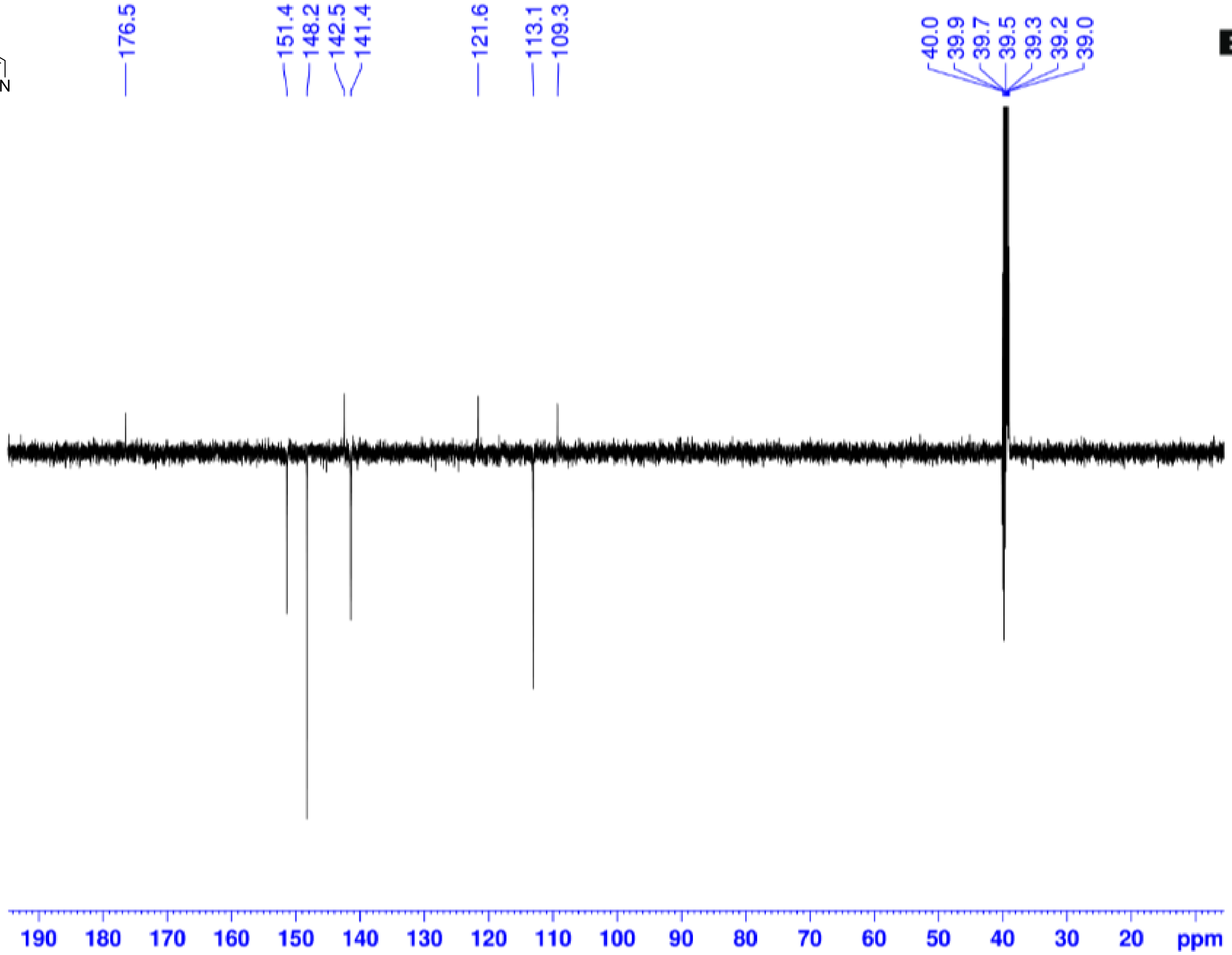

BRUKER

Current Data Parameters
NAME NMR
EXPNO 730
PROCNO 1

F2 - Acquisition Parameters
Date_ 20201208
Time 13.46
INSTRUM spect
PROBHD 5 mm PABBO BB-
PULPROG jmod
TD 65536
SOLVENT DMSO
NS 512
DS 4
SWH 29761.904 Hz
FIDRES 0.454131 Hz
AQ 1.1010048 sec
RG 2050
DW 16.800 usec
DE 6.50 usec
TE 295.3 K
CNST2 145.0000000
CNST11 1.0000000
D1 2.00000000 sec
D20 0.00689655 sec
TD0 1

======== CHANNEL f1 ======
SFO1 125.7459782 MHz
NUC1 13C
P1 9.20 usec
P2 18.40 usec
PLW1 140.00000000 W

======== CHANNEL f2 ======
SFO2 500.0350280 MHz
NUC2 1H
CPDPRG[2 waltz16
PCPD2 80.00 usec
PLW2 14.50000000 W
PLW12 0.32624999 W

F2 - Processing parameters
SI 32768
SF 125.7334610 MHz
WDW EM
SSB 0
LB 1.00 Hz
GB 0
PC 1.40

8-Phenyl-1,6-naphthyridin-4(1*H*)-one (**12**), ^{1}H NMR in DMSO-d_6, 500 MHz

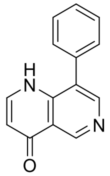

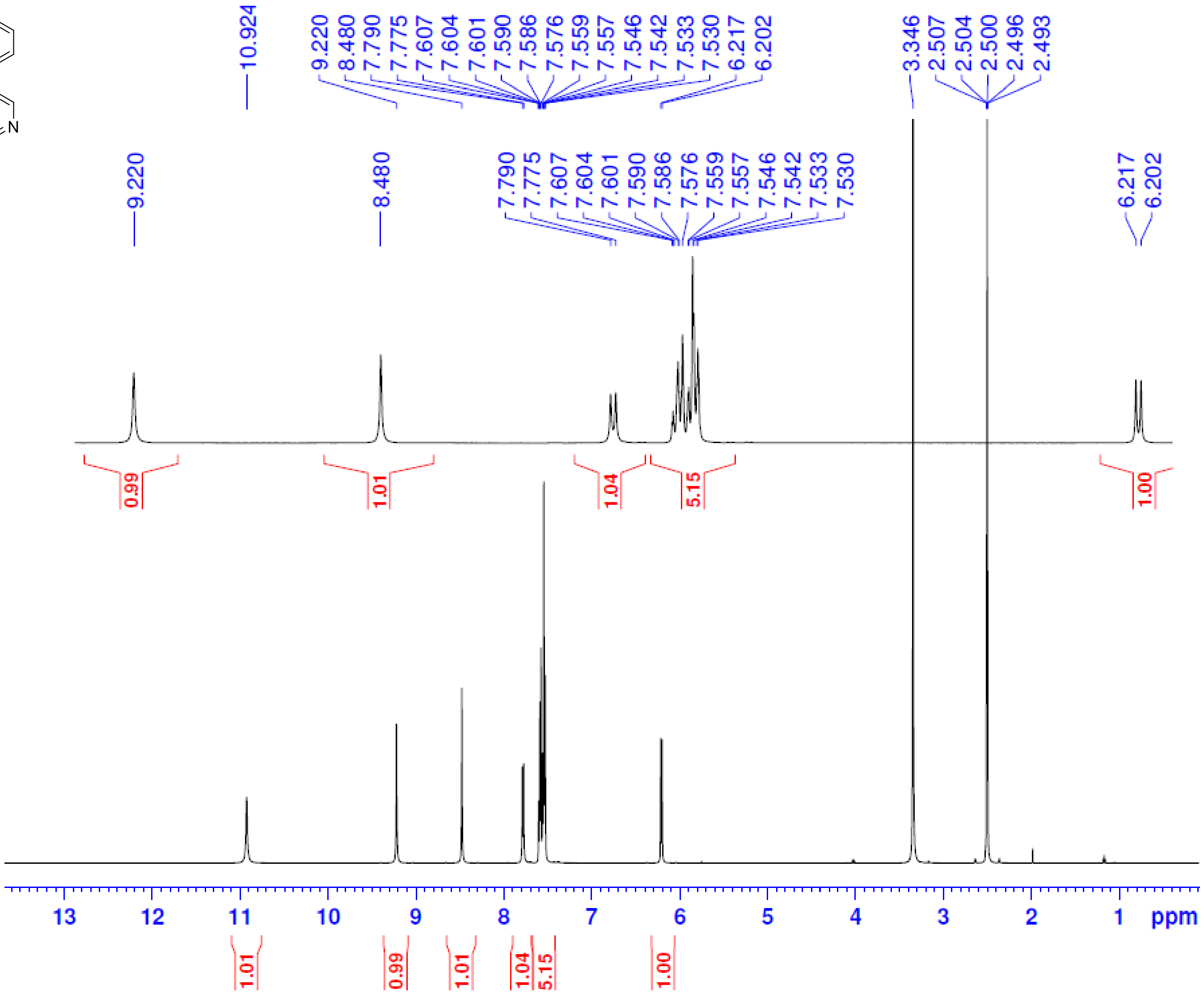

Current Data Parameters
NAME Maria
EXPNO 1137
PROCNO 1

F2 - Acquisition Parameters
Date_ 20210713
Time 20.11
INSTRUM spect
PROBHD 5 mm PABBO BB-
PULPROG zg30
TD 65536
SOLVENT DMSO
NS 16
DS 2
SWH 10000.000 Hz
FIDRES 0.152588 Hz
AQ 3.2767999 sec
RG 101
DW 50.000 usec
DE 6.50 usec
TE 298.1 K
D1 1.00000000 sec
TD0 1

======== CHANNEL f1 =====
SFO1 500.0361158 MHz
NUC1 1H
P1 12.00 usec
PLW1 14.50000000 W

F2 - Processing parameters
SI 65536
SF 500.0330321 MHz
WDW EM
SSB 0
LB 0.30 Hz
GB 0
PC 1.00

8-Phenyl-1,6-naphthyridin-4(1*H*)-one (**12**), ^{13}C NMR in DMSO-d_6, 125 MHz

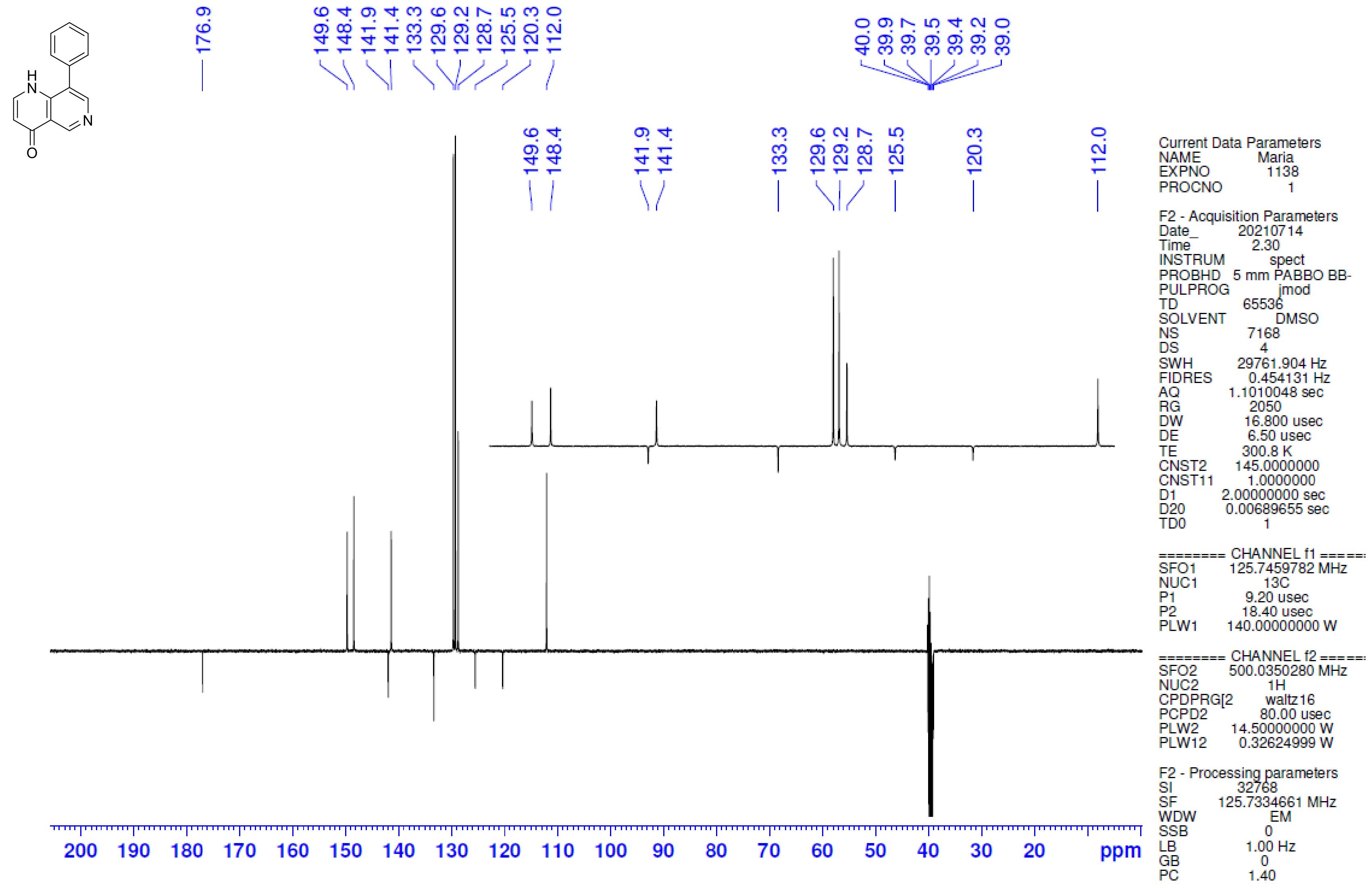

5-{[(2-Bromopyrid-3-yl)amino]methylene}-2,2-dimethyl-1,3-dioxane-4,6-dione (**14**), ^{1}H NMR in $CDCl_3$, 500 MHz

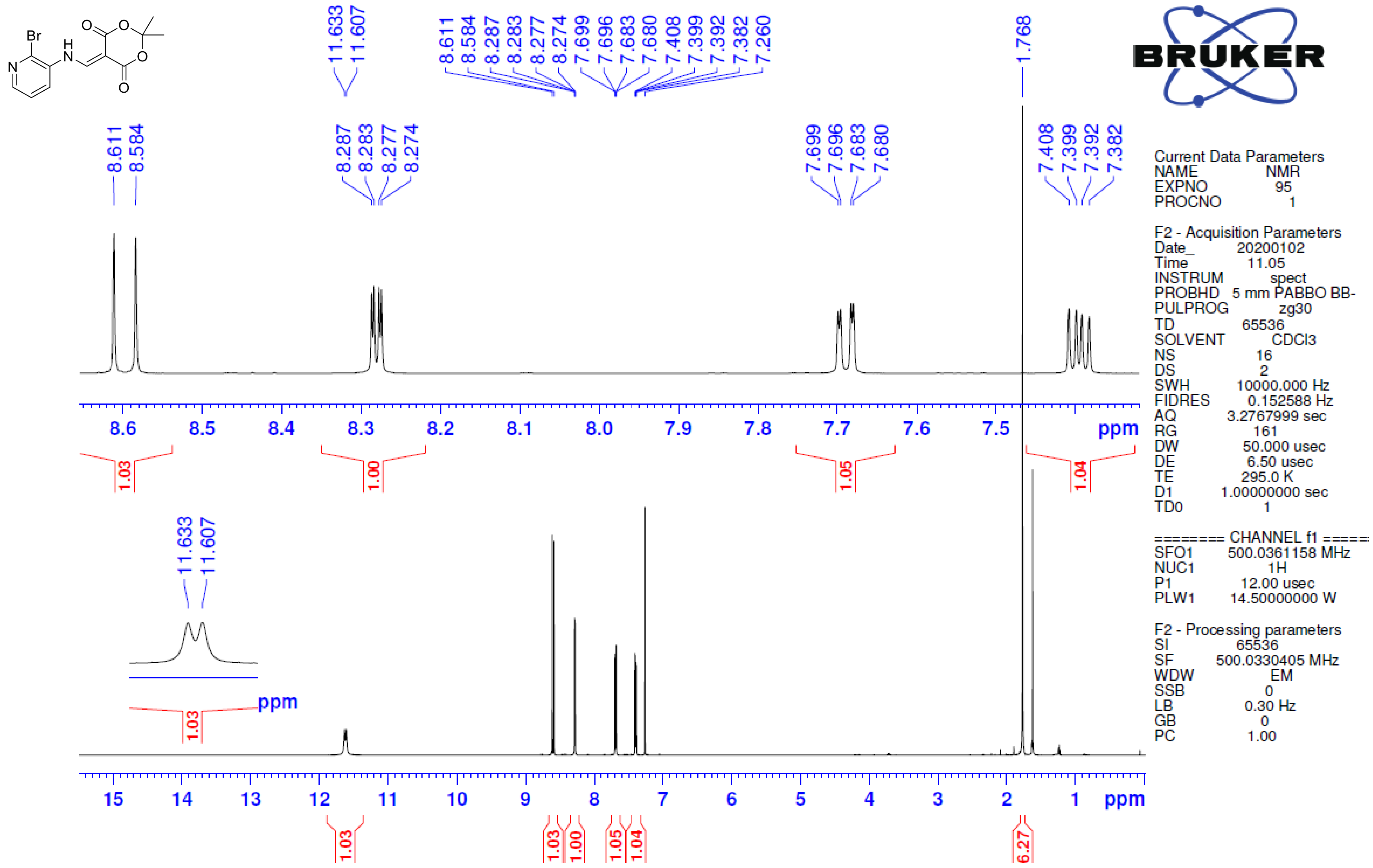

Current Data Parameters
NAME NMR
EXPNO 95
PROCNO 1

F2 - Acquisition Parameters
Date_ 20200102
Time 11.05
INSTRUM spect
PROBHD 5 mm PABBO BB-
PULPROG zg30
TD 65536
SOLVENT CDCl3
NS 16
DS 2
SWH 10000.000 Hz
FIDRES 0.152588 Hz
AQ 3.2767999 sec
RG 161
DW 50.000 usec
DE 6.50 usec
TE 295.0 K
D1 1.00000000 sec
TD0 1

======== CHANNEL f1 ======
SFO1 500.0361158 MHz
NUC1 1H
P1 12.00 usec
PLW1 14.50000000 W

F2 - Processing parameters
SI 65536
SF 500.0330405 MHz
WDW EM
SSB 0
LB 0.30 Hz
GB 0
PC 1.00

5-{[(2-Bromopyrid-3-yl)amino]methylene}-2,2-dimethyl-1,3-dioxane-4,6-dione (**14**), ^{13}C NMR in $CDCl_3$, 125 MHz

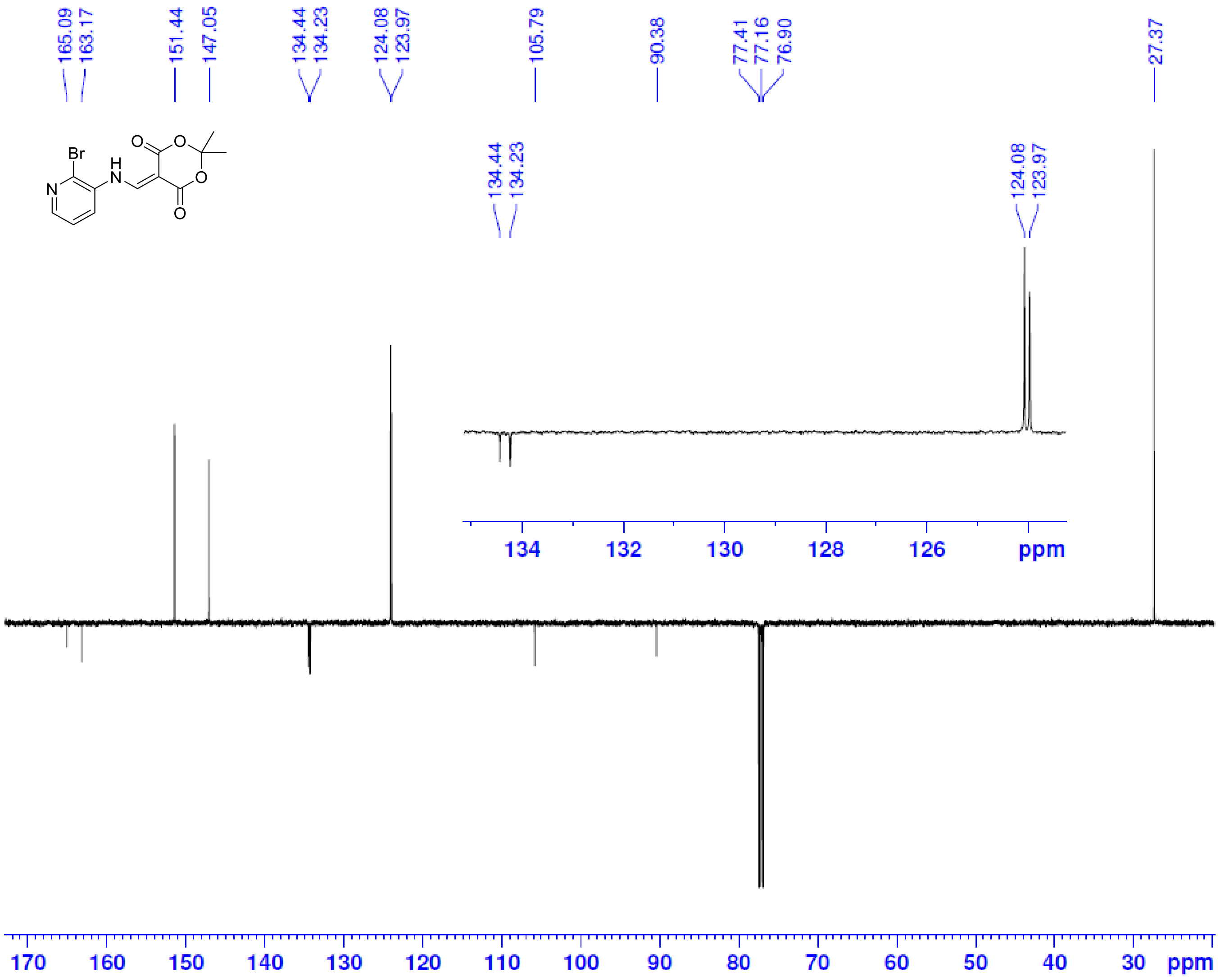

Current Data Parameters
NAME CANTHIN4ONE
EXPNO 96
PROCNO 1

F2 - Acquisition Parameters
Date_ 20200102
Time 12.12
INSTRUM spect
PROBHD 5 mm PABBO BB-
PULPROG jmod
TD 65536
SOLVENT CDCl3
NS 1382
DS 4
SWH 29761.904 Hz
FIDRES 0.454131 Hz
AQ 1.1010048 sec
RG 2050
DW 16.800 usec
DE 6.50 usec
TE 296.3 K
CNST2 145.0000000
CNST11 1.0000000
D1 2.00000000 sec
D20 0.00689655 sec
TD0 1

======== CHANNEL f1 =====
SFO1 125.7459782 MHz
NUC1 13C
P1 9.20 usec
P2 18.40 usec
PLW1 140.00000000 W

======== CHANNEL f2 =====
SFO2 500.0350280 MHz
NUC2 1H
CPDPRG[2 waltz16
PCPD2 80.00 usec
PLW2 14.50000000 W
PLW12 0.32624999 W

F2 - Processing parameters
SI 32768
SF 125.7333898 MHz
WDW EM
SSB 0
LB 1.00 Hz
GB 0
PC 1.40

8-Bromo-1,7-naphthyridin-4(1*H*)-one (**15**), ^{1}H NMR in DMSO-d_6, 300 MHz

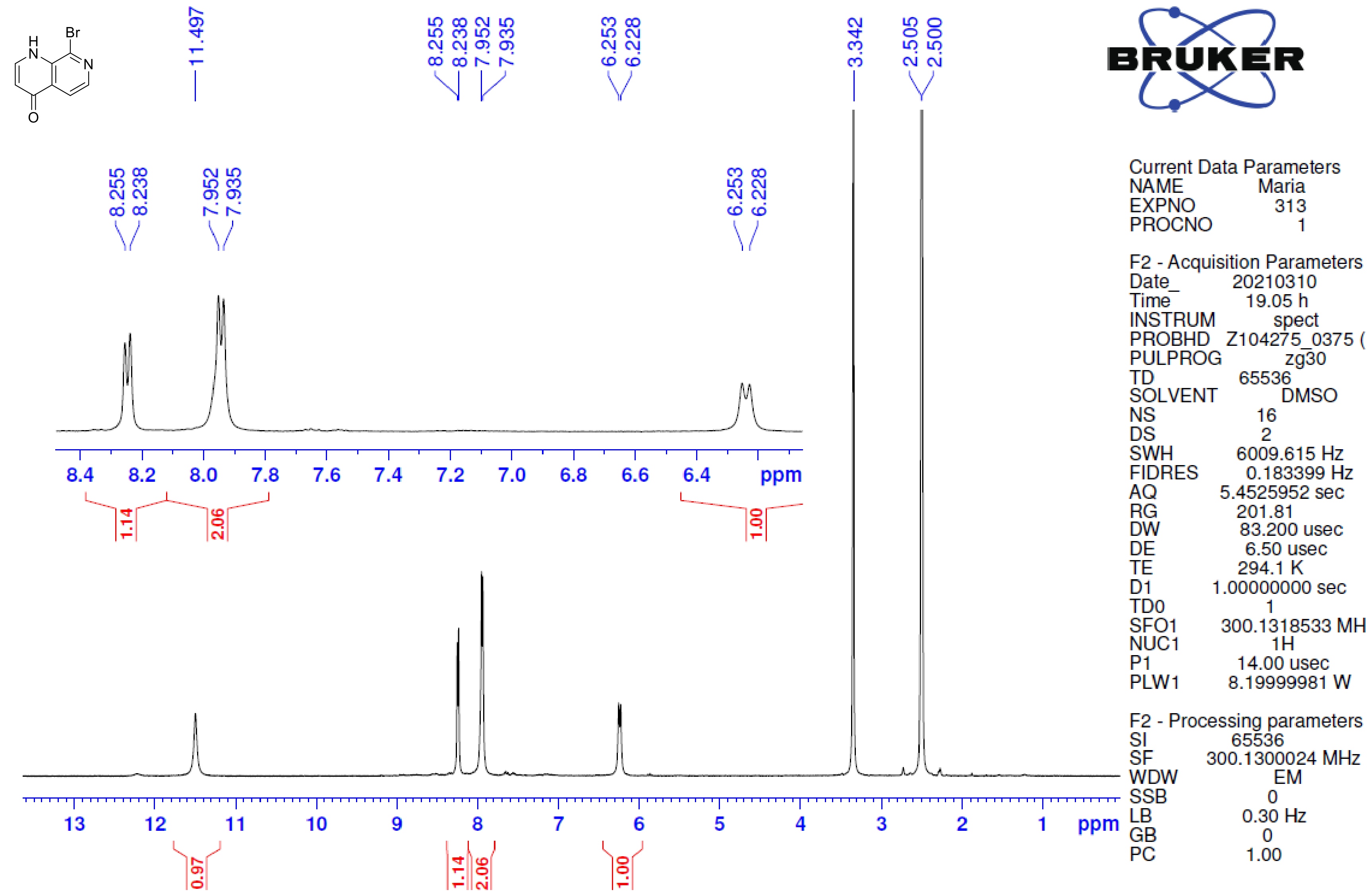

8-Bromo-1,7-naphthyridin-4(1*H*)-one (**15**), ^{13}C NMR in DMSO-d_6, 75 MHz

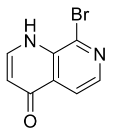

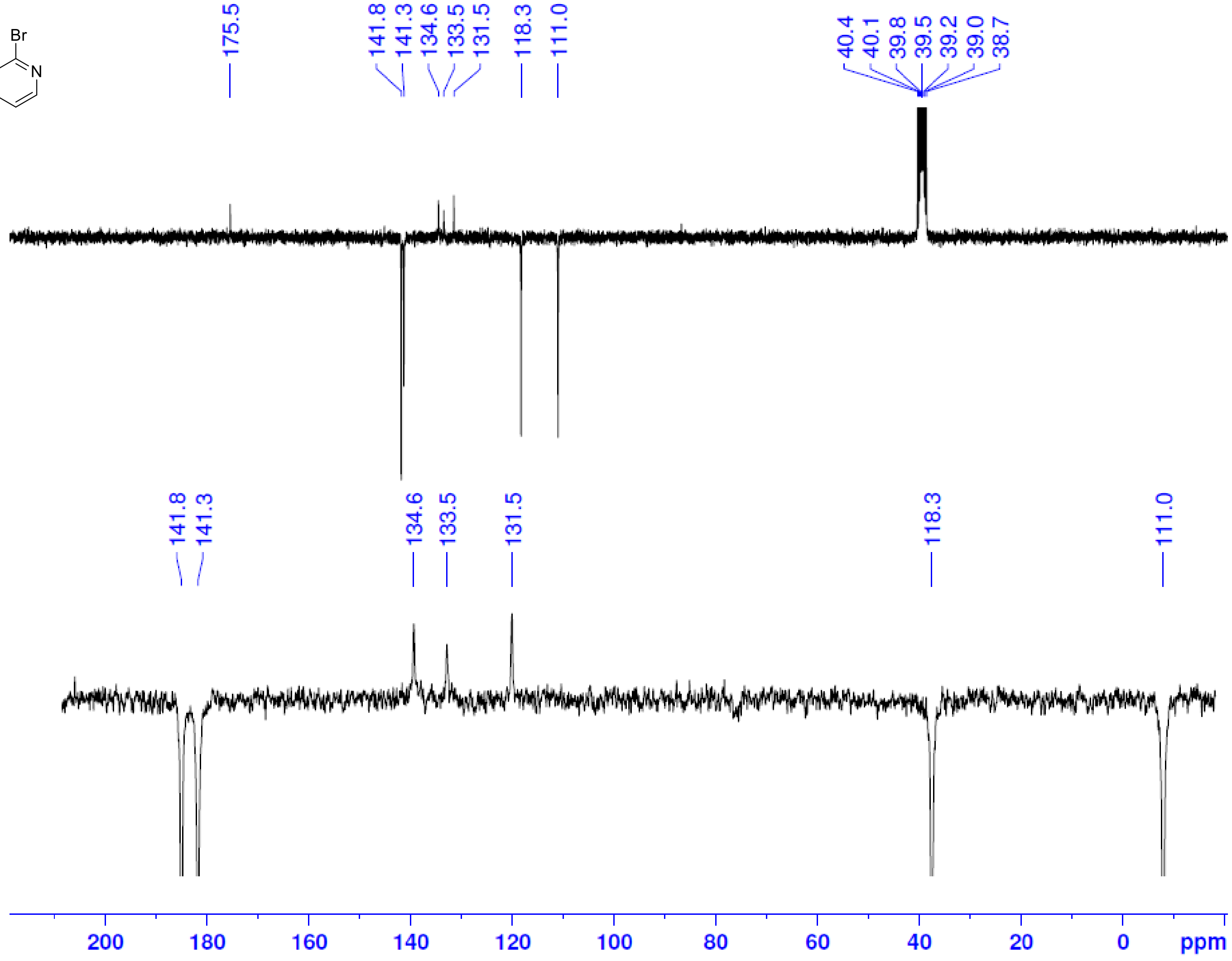

Current Data Parameters
NAME Maria
EXPNO 314
PROCNO 1

F2 - Acquisition Parameters
Date_ 20210311
Time 8.17 h
INSTRUM spect
PROBHD Z104275_0375 (
PULPROG jmod
TD 65536
SOLVENT DMSO
NS 12288
DS 4
SWH 18115.941 Hz
FIDRES 0.552855 Hz
AQ 1.8087935 sec
RG 201.81
DW 27.600 usec
DE 6.50 usec
TE 294.5 K
CNST2 145.0000000
CNST11 1.0000000
D1 2.00000000 sec
D20 0.00689655 sec
TD0 1
SFO1 75.4752953 MHz
NUC1 13C
P1 10.00 usec
P2 20.00 usec
PLW1 41.00000000 W
SFO2 300.1312005 MHz
NUC2 1H
CPDPRG[2 waltz16
PCPD2 80.00 usec
PLW2 8.19999981 W
PLW12 0.25112000 W

F2 - Processing parameters
SI 32768
SF 75.4677840 MHz
WDW EM
SSB 0
LB 1.00 Hz
GB 0
PC 1.40

8-Phenyl-1,7-naphthyridin-4(1*H*)-one (**16**), ^{1}H NMR in $CDCl_3$, 500 MHz

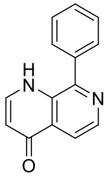

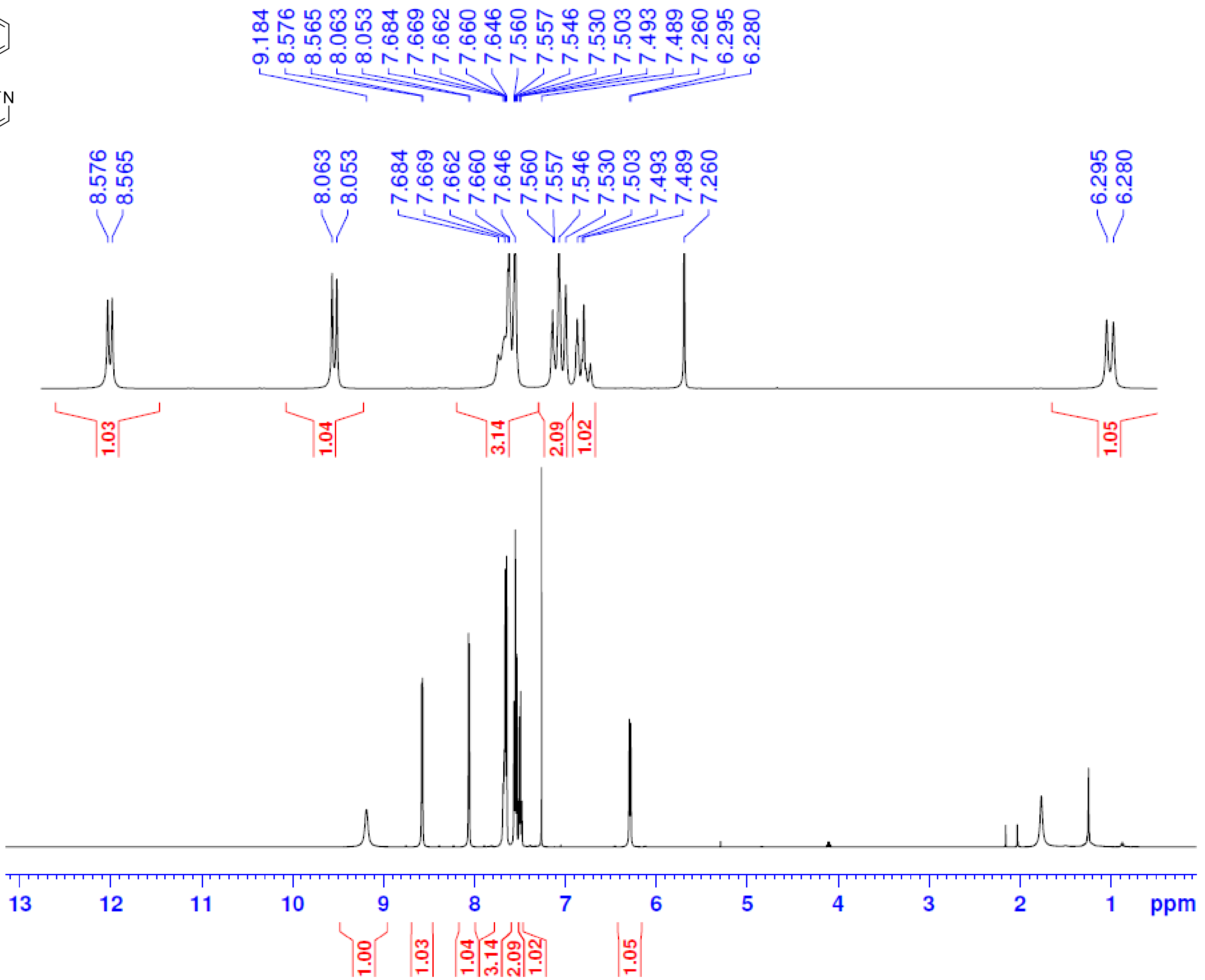

Current Data Parameters
NAME Maria
EXPNO 1108
PROCNO 1

F2 - Acquisition Parameters
Date_ 20210611
Time 11.43
INSTRUM spect
PROBHD 5 mm PABBO BB-
PULPROG zg30
TD 65536
SOLVENT CDCl3
NS 16
DS 2
SWH 10000.000 Hz
FIDRES 0.152588 Hz
AQ 3.2767999 sec
RG 144
DW 50.000 usec
DE 6.50 usec
TE 295.3 K
D1 1.00000000 sec
TD0 1

======== CHANNEL f1 =====
SFO1 500.0361158 MHz
NUC1 1H
P1 12.00 usec
PLW1 14.50000000 W

F2 - Processing parameters
SI 65536
SF 500.0330406 MHz
WDW EM
SSB 0
LB 0.30 Hz
GB 0
PC 1.00

8-Phenyl-1,7-naphthyridin-4(1*H*)-one (**16**), ^{13}C NMR in $CDCl_3$, 125 MHz

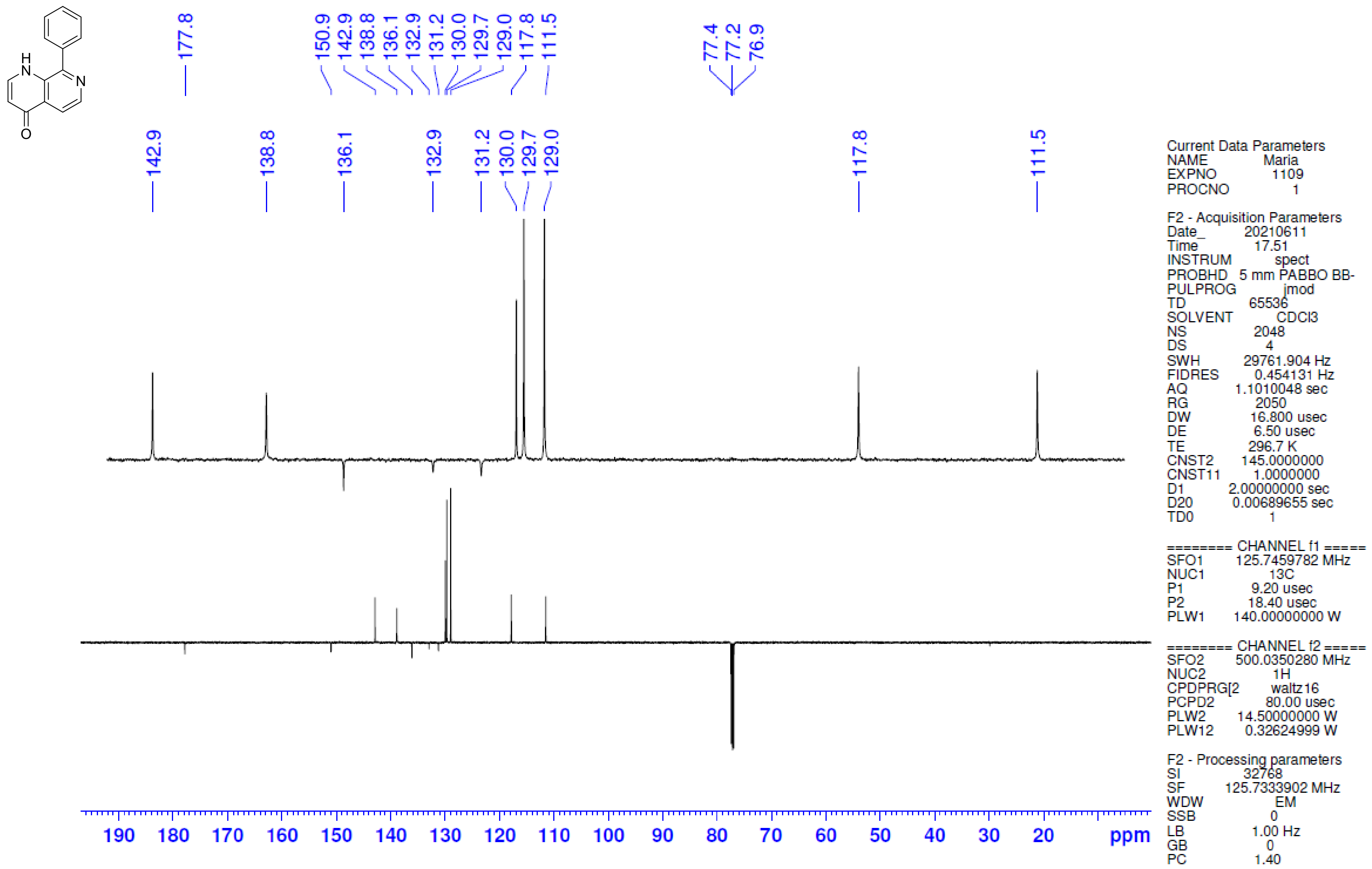

Supplement: Supplementary file 1 — jo4c00440_si_001.pdf [file jo4c00440_si_001.pdf]
